# Priming with a Simplified Intradermal HIV-1 DNA Vaccine Regimen followed by Boosting with Recombinant HIV-1 MVA Vaccine Is Safe and Immunogenic: A Phase IIa Randomized Clinical Trial

**DOI:** 10.1371/journal.pone.0119629

**Published:** 2015-04-15

**Authors:** Patricia. J. Munseri, Arne Kroidl, Charlotta Nilsson, Agricola Joachim, Christof Geldmacher, Philipp Mann, Candida Moshiro, Said Aboud, Eligius Lyamuya, Leonard Maboko, Marco Missanga, Bahati Kaluwa, Sayoki Mfinanga, Lilly Podola, Asli Bauer, Karina Godoy-Ramirez, Mary Marovich, Bernard Moss, Michael Hoelscher, Frances Gotch, Wolfgang Stöhr, Richard Stout, Sheena McCormack, Britta Wahren, Fred Mhalu, Merlin L. Robb, Gunnel Biberfeld, Eric Sandström, Muhammad Bakari

**Affiliations:** 1 Department of Internal Medicine, Muhimbili University of Health and Allied Sciences (MUHAS), Dar es Salaam, Tanzania; 2 Venhalsan, Karolinska Institutet, Sodersjukhuset, Stockholm, Sweden; 3 National Institute for Medical Research—Mbeya Medical Research Center, Mbeya, Tanzania; 4 Department of Infectious Diseases and Tropical Medicine, Klinikum of the University of Munich, Munich, Germany; 5 German Center for Infection Research (DZIF), partner site Munich, Munich, Germany; 6 The Public Health Agency of Sweden, Solna, Sweden; 7 Department of Microbiology, Tumor and Cell biology, Karolinska Institutet, Stockholm, Sweden; 8 Department of Laboratory Medicine, Karolinska Institutet, Huddinge, Sweden; 9 Department of Microbiology and Immunology, Muhimbili University of Health and Allied Sciences (MUHAS), Dar es Salaam, Tanzania; 10 Department of Epidemiology and Biostatistics, Muhimbili University of Health and Allied Sciences (MUHAS), Dar es Salaam, Tanzania; 11 National Institute for Medical Research—Muhimbili Medical Research Centre, Dar es Salaam, Tanzania; 12 Walter Reed Army Institute of Research (WRAIR), Rockville, MD, United States of America and The Henry M. Jackson Foundation, Rockville, MD, United States of America; 13 Laboratory of Viral Diseases, NIAID, NIH, Bethesda, MD, United States of America; 14 Imperial College, London, United Kingdom; 15 Medical Research Council Clinical Trials Unit, University College London, London, United Kingdom; 16 Bioject Medical Technologies, 7180 SW Sandburg St, Tigard, Oregon, United States of America; University of Alabama, UNITED STATES

## Abstract

**Background:**

Intradermal priming with HIV-1 DNA plasmids followed by HIV-1MVA boosting induces strong and broad cellular and humoral immune responses. In our previous HIVIS-03 trial, we used 5 injections with 2 pools of HIV-DNA at separate sites for each priming immunization. The present study explores whether HIV-DNA priming can be simplified by reducing the number of DNA injections and administration of combined versus separated plasmid pools.

**Methods:**

In this phase IIa, randomized trial, priming was performed using 5 injections of HIV-DNA, 1000 μg total dose, (3 Env and 2 Gag encoding plasmids) compared to two “simplified” regimens of 2 injections of HIV-DNA, 600 μg total dose, of Env- and Gag-encoding plasmid pools with each pool either administered separately or combined. HIV-DNA immunizations were given intradermally at weeks 0, 4, and 12. Boosting was performed intramuscularly with 10^8^ pfu HIV-MVA at weeks 30 and 46.

**Results:**

129 healthy Tanzanian participants were enrolled. There were no differences in adverse events between the groups. The proportion of IFN-γ ELISpot responders to Gag and/or Env peptides after the second HIV-MVA boost did not differ significantly between the groups primed with 2 injections of combined HIV-DNA pools, 2 injections with separated pools, and 5 injections with separated pools (90%, 97% and 97%). There were no significant differences in the magnitude of Gag and/or Env IFN-γ ELISpot responses, in CD4+ and CD8+ T cell responses measured as IFN-γ/IL-2 production by intracellular cytokine staining (ICS) or in response rates and median titers for binding antibodies to Env gp160 between study groups.

**Conclusions:**

A simplified intradermal vaccination regimen with 2 injections of a total of 600 μg with combined HIV-DNA plasmids primed cellular responses as efficiently as the standard regimen of 5 injections of a total of 1000 μg with separated plasmid pools after boosting twice with HIV-MVA.

**Trial Registration:**

World Health Organization International Clinical Trials Registry Platform PACTR2010050002122368

## Introduction

The global HIV pandemic is not yet under control despite reported recent decline in incidence [1]. According to the UNAIDS report for the year 2014 there were a total of 35.3 million people living with HIV, 2.1 million new infections, with 69% of all people living with HIV from sub-Saharan Africa and 1.5 million deaths attributed to HIV [2]. The currently available HIV preventive and control interventions require strict adherence to be effective [3,4,5] with a threat of recidivism [6,7]. Therefore there is still a need to prevent and control the large number of new infections by complementing on-going interventions such as early detection, education on behavioral change and biomedical strategies with a safe, affordable and effective preventative HIV vaccine.

The search for an HIV vaccine during the past 25 years has been a challenge due to viral diversity and the ability of the persistently virus—infected cells to evade the immune system [8]. However pre-clinical studies have identified immune and genetic biomarkers associated with protection against challenge that provide further insights for an HIV preventive vaccine for humans [9,10,11,12,13].

So far there have been more than 180 clinical HIV-1 vaccine trials conducted in humans ranging from phase I to phase III [14], including the recently concluded RV 144 phase III trial in Thailand that showed a modest efficacy of 31% [15]. Post-hoc analysis of the RV144 trial evaluating associations between immune responses to vaccine and protection suggests that binding IgG antibodies specific to the variable regions 1 and 2 of the HIV-1 envelope protein are important [16,17,18]. An effective vaccine would be one that is capable of eliciting both antibodies and T cells that have antiviral capabilities [19].

Tanzania is one of the sub-Saharan countries that has been highly affected by HIV, and has participated in early phase I/II HIV vaccine trials [20]. Earlier studies evaluated different routes for HIV-DNA vaccine administration comparing intradermal to intramuscular routes of HIV-DNA delivery [20,21]. We have shown that intradermal priming thrice with 1000 μg of an HIV-DNA vaccine per immunization given as 5 injections of 0.1 ml and separating Env and Gag plasmid pools prior to boosting twice with an HIV-MVA vaccinia vector vaccine was safe and resulted in strong and broad antigen-specific cellular immune responses to HIV Gag and Env [20,22]. Importantly this study also showed that all vaccinees developed binding anti-HIV antibodies, and a high proportion had antibodies reactive in a peripheral mononuclear cell (PBMC) neutralization assay after the second HIV-MVA boost[20,22].

With overall feasibility in mind, it would be ideal to reduce the number of injections and combine the plasmid pools into a single injection. We therefore set out to address two questions. Firstly, could the number of injections be reduced to 2 per immunization, and secondly could the regimen be further simplified by combining the plasmid pools. Due to constraints in the volume that could be delivered intradermally and the highest available DNA concentration we compared the safety and immunogenicity of administering five injections of HIV-DNA at a total dose of 1000 μg (5x0.1mL of 2mg/mL) of separated plasmids (“standard regimen”), with a “simplified regimen” comprising 2 injections of a lower overall dose of 600 μg (2x0.1mL of 3mg/mL). We also compared the effect of combining versus separating the plasmid pools for the overall safety and immunogenicity.

## Materials and Methods

### Ethics Statement

Ethical approval was obtained from the institutional review boards of the Muhimbili University of Health and Allied Sciences, and the Mbeya Medical Research Ethics Committee. The Tanzania National Institute for Medical Research (NIMR), serving as the National Ethics Committee, and the Swedish Ethics Committee also approved the study. The Tanzania Food and Drugs Authority (TFDA) approved the candidate HIV-DNA and HIV-MVA vaccines for use in humans in Tanzania. This study was conducted according to the principles of International Community of Harmonization and Good Clinical Practice guidelines (ICH-GCP). All participants had at least primary level education and non had a compromised ability to consent. All participants were provided with an information sheet to read and were recruited after having signed the study informed consent.

### Study Design and Population

This was a phase IIa randomized clinical trial that recruited participants from two centers in Tanzania: the Muhimbili University of Health and Allied Sciences (MUHAS) in Dar es Salaam, and the National Institute for Medical Research (NIMR)-Mbeya Medical Research Center (NIMR-MMRC) in Mbeya. The study protocol and CONSORT checklist are available as supporting information; see Checklist A and Protocol A in S1 File. The trial is registered at the World Health Organization International Clinical Trials Registry Platform, on the 31^st^ of May 2010 with registration number PACTR2010050002122368 that is available at http://apps.who.int/trialsearch/trial.aspx?trialid=PACTR2010050002122368. The data are filed at the study sponsor and will be made available upon written request to the study Sponsor, the Public Health Agency of Sweden, at registrator@folkhalsomyndigheten.se.

The trial participants were recruited among officers in Police and Prison forces, as well as youths from a Youth friendly clinic in Dar es Salaam. In Mbeya participants were recruited from the general population.

The study protocol aimed to include 120 healthy individuals (60 from each center), aged 18–40 years who were regarded to be at low risk for acquiring HIV infection, and were willing to undergo HIV and pregnancy testing (for females) at screening and during the trial duration of 70 weeks. Participants were also required to use an effective method of contraception throughout the trial.

Replacements were performed for early dropouts or withdrawals from immunization during ongoing recruitment. All participants received detailed study information prior to screening and enrollment during pre-screening and briefing workshops. All participants were required to sign an informed consent form before enrollment. The participants were also required to have undergone and passed a test of understanding about the study prior to enrollment.

Volunteers were excluded if they were HIV-infected, pregnant, had any clinically relevant medical condition or abnormal laboratory findings that included Hepatitis B virus infection, syphilis, diabetes, were known to be using immunosuppressive medications or had baseline ECG findings indicating cardiac disease or likely to complicate later interpretation of peri/myocarditis. Recruitment began on the 10^th^ of June 2010 and the final follow up visit was in June 2012. The study protocol received approval from the local ethics and National ethics committee the 9th of November 2009 and the 31^st^ of December 2009 respectively

### Randomization and Vaccinations

**Fig 1:**
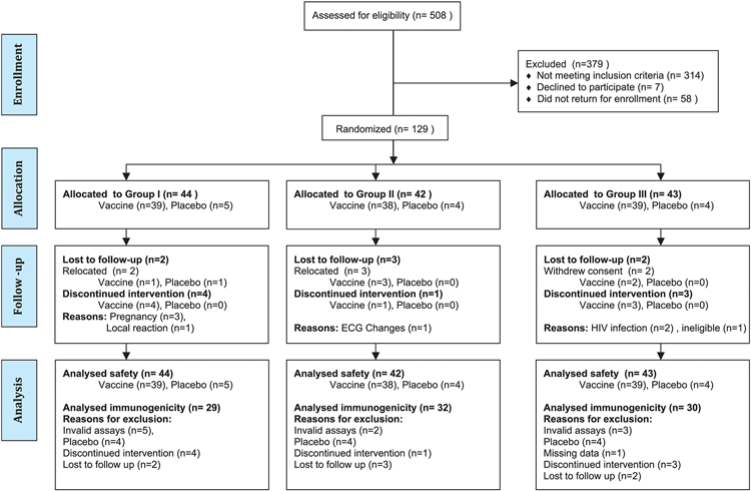
Number of individuals screened, randomized and allocated to the three vaccination groups and withdrawn from the trial.

The trial participants were randomized to one of the three main study groups as summarized in Table 1 and Fig 1: “2 injections combined”, “2 injections separated” and “standard”5 injections separated”. Within each main group participants were block randomized to vaccine or placebo in a ratio of 9:1. Blocks of 10 individuals consisting of 3 in group I, 3 in group II, 3 in group III vaccine and one placebo allocation were randomized by the sponsor using random numbers generated from http://www.random.org/list/. Six blocks were assigned to each site in the form of a list maintained by the study pharmacist at each site. On the request of the clinic the pharmacist allocated the enrolled volunteer to a consecutive slot on the list. The clinic was not informed of vaccine/placebo allocation. Saline was used as a placebo to minimize the risk that participants would mistakenly believe themselves to be protected against HIV. The placebo was also used to blind staff and participants when reporting adverse events. The three sets of intradermal (ID) injections of HIV-DNA/Placebo were administered at weeks 0, 4 and 12. The two intramuscular (IM) boosting vaccinations with HIV-MVA/Placebo were given at weeks 30 and 46. The HIV-DNA/Placebo was administered in doses of 0.1 ml intradermally in the skin over the deltoid muscle using a Biojector needle-less device (Bioject Medical Technologies, Inc., Tualatin, OR, USA). The HIV- MVA/Placebo vaccinations were administered with a needle and syringe in a volume of 1 ml in the left deltoid muscle.

**Table 1:** Randomized study groups, doses and routes of HIV-DNA and HIV-MVA vaccinations.

Group	Number of participants	HIV-DNA/Placebo weeks 0,4 and12		HIV-MVA/Placebo weeks 30 and 46
		Left arm	Right arm	Left arm
IA	36	1 injection ID of 0.1ml (3mg/ml). Pool 1 (EnvABC/RevB) and Pool 2 (GagAB/RTmutB). Total dose 300 μg DNA. Pool 1& Pool 2 combined	1 injection ID of 0.1ml (3mg/ml). Pool 1(EnvABC/RevB) and Pool 2 (GagAB/RTmutB). Total dose 300 μg DNA. Pool 1 and Pool 2 combined	1 injection IM of MVA-CMDR A_E. 1 ml 10^8^ pfu.
IB	4	1injection ID of 0.1ml saline	1injection ID of 0.1ml saline	1 injection IM of 1ml saline
IIA	36	1 injection ID of 0.1ml (3mg/ml). Pool 1 (EnvABC/RevB) separated. Total dose 300 μg DNA	1 injection ID of 0.1ml (3mg/ml). Pool 2 (GagAB/RTmutB) separated. Total dose 300 μg DNA	1 injection IM of MVA-CMDR A_E. 1 ml 10^8^ pfu.
IIB	4	1injection ID of 0.1ml saline	1injection ID of 0.1ml saline	1 injection IM of 1ml saline.
IIIA	36	3 injections ID of 0.1ml (2mg/ml). Pool 1 (EnvABC/RevB) separated. Total dose 600 μg DNA	2 injections ID of 0.1ml (2mg/ml) Pool 2 (GagAB/RTmutB) separated. Total dose 400 μg DNA	1 injection I.M, MVA-CMDR A_E. 1 ml 10^8^ pfu.
IIIB	4	3 injections ID of 0.1 ml saline	2 injections ID of 0.1 saline	1 injection I.M, 1ml saline.

Note: ID = intradermal, IM = intramuscular, pfu = plaque forming units.

Combined refers to a combination of plasmid pools 1 and 2, separated refers to each plasmid pool given separately.

### Vaccines

The HIV-1 DNA (HIVIS DNA) vaccine was manufactured by Vecura, (Huddinge, Stockholm, Sweden). The HIV-DNA vaccine was composed of 7 plasmids carrying different HIV-1 genes. Pool 1 comprised plasmids encoding Env subtypes A, B and C and Rev subtype B while pool 2 comprised plasmids encoding Gag subtypes A, B and RTmut subtype B. A more detailed description of the HIV-DNA vaccine is given by Brave et al [23,24].

The HIV-DNA concentration was 3 mg/ml for groups I and II and 2 mg/ml for group III as summarized in Table 1.

The Modified Vaccinia Ankara-Chiang Mai double recombinant (MVA-CMDR) vaccine was manufactured by WRAIR Pilot Bio production facility (Forest Glen, MD, USA). The HIV-MVA vaccine is a recombinant live non-replicating poxvirus vector that was genetically engineered to express HIV-1 gp150 (Subtype E, isolate CM235) and Gag and Pol (integrase-deleted and reverse transcriptase nonfunctional, Subtype A, isolate CM240), both under control of the early and late mH5 promoter. A description of the MVA-CMDR vaccine is given by Earl et al [25]. Pre-study titration indicated a concentration of up to 10^8^ pfu/ml.

### Safety Assessments

Safety assessments were performed at each visit after the first immunization using an open question, and by soliciting information on adverse events recognized to be associated with licensed vaccines. These were local (pain, redness, swelling and induration) and general (fever, headache, malaise, chills, nausea, vomiting, myalgia and arthralgia). Participants were also asked to make a daily record of these events in a diary card for seven days after each immunization. The diary card was reviewed by the study team and transcribed into the case report form. The study team monitored vital signs before and immediately after each immunization. Routine laboratory parameters (complete blood count, ALT, direct and indirect bilirubin, random blood glucose and creatinine) were collected at 2 weeks after each immunization.

Participants were given instructions on how to fill in the diary card and how to measure temperature, local swelling and grading of the symptoms based on predefined criteria. The study participants were also required to record or report on any medications used during the entire study duration. The clinical and laboratory events were graded for severity as either mild, moderate, severe based on the DAIDS toxicity scale (Division of AIDS, Natl. Institutes of Health) [26] except for neutropenia that was considered as mild with levels of 1100 cells/μl that is lower than the DAIDS scale and was based on the local reference ranges. HIV infection was regarded as a grade 4 event according to the study protocol. The clinical events were defined as solicited or non-solicited events. Each of the clinical events that occurred was evaluated for a relationship to vaccination and was classified as not related, probably not related, possibly related, probably related and definitely related to the study products.

Urinalysis, pregnancy and HIV tests were performed at screening, on the day of each vaccination and during the final visit. All women were required to have a negative pregnancy test at screening and prior to each vaccination.

Participants who were HIV infected or pregnant were stopped from further vaccination but were followed up until the end of the trial or post-delivery for pregnant women. Monitoring for HIV-1 infection was performed using a sequential algorithm of Murex HIV antigen/antibody combination (Diasorin S.p.A, Dartford, UK) and Enzygnost^®^ HIV Integral II (Siemens Health Care, Marburg, Germany) enzyme-linked immunosorbent assays followed by INNO-LIA^™^ HIV I/II immunoblot (Innogenetics, Gent, Belgium) line immunoassay and Amplicor^®^ HIV-1 DNA test version 1.5 (Roche Molecular Systems, Inc., Branchburg, NJ, USA) in case of reactive ELISA results for MUHAS center. For NIMR-MMRC center, HIV testing was performed using Biorad GS HIV-1/HIV-2 PLUS O EIA. If the EIA indicated HIV infection twice, Biorad GS HIV-1 Western Blot, Biorad laboratories GmbH, Munich, Germany was performed, followed by COBAS TaqMan HIV-1 test, v.2.0 Roche. Only cases with detectable HIV-RNA were considered HIV infected, reports to the study clinics were termed HIV infected or not infected to maintain blinding.

A twelve lead electrocardiography (ECG) and troponin tests were performed at screening and 2 weeks after each HIV-MVA vaccination to monitor for possible peri/myocarditis according to US FDA requirements. A panel of two independent experienced cardiologists interpreted the ECGs.

### Immunological Assessments

Whole blood samples for analysis of cell-mediated immune responses were collected in vacutainer tubes containing sodium heparin as anticoagulant. Peripheral blood mononuclear cells (PBMC) were purified using LeucoSep tubes according to the manufacturer´s instructions (Greiner Bio-One).

IFN-γ ELISpot assays were performed using the human IFN-γ ELISpotPLUS kit in a two-step detection system according to the manufacturer’s instructions (Mabtech, Nacka, Sweden) as previously described [20]. Freshly prepared purified PBMCs were used in the assay. Phytohaemagglutinin (PHA, positive control), a peptide pool (CEF) composed of a panel of 23 peptides from cytomegalovirus (CMV), Epstein-Barr virus and influenza virus [27], a peptide pool of 138 peptides (15 mers with an overlap of 11 amino acids) spanning the pp65 protein of human CMV (PepMix, JPT, Berlin, Germany) and HIV-1 specific peptide pools representing MVA-CMDR subtype A Gag and subtype E envelope (Env) were used for stimulation (purity >80%, JPT, Berlin, Germany). A final concentration of 5 μg/ml was used for PHA and CEF. The pp65 CMV peptide pool and the two MVA-CMDR-specific peptide pools were used at 1 μg/ml. RPMI medium without stimuli was used as negative control (background). Fifty microliters cell suspension of PBMC was added to each well giving 2x10^5^ cells/well. The plates were incubated for 20 hours at 37°C, with 7.5% CO_2_.

Frequencies of antigen-specific spot-forming cells (SFC) were measured using an automated Immunospot analyzer (CTL-Europe, Bonn, Germany). Results were expressed as SFC per million PBMCs and were calculated for each pool of peptides as follows: 5 x the mean SFC from three stimulated wells, without subtracting background. ELISpot responses were considered positive if the number of SFC was >55 spots/10^6^ PBMC and at least 4 times the background value. Data where background responses in three medium wells exceeded a median of 60 per million PBMCs were excluded from analyses.

For the determination of CD4^+^ and CD8^+^ T cell responses, a 4-colour ICS assay was performed on fresh PBMC which had been rested overnight following the purification procedure as detailed previously [20]. 4-color ICS testing was performed on samples from only one center (MUHAS). Briefly, PBMC were incubated with co-stimulatory anti-CD28 (1 μg/ml) and anti-CD49d (1 μg/ml) monoclonal antibodies (Becton Dickinson, BD Pharmingen, San Diego, CA), in either medium only (negative control) or in medium containing stimuli and brefeldin A (0.5 μg/ml) (Sigma, St. Louis, MO). Staphylococcal enterotoxin A and B (SEAB, 1 μg/ml) (positive control) (Sigma, St. Louis, MO), a CEF peptide pool (1μg/ml), a CMV peptide pool (PepMix, 0.5μg/mL), two HIV-1 Gag-specific peptide pools representing the HIV-DNA vaccine or MVA-CMDR vaccine and a HIV-1 Env-specific peptide pool representing MVA-CMDR were used for stimulation. The cells were incubated for 6 hours at 37°C in a 7.5% CO_2_ incubator and the stimulated cells were stored at 4°C overnight prior to staining with an antibody cocktail containing anti-CD3–APC, anti-CD4–FITC, anti-CD8–PerCpCy5.5, anti-IFN-γ-PE and anti-IL-2-PE (Becton Dickinson, San Jose, CA). Acquisition of samples was performed using a FACSCalibur flow cytometer and samples were analyzed using FlowJo software, version 8.7.1 (Tree Star, Ashland, OR). A minimum of 50,000 CD3^+^ lymphocytes per well was required for a sample to be included in the analysis. Background reactivity to Gag and Env peptide pool stimulation was defined using pre-immunization samples collected from the vaccinees. ICS responses were considered positive if they were at least 2.5-fold higher than the mean of background (medium samples) and above 0.05% for CD4^+^ T lymphocytes and above 0.1% for CD8^+^ T lymphocytes.

Binding antibodies to native gp160 (IIIB, Advanced Biotechnologies Inc.) were tested using a standardized enzyme-linked immunosorbent assay (ELISA). Starting from a dilution of 1:100, two-fold dilution steps were employed to determine antibody titres [22].

All safety and immunological laboratory tests were performed at the Department of Microbiology and Immunology at the MUHAS and at the MMRC main laboratories at the respective centers. These two laboratories implement strict internal quality control programs and participate in external proficiency testing programs including College of American Pathologists (CAP), United Kingdom National External Quality Assurance Scheme (UKNEQAS) and USA Virology Quality Assurance (VQA).

### Study Endpoints

#### Primary safety endpoints

The primary safety endpoint was defined as any grade 3 and above clinical or laboratory adverse event that occurred after the first immunization up until 24 weeks from the last immunization.

#### Secondary safety endpoints

The secondary safety endpoint was defined as any grade 1 or 2 clinical or laboratory adverse event that occurred after the first immunization up until 24 weeks from the last immunization.

#### Primary immunogenicity endpoint

The primary immunogenicity end point was assessed as positive Interferon-γ ELISpot responses to either Gag and/or Env peptide pools 2 weeks after the second HIV-MVA vaccination in participants that completed the immunization regimen.

#### Secondary immunogenicity endpoints

Secondary immunogenicity endpoints were evaluated as the magnitudes of the IFN-γ ELISpot responses to Gag or Env peptide pool stimulation determined two weeks after the first and second HIV-MVA vaccination, the proportion of 4-colour ICS IFN-γ/IL-2 responders and the magnitude of IFN-γ/IL-2 responses to Gag and Env peptide pool stimulations two weeks after the first and second HIV-MVA vaccination, as well as the antibody responses to HIV-1 subtype B gp160 as determined by ELISA in samples collected four weeks after the second HIV-MVA vaccination.

### Statistical Methods

Assuming a response rate of 100% in the group that received the ‘standard regimen’ of 5 injections with a total dose of 1000 μg HIV-DNA x 3 followed by HIV-MVA boosts, we required 36 participants in each group to be able to detect a 20% difference in the groups that received the simplified HIV-DNA regimen with a power of 80% and 5% significance level.

Clinical and laboratory safety data were recorded in study specific case report forms and entered twice into the study database which was programmed in SQL. Discrepancies between the data records were resolved before the data files were extracted for analysis. Immunological data were exported directly into Excel from the ELISpot reader and thereafter into STATA version 11 for analysis.

Datasets were created for analysis as follows: The safety analysis dataset included all solicited, non-solicited and routine laboratory data that were collected after the first vaccination up to 24 weeks after the 2^nd^ HIV-MVA/Placebo vaccination. The solicited and non-solicited events were summarized according to the maximum grade of severity as mild, moderate or severe. Comparisons of the proportion of participants with solicited and non-solicited events were made between the randomization groups in relation to the HIV-DNA and HIV-MVA vaccinations. Routine laboratory data were summarized as the number of events by grades and proportion of participants with events by grades in relation to the randomization group.

The immunological analysis dataset was limited to participants who completed all the scheduled immunizations and included all responses to interferon-γproduction by ELISpot assay to Gag-CMDR and/or Env-CMDR peptides, measured as the proportion of participants with a valid assay that had ELISpot responses and the median magnitude of these responses by peptide pool.

We compared the proportion of responders in the 2 injection-separated-plasmid group to the standard 5 injection-separated-plasmid group. We thereafter compared the proportion of responders in the 2 injection—combined-plasmid group to the 2 injection-separated-plasmid group. Finally we compared the proportion of responders in the 2 injection-combined-plasmid-group to the standard 5 injection-separated-plasmid group.

Comparisons between proportions were made using chi-square test or Fisher’s exact test where appropriate, and no adjustments were made for multiple comparisons. A two sided p- value of <0.05 was considered to be statistically significant.

The magnitudes of ELISpot responses were measured two weeks after the first HIV-MVA and two weeks after the second HIV-MVA. The magnitude of responses in responding individuals only was described using median and interquartile range (IQR), and comparisons between the randomization groups were made by Wilcoxon rank-sum test.

The data were analyzed using STATA version 11 (StataCorp LP 1985–2011, College, Station, TX, USA).

## Results

### Demographics, Recruitment and Inclusion

Overall, 743 volunteers attended the briefing and pre-screening sessions, of whom 508 (68%) proceeded to a screening visit between March 2010 and June 2011. Of those screened, 129 (25%) were enrolled and these individuals were balanced across the randomization groups for age, sex and center (Table 2). Scars compatible with previous vaccinia vaccination were observed in 7% (9/129) of the enrollees whose age range was 29–38 years.

**Table 2:** Baseline characteristics.

Characteristics	Group I.	Group II	Group III	Placebo	Total
	2 injections, 600 μg HIV-DNA combined pools	2 injections, 600 μg HIV-DNA separated pools	5 injections, 1000 μg HIV-DNA separated pools		
	n = 39	n = 38	n = 39	n = 13	n = 129
Site					
MMRP	20 (51)	20 (53)	20 (51)	7 (54)	67 (52)
MUHAS	19 (49)	18 (47)	19 (49)	6 (46)	62 (48)
Sex					
Female	16 (41)	16 (42)	15 (38)	7 (54)	54 (42)
Male	23 (59)	22 (58)	24 (62)	6 (46)	75 (58)
Age (years)	23 (20–29)	23 (20–27)	26 (20–31)	22 (19–24)	23 (20–29)
BMI (kg/m^2^)	22 (20–25)	22 (20–24)	23 (20–26)	25 (20–26)	22 (20–26)
Laboratory					
Hemoglobin (g/dl)	15 (13–16)	15 (12–15)	15 (14–16)	14 (12–16)	15 (13–16)
White cell count (10^9^ cells/l)	4.8 (3.8–5.5)	4.7 (3.9–5.3)	4.9 (4.3–5.8)	5.2 (3.9–6.1)	4.8 (4.0–5.6)
Neutrophils (10^9^ cells/l)	2.0 (1.5–2.8)	2.3 (2.0–2.7)	2.4 (1.8–3.0)	2.4 (2.1–2.6)	2.2 (1.8–2.8)
Lymphocytes (10^9^ cells/l)	2.0 (1.6–2.3)	1.7 (1.4–2.2)	2.0 (1.5–2.4)	2.1 (1.8–2.6)	1.9 (1.5–2.3)
Platelets (10^9^ cells/l)	240 (197–275)	258 (199–305)	277 (227–315)	271 (215–322)	258 (211–311)
CD4^+^ cell count (10^6^ cells/l)	793 (660–955)	694 (589–856)	779 (652–958)	857 (686–971)	784 (630–924)
ALT (U/L)	18 (14–23)	18 (14–25)	16 (13–24)	15 (11–19)	17 (13–23)
Total Bilirubin (μmol/l)	8 (5–11)	6 (4–11)	8 (6–10)	7 (4–10)	8 (5–11)
Direct Bilirubin (μmol/l)	2 (2–3)	2 (1–3)	2 (2–3)	2 (2–3)	2 (2–3)
Creatinine (μmol/l)	59 (53–69)	58 (50–66)	60 (49–66)	51 (43–71)	59 (51–67)
Glucose (mmol/l)	4.3 (4.1–5.1)	4.5 (4.2–4.9)	4.5 (4.1–5.0)	4.4 (4.2–5.0)	4.4 (4.1–4.9)
Follow up (weeks)	65 (61–68)	66 (61–69)	66 (62–69)	66 (61–70)	66 (61–69)

Note: Values are numbers (%) or medians (interquartile ranges).

Combined refers to a combination of plasmid pools 1 and 2, separated refers to each plasmid pool given separately.

The overall briefing to screening to enrollment ratio was 6:4:1. Three hundred and seventy nine participants were screened out, and more than one reason could be given. Frequent reasons for screen out included laboratory abnormalities, 102 (27%), medical reasons, 76 (20%), inability to comply with the study schedule, 61 (16%), not returning for enrolment, 41 (11%), failed test of understanding, 39 (10%), and high risk behaviour related to HIV acquisition, 25 (7%). Of note, ECG abnormalities in otherwise healthy volunteers contributed to the screen out in 32 participants (8%). The HIV prevalence among those screened was 1% (3/235) for Dar es Salaam and 6% (16/273) for Mbeya.

### Withdrawals/Termination from Vaccination

The retention and adherence to the study schedule by the participants is shown in Fig 1. Nineteen participants did not complete the study schedule (not shown in Fig 1). Of these, 15 participants did not complete the vaccination schedule. As a result nine participants were replaced in the randomization scheme during recruitment. The reasons for early vaccination terminations included: not meeting the eligibility criteria due to an abnormal ECG (1), pregnancy (3), lost to follow up (5), consent withdrawn (2), HIV infection (2). Vaccinations were discontinued in (2) participants following adverse events. One had moderate itching and excoriation at the injection site that occurred after the 2^nd^ HIV-DNA vaccination and the other had non-specific ST changes on ECG after the 1^st^ HIV-MVA vaccination. He had no clinical symptoms, the troponin test was negative and echocardiography did not suggest peri/myocarditis. Four other participants relocated and could not continue visits after completing the vaccination schedule. The last study visit was completed on the 13^th^ of June 2012.

### Safety and Tolerability

#### Solicited adverse events

**Fig 2:**
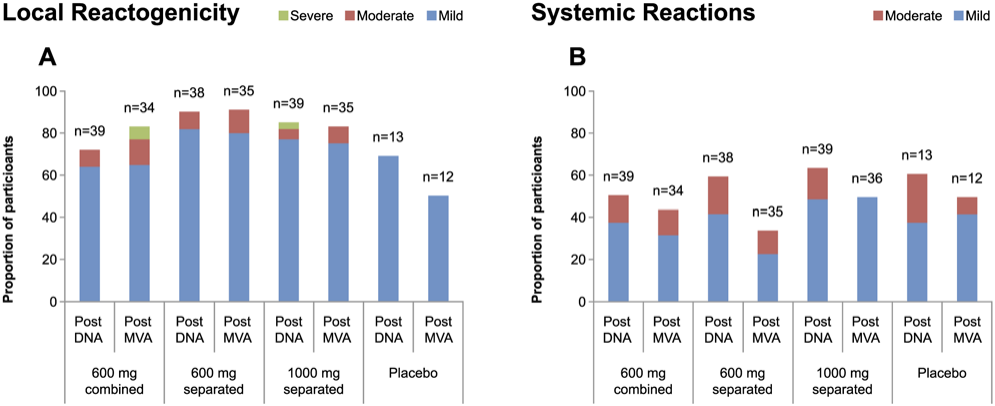
Proportion and number of participants with (A) local and (B) systemic solicited adverse events post HIV-DNA and HIV-MVA by randomization arm.

Of the 129 participants who received at least 1 immunization, 114 (88%) reported a local solicited event and 87 (67%) a solicited systemic adverse event that started within one week of an immunization. The majority of these events were mild, and the distribution by randomization groups and immunogens was similar as summarized in Fig 2a and 2b for local and systemic adverse events respectively. The most common solicited local event was pain, which occurred in 89 (69%) participants, three of whom experienced short duration severe pain (1 post HIV-DNA, 2 post HIV-MVA). The most common systemic solicited event was headache that occurred in 70 (54%) participants. In one participant (given 2 injections of combined HIV-DNA pools) moderate itching and excoriations after two HIV-DNA vaccinations was observed and the participant was withdrawn from further vaccination.

#### Non-solicited adverse events

One hundred and seven participants reported 364 non-solicited clinical adverse events, of which 270 (74%) were mild and 86 (24%) were moderate. Eight (2%) were considered severe and three of these met the protocol criteria for a serious adverse event (SAE). Two were HIV infections (both given the standard regimen of 5 injections with separated HIV-DNA pools), detected at the time of the 2^nd^ HIV-MVA boost. Based on retrospective HIV-DNA analysis (Gen-Probe Aptima HIV-1) the infection time point for both participants was possibly around the 1^st^ HIV-MVA boost. The third SAE was a skull fracture complicated by osteomyelitis (600 μg HIV-DNA combined group). Five other severe events that did not meet the criteria for SAE included: a left axilla abscess (placebo group), a dislocated knee associated with fainting (standard dose separate), and two participants with anemia (low dose separate and placebo group). None of these events were considered to be related to the vaccines.

Of the 364 non-solicited clinical events, 101 started within 2 weeks of an immunization, 75 occurred within 2 weeks of the HIV-DNA/placebo vaccinations, and 26 occurred within 2 weeks of the HIV-MVA/placebo vaccinations. The distribution by randomization groups was similar as summarized in Table 3.

**Table 3:** Numbers and percent of non-solicited clinical adverse events by grade and relationship to vaccination.

Relationship	Adverse events											
	Mild				Moderate				Severe			
	Group I	Group II	Group III	Placebo	Group I	Group II	Group III	Placebo	Group I	Group II	Group III	Placebo
	2 injections600 μg HIV-DNA combined	2 injections600 μg HIV-DNA separated	5 injections 1000 μg HIV-DNA separated		2 injections 600 μg HIV-DNA combined	2 injections 600 μg HIV-DNA separated	5 injections 1000 μg HIV-DNA separated		2 injections, 600 μg HIV-DNA combined	2 injections 600 μg HIV-DNA separated	5 injections 1000 μg HIV-DNA separated	
	N = 84	N = 85	N = 80	N = 21	N = 17	N = 25	N = 31	N = 13	N = 2	N = 0	N = 4	N = 2
Not related	50 (60)	48 (56)	48 (60)	5 (24)	12 (71)	12 (48)	12 (39)	4 (31)	1 (50)	0	1 (25)	0
Probably not related	33 (39)	35 (41)	31 (39)	16 (76)	4 (24)	13 (52)	18 (58)	9 (69)	1 (50)	0	3 (75)	2 (100)
Possibly related	1 (1)	1 (1)	1 (1)	0	1 (6)	0	1 (3)	0	0	0	0	
Probably related	0	1 (1)	0	0	0	0	0	0	0	0	0	

Note: Values represent numbers (%).

The majority of the adverse events, 194 (53.3%), were ‘not related’ to the vaccines, 165 (45.3%) were considered ‘probably not related’ to the vaccines, 5 (1.4%) were considered ‘possibly related’ to the vaccines and 1 (0.3%) was considered as ‘probably related’ to the vaccines.

The five adverse events that were considered possibly related to vaccinations were: altered menstruation cycles (2), herpes zoster (1), herpes labialis (1) and allergic conjunctivitis (1).

Of the non-solicited adverse events, infections were the most commonly reported. There were 174 (48%) infections with the most common infections being: malaria, 51(14%), gastroenteritis, 16 (4%), upper respiratory tract infections, 15 (4%) and tonsillitis, 15(4%). Other non-infectious non-solicited events that were frequently reported were headache, which was reported in 25 participants (7%) and anemia requiring iron supplementation in 22 participants (6%).

#### Laboratory adverse events

There were 287 laboratory adverse events detected according to the DAIDS grading that occurred in 126 participants. Sixty-one (21%) events were detected at safety visits within 2 weeks of an immunization in 41 (33%) participants. Of the 287 laboratory events 198 (69%) were mild, 67 (23%) were moderate, 15 (5%) were severe and 7 (2%) were of grade 4 severity. There was no difference in the proportion of participants with laboratory events between the randomization groups including placebo (data not shown).

Of the 22 grade 3 and above events that occurred in 12 participants, 1 event (neutropenia) occurred within 2 weeks of vaccination. The majority, 15 (68%), of the severe laboratory events were related to asymptomatic neutropenia, which occurred 12 to 24 weeks after the last vaccination and were evenly distributed across the randomization groups.

One male participant (given 2 injections of combined HIV-DNA pools) presented with severe elevation of ALT levels 1 month after the 2^nd^ HIV-MVA. This was associated with transient excessive alcohol intake that resolved after counseling and abstinence with subsequent normalization of the ALT levels.

### Immunogenicity

#### IFN-γ ELISpot responses in the three study groups

The IFN-γ ELISpot response rates to Gag and/or Env peptides two weeks after the second HIV-MVA boost were very high and did not differ significantly between the groups primed with 2 injections of combined HIV-DNA plasmid pools, 2 injections of separated plasmid pools, and 5 injections of separated plasmid pools (response rates 90%, 97% and 97% respectively, Table 4). There was no significant difference in response rates to Env after the second HIV-MVA boost. However, after the first HIV-MVA boost, there was a higher proportion of Env responders in the group given 2 injections of separated plasmid pools compared to the group given 5 injections of separated plasmid pools (p = 0.037).

**Table 4:** Proportion of ELISpot responders to Gag and/or Env peptides two weeks post first and second HIV-MVA vaccination in each of the HIV-DNA primed groups.

	Randomization group					
Peptide Pool	Group I	Group II	Group III	P-value (95% CI)	P-value (95% CI)	P-value (95% CI)
	2 injections, 600 μg HIV-DNA combined	2 injections, 600 μg HIV-DNA separated	5 injections, 1000 μg HIV-DNA separated	Group (I vs III)	Group (II vs III)	Group (I vs II)
	n (%)	n (%)	n (%)			
**2 weeks after 1** ^**st**^ **HIV-MVA**						
Gag-CMDR	24/28 (86)	26/30 (87)	27/31 (87)	1.00 (-18.9, 16.1)	1.00 (-17.4, 16.5)	1.00 (-18.7, 16.8)
Env-CMDR	18/27 (67)	24/29 (83)	18/31 (58)	0.50 (-16.3, 33.5)	**0.037** (2.5, 46.8)	0.17 (-38.6, 6.4)
Gag and/or Env-CMDR	25/28 (89)	29/30 (97)	28/31 (90)	1.00 (-16.5, 14.4)	0.61 (-5.9, 18.6)	0.34 (-20.5, 5.8)
**2 weeks after 2** ^**nd**^ **HIV-MVA**						
Gag-CMDR	22/29 (75)	29/32 (91)	25/30 (83)	0.48 (-28.0, 13.0)	0.47 (-9.4, 24.0)	0.12 (-33.3, 3.8)
Env-CMDR	23/28 (82)	21/30 (70)	25/30 (83)	1.00 (-20.7, 18.3)	0.22 (-34.5,7.8)	0.28 (-9.5, 33.8)
Gag and/or Env-CMDR	26/29 (90)	31/32 (97)	29/30 (97)	0.35 (-19.8, 5.8)	1.0 (-8.6,9.0)	0.34 (-19.8, 5.4)

**Fig 3:**
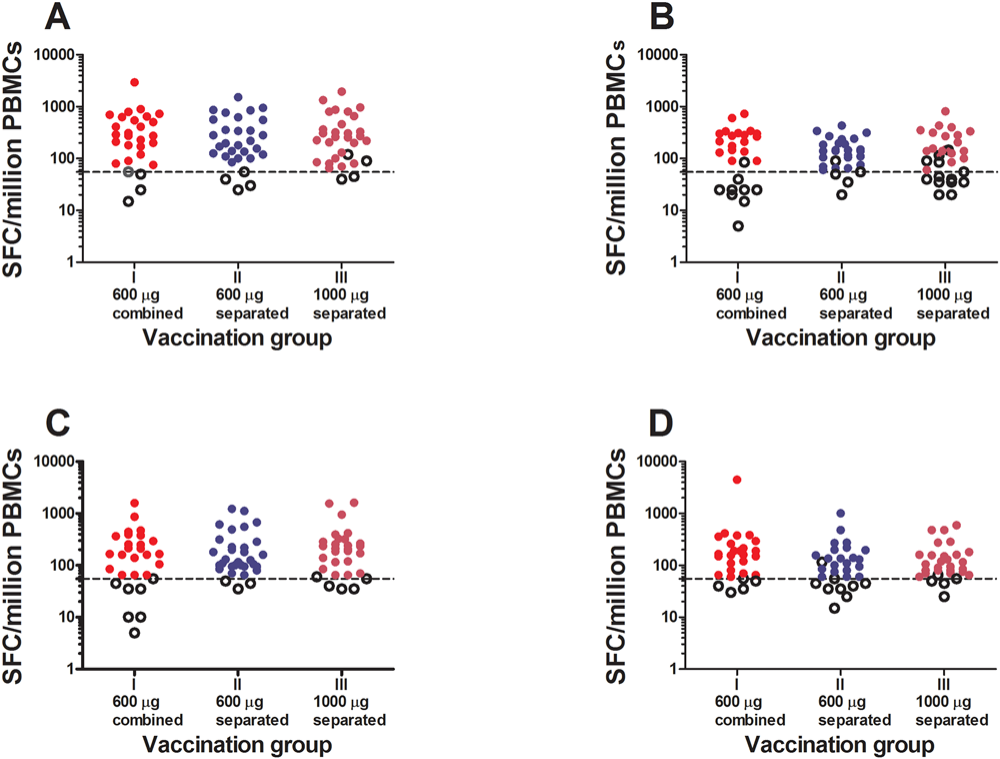
Magnitude of IFN-γ ELISpot responses. The magnitude of IFN-γ ELISpot responses to (A) Gag and (B) Env two weeks after the first HIV-MVA vaccination and to (C) Gag and (D) Env two weeks after the second HIV-MVA vaccination. Data is shown for each of the HIV-DNA primed groups. ELISpot responses were considered positive if the number of SFC was >55 spots/million PBMCs and 4 times the background value. The dashed line is at a cut-off of 55 SFC/million PBMCs. Responders are shown by filled circles and non-responders are shown by open circles.

The overall magnitude of IFN-γ ELISpot responses in responders to Gag was significantly higher after the first than after the second HIV-MVA (median 290 vs 200 SFC/million PBMCs, p<0.001, calculated from but not shown in Table 5) while the magnitude of Env responses after the first and second HIV-MVA was not significantly different (medians 205 vs 155 SFC/million PBMCs, p = 0.15). The magnitude of IFN-γ ELISpot responses to Gag and Env are shown in Fig 3 and Table 5. The magnitude of IFN-γ ELISpot responses to Gag or Env was not significantly different between the three study groups after the second HIV-MVA. However the median of SFC/million PBMCs to Env was higher after the first HIV-MVA boost in the group given the 2 injections of combined plasmid pools compared to that found in vaccinees receiving 2 injections of separated plasmid pools (p = 0.017).

**Table 5:** Magnitude of ELISpot responses measured as SFC/10^6^ PBMCs 2 weeks after the first and second HIV-MVA vaccination in each of the HIV-DNA primed groups.

Time point 2 weeks after	Peptide pool	Randomization group			P value
		Group I	Group II	Group III	Group II vs group III*; Group I vs group II**; Group I vs group III***
		2 injections 600 μg HIV-DNA combined Median (IQR)	2 injections 600 μg HIV-DNA separated Median (IQR)	5 injections1000 μg HIV-DNA separated Median (IQR)	
1^st^ HIV-MVA	Gag-CMDR	300 (190–640)	250 (135–565)	270 (130–660)	*p = 0.90; **p = 0.50; ***p = 0.66
	Env-CMDR	270 (145–310)	143(105–215)	180 (140–335)	*p = 0.12; **p = 0.017; ***p = 0.60
2^nd^ HIV-MVA	Gag-CMDR	213 (140–375)	130 (100–285)	235 (140–320)	*p = 0.18; **p = 0.37; ***p = 0.87
	Env-CMDR	165 (110–295)	135 (85–200)	115 (80–160)	*p = 0.72; **p = 0.24; ***p = 0.15

Two out of 13 placebo recipients were positive in the IFN-γ ELISpot assay (not included in the analysis). One placebo recipient was positive on one occasion out of a total of seven time points. Another placebo recipient was sporadically positive and exhibited reactivity to either Gag or Env peptides on four of seven occasions.

#### CD4^+^ and CD8^+^ T cell responses in the three study groups

**Fig 4:**
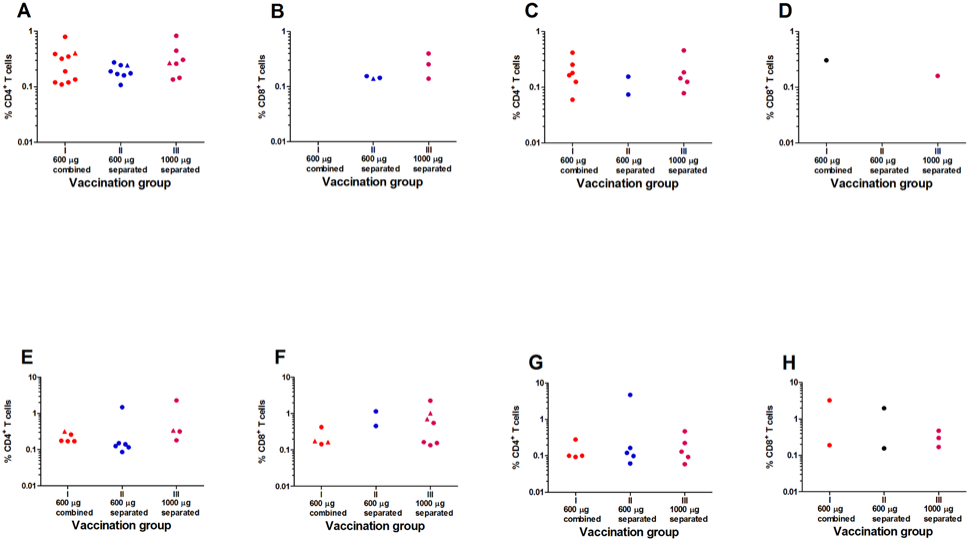
Magnitude of HIV-specific IFN-γ/IL-2 T cell responses. The magnitude of HIV-specific IFN-γ/IL-2 T cell responses in responders as assessed by 4-colour ICS two weeks after the first (upper panel) and second (lower panel) HIV-MVA vaccination presented as Gag reactivity of CD4^+^ T cells (panels A and E) and of CD8^+^ T cells (panels B and F), and Env reactivity of CD4^+^ T cells (panels C and G) and of CD8^+^ T cells (panels D and H). The Gag-reactivity represents reactivity to either the MVA-CMDR-specific peptide pools or the HIV-DNA-specific peptide pool. A Gag HIV-DNA-specific peptide pool response is only given when the Gag MVA-CMDR-specific peptide pool response is negative. Gag-HIV-DNA-specific responses are given by triangles, while Gag and Env-HIV-CMDR-specific responses are given by circles.

The overall proportion of vaccinees with CD4^+^ T cell responses to Gag and/or Env measured as IFN-γ/IL-2 producing cells by ICS was not significantly different two weeks after the first as compared to two weeks after the second HIV-MVA boost, 51% (23 of 45) and 47% (21 of 45), respectively (p = 0.67, calculated from but not seen in Table 6). However, the overall CD8^+^ T cell response rate to Gag and/or Env was significantly higher after the second HIV-MVA boost, 39% (17 of 44), compared to 18% (8 of 44) after the first MVA boost (p = 0.029). The proportion of vaccinees with CD4^+^ and CD8^+^ T cell responses to Gag and/or Env in the three study groups is shown in Table 6. The CD4^+^ T cell response rates to Gag and/or Env peptides were not significantly different between the two groups given the simplified 2 injection-HIV-DNA regimens or between any of the two groups and the group given the standard 5 injection-HIV-DNA regimen. Neither was there a difference in CD8^+^ T cell response rates to Env. However, there was a trend towards a higher CD8^+^ T cell response rate to Gag in the group receiving 5 injections of separated HIV-DNA plasmids compared to the group given 2 injections of separated HIV-DNA plasmids after the second HIV-MVA boost (p = 0.05, Table 6). The magnitudes of CD4^+^ and CD8^+^ T cell responses to Gag and Env in the three groups are shown in Fig 4. Placebo recipients did not show any reactivity in the 4-color ICS assay.

**Table 6:** The proportion of ICS responders to Gag or Env peptide pools two weeks after the first and second HIV-MVA vaccination in each of the HIV-DNA primed groups.

		CD4^+^ Responses				CD8^+^ Responses			
		Randomization group				Randomization group			
Time point 2 weeks after	Peptide pool	Group I	Group II	Group III	P-value: (II vs III)*; (I vs II)**; (I vs III)***	Group I	Group II	Group III	P-value: (II vs III)*; (I vs II)**; (I vs III)***
		2 injections, 600 μg HIV-DNA combined	2 injections, 600 μg HIV-DNA separated	5 injections, 1000 μg HIV-DNA separated		2 injections, 600 μg HIV-DNA combined	2 injections, 600 μg HIV-DNA separated	5 injections, 1000 μg HIV-DNA separated	
		n (%)	n (%)	n (%)		n (%)	n (%)	n (%)	
1^st^ HIV-MVA	Any Gag*	10/15(67)	8/15 (53)	7/17 (41)	0.49*	0/15 (0)	3/15 (20)	3/17 (18)	1.00*
					0.46**				0.22**
					0.15***				0.23***
	Env-CMDR	6/15 (40)	2/13 (15)	5/17 (29)	0.43*	1/15 (7)	0/13 (0)	1/17 (6)	1.00*
					0.22**				1.00**
					0.53***				1.00***
	Any Gag or Env	10/15 (67)	8/15 (53)	7/17 (41)	0.49*	1/15 (7)	3/15 (20)	4/17 (24)	1.00*
					0.46**				0.60**
					0.15***				0.34***
2^nd^ HIV-MVA	Any Gag*	5/13 (38)	6/17 (35)	4/16 (25)	0.71*	4/13 (31)	2/17 (12)	7/15 (47)	0.05*
					1.00**				0.36**
					0.69***				0.39***
	Env-CMDR	4/13 (31)	5/17 (29)	5/16 (31)	1.00*	2/13 (15)	2/17 (12)	3/15 (20)	0.65*
					1.00**				1.00**
					1.00***				1.00**
	Any Gag or Env	6/13 (46)	7/17 (41)	8/17 (47)	0.73*	5/13 (38)	4/17 (24)	8/16 (50)	0.11*
					0.79**				0.44**
					0.96**				0.53***

#### Antibody response in the three study groups

**Fig 5:**
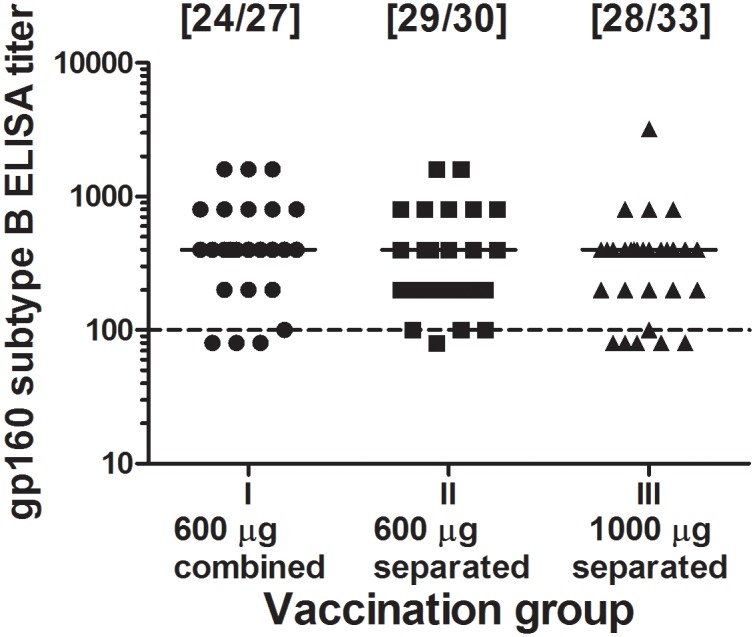
Antibody endpoint titers. Antibody endpoint titers to native HIV-1_IIIB_ subtype B gp160 one month after the second HIV-MVA vaccination. Data is shown for each of the HIV-DNA priming groups. The dashed line shows a titer of 100, corresponding to a 1:100 serum dilution, the lowest dilution used in the assay.

Antibodies to native HIV-1 gp160 were observed in 81 of 90 (90%) of the evaluable vaccinees 4 weeks after the 2^nd^ HIV-MVA boost. The response rate was similar in the three groups; 89% (24 of 27), 97% (29 of 30) and 85% (28 of 33), respectively (Fig 5). The median antibody titer was 400 in all three groups.

## Discussion

This phase IIa HIV-1 vaccine immunogenicity trial builds on the previous HIVIS 03 phase I/II trial that used the same multigene, multiclade HIV-DNA prime and HIV-MVA boost antigens which demonstrated that HIV-DNA at a priming dose of 1000 μg given intradermally elicited a higher and broader cellular immune response compared to a priming dose of 3800 μg of HIV-DNA given intramuscularly [20]. The 1000 μg of HIV-DNA was administered in five different injections due to the limited volume that could be administered intradermally using the Bioject needle-free device.

In the current trial we have shown that the regimen can be simplified by reducing the number of injections and by combining the HIV-DNA plasmids before boosting with the vaccinia-based immunogen, HIV-MVA. We considered the five intradermal HIV-DNA injections with a total of 1000 μg DNA to be the “standard” regimen, and compared this to two intradermal HIV-DNA injections using a higher HIV-DNA concentration of 3mg/ml, leading to 600 μg in total, termed the “simplified” regimen.

We also assessed the effect on the immune response of combining the HIV-DNA plasmids into a single injection compared to separating the Gag and Env plasmid pools in two different injections, since immunocompetition between Gag and Env antigens has been previously observed in mouse experiments [24].

In accordance with what has been reported previously, there were no major safety concerns with the use of HIV-DNA and recombinant HIV-MVA candidate vaccines and the events that occurred were mild and tolerable, irrespective of the number of injections [20,21].

Two participants were discontinued from further immunizations due to adverse events. One of them had ECG changes following the first HIV-MVA, but these were shown to be of no clinical significance following further investigations. The other had moderate itching following the first and second HIV-DNA vaccinations. However these events were not considered sufficiently severe to interrupt a licensed vaccine regimen. In one participant an elevated ALT was noted one month after the second HIV-MVA that was attributed to acute alcohol induced hepatitis.

We observed a number of male study participants with transiently low neutrophil counts, which commonly occurred 12–24 weeks from the last immunization. These participants had significantly low neutrophil counts without clinical signs of infection or an increased susceptibility to infection. This perhaps is an indication of low transient neutrophil count which could be a normal variation in an African population, as has been described previously and regarded as benign ethnic neutropenia [28,29,30].

The simplified regimen with 2 intradermal injections of a total of 600 μg of HIV-DNA by Bioject had a similar priming effect as the standard regimen of 5 intradermal injections of a total of 1000 μg HIV-DNA, also given by the Bioject device. Further combining the plasmids into one injection had a similar effect on the frequency of immune responses when compared to separating the plasmids in two injections. With these findings the rather cumbersome regimen of 5 intradermal HIV-DNA injections at a somewhat higher dose of 1000 μg of HIV-DNA given as separated plasmids could be simplified to a regimen of intradermal HIV-DNA priming injections at two sites with combined or separated plasmids. Administration of fewer injections has clinical and public health relevance since the acceptability of a lower total DNA dose will reduce the cost of vaccine production. Presently these considerations are of importance especially when planning for larger future phase IIB or III clinical trials.

This study confirms the high response rates we observed in a previous study with this heterologous regimen [20]. This could possibly be explained by the recent finding of potentiation of immune responses by a boost with a heterologous vector and heterologous HIV-genes [31]

There is paucity of data in the literature on the effect of combining compared to separating HIV-DNA antigens in humans. Studies in mice have indicated that separating the Gag from Env plasmids resulted in an increased immune response to Env, while combining the peptides made no difference in responses towards Gag [24]. We did not observe such effects in our human study.

We observed that the HIV-specific CD4^+^ T cell response rates measured by ICS were comparable after the first and second HIV-MVA vaccinations. In contrast the HIV-specific CD8^+^ T cell response rates were significantly higher after the second HIV-MVA compared to the response rates after the first HIV-MVA. Similarly, in a trial where GeoVax pGA2/JS7 HIV-DNA was given as a prime and MVA/HIV62 as a boost, CD8^+^ T cells response rates increased after the second dose of HIV-MVA in vaccinees who had received one or two HIV-DNA vaccinations followed by the two HIV-MVA doses [32].

This study has some limitations. The highest concentration of HIV-DNA available was 3 mg/ml, thus a strict comparison of 2 and 5 injections could not be made since only 0.1 mL could be delivered intradermally with the Biojector. However, this difference in total dose, 600 μg vs 1000 μg, did not significantly affect the simpler regimen. A new study is underway to explore the effect of delivering 0.2mL intradermally with the Zetajet to address this problem. The comparison of the HIV-specific ICS responses in the three study groups included a smaller number of vaccinees than the corresponding comparison of IFN-γ ELISpot and antibody responses since 4-colour ICS was only performed at one of the two study sites. The choice of the primary endpoint as IFN-γ ELISpot responses after the second HIV-MVA was not optimal since we observed that the magnitude of ELISpot responses to Gag was higher after the first HIV-MVA vaccination compared to the responses after the second HIV-MVA boost similar to what was observed in our previous study [20]. Similar findings have also been reported in a phase I trial that included priming with two doses of HIV-DNA subtype C and boosting with two doses of recombinant HIV-MVA vaccines [33]. In the present trial, we employed a short 4 month interval between the two HIV-MVA vaccinations. A lack of enhanced ELISpot responses to Gag peptides was also observed in our previous study when a 12 month interval was used between the two HIV-MVA vaccinations [20].

Our three immunization schedules also induced similar antibody titers to HIV Env. As was observed in the HIVIS03 study [20], a majority of the vaccinees had binding antibodies to gp160 following the second HIV-MVA boost. A study of antibody responses in a subset of the vaccinees who received recombinant gp140 in GLA adjuvant after the second HIV-MVA boost, will be presented in a separate paper

In conclusion, intradermal HIV-DNA priming with a simplified regimen of 2 injections of 600 μg HIV-DNA, with combined plasmid pools followed by boosting with HIV-MVA is safe, and elicits cellular and antibody immune responses that are similar to the standard regimen of 5 injections of 1000 μg HIV-DNA, with separated plasmid pools. It is thus possible to explore if the immune response to HIV-DNA can be further enhanced with intradermal electroporation of HIV-DNA given in 2 injections of combined plasmid pools at a total dose of 600 μg in the ongoing phase II, TaMoVac-II study.

## Supporting Information

S1 File Trial Protocol A; CONSORT Checklist A; Ethical Approval Request A; Ethical Approval A; Ethical Approval B; Ethical Approval C; Ethical Approval D; Regulatory Approval; Trial Registration A. (ZIP)
