# Supplementary material for: Priming with a Simplified Intradermal HIV-1 DNA Vaccine Regimen followed by Boosting with Recombinant HIV-1 MVA Vaccine Is Safe and Immunogenic: A Phase IIa Randomized Clinical Trial
Source: PLoS One. 2015 Apr 15;10(4):e0119629. doi: 10.1371/journal.pone.0119629 (PMC4398367; doi:10.1371/journal.pone.0119629)
Supplement: S1 File — (ZIP) [file pone.0119629.s001.zip › Supplemental Information/Approval Request and amendment A.pdf]

MUHIMBILI UNIVERSITY OF HEALTH AND ALLIED SCIENCES (MUHAS)

School of Medicine

DEPARTMENT OF INTERNAL MEDICINE

Telephone 255-754 387328; 255-713702211

Fax 255-22-2151350
255-22-2150465

E-mail: drbakari@yahoo.com
mbakari@muhas.ac.tz

Postal Address
P.O. Box 65001
DAR ES SALAAM
TANZANIA

6th Sept 2010

Chairman
National Health Research Ethics Committee
National Institute for Medical Research (NIMR)
P.O. Box 9653
Dar es Salaam

Chairman
Senate Research and Publications Committee
MUHAS
P.O. Box 65001
Dar es Salaam

Chairman
Mbeya Medical Research and Ethics Committee
Mbeya Referral Hospital
P.O. Box 419
Mbeya

Dear,

RE: Request for an Ethical Clearance of an amended Research Protocol titled "*A Phase I/II trial to assess safety and immunogenicity of i.d. DNA priming and i.m. MVA boosting in healthy volunteers in Tanzania and to develop further HIV vaccine trial capacity building in Tanzania*"

On behalf of my other collaborators, I do hereby request for ethical clearance of the above-mentioned amended version (3.0) of the research protocol dated 19th August 2010.

MUHIMBILI UNIVERSITY OF HEALTH AND ALLIED SCIENCES (MUHAS)

School of Medicine

DEPARTMENT OF INTERNAL MEDICINE

ELIMU TIBA UTAFITI

Telephone 255-754 387328; 255-713702211

Fax 255-22-2151350
255-22-2150465

E-mail: drbakari@yahoo.com
mbakari@muhas.ac.tz

Postal Address
P.O. Box 65001
DAR ES SALAAM
TANZANIA

6th Sept 2010

Director General
Tanzania Food and Drugs Authority
P.O. Box 77150
Dar es Salaam, TANZANIA

Dear,

RE: Request for approval of an amended Research Protocol titled "*A Phase I/II trial to assess safety and immunogenicity of i.d. DNA priming and i.m. MVA boosting in healthy volunteers in Tanzania and to develop further HIV vaccine trial capacity building in Tanzania*"

On behalf of my other collaborators, I do hereby request for approval of the above-mentioned amended version (3.0) of the research protocol dated 19th August 2010.

One of the essential amendments to the protocol relates to shortened durations between vaccinations as follows:

1. A shortened duration between the 3rd DNA/placebo vaccination and the first MVA/placebo from **24** to **18** weeks.

2. A shortened duration between the 1st MVA /placebo vaccination and the 2nd MVA/placebo from **24** to **16** weeks.

These changes have been necessitated by the desire to have volunteers complete the vaccination schedule as quickly as possible and to keep the time without immunity to a minimum. Since laboratory competency had also been achieved, this was decided to be very rational.
Furthermore, the proposed amendment in durations will not affect the scientific integrity of the study and will indeed conform more closely to other current DNA prime - pox boost trial schedules (*McCormack S, Stöhr W, Barber T, Bart PA, Harari A, Moog C, Ciuffreda D, Cellerai C, Cowen M, Gamboni R, Burnet S, Legg K, Brodnicki E, Wolf H, Wagner R, Heeney J,Frachette MJ, Tartaglia J, Babiker A, Pantaleo G, Weber J. EV02: A Phase I trial to compare the safety and immunogenicity of HIV DNA-C prime-NYVAC-C boost to NYVAC-C alone. Vaccine. 2008 Jun 13;26(25):3162-74*)

One of the essential amendments to the protocol relates to shortened durations between vaccinations as follows:

1. A shortened duration between the 3rd DNA/placebo vaccination and the first MVA/placebo from **24** to **18** weeks.

2. A shortened duration between the 1st MVA /placebo vaccination and the 2nd MVA/placebo from **24** to **16** weeks.

These changes have been necessitated by the desire to have volunteers complete the vaccination schedule as quickly as possible and to keep the time without immunity to a minimum. Since laboratory competency had also been achieved, this was decided to be very rational. Furthermore, the proposed amendment in durations will not affect the scientific integrity of the study and will indeed conform more closely to other current DNA prime - pox boost trial schedules (*McCormack S, Stöhr W, Barber T, Bart PA, Harari A, Moog C, Ciuffreda D, Cellerai C, Cowen M, Gamboni R, Burnet S, Legg K, Brodnicki E, Wolf H, Wagner R, Heeney J,Frachette MJ, Tartaglia J, Babiker A, Pantaleo G, Weber J. EV02: A Phase I trial to compare the safety and immunogenicity of HIV DNA-C prime-NYVAC-C boost to NYVAC-C alone. Vaccine. 2008 Jun 13;26(25):3162-74*)

Otherwise details of all the amendments that have been made on the protocol are as shown in the attached document.

We are therefore submitting copies of the revised protocol (Version 3, 19th Sept 2010) whereby the changes are highlighted.

Please note that a request for approval of the amended protocol has also been sent to the Tanzania Food and Drugs Authority (TFDA) while being fully aware that such an approval is conditional upon receipt of ethical clearance from your committees.

We do thank you very much for your continuing support and collaboration, and a speedy response is eagerly awaited from an Expedited review process.

Sincerely,

Dr Muhammad Bakari (MD, MMed, PhD)
Project Co-ordinator and Principal Investigator

Cc: Director General
Tanzania Food and Drugs Authority
P.O. Box 77150
Dar es Salaam, TANZANIA

Soren Andersson (Representing SMI as a Sponsor)
Swedish Institute for Infectious Disease Control
Nobels väg 18
171 82
Solna, SWEDEN

Otherwise details of all the amendments that have been made on the protocol are as shown in the attached document.

We are therefore submitting copies of the revised protocol (Version 3, 19th Sept 2010) whereby the changes are highlighted.

Please note that a request for ethical approval of the amended protocol has been sent to National Ethics Committee at NIMR, as well as the Institutional Review Boards of MUHAS and Mbeya.

We do thank you very much for your continuing support and collaboration, and a speedy response is eagerly awaited from an Expedited review and approval process.

.

Sincerely,

Dr Muhammad Bakari (MD, MMed, PhD)
Project Co-ordinator and Principal Investigator

cc: Soren Andersson (Representing SMI as a Sponsor)
Swedish Institute for Infectious Disease Control
Nobels väg 18
171 82, Solna, SWEDEN

Bakari Lembariti (Representing MUHAS as a Sponsor)
Deputy Vice Chancellor (Planning, Financeand Administration)
Muhimbili University of Health and Allied Sciences (MUHAS)
P.O. Box 65001
Dar es Salaam, TANZANIA

Dr Leonard Maboko (MMRP site PI)
Mbeya Referral Hospital,
P.O. Box 419,
Mbeya, Tanzania

The Chairman
National Health Research Ethics Committee
National Institute for Medical Research (NIMR)
P.O. Box 9653
Dar es Salaam

Chairman
Senate Research and Publications Committee
MUHAS
P.O. Box 65001
Dar es Salaam

Chairman
Mbeya Medical Research and Ethics Committee
Mbeya Referral Hospital
P.O. Box 419
Mbeya

Bakari Lembariti (Representing MUHAS as a Sponsor)
Deputy Vice Chancellor (Planning, Financeand Administration)
Muhimbili University of Health and Allied Sciences (MUHAS)
P.O. Box 65001
Dar es Salaam, TANZANIA

Dr Leonard Maboko (MMRP site PI)
Mbeya Referral Hospital,
P.O. Box 419,
Mbeya, Tanzania

Changes in the Amendment 1 of the TaMoVac I Protocol, Version 3.0, 19 August 2010:

"A Phase I/II trial to assess safety and immunogenicity of i.d. DNA priming and i.m. MVA in healthy volunteers in Tanzania and to develop further HIV vaccine trial capacity building in Tanzania."

1. Changes for study investigators (Section 1 of the protocol)

At MUHAS the following study investigators have changed or dropped out:

1. ***Laboratory:*** Willy Urassa was removed from the protocol
2. ***Clinical:*** Dr. Joel Francis was removed from the protocol and is replaced by Dr. Patricia Munseri who also took over his position in the Protocol Team, the Trial Coordinating Committee (TCC) and the Trial Management Group (TMG)
3. ***Trial Nurses:*** Irene Chopeta was removed from the protocol

At MMRP the following study investigators have changed or dropped out:

1. ***Laboratory:*** Britta Flach and Alexandra Schütz (immunology) were removed from the protocol and replaced by Lilly Podola
2. ***Clinical:*** Dr. Lucas Maganga and Dr. Yesaya Mwasubila were removed from the protocol. Dr. Bahati Kaluwa (also added in the TMG) and Dr. Issakwisa Mwakyula were added to the protocol.
3. ***Trial Nurses:*** Mrs. Mercy Lutomo was removed from the protocol and Mrs. Rosemary Mwilinga was added.
4. ***Pharmacist:*** Mrs. Theopista Lotto was added as a 2nd pharmacist to the protocol.
5. ***Internal Monitor:*** Mr. Revocatus Kissendi was removed from the protocol and replaced by Mrs. Doreen Pamba. (also added in the TMG).

2. Changes for the Trial Sponsor

Prof. Eligius Lyamuya was replaced as the representative of the MUHAS Sponsor as he is also a study investigator. He is replaced by:

Professor Bakari Lembariti (DDS, PhD)
Deputy Vice Chancellor (Planning, Financeand Administration)
Muhimbili University of Health and Allied Sciences (MUHAS)
P.O. Box 65001
Dar es Salaam
TANZANIA
Phone: +255 22 2151306-6 Ext 219
E-mail: dvcpfa@muhas.ac.tz;

3. Modification in the immunization schedule (Study Groups, Section 2.3 and 6.5 of the protocol)

The interval between DNA I and MVA I will be shortened from 24 weeks to 18 weeks and the interval between MVA I and MVA II will be shortened from 24 weeks to 16 weeks. The main reason for making these changes agreed within the TaMoVac I Scientific Committee was that it is desirable for individuals to complete any vaccination schedule as quickly as possible and to keep the time without immunity to a minimum. Furthermore shortening of the study duration was

advised to comply with the EDCTP funding time table. The following immunization schedule will be applicable:

Group	Number of Volunteers	**DNA immunization** weeks 0, 4 and 12	**MVA boost** weeks 30 (before 36) and 46 (before 60)
IA	36	600 µg i.d (combined plasmid pools)	MVA, at 10^8 pfu i.m
IB	4	Saline 2 x 0.1ml i.d	Saline, im
IIA	36	600 µg i.d (separate plasmids pools)	MVA, at 10^8 pfu i.m
IIB	4	Saline 2 x 0.1 ml i.d	Saline, im
IIIA	36	1000 µg i.d (separate plasmids pools)	MVA, at 10^8 pfu i.m
IIIB	4	Saline 5 x 0.1 ml i.d	Saline, im

Changes according to the altered vaccination schedule were included into other protocol sections as applicable and highlighted in grey.

4. Modification in the follow-up visits schedule

Visit 11 pre-vaccination visit
Visit 11 was deleted. This visit was created as a pre-vaccination visit to reassure on the medical status and availability of the study participant after a long pause of scheduled visits weeks before the first MVA vaccination. Due to the shortened interval between the DNA I and MVA I a follow-up pre vaccination visit in no longer necessary. As this visit will be deleted:

1. The planned blood volume of 9 ml for safety blood will not be necessary reducing the total blood volume by 9 ml.
2. The total number of study visits will be reduced from 19 to 18 visits and visits are renumbered

Long term follow-up visits (Visit 18 and Visit 19)
The interval between the long-term follow up visits (Visit 18 and 19) and the last vaccination (MVA II) have been shortened form 24 weeks to 12 weeks for Visit 18 and from 48 weeks to 24 weeks.

Duration of the study (section 6.3 of the protocol)
Due to changes in the visits schedule volunteers will be monitored until week 24 *(before 48 weeks)* after the last injection i.e. a total of 70 weeks *(before 108 weeks)*

All sections in the protocol have been modified according to the new schedule as applicable especially applicable in ***Section 8 Study Procedures*** as followed:

The **Study visit 11 – Follow up** (pre-vaccination visit) was deleted. **Study visit 12 – Vaccination IV (1st MVA/Placebo)** as indicated in the initial protocol is now numbered as Study Visit 11 and all following visits have been re-numbered (Visits 11-18) and corrected according to study weeks and study windows (highlighted in grey).

Study visit 11 – Vaccination IV (1st MVA/Placebo) – ***(Section 8.11 of the protocol)***
(Study week 30; 126 days post V8 +14/-28 days)

The following paragraph in the visit description was deleted: "*In addition an ECG will be performed on study volunteers. Interpretation of ECG findings with comparison of baseline ECG results during screening will be performed by a competent study physician and subject to potential exclusion from further vaccinations in the case of clinically significant findings.*" No additional ECG will be performed as baseline to compare with ECG finding after MVA vaccinations. It was decided that the ECG's performed during screening visits are sufficient as a baseline record.

Study visit 12 – Follow up – *(Section 8.12 of the protocol)*
(Study week 32; 14 days after V11 +/- 3 days)
No changes for visit procedures

Study visit 13 – Follow up – *(Section 8.13 of the protocol)*
(Study week 34; 28 days after V11 +/- 5 days)
No changes for visit procedures

Study visit 14 – Vaccination V (2nd MVA/Placebo) – *(Section 8.14 of the protocol)*
(Study week 46; 168 days post V11 +14/-28 days)
No changes for visit procedures

Study visit 15 – Follow up – *(Section 8.15 of the protocol)*
(Study week 48; 14 days after V14 +/- 3 days)
No changes for visit procedures

Study visit 16 – Follow up – *(Section 8.16 of the protocol)*
(Study week 50; 28 days after V14 +/- 5 days)
No changes for visit procedures

Study visit 17 – Follow up – *(Section 8.17 of the protocol)*
(Study week 58; 84 days post V14 +/- 14 days)
No changes for visit procedures

Study visit 18 – Final study visit – *(Section 8.18 of the protocol)*
(Study week 70; 168 days post V14 +/- 14 days)
No changes for visit procedures

Modification according to the new visits schedules are done throughout other part of the protocol where applicable and highlighted in grey. These are especially applicable in:

- **Appendix 1:** Study Timetable
- **Appendix 2:** TaMoVac I Visit schedule and CRF Flow Chart
- **Appendix 3:** TaMoVac I Immunization and Lab Flow Chart

5. Modification concerning the immunogenicity testing schedule (Appendix 3 of the protocol)

The immunogenicity test with large blood volume (109 ml) for the MVA I vaccination day (Visit 11 of new visit schedule) has been replaced by a small blood volume (9 ml) safety blood. The small blood volume (9 ml) safety blood at the 28 days post MVA safety visit (Visit 13 of new visit schedule) will be replaced by a large blood volume (109 ml) immunogenicity test blood. The total blood volume for the study will not be modified due to these changes. It was agreed within the TaMoVac I lab group and Steering Committee that shifting immunogenicity testing from a

baseline value on the MVA I vaccination day to the second post MVA vaccination visit would scientifically contribute more information.

6. Modifications concerning study volunteers (section 6.4 of the protocol)

Primarily, volunteers will be recruited in Dar es Salaam from the Police force with whom there has been extensive prior contact for the last 10 years. ***Additionally volunteers will also be recruited from the Prison force.*** Volunteers from the general public ***including youths attending the Infectious Disease Clinic (IDC)*** who would have been assessed by the clinical team regarding their availability for long-term follow up will also be invited.
Differential recruitment will be accommodated in case a site is not able to recruit the 60 volunteers or is unable to recruit the 15 females in the specified time of enrolment

7. Modifications concerning Inclusion Criteria (section 6.7 of the protocol)

Effective contraception will be required until 6 months (24 weeks) which is the end of the study follow-up period. All detected pregnancies until 6 months after the last vaccination will be reported and followed-up until delivery. It has been found that different parts regarding contraception and pregnancy reporting are not uniform in the protocol and assessment tools and needed to be corrected as followed:

Inclusion Criteria, item 5
As reported as a Memo to the Ethics Committees on 17.08.2010 the Assessment of Understanding was reduced from 20 to 19 questions allowing still 2 wrong (17 correct) answers for inclusion. As 17/19 questions equals 89% a correction in the inclusion criteria was performed:

> *"Satisfactory completion of an assessment of understanding prior to enrolment defined as 90% correct answers after three opportunities to take test."*
>
> Changed to
>
> *"Satisfactory completion of an assessment of understanding prior to enrolment defined as 89% correct answers after three opportunities to take test."*

Inclusion Criteria, item 9

> *"Verbal assurances that adequate birth control measures are used not to conceive/father a child during the study and up to **3 months** after the last vaccine injection"*
>
> Changed to:
>
> *"Verbal assurances that adequate birth control measures are used not to conceive/father a child during the study and up to **6 months** after the last vaccine injection"*

This procedure was reported on 10th March 2010 as a "Submission of a Memo on Minor Protocol Changes in the TaMoVac-01 Clinical Trial" to the ethical committees.

Lab inclusion criteria

Lab inclusion critereria had to be modified as at the beginning of the screening period as inconsistencies were noted which were basically due to typographical errors. Changes were discussed with in the TaMoVac Trial Committee and corrected. An Information Letter regarding these corrections was submitted on 31 May 2010 to the Ethics Committee's by the TaMoVac PI.

The following paragraph was modified:

"Reference ranges will be in accordance with data generated at MUHAS for the Dar es Salaam site, and that generated at Mbeya (MMRP) for the Mbeya site. Exclusion by presence of Diabetes mellitus will be based on the WHO cut-off value of a Fasting Blood Glucose <7.8 mmol/l. Local reference ranges will be use if the DAIDS Table for Lab Criteria refers to upper limit of normal (ULN), e.g. liver enzymes, creatinine, bilirubin) or lower limit of normal, e.g. albumin (LLN). For hematology and urine analysis results of the DAIDS toxicity table criteria will be applicable.
No grade 1 or higher routine laboratory parameters (see DAIDS Table for Clinical and Lab Criteria for Definitions, Appendix 7):"

Modified paragraph:

Reference ranges will be in accordance with data generated at MUHAS for the Dar es Salaam site, and that generated at Mbeya (MMRP) for the Mbeya site. Exclusion by presence of Diabetes mellitus will be based on the ***DAIDS Table for Lab Criteria cut-off value of a Fasting Blood Glucose ≥6.11 mmol/L if random Blood Glucose is ≥6.44 mmol/L (Grade 1 Toxicity).*** *Local reference ranges will be use if the DAIDS Table for Lab Criteria refers to upper limit of normal (ULN), e.g. liver enzymes, creatinine, bilirubin) or lower limit of normal, e.g. albumin (LLN). For hematology and urine analysis results of the DAIDS toxicity table criteria will be applicable.*

I addition the following changes in the lab inclusion was performed:

Initial protocol	Changes made
Hb >10.5g/dl	*Hb >10.5g/dl*
White blood cell count >1,300/mm^3	***White blood cell count <13,000/mm^3***
Neutrophils (not mentioned)	***Neutrophils >1,200/mm^3***
Granulocytes <6.4/ mm^3	***Parameter was deleted***
Lymphocytes >1.0/ mm^3	*Lymphocytes >1.0/ mm^3*
Platelets >120,000/ mm^3	*Platelets >120,000/ mm^3*
Random Blood Glucose 2.5-7.0 mmol/L; if elevated, then a Fasting Blood Glucose <7.8 mmol/l	*Random Blood Glucose <6.44 mmol/L; if elevated, then a Fasting Blood Glucose <6.11 mmol/l*
Bilirubin <1.25 x uln	*Bilirubin <1.25 x uln*
ALT <1.25 x uln	*ALT <1.25 x uln*
Creatinine <1.25 x uln	*Creatinine <1.25 x uln*

8. Modifications concerning labeling procedures (referring to section 8.6.1 of the protocol)

Site and participant code number will contain 4 digitals indicating the first digital **3** *(before 1)*for Dar es Salaam and 2 for Mbeya followed by 3 digitals for participant identification (e.g. Dar: No. **3001**, Mbeya: No. 2001).

9. Modification concerning Informed Consent Procedures

Informed consent procedures and study information (referring to section 8.1.1 of the protocol

The following sentence was added into the section:
"Study Participants who have received and signed the initial informed consent (Version 2.0, September 2009) will receive written information regarding the changed visit schedule. Signed and dated informed consent will be requested ***(see Appendix 4 C and D).****"*

Enrolment and Vaccination I (1st DNA/Placebo) (Study Visit 3, week 0) (referring to section 8.3 of the protocol)

"A second short written informed consent will be performed to reassure that the volunteer fully understands the concept of the study, to confirm the voluntary nature of the study participation and to reassure on the study commitment by the participant."

This was changed to:

"A senior Physician will confirm if the volunteer understands the concept of the study assess the willingness and voluntariness the study volunteer. "

No second written informed consent will be signed but volunteers are undergoing pre-inclusion and eligibility check concerning understanding, willingness and voluntariness which signed of in an enrolment form by a senior physician. This procedure was reported on 10th March 2010 as a "Submission of a Memo on Minor Protocol Changes in the TaMoVac-01 Clinical Trial" to the ethical committees.

10. Modification concerning screening procedures (referring to section 8.1. and 8.2 of the protocol)

It has been found to be more feasible if ECG's are not performed during Screening Visit 1 but at Screening Visit 2 after having reviewed all other eligibility criteria. Therefore, the following sentence was removed from section 8.1.3 (Clinical History and Physical Examination, Screening I): *"An ECG will be performed for baseline assessment* ***(CRF9-III)****. A competent study physician will do interpretation of ECG results."*
The following sentence was included in section 8.2, Screening II: ***"An ECG will be performed for baseline assessment (CRF9-III). A competent study physician will do interpretation of ECG results and all ECG results will be sent as a PDF for interpretation/approval to a cardiologist panel."***

11. Modification concerning pregnancy reporting and follow up (referring to section 8.21 of the protocol)

In the last two sentences of this paragraph it was described: *"According to the study protocol female participants will be required to use an effective contraceptive method until 3 months after the last vaccination, which is Visit 18. At this point the last pregnancy test will be performed according to the study flow schedule. All pregnancies after Visit 18 will not have to be followed up anymore according to the study requirements."*

An effective contraception should be used until ***6 months*** after the last vaccination which was not consistent in different section of the initial protocol. According to the new visit schedule these paragraphs are changed as followed: ***"According to the study protocol female***

participants will be required to use an effective contraceptive method until 6 months after the last vaccination, which is Visit 18. Pregnancy test will be performed according to the study flow schedule."

This procedure was reported on 10th March 2010 as a "Submission of a Memo on Minor Protocol Changes in the TaMoVac-01 Clinical Trial" to the ethical committees.

12. Modification concerning the Vaccine (Trial Products) - (section 10 of the protocol)

The following sentence was replaced in **Sections 2.3, 10.1 and 10.2:**
"Locally available normal saline will be used as placebo." by: ***"Sterile commercially available normal saline for human use will be used as the placebo."***

MVA Boosting (referring to section 10.2 of the protocol)

Supply, storage and composition of MVA
The following paragraph was modified:
"The presentation is in liquid form at 10^8 pfu per mL with an extractable volume of 1ml vials, which should be stored at -80 ^{0}C until use. Care should be taken not to break the cold chain."
According to the provider the MVA vaccine can be stored at -20°C for up to 3 months and the sentence was changed to: ***"The presentation is in liquid form at 10^8 pfu per mL with an extractable volume of 1ml vials, which should be stored at -20 ^{0}C up to 3 months (-80°C if longer then 3 months) until use."***

13. Modification concerning Endpoints - (section 11 of the protocol)

Safety endpoints (referring to 11.1 of the protocol)
The following paragraph was modified due to the shortened visit schedule and follow-up period: *"Adverse events will be assessed using a standard format for soliciting local and systemic reactogenicity to the vaccine and collection of unsolicited adverse events. Solicited reactogenicity will be evaluated for 7 days following each vaccination. All other AE will be collected from the time of first injection until 3 months after the last injection."*

Modified paragraph:
*"Adverse events will be assessed using a standard format for soliciting local and systemic reactogenicity to the vaccine and collection of unsolicited adverse events. Solicited reactogenicity will be evaluated for 7 days following each vaccination. All other AE will be collected from the time of first injection **until the end of the study follow-up period.**"*

14. Modification concerning Immunogenicity and Safety Assessments - (section 12 of the protocol)

Routine laboratory parameters for safety assessments (referring to 12.1.4 of the protocol)
The following paragraph was modified: *"The following safety assessments will be undertaken at appropriate laboratories at MUHAS and MMRP according to standard operating procedures. Lab results will be graded according to the DAIDS Table for Clinical and Lab Criteria for Definitions **(see Appendix 9)**."*

Modified and edited paragraph:
"The following safety assessments will be undertaken at appropriate laboratories at MUHAS and MMRP according to standard operating procedures. All lab values will be interpreted according

to established MMRP and MUHAS local reference ranges. Lab results requiring AE reporting after enrolment will be graded according to the DAIDS Table for Clinical and Lab Criteria for Definitions ***(see Appendix 9)****. In order to harmonize with local reference ranges, the cut-off for Grade I lab toxicity reporting have been modified for neutrophils from ≤1.300 cells/µl (DAIDS) to ≤1.100 cells/µl (local values)."*

Clinical Chemistry: Troponin I (not Troponin T) will be used for post MVA safety evaluation. Tables and all sections were Troponin T was mentioned has been changed to Troponin I.

Pregnancy test: The following sentence was modified: *"Pregnancy test will be performed by study nurses at the clinic from urine during screening, at each vaccination day with results available before vaccine administration and at study week 72 (Visit 18)."*
According to the new schedule the sentence is changed to: ***"Pregnancy test will be performed by study nurses at the clinic or at the study lab from urine during screening, at each vaccination day with results available before vaccine administration and at the long term follow-up visits 17 (week 58) and 18 (week 70)."***

Other Tests
This section was renamed to ***ECG Tests*** and more detailed procedures were added replacing the initial version: *"ECG will be done at enrolment and 2 weeks post MVA/Placebo vaccinations. It will also be done at anytime whenever a volunteer comes with symptoms and signs suggestive of peri-myocarditis. Results will be interpreted by competent study physicians and graded as normal, abnormal/clinically insignificant and abnormal/clinically significant."*

ECG
ECG will be done during screening/before enrolment as a baseline assessment to exclude volunteers with significant ECG findings. Significant ECG findings leading to exclusion are defined as "Abnormality in ECG that could indicate risk or make interpretation of vaccine effects difficult according to the study operating procedures." According to this definition volunteers will be excluded who have:

1. Evidence in the ECG for cardiologic disease
2. Variations in the ECG which might not be clinical significant but will complicate later interpretation for post vaccination ECG results.

ECG's will be interpreted by competent study physicians and be graded as ***normal, abnormal/clinically insignificant*** and ***abnormal/clinically significant.*** Any abnormal finding will be commented in the case report form ***(CRF 9-III).*** All ECG's will be sent prior to enrolment to a cardiologist panel for review and approval via email. The final verdict on the ECG with regards to enrolling or not enrolling the volunteer into the study will be provided by the Chairperson of the panel. The ECG panel will consist of:

Dr. Bernard R Chaitman (Chairperson)
chaitman@swbell.net

Dr. Johnson Lwakatare
medi@raha.com

ECG will be repeated 2 weeks post MVA/Placebo vaccinations or at anytime whenever a volunteer comes with symptoms and signs suggestive of peri-myocarditis. Results will be interpreted and compared to baseline ECG results by competent study physicians and send to the cardiologist panel for review and approval. Post vaccination ECG will be graded as ***normal, abnormal/clinically insignificant*** and ***abnormal/clinically significant***. Post vaccination ECG findings will be graded as ***no changes, changes/not significant*** and ***changes/significant*** as compared to baseline results. Any abnormal finding and changes will be commented in the case report form ***(CRF 9-III)***.

15. Modification concerning Reporting of Adverse Events - (section 14.3 of the protocol)

A detailed table of contacts to be notified for SAE's has been established and included in the new version including representation of the Sponsor, the ethical bodies, TFDA, DSMB, the internal and external monitors, the site PI's and clinical research coordinator, WRAIR, the London Medical Research Council (MRC) and LMU.

Name	***Position***	***email***
Sören Andersson	SMI, Sponsor	soren.andersson@smi.se
Muhammad Bakari	PI	drbakari@yahoo.com
Muhsin Aboud	MUHAS Sponsor	maboud@muhas.ac.tz<br>abouduhsin@gmail.com
Eric Sandström	Internal monitor, Karolinska Institute	eric.sandstrom@sodersjukhuset.se
Gunnel Biberfeld	SMI	gunnel.biberfeld@smi.se
Britta Wahren	SMI	britta.wahren@smi.se
Eligius Lyamuya	MUHAS	eligius_lyamuya@yahoo.com
Leonard Maboko	MMRP, site PI	lmaboko@mmrp.org
Arne Kroidl	MMRP, site CRC	akroidl@mmrp.org
Patricia Munseri	MUHAS, site CRC	pmunseri@yahoo.com
Michael Hoelscher	LMU	hoelscher@lrz.uni-muenchen.de
Merlin Robb	WRAIR	mrobb@hivresearch.org
Mary Marovich	WRAIR	mmarovich@hivresearch.org
Beryl Wessner	External monitor, WARIR	bwessner@hivresearch.org
Sheena McCormack	MRC	smc@ctu.mrc.ac.uk
Innocent Semali	Chairman of DSMB	isemali@muhas.ac.tz
Sayoki Mfinanga	NIMR	gsmfinanga@yahoo.com
NIMR chairman	NIMR ethics	headquarters@nimr.or.tz
MUHAS chairman	MUHAS IRB	drp@muhas.ac.tz
Mbeya chairman	Mbeya IRB	esamky@muchs.ac.tz
TFDA chairman	TFDA	info@tfda.or.tz

Concerning regular safety calls the following sentence was modified: "*The PI and the monitor will participate in the weekly WRAIR research safety calls.*"

Modified sentence:
"Two weekly Trial Management Group (TMG) research safety calls will be performed regularly and upon request/need. The calls will be organized by the Medical Research Council Clinical Trials Unit in London/UK chaired by Sheena McCormack."

16. Modification concerning Criteria for pausing the study - (section 16.2 of the protocol)

The following paragraph was added for third criterion in order to define feasible consensus among important safety board members for study pausing:

3. *If any AE report raises concern that the study may cause significant harm to the participants a consensus decision regarding study pausing will be searched from a safety board including study monitors, sponsor, TCC, WRAIR and DSMB (see section 14.3: table of important contacts)*

17. Modification in the Study Timetable (referring to Appendix 1 in the Protocol)

Target day		Week		Visit		Visit purpose	Visit Window	
Prev.	Mod.	Prev.	Mod.	Prev.	Mod.		Previous	Modified
		-4-8	-4-8	V1	V1	Screening I	4-8 weeks before V3 enrolment	4-8 weeks before V3 enrolment
		-2-4	-2-4	V2	V2	Screening II + baseline immunogenicity I	2-4 weeks before V3 enrolment	2-4 weeks before V3 enrolment
0	0	0	0	V3	V3	Enrolment + DNA I + baseline immunogenicity II	Week 0	Week 0
0	0			V3A	V3A	30 min post vaccination		
0	0			V3B	V3B	Evening of vaccination day		
1	1			V3C	V3C	Day 1 post vaccination		
2	2			V3D	V3D	Day 2 post vaccination		
3	3			V3E	V3E	Day 3 post vaccination		
4	4			V3F	V3F	Day 4 post vaccination		
5	5			V3G	V3G	Day 5 post vaccination		
6	6			V3H	V3H	Day 6 post vaccination		
7	7			V3I	V3I	Day 7 post vaccination		
14	14	2	2	V4	V4	Follow-up safety (+ immunogenicity)	14 days post V3 +/- 3 days	14 days post V3 +/- 3 days
28	28	4	4	V5	V5	DNA II + Follow-up safety	28 days post V3 +/- 3 days	28 days post V3 +/- 3 days
28	28			V5A	V5A	30 min post vaccination		
28	28			V5B	V5B	Evening of vaccination day		
29	29			V5C	V5C	Day 1 post vaccination		
30	30			V5D	V5D	Day 2 post vaccination		
31	31			V5E	V5E	Day 3 post vaccination		
32	32			V5F	V5F	Day 4 post vaccination		
33	33			V5G	V5G	Day 5 post vaccination		
34	34			V5H	V5H	Day 6 post vaccination		
35	35			V5I	V5I	Day 7 post vaccination		
42	42	6	6	V6	V6	Follow-up safety + immunogenicity	14 days post V5 +/- 3 days	14 days post V5 +/- 3 days
56	56	8	8	V7	V7	Follow-up safety	28 days post V5 +/- 5 days	28 days post V5 +/- 5 days
84	84	12	12	V8	V8	DNA III	84 days post V3 +/- 7 days	84 days post V3 +/- 7 days
84	84			V8A	V8A	30 min post vaccination		
84	84			V8B	V8B	Evening of vaccination day		
85	85			V8C	V8C	Day 1 post vaccination		
86	86			V8D	V8D	Day 2 post vaccination		
87	87			V8E	V8E	Day 3 post vaccination		
88	88			V8F	V8F	Day 4 post vaccination		

89	89			V8G	V8G	Day 5 post vaccination		
90	90			V8H	V8H	Day 6 post vaccination		
91	91			V8I	V8I	Day 7 post vaccination		
98	98	14	14	V9	V9	Follow-up safety + immunogenicity	14 days post V8 +/- 3 days	14 days post V8 +/- 3 days
112	112	16	16	V10	V10	Follow-up safety	28 days post V8 +/- 5 days	28 days post V8 +/- 5 days
**224**	Visit deleted	32	Visit deleted	V11	Visit deleted	Follow-up medical status pre MVA **(Visit deleted)**	28 days prior V12 +/- 7 days	**Visit deleted**
**252**	**210**	36	**30**	V12	**V11**	MVA I + immunogenicity	168 days post V8 +/- 14 days	**126 days post V8 +14 /-28 days**
**252**	**210**			V12A	**V11A**	30 min post vaccination		
**252**	**210**			V12B	**V11B**	Evening of vaccination day		
**253**	**211**			V12C	**V11C**	Day 1 post vaccination		
254	**212**			V12D	**V11D**	Day 2 post vaccination		
255	**213**			V12E	**V11E**	Day 3 post vaccination		
256	**214**			V12F	**V11F**	Day 4 post vaccination		
257	**215**			V12G	**V11G**	Day 5 post vaccination		
258	**216**			V12H	**V11H**	Day 6 post vaccination		
259	**217**			V12I	**V11I**	Day 7 post vaccination		
266	**224**	38	**32**	V13	**V12**	Follow-up safety + immunogenicity	14 days post V12 +/- 3 days	**14 days post V11 +/- 3 days**
280	**238**	40	**34**	V14	**V13**	Follow-up safety	28 days post V12 +/- 5 days	**28 days post V11 +/- 5 days**
420	**322**	60	**46**	V15	**V14**	MVA II + immunogenicity	168 days post V12 +/- 14 days	**112 days post V11 +14 /-28 days**
420	**322**			V15A	**V14A**	30 min post vaccination		
420	**322**			V15B	**V14B**	Evening of vaccination day		
421	**323**			V15C	**V14C**	Day 1 post vaccination		
422	**324**			V15D	**V14D**	Day 2 post vaccination		
423	**325**			V15E	**V14E**	Day 3 post vaccination		
424	**326**			V15F	**V14F**	Day 4 post vaccination		
425	**327**			V15G	**V14G**	Day 5 post vaccination		
426	**328**			V15H	**V14H**	Day 6 post vaccination		
427	**329**			V15I	**V14I**	Day 7 post vaccination		
434	**336**	62	**48**	V16	**V15**	Follow-up safety + immunogenicity	14 days post V15 +/- 3 days	**14 days post V14 +/- 3 days**
448	**350**	64	**50**	V17	**V16**	Follow-up safety + immunogenicity	28 days post V15 +/- 5 days	**28 days post V14 +/- 5 days**
588	**406**	84	**58**	V18	**V17**	Follow-up immunogenicity	168 days post V15 +/- 28 days	**84 days post V14 +/- 14 days**
756	**490**	108	**70**	V19	**V18**	Follow-up immunogenicity	336 days post V15 +/- 28 days	**168 days post V14 +/- 14 days**

Appendix 2: TaMoVac I Visit Schedule and CRF Flow Chart

**Visit Week**	-4-8	-2-4	0	2	4	6	8	12	14	16	30	32	34	46	48	50	58	70
**Target day**			0	14	28	42	56	84	98	112	210	224	238	322	336	350	406	490
**Visits**	V1	V2	V3	V4	V5	V6	V7	V8	V9	V10	V11	V12	V13	V14	V15	V16	V17	V18
**Visit description**	Screening 1	Screening 2	Enrolment + DNA 1	Follow-up	DNA 2	Follow-up	Follow-up	DNA 3	Follow-up	Follow-up	MVA 1	Follow-up	Follow-up	MVA 2	Follow-up	Follow-up	Follow-up	Follow-up
**Clinical Case Forms**																		
CRF Informed Consent Screening	x																	
CRF 1-I Assessment of Understanding	x																	
CRF 1-II Risk Assessment	x																	
CRF 5-III Eligibility Form		(x)	x															
CRF 5-II Inclusion/Exclusion Form		(x)	x															
CRF 2-I Visit Contact Form Screening	x	x																
CRF 3-I Physical Exam Form Screening	x																	
CRF 5-IV Screening Outcome Form		(x)	x															
CRF 5-I Basic Demographic Form	x																	
CRF 2-II Visit Contact Form Enrolment			x															
CRF 2-III Visit Contact Form				x	x	x	x	x	x	x	x	x	x	x	x	x	x	x
CRF 3-II Physical Examination Form		x	x	x	x	x	x	x	x	x	x	x	x	x	x	x	x	x
CRF 4-II Previous Condition Form	(x)	(x)	x															
CRF 4-I Concomitant Medication Form	(x)	(x)	(x)	(x)	(x)	(x)	(x)	(x)	(x)	(x)	(x)	(x)	(x)	(x)	(x)	(x)	(x)	x
CRF 6-I Adverse Events Form				(x)	(x)	(x)	(x)	(x)	(x)	(x)	(x)	(x)	(x)	(x)	(x)	(x)	(x)	x
CRF 6-II SAE Reporting Events Form				(x)	(x)	(x)	(x)	(x)	(x)	(x)	(x)	(x)	(x)	(x)	(x)	(x)	(x)	(x)
Diary Card			x		x			x			x			x				
CRF 10-I Vaccine/Reaction Form			x		x			x			x			x				
CRF 10-II Vaccine Request Form			x		x			x			x			x				
CRF 8-I Randomization Pharmacy			x															
CRF 7-I Subject Status Form			x	(x)	(x)	(x)	(x)	(x)	(x)	(x)	(x)	(x)	(x)	(x)	(x)	(x)	(x)	x
CRF 7-II Protocol Deviation Form			(x)	(x)	(x)	(x)	(x)	(x)	(x)	(x)	(x)	(x)	(x)	(x)	(x)	(x)	(x)	x
CRF 11-I Pregnancy Report Form			(x)	(x)	(x)	(x)	(x)	(x)	(x)	(x)	(x)	(x)	(x)	(x)	(x)	(x)	(x)	x
CRF 9-IV Hematology Form	x		x	x	x	x	x	x	x	x	x	x	x	x	x	x	x	x
CRF 9-V Chemistry Form	x		x	x	x	x	x	x	x	x	x	x	x	x	x	x	x	x
CRF 9-VII Urine Analysis Form	x		x		x			x			x			x			x	x
CRF 9-VI Serology Form	x																	
CRF 9-VIII CD4 Count Form			x															
CRF 9-I HIV Status Form	x		x		x			x			x			x			x	x
CRF 9-III ECG Result Form		x										x			x			
CRF 9-II Pregnancy Result Form	x		x		x			x			x			x			x	x

20. Modifications in the Informed Consent (referring to Appendix 4 of the protocol)

Changes according to the changed vaccination and visit schedule were performed:

Page 2

The study

Through a chance process (randomization), 108 volunteers will receive 3 doses of DNA vaccine (Priming) at 0, 4, and 12 weeks; to be followed by 2 doses of MVA vaccine (Boosting). The first MVA vaccine will be given 18 weeks after the last DNA injection, and the second MVA vaccine will be given 16 weeks after the first MVA vaccine. The remaining 12 volunteers will receive a Placebo (Saline) injection, which is an inactive substance that looks like the vaccine candidate given at the same time intervals as the vaccine candidates. All volunteers will then be closely followed up for 24 weeks after the last injection.

Visits	1	2	3	4	5	6	7	8	9	10	11	12	13	14	15	16	17	18
Visit weeks	-4-8	-2-4	0	2	4	6	8	12	14	16	30	32	34	46	48	50	58	70
Visit description	Screening 1	Screening 2	Enrolment and DNA 1	Follow-up 1	DNA 2	Follow-up 2	Follow-up 3	DNA 3	Follow-up 4	Follow-up 5	MVA 1	Follow-up 7	Follow-up 8	MVA 2	Follow-up 9	Follow-up 10	Follow-up 11	Follow-up 12

Kwa kupitia utaratibu wa kubahatisha bila ya anayetoa chanjo au anayepewa chanjo kuelewa wanachopewa (Randomization/mfano wa "Kutupa shilingi"), washiriki 108 kati ya hao 120 watapatiwa chanjo 3 za awali za DNA katika wiki ya mwanzoni (0), wiki ya 4 na wiki ya 12, ikifuatiwa na chanjo mbili za MVA (kipiga jeki). Chanjo ya kwanza ya MVA itatolewa wiki 18 baada ya chanjo ya tatu ya DNA, na chanjo ya pili ya MVA itatolewa wiki 16 baada ya chanjo ya kwanza ya MVA.

Washiriki 12 waliobaki, bila kutambua, watapatiwa chanjo inayofanana na zile chanjo halisi kwa mwonekano, lakini itakuwa tu na maji ya chumvi yatumikayo katika sindano za kawaida (Placebo) kwa nyakati zile zile zinazofanana na zile za chanjo halisi. Baada ya hapo, washiriki wote watafuatiliwa kwa ukaribu sana kwa takriban wiki 24, Haya yanaonyweshwa vizuri zaidi katika jedwali hili:

Hudhurio	1	2	3	4	5	6	7	8	9	10	11	12	13	14	15	16	17	18
Wiki	-4-8	-2-4	0	2	4	6	8	12	14	16	30	32	34	46	48	50	58	70
Maelezo ya hudhurio	Uchunguzi 1	Uchunguzi 2	Chanjo ya 1 ya DNA	Ufuatiliaji 1	Chanjo ya 2 ya DNA	Ufuatiliaji2	Ufuatiliaji 3	Chanjo ya 3 ya DNA	Ufuatiliaji 4	Ufuatiliaji 5	Chanjo ya 1 ya MVA	Ufuatiliaji 7	Ufuatiliaji 8	Chanjo ya 2 ya MVA	Ufuatiliaji 9	Ufuatiliaji 10	Ufuatiliaji 11	Ufuatiliaji 12

Page 5

In total, during the entire study period of about 17 months, you will be required to visit the clinic about 18 times and you will be requested to donate a total of 1200 milliliters of blood (about two normal blood donations) at different intervals to assess your health as well as the immunological response to immunization. Normally it is quite safe to make a blood donation of 500 milliliters after every 6 months.

Kwa ujumla katika kipindi chote cha utafiti kitakachokuwa takribani miezi 17 utatakiwa kufanya mahudhurio yapatayo 18 katika kliniki ya utafiti huu na utatakiwa kutoa damu takriban mililita 1,200 (kama mara mbili ya kiwango cha kawaida ambacho watu hujitolea damu mahospitalini kwa mara moja) kwa ajili ya vipimo katika nyakati tofauti ili kufuatilia afya yako, na vile vile kufuatilia uwezo wa mwili wako kutengeneza vichocheo vya kinga kufuatia chanjo hii ya majaribio. Kwa kawaida ni salama kabisa kwa mtu kujitolea damu kiwango cha mililita 500 kila baada ya miezi sita.

Page 7

Risks of the MVA vaccine:
The original smallpox vaccine has been seen to cause inflammation in the heart in very few people. This has so far not been seen with MVA, but an electrical tracing of your heart will be done at baseline (known as an ECG). The tracing will be reviewed by a panel of 2 heart experts, who will advise whether or not you can enroll, and whether further investigations are needed.

Majibu ya kipimo hicho cha ECG yataangaliwa na jopo la watalaamu wawili wa magonjwa ya moyo ambao watatoa ushauri kama ni sawa au si sawa kukuingiza kwenye utafiti huu, au kama vipimo zaidi vitahitajika.

To explain changes in the vaccination and visit schedule a short information sheet which needs to be read and signed for a approval by enrolled participants was created. See:

1. Appendix 4C: TaMoVac I Amendment 1 Information Sheet, August 2010 (English)
2. Appendix 4D: TaMoVac I Amendment 1 Information Sheet, August 2010 (Swahili)

21. Modification of the TaMoVac I Assessment of Understanding (referring to Appendix 5 of the protocol)

Changes in the Assessment of Understanding were performed according to the new visit schedule for Question 6 as followed:

Question 6

*"This study will require you to come to the clinic multiple times for visits and blood draws over the next **24 months.**"*

*"Utahitajika ufike kliniki mara nyingi kwa ajili ya mahudhurio na vipimo vya damu kwa kipindi cha **miezi 24**."*

Changed to:

*"This study will require you to come to the clinic multiple times for visits and blood draws over the next **17 months.**"*

*"Utahitajika ufike kliniki mara nyingi kwa ajili ya mahudhurio na vipimo vya damu kwa kipindi cha **miezi 17**."*

A discrepancy in the Assessment of Understanding regarding questions #13 and #15 was detected between the MUHAS and the MMRP study team.

		MUHAS interpretation	***MMRP interpretation***
#13	DNA and MVA based vaccines have been used for other diseases and shown to be safe in humans.<br>Chanjo za DNA naMVA zimekuwa zikitumika dhidi ya magonjwa mengine na zimeonekana kuwa ni salama kwa binadamu.	*False*	*True*
#15	This study tests if this HIV vaccine can induce an immune response against HIV infection.<br>Utafiti huu unapima kama chanjo hii ya VVU inaweza kuleta kinga dhidi ya maambukizi ya VVU.	*False*	*True*

The discrepancy was discussed to be due to the misunderstanding character of both questions as well as translation into Swahili. The discrepancy was reported as a Memo to the IRB on 17 Aug 2010 and the reported changes are implemented into the protocol.

1. Question #13 was deleted and the total amount of questions was reduced from 20 to 19 questions with still two false questions allowed for eligibility.
2. Question #15 (now #14 in the new protocol) was rephrased as followed:

		Correct answer
#14	This study tests if this HIV vaccine can protect individuals from acquiring HIV infection.<br>Utafiti huu unapima kama chanjo hii ya VVU inaweza kuzuia watu wasipate maambukizi ya VVU.	*False*

Modifications in the Diary Cards (referring to Appendix 8 of the protocol)

Translation problems in the Diary Card were detected before study initiation. This procedure was reported on 10th March 2010 as a "Submission of a Memo on Minor Protocol Changes in the TaMoVac-01 Clinical Trial" to the ethical committees.
The English to Swahili translations for reactogenicity assessment in the diary cards was changed as followed:

English Version	Current Swahili Version	Corrected Swahili Version
**General Symptoms**	**Dalili za Jumla**	**Dalili za Jumla**
Malaise/ Fatigue	Kuchoka Sana	Kuchoka Sana
Chills	Kutetemeka	Kutetemeka
Aching Joints	Maumivu ya Viungo	Maumivu ya Viungo
Muscle Aches	Maumivu ya Misuli	Maumivu ya Misuli
Headache	Kuumwa Kichwa	Kuumwa Kichwa
Nausea	Kichefuchefu	Kichefuchefu
Vomiting	Kutapika	Kutapika
**Injection Site Symptoms**	**Dalili Sehemu ya Uchomaji**	**Dalili Sehemu ya Uchomaji**
Local Pain	Maumivu ya Sehemu	Maumivu ya Sehemu
Local Itching	Kuwashwa	Kuwashwa
Warmth	Ujotojoto	Ujotojoto
Swelling (in cm)	Uvimbe kwa sm	Uvimbe kwa sm
Redness (in cm)	Ukubwa wa Uwekundu kwa sm.	Ukubwa wa Uwekundu kwa sm.
Induration (in cm)	Ukubwa wa Ugumu kwa sm.	Ukubwa wa Ugumu kwa sm.
Clear blister (in cm)	Malengelenge yasiyo na damu kwa sm.	Malengelenge yasiyo na damu kwa sm.
Blood blister (in cm)	Malenegelenge yenye damu kwa sm.	Malenegelenge yenye damu kwa sm.
Papule (in cm)	Uvimbe kwa sm.	Kivimbe/Kipele kwa sm

Also changes in the diary card were performed by editing the space for local reactogenicity reporting from one arm to the left and right arm, as both arms receive vaccinations.

Old Diary Card Version (as an example for the evening of the vaccination)

Jioni ya Siku ya chanjo
Tarehe:

Jotoridi: ___________ **Muda wa Kupima:** ___________

Kama una homa na unataka kupima jotoridi wakati wa usiku, rekodi jotoridi la ziada hapa:
Jotoridi: ___________ Muda wa Kupima: ___________
Jotoridi: ___________ Muda wa Kupima: ___________

Je, unatumia dawa? Ndiyo Hapana
Elezea:

Tafadhali tumia mizani ya dalili kama ilivyo chini ya Ukurasa

**Dalili za Jumla**	**Hakuna**	**Kidogo**	**Wastani**	**Sana**
Kuchoka Sana				
Kutetemeka				
Maumivu ya Viungo				
Maumivu ya Misuli				
Kuumwa Kichwa				
Kichefuchefu				
Kutapika				

**Dalili Sehemu ya Uchomaji**	**Hakuna**	**Kidogo**	**Wastani**	**Sana**
Maumivu ya Sehemu				
Kuwashwa				
Ujotojoto				

Uvimbe kwa sm	__ __. __X__ __. __ sm	**Tafadhali rekodi vipimo kwa sm, urefu na upana mkubwa zaidi** *(Mfano: 01.5 X 02.5 sm)*
Ukubwa wa Uwekundu kwa sm.	__ __. __X__ __. __ sm	
Ukubwa wa Ugumu kwa sm.	__ __. __X__ __. __ sm	
Malengelenge yasiyo na damu kwa sm.	__ __. __X__ __. __ sm	
Malenegelenge yenye damu kwa sm.	__ __. __X__ __. __ sm	
Uvimbe kwa sm.	__ __. __X__ __. __ sm	

Mizani ya Ukubwa wa Dalili

Hakuna
Kidogo = dalili ndogo; hazijasababisha mtu kutokwenda kazini, shuleni au kushindwa kujifanyia usafi wa mwili.
Wastani = dalili za kuonekana; zimeathiri utendaji lakini hazijasababisha mtu kutofanya kazi au kutojishughulisha na michezo au shughuli za kila siku.
Sana = dalili kubwa za kuhitaji kupumzika kitandani na/au zimesababisha mtu kuacha kazi au shughuli nyingine za kila siku.

Mizani ya Dalili ya Sehemu ya Uchomaji

Hakuna
Kidogo = maumivu kidogo; hayajazuia matumizi yoyote ya mkono
Wastani = maumivu bayana; yamezuia matumizi fulani ya mkono.
Sana = maumivu makali; yamezuia kabisa matumizi ya mkono.

Kwa Tatizo lolote Piga simu .... Namba (..........) au (............)

New Diary Card Version (as an example for the evening of the vaccination)

Jioni ya Siku ya chanjo
Tarehe:

Jotoridi: ___________ **Muda wa Kupima:** ____________

Kama una homa na unataka kupima jotoridi wakati wa usiku, rekodi jotoridi la ziada hapa:
Jotoridi: _____________ Muda wa Kupima: ___________
Jotoridi: _____________ Muda wa Kupima: ___________

Je, unatumia dawa? Ndiyo Hapana
Elezea:

Tafadhali tumia mizani ya dalili kama ilivyo chini ya Ukurasa

**Dalili za Jumla**	**Hakuna**	**Kidogo**	**Wastani**	**Sana**
Kuchoka sana				
Kutetemeka				
Maumivu ya viungo				
Maumivu ya misuli				
Kuumwa kichwa				
Kichefuchefu				
Kutapika				

	**Mkono wa kulia**				**Mkono wa kushoto**			
**Dalili Sehemu ya Uchomaji**	**Hakuna**	**Kidogo**	**Wastani**	**Sana**	**Hakuna**	**Kidogo**	**Wastani**	**Sana**
Maumivu ya Sehemu ya uchomaji								
Kuwashwa sehemu ya uchomaji								
Ujotojoto sehemu ya uchomaji								
Uvimbe sehemu ya uchomaji kwa sm	__ __. __X__ __. __ sm				__ __. __X__ __. __ sm			
Ukubwa wa Uwekundu (kwa sm)	__ __. __X__ __. __ sm				__ __. __X__ __. __ sm			
Ukubwa wa Ugumu (kwa sm)	__ __. __X__ __. __ sm				__ __. __X__ __. __ sm			
Malengelenge yasiyo na damu (kwa sm)	__ __. __X__ __. __ sm				__ __. __X__ __. __ sm			
Malenegelenge yenye damu (kwa sm)	__ __. __X__ __. __ sm				__ __. __X__ __. __ sm			
Kipele (kwa sm)	__ __. __X__ __. __ sm				__ __. __X__ __. __ sm			

Tafadhali rekodi vipimo kwa sm, urefu na upana mkubwa zaidi *(Mfano: 01.5 X 02.5 sm)*

Mizani ya Ukubwa wa Dalili

Hakuna
Kidogo = dalili ndogo; hazijasababisha mtu kutokwenda kazini, shuleni au kushindwa kujifanyia usafi wa mwili.
Wastani = dalili za kuonekana; zimeathiri utendaji lakini hazijasababisha mtu kutofanya kazi au kutojishughulisha na michezo au shughuli za kila siku.
Sana = dalili kubwa za kuhitaji kupumzika kitandani na/au zimesababisha mtu kuacha kazi au shughuli nyingine za kila siku.

Mizani ya Dalili ya Sehemu ya Uchomaji

Hakuna
Kidogo = maumivu kidogo; hayajazuia matumizi yoyote ya mkono
Wastani = maumivu bayana; yamezuia matumizi fulani ya mkono.
Sana = maumivu makali; yamezuia kabisa matumizi ya mkono.

Kwa Tatizo lolote Piga simu .... Namba (..........) au (............)
