# Supplementary material for: Priming with a Simplified Intradermal HIV-1 DNA Vaccine Regimen followed by Boosting with Recombinant HIV-1 MVA Vaccine Is Safe and Immunogenic: A Phase IIa Randomized Clinical Trial
Source: PLoS One. 2015 Apr 15;10(4):e0119629. doi: 10.1371/journal.pone.0119629 (PMC4398367; doi:10.1371/journal.pone.0119629)
Supplement: S1 File — (ZIP) [file pone.0119629.s001.zip › Supplemental Information/Consort check list A.pdf]

CONSORT 2010 checklist of information to include when reporting a randomised trial*

Section/Topic	Item No	Checklist item	Reported on page No
**Title and abstract**			
	1a	Identification as a randomised trial in the title	In Title
	1b	Structured summary of trial design, methods, results, and conclusions (for specific guidance see CONSORT for abstracts)	In Abstract
**Introduction**			
Background and objectives	2a	Scientific background and explanation of rationale	In Introduction
	2b	Specific objectives or hypotheses	In Introduction
**Methods**			
Trial design	3a	Description of trial design (such as parallel, factorial) including allocation ratio	In Materials and Methods in sub heading Study Design and Population
	3b	Important changes to methods after trial commencement (such as eligibility criteria), with reasons	Not applicable
Participants	4a	Eligibility criteria for participants	In Materials and Methods in sub heading Study Design and Population
	4b	Settings and locations where the data were collected	In Materials and Methods in sub heading Study Design and

			Population
Interventions	5	The interventions for each group with sufficient details to allow replication, including how and when they were actually administered	In Material and Methods under sub-heading Randomizatio n and vaccination, Table 1 and Figure 1
Outcomes	6a	Completely defined pre-specified primary and secondary outcome measures, including how and when they were assessed	In Material and Methods under sub-headings Safety Assessments. Immunologica l Assessments and sub-heading Study Endpoints
	6b	Any changes to trial outcomes after the trial commenced, with reasons	Not applicable
Sample size	7a	How sample size was determined	In Materials and Methods under sub-heading Statistical Methods
	7b	When applicable, explanation of any interim analyses and stopping guidelines	Not applicable
Randomisation:			
Sequence	8a	Method used to generate the random allocation sequence	In Materials

generation	8a	Method used to generate the random allocation sequence	and Methods under sub-heading Randomizatio n and Vaccinations
	8b	Type of randomisation; details of any restriction (such as blocking and block size)	In Materials and Methods under sub-heading Randomizatio n and Vaccinations
Allocation concealment mechanism	9	Mechanism used to implement the random allocation sequence (such as sequentially numbered containers), describing any steps taken to conceal the sequence until interventions were assigned	In Materials and Methods under sub-heading Randomizatio n and Vaccinations
Implementation	10	Who generated the random allocation sequence, who enrolled participants, and who assigned participants to interventions	In Materials and Methods under sub-heading Randomizatio n and Vaccinations
Blinding	11a	If done, who was blinded after assignment to interventions (for example, participants, care providers, those assessing outcomes) and how	In Materials and Methods under sub-heading Randomizatio n and Vaccinations

	11b	If relevant, description of the similarity of interventions	In Materials and Methods under sub-heading Randomization and Vaccinations
Statistical methods	12a	Statistical methods used to compare groups for primary and secondary outcomes	In Materials and Methods under sub-heading Statistical Methods
	12b	Methods for additional analyses, such as subgroup analyses and adjusted analyses	Not applicable
**Results**			
Participant flow (a diagram is strongly recommended)	13a	For each group, the numbers of participants who were randomly assigned, received intended treatment, and were analysed for the primary outcome	Figure 1
	13b	For each group, losses and exclusions after randomisation, together with reasons	Figure 1
Recruitment	14a	Dates defining the periods of recruitment and follow-up	In Results under sub-heading Demographics, recruitment, and inclusion and sub-heading Withdrawals/Terminations from Vaccinations
	14b	Why the trial ended or was stopped	Not applicable
Baseline data	15	A table showing baseline demographic and clinical characteristics for each group	Table 2
Numbers analysed	16	For each group, number of participants (denominator) included in each analysis and whether the analysis was	Tables

		by original assigned groups	2,3,4,5, 7 and figure 1
Outcomes and estimation	17a	For each primary and secondary outcome, results for each group, and the estimated effect size and its precision (such as 95% confidence interval)	Tables 2, 3, 5,6,7
	17b	For binary outcomes, presentation of both absolute and relative effect sizes is recommended	Not applicable
Ancillary analyses	18	Results of any other analyses performed, including subgroup analyses and adjusted analyses, distinguishing pre-specified from exploratory	Not applicable
Harms	19	All important harms or unintended effects in each group (for specific guidance see CONSORT for harms)	Under Results in sub-heading Safety and Tolerability, Solicited adverse events, Non-Solicited adverse and Laboratory adverse events, Table 3 and Table 4
**Discussion**			
Limitations	20	Trial limitations, addressing sources of potential bias, imprecision, and, if relevant, multiplicity of analyses	In Discussion in paragraph 6
Generalisability	21	Generalisability (external validity, applicability) of the trial findings	In Discussion in paragraph 3, 4 and 5
Interpretation	22	Interpretation consistent with results, balancing benefits and harms, and considering other relevant evidence	Not applicable
**Other information**			
Registration	23	Registration number and name of trial registry	WHO International Clinical Trial

			Registry ATM2010050 0021223668
Protocol	24	Where the full trial protocol can be accessed, if available	Attached supporting documents
Funding	25	Sources of funding and other support (such as supply of drugs), role of funders	In Materials and Methods under sub-heading Role of the funding sources

*We strongly recommend reading this statement in conjunction with the CONSORT 2010 Explanation and Elaboration for important clarifications on all the items. If relevant, we also recommend reading CONSORT extensions for cluster randomised trials, non-inferiority and equivalence trials, non-pharmacological treatments, herbal interventions, and pragmatic trials. Additional extensions are forthcoming: for those and for up to date references relevant to this checklist, see www.consort-statement.org.
