# Supplementary material for: Priming with a Simplified Intradermal HIV-1 DNA Vaccine Regimen followed by Boosting with Recombinant HIV-1 MVA Vaccine Is Safe and Immunogenic: A Phase IIa Randomized Clinical Trial
Source: PLoS One. 2015 Apr 15;10(4):e0119629. doi: 10.1371/journal.pone.0119629 (PMC4398367; doi:10.1371/journal.pone.0119629)
Supplement: S1 File — (ZIP) [file pone.0119629.s001.zip › Supplemental Information/Ethical Approval A.pdf]

THE UNITED REPUBLIC OF
TANZANIA

National Institute for Medical Research
P.O. Box 9653
Dar es Salaam
Tel: 255 22 2121400/390
Fax: 255 22 2121380/2121360
E-mail: headquarters@nimr.or.tz

Ministry of Health and Social Welfare
P.O. Box 9083
Dar es Salaam
Tel: 255 22 2120262-7
Fax: 255 22 2110986

NIMR/HQ/R.8c/Vol. I /139

25th November 2010

Dr Muhammad Bakari
MUHAS
School of Medicine, Department of Internal Medicine
P O Box 65001
DAR ES SALAAM

CLEARANCE CERTIFICATE FOR CONDUCTING MEDICAL RESEARCH IN TANZANIA

This letter is to confirm that your application for an amendment 01 on the research entitled: A phase I/II Trial to assess safety and immunogenicity of i.d. DNA priming and i.m. MVA boosting in health y volunteers in Tanzania and to develop further HIV vaccine trial capacity building in Tanzania, (Bakari M *et al)*, NIMR/HQ/R.8a/Vol. IX/912, dated 31st December 2009 has been granted ethics clearance to be conducted in Tanzania.

The Principal Investigator Dr Muhammad Bakari, must ensure that the approval is for:
Amendment 01:

1. A shortened duration between the 3rd DNA/Placebo vaccination and the first MVA/Placebo from 24 to 18 weeks.
2. A shortened duration between the 1st MVA/ Placebo vaccination and the 2nd MVA/Placebo from 24 to 16 weeks

Approval of the study protocol is up to 31st December 2011.

Name: Dr Mwelecele N Malecela

Signature

ACTING CHAIRPERSON
MEDICAL RESEARCH
COORDNATING COMMITTEE

Name: Dr Deo Mtasiwa

Signature

CHIEF MEDICA L OFFICER
MINISTRY OF HEALTH
AND SOCIAL WELFARE

RMO
DMO
