# Supplementary material for: Priming with a Simplified Intradermal HIV-1 DNA Vaccine Regimen followed by Boosting with Recombinant HIV-1 MVA Vaccine Is Safe and Immunogenic: A Phase IIa Randomized Clinical Trial
Source: PLoS One. 2015 Apr 15;10(4):e0119629. doi: 10.1371/journal.pone.0119629 (PMC4398367; doi:10.1371/journal.pone.0119629)
Supplement: S1 File — (ZIP) [file pone.0119629.s001.zip › Supplemental Information/Ethical Approval B.pdf]

THE UNITED REPUBLIC OF TANZANIA
MINISTRY OF HEALTH AND SOCIAL WELFARE

Cable Referral Hospital
Telephone: 2503456/2503351
Fax 2503577
Email: mrh@muchs.ac.tz

MBEYA CONSULTANT HOSPITAL
P. O. BOX 419
MBEYA

Please quote:
Ref. No. MRH/T.30/44/12

20th January 2011

Dr Leonard Maboko
NIMR-Mbeya Medical Research Program
P.O. Box 2410
MBEYA

RE: Changes in the Amendment 1 of the TaMoVac1 Protocol Version 3,0 19 August 2010

Thank you for submitting your application on which was considered at the Mbeya Medical Research and Ethics committee in its meeting of 7th December 2010.

Documents which were received include; TaMoVac 1 Protocol, version 3.0, and change in the amendment 1 of the TaMoVac 1 protocol version 3.0 of 19 August 2010.

Mbeya Medical Research and Ethics Committee approved the above mentioned document from an ethical point of view on 07th December, 2010.

If serious advice events or any other or significant change which may occurs in the connection with this study and/or which may alter in ethical consideration must be reported immediately to Mbeya Medical Research and Ethics Committee.

You are also required to

i. Submit progress report to Mbeya Medical Research and Ethics Committee every 6 months
ii. Inform the Mbeya Medical Research and Ethics Committee when the research has completed
iii. Final result to be submitted to MMREC before publication.

Thank you for what you have contributed so far in Health research advancement

Yours sincerely

Dr Humphrey Kiwelu
Secretary – Mbeya Medical Research and Ethics Committee

FOR DIRECTOR
MBEYA REFERRAL HOSPITAL
P.O. Box 419 MBEYA

cc. The chairman
National Health Research Ethics Committee
National Institute for Medical Research
P.O.Box 9653
DAR ES SALAAM.

All Communication should be addressed to Director
