# Supplementary material for: Priming with a Simplified Intradermal HIV-1 DNA Vaccine Regimen followed by Boosting with Recombinant HIV-1 MVA Vaccine Is Safe and Immunogenic: A Phase IIa Randomized Clinical Trial
Source: PLoS One. 2015 Apr 15;10(4):e0119629. doi: 10.1371/journal.pone.0119629 (PMC4398367; doi:10.1371/journal.pone.0119629)
Supplement: S1 File — (ZIP) [file pone.0119629.s001.zip › Supplemental Information/Ethical Approval C.pdf]

MUHIMBILI UNIVERSITY OF HEALTH AND ALLIED SCIENCES
DIRECTORATE OF RESEARCH AND PUBLICATIONS

P.O. BOX 65001
DAR-ES-SALAAM
TANZANIA
Telefax: 2152489
Telegrams: UNIVMED

E-MAIL *drp@muhas.ac.tz*
TEL: (255-022)-2150302-6 Ext. 207
Direct line: 2152489

Ref. No. MU/DRP/PA/ VOL. II/30

18th November 2010

Prof. Muhammad Bakari.
School of Medicine,
MUHAS.

RE: APPROVAL FOR PROTOCOL AMENDMENT FOR STUDY TITLED: "A PHASE III TRIAL TO ASSESS THE SAFETY AND IMMUNOGENICITY OF i.d. DNA PRIMING AND i.m. MVA BOOSTING IN HEALTHY VOLUNTEERS IN TANZANIA AND TO DEVELOP FURTHER HIV VACCINE TRIAL CAPACITY BUILDING IN TANZANIA"

I am pleased to inform you that the Chairman has on behalf of the Senate, approved protocol amendment for the above mentioned study based on recommendation of the Expedited Review Sub-Committee of the Senate Research and Publications Committee held on 28th October 2010.

The recommended amendment includes the following:

- A shortened duration between the 3rd DNA/Placebo vaccination and the first MVA/Placebo from 24 to 18 weeks.

- A shortened duration between the 1st MVA/Placebo vaccination and the 2nd MVA/Placebo from 24 to 16 weeks.

The committee recommended approval of the requested amendments since they are targeting to safeguard the participants and will not pose any additional risks.

M. Aboud

Prof. M. Aboud
DIRECTOR OF RESEARCH AND PUBLICATIONS

Copy to: Vice Chancellor, MUHAS. Your letter Ref. No. MU/01/1022/0112/2 of 18th November 2010.
Copy to: Deputy Vice Chancellor, Academic, Research and Consultancy, MUHAS.
Copy to: Dean, School of Nursing, MUHAS.
