# Supplementary material for: Priming with a Simplified Intradermal HIV-1 DNA Vaccine Regimen followed by Boosting with Recombinant HIV-1 MVA Vaccine Is Safe and Immunogenic: A Phase IIa Randomized Clinical Trial
Source: PLoS One. 2015 Apr 15;10(4):e0119629. doi: 10.1371/journal.pone.0119629 (PMC4398367; doi:10.1371/journal.pone.0119629)
Supplement: S1 File — (ZIP) [file pone.0119629.s001.zip › Supplemental Information/Ethical Approval D.pdf]

MUHIMBILI UNIVERSITY OF HEALTH AND ALLIED SCIENCES
DIRECTORATE OF RESEARCH AND PUBLICATIONS

P.O. BOX 65001
DAR-ES-SALAAM
TANZANIA
Telefax: 2152489
Telegrams: UNIVMED

E-MAIL drp@muhas.ac.tz
TEL: (255-022)-2150302-6 Ext. 207
Direct line: 2152489

Ref.No.MU/RP/AEC/Vol.XIII/81

16th November 2009

Dr. M. Bakari
Department of Internal Medicine
School of Medicine
MUHAS.

RE: Approval for Ethical Clearance for the Study Titled "A Phase III Trial to Assess the Safety and Immunogenocity of i.d. DNA Proming and i.m. MVA Boosting in Health Volunteers in Tanzania and to Develop Further HIV Vaccine Trial Capacity Building in Tanzania"

Reference is made to the above heading.

I am pleased to inform you that the Chairman has on behalf of the Senate, approved extension of ethical clearance of the above mentioned study, based on recommendation of the Expedited Senate Research and Publications Committee meeting held on 30th October, 2009.

The validity of this ethical clearance is one year effective from 30th October 2009- 29th October 2010. You will therefore be required to apply for renewal of ethical clearance on a yearly basis if the study is not completed at the end of this clearance. You will be expected to provide adverse events reports regularly, Data Safety Monitoring Board (DSMB) reports, six month progress reports and final project report upon completion of your study.

Dr. J. R. Masalu
Ag: CHAIRPERSON, SENATE RESEARCH & PUBLICATIONS COMMITTEE

c.c. Vice Chancellor, MUHAS-Your letter Ref. No. MU/01/1022/

c.c. Deputy Vice Chancellor, Academics, Research & Consultancy (MUHAS)

c.c. Dean, School of Medicine - MUHAS
