# Supplementary material for: Priming with a Simplified Intradermal HIV-1 DNA Vaccine Regimen followed by Boosting with Recombinant HIV-1 MVA Vaccine Is Safe and Immunogenic: A Phase IIa Randomized Clinical Trial
Source: PLoS One. 2015 Apr 15;10(4):e0119629. doi: 10.1371/journal.pone.0119629 (PMC4398367; doi:10.1371/journal.pone.0119629)
Supplement: S1 File — (ZIP) [file pone.0119629.s001.zip › Supplemental Information/Regulatory Approval A.pdf]

TANZANIA FOOD AND DRUGS AUTHORITY

E-Mail: info@tfda.or.tz
Telephone: +255 22 2450512, 2450751
+255 22 2452108
Fax No. +255 22 2450793
Website: www.tfda.or.tz
All letters should be addressed to the Director General
In reply please quote Our Ref No:

TFDA
Tanzania Food & Drugs Authority

Nelson Mandela Road,
EPI - Mabibo External,
P.O. Box 77150,
DAR ES SALAAM,
TANZANIA.

Ref. No. CE.57/180/03A/43 **16th December 2010**

Dr Mohammad Bakari
Principal Investigator TaMoVac 01
Department of internal Medicine
School of Medicine
P.O BOX 65001
DAR ES SALAAM

RE: APPROVAL FOR PROTOCOL AMMENDMENT FOR A STUDY ENTITLED "A PHASE I/II TRIAL TO ASSESS SAFETY AND IMMUNOGENICITY OF I.D DNA PRIMING AND INTRAMUSCULAR MVA BOOSTING IN HEALTHY VOLUNTEERS IN TANZANIA AND TO DEVELOP FURTHER HIV VACCINE TRIAL CAPACITY BUILDING IN TANZANIA"

Approval is hereby granted for you to continue conducting above study with amended **Protocol number Tamovac-01 Version 3** dated **19th August 2010.**

The approved study sites are **MUHAS-Dar es Salaam** and **NIMR-MMRP-Mbeya Tanzania**. The approval is still subject to the following conditions;

1. Complying with the approved protocol.

2. If for any reason the trial is prematurely terminated or suspended, a detailed written explanation must be submitted to TFDA within 21 days.

3. The Authority may withdraw the approval already given if it is dissatisfied with the conduct of study or there are breaches of any conditions prescribed in this letter or law provision.

4. Six monthly progress and final reports should be submitted to TFDA, including interim analyses done by the Data Safety Monitoring Board (DSMB) or related Committee. The progress reports should be submitted within three weeks after the end of the period being reported and the final report within 60 days of conclusion of the trial.

5. All relevant documents and records pertaining to the trial should be retained for a period of at least 3 years after the completion of the trial and made available upon request by TFDA.

6. Any amendment of the protocol, product or investigators brochure should be reported to TFDA and approval obtained before its implementation.

7. All serious adverse events should be reported within two weeks and for fatal ones within 24 hours after becoming aware of their occurrence in any of the study sites.

8. Copies of publications of any part of the study should be submitted.

9. The study participants should be insured before the study commences and copies of insurance cover submitted to this office.

Looking forward to your continued cooperation.

Mitangu A. Fimbo
For: DIRECTOR GENERAL

C.c 1. Prof. David Ngassapa (PhD)
Deputy Vice Chancellor (Academic Research and Consultancy)
Muhimbili University of Health and Allied Sciences (MUHAS)
P.O.Box 65001
DAR ES SALAAM

2. Bakari Lembariti
MUHAS Sponsor Representative
Deputy Vice Chancellor (Planning, Finance and Administration)
Muhimbili University of Health and Allied Sciences (MUHAS)
P.O.Box 65001
DAR ES SALAAM

3. Soren Andersson
Swedish Institute for Infectious Disease Control
NobelsVag18, SE-171 82
Solna, Sweden.

4. Dr. Leonard Maboko
TaMoVac 01 Mbeya Site Investigator
NIMR Mbeya medical Research Programme
P.O. Box 2410
MBEYA

MF/hi/am
