# Supplementary material for: Priming with a Simplified Intradermal HIV-1 DNA Vaccine Regimen followed by Boosting with Recombinant HIV-1 MVA Vaccine Is Safe and Immunogenic: A Phase IIa Randomized Clinical Trial
Source: PLoS One. 2015 Apr 15;10(4):e0119629. doi: 10.1371/journal.pone.0119629 (PMC4398367; doi:10.1371/journal.pone.0119629)

A Phase I/II trial to assess safety and immunogenicity of i.d. DNA priming and i.m. MVA boosting in healthy volunteers in Tanzania and to develop further HIV vaccine trial capacity building in Tanzania.

The trial is funded by the European and Developing Countries Clinical Trials Partnership Programme (EDCTP)

Muhimbili University of Health and Allied Sciences, MUHAS, will act as the Sponsor in collaboration with the Swedish Institute for Infectious Disease Control, SMI

ATM Registration: ATM2010050002122368

For the TCC

.... Date 23rd Aug 2010

Name: Muhammad Bakari

For MUHAS

.... Date 23/08/10

Name: [redacted]

For NIMR

.... Date 24/08/10

Name: Andrew Kitua/Sayoki Mfinanga

For SMI

.... Date 31/08/2010

Name: Johan Carlson

For MMRP

.... Date 21 AUGUST, 2010

Name: Leonard Maboko

For LMU

.... Date 21.08.2010 ....

Name: Michael Hoelscher

For Imperial College

.... Date 23 August 2010

Name: Frances Gotch

Table of Content

TABLE OF CONTENT 3

1 STUDY TEAM 9
1.1 Principal Investigator 9
1.2 Muhimbili University of Health and Allied Sciences (MUHAS) 9
1.3 NIMR- Mbeya Medical Research Programme (MMRP) 10
1.4 Vaccine supplier 11
1.5 Study laboratories 11
1.6 Protocol team 11
1.7 Trial Coordinating Committee, TCC 12
1.8 Trial Management Groups, TMG at each site 12
1.9 DSMB 13
1.10 TaMoVac Steering Committee 13
1.11 Trial Sponsors 13

2 SYNOPSIS 14
2.1 Primary objectives: 14
2.2 Secondary objective 14
2.3 Methodology 14
2.4 Endpoints 15

3 BACKGROUND AND LITERATURE REVIEW 16
3.1 TaMoVac Project 16
3.2 The global HIV-1 Situation 16
3.3 HIV-1 situation in Tanzania 17
3.4 Need for an HIV vaccine in Africa 18
3.5 Need for developing countries especially in Africa to participate in HIV vaccine development and trials 18
3.6 Tanzania's National HIV Vaccine Strategic Framework 19
3.7 African experience in AIDS vaccine research and development 20

3.8 Global efforts to develop an AIDS Vaccine ... 20

4 IMPLICATIONS OF RECENT HIV VACCINE TRIAL RESULTS ... 21

4.1 Table 1: Differences of immunogens and delivery by different recent prime boost candidate HIV DNA HIV-1 vaccines ... 22

4.2 DNA HIV Vaccines ... 23

4.3 Modified Vaccinia Ankara (MVA) ... 24

4.4 Prime – boost strategy ... 25

4.5 A Phase I HIV-1 vaccine trial in Sweden ... 25

4.6 Preliminary work on HIV vaccine trials in Dar es Salaam ... 25

4.6.1 HIVIS 03 ... 27
4.6.2 Cohort capacity building ... 27
4.6.3 Police Officers Cohort in Dar es Salaam ... 27
4.6.4 Ongoing or planed HIV vaccine and cohort studies in Dar es Salaam ... 28

4.7 Preliminary work on HIV vaccine trials and HIV cohorts in Mbeya ... 28

4.7.1 HIV cohorts ... 28
4.7.2 Phase I/II HIV vaccine trial in Mbeya ... 28
4.7.3 Ongoing or planed HIV vaccine and cohort studies in Mbeya ... 29

4.8 Rationale for the proposed study objectives ... 29

5 STUDY OBJECTIVES ... 30

5.1 Primary objectives ... 30

5.2 Secondary objectives ... 30

6 METHODOLOGY ... 30

6.1 Study design ... 30

6.2 Study setting ... 30

6.3 Duration of study ... 30

6.4 Study volunteers ... 30

6.5 Study groups ... 31

6.6 Randomization procedure ... 31

6.7 Inclusion criteria ... 31

6.8 Exclusion criteria ... 32

7 RECRUITMENT PROCEDURE ... 33

7.1 Recruitment in Dar es Salaam ... 33

7.2 Recruitment in Mbeya **33**

8 STUDY PROCEDURES **34**

8.1 Screening I (Study Visit 1, 4-8 weeks before enrolment) **34**

8.1.1 Informed consent procedures and study information 34
8.1.2 HIV counseling and testing 34
8.1.3 Clinical history and examination 34
8.1.4 Safety laboratory tests and HIV testing 35
8.1.5 Blood and urine collection 35
8.1.6 Labeling procedures 36

8.2 Screening II (Study Visit 2, 2-4 weeks before enrolment) **36**

8.3 Enrolment and Vaccination I (1st DNA/Placebo) (Study Visit 3, week 0) **36**

8.3.1 Randomization procedure 37
8.3.2 Blinding procedure 37
8.3.3 Vaccination 37

8.4 Study visit 4 – Follow up **38**

8.5 Study visit 5 – Vaccination II (2nd DNA/Placebo) **38**

8.6 Study visit 6 – Follow up **38**

8.7 Study visit 7 – Follow up **38**

8.8 Study visit 8 – Vaccination III (3rd DNA/Placebo) **38**

8.9 Study visit 9 – Follow up **38**

8.10 Study visit 10 – Follow up **39**

8.11 Study visit 11 – Vaccination IV (1st MVA/Placebo) **39**

8.12 Study visit 12 – Follow up **39**

8.13 Study visit 13 – Follow up **39**

8.14 Study visit 14 – Vaccination V (2nd MVA/Placebo) **39**

8.15 Study visit 15 – Follow up **39**

8.16 Study visit 16 – Follow up **39**

8.17 Study visit 17 – Follow up **40**

8.18 Study visit 18 – Final study visit **40**

8.19 Additional visits **40**

8.20 Compensation for study participation **40**

8.21 Pregnancy reporting and follow up **41**

8.22 In the event of study discontinuation 41

9 IMMUNIZATION 42
9.1 Immunization schedule 42
9.2 Doses and mode of vaccine delivery 43
9.3 Vaccination procedures and safety follow-up after immunization 43

10 THE VACCINE (TRIAL PRODUCTS) 44
10.1 DNA Priming 44
10.2 MVA Boosting 45
10.3 Dispensing records and disposal of unused product 46

11 ENDPOINTS 46
11.1 Safety 46
11.2 Immunogenicity 47

12 SAFETY AND IMMUNOGENICITY ASSESSMENTS 47
12.1 Safety assessment 47
12.1.1 Local adverse events 48
12.1.2 Systemic adverse events 48
12.1.3 Other adverse events 48
12.1.4 Routine laboratory parameters for safety assessments 49
12.1.5 Following adverse event 50
12.2 Immunogenicity assessment 50
12.2.1 Antibody responses 50
12.2.2 Cellular responses 51
12.2.3 Storage of Cell pellets for HLA testing 51
12.3 Other assessments 51
12.3.1 HIV testing 51
12.3.2 Antibodies against Vaccinia 52

13 CONCOMITANT MEDICATION 52

14 ADVERSE EVENTS 52
14.1 Definitions and Classification 52
14.2 Serious Adverse Events (SAEs) 53
14.3 Reporting of adverse events 53

15 PROTOCOL DEVIATION 56

16 DISCONTINUATION PROCEDURES 56
16.1 Criteria for withdrawal of volunteers from injection schedule 56
16.2 Criteria for pausing the study 57
17 CLINICAL MANAGEMENT 57
17.1 Management of HIV issues during and following the trial 57
17.2 HIV testing 57
17.3 Verification of HIV status of volunteers 58
17.4 Management of volunteers who acquire HIV infection during the study period 58
17.5 Social discrimination as result of a post-vaccine response 59
18 TRIAL DATA MANAGEMENT 59
18.1 Data management and entry at the clinical centre 59
18.2 Data management in the clinical/immunology laboratories 59
18.3 Data monitoring at the Central Data Management Centre 60
18.4 Archiving 60
18.5 Data ownership 60
19 STUDY MONITORING 60
19.1 Preparation of reports to assist the monitoring 60
19.2 Trial committees 61
19.2.1 Trial Coordinating Committee (TCC) 61
19.2.2 Trial Management Group (TMG) 61
19.2.3 TaMoVac Steering committee 61
19.3 Data and Safety Monitoring Board (DSMB) 61
19.4 Community Advisory Board (CAB) 61
19.5 Indication for additional review 62
20 STATISTICAL CONSIDERATIONS 62
20.1 Analysis 62
20.2 Statistical analysis 63
20.3 Volunteer characteristics 63
20.4 Safety data 63

20.5 Immunogenicity Data 63

21 ETHICAL CONSIDERATIONS 63

22 INDEMNITY 64

23 PROTOCOL AMENDMENTS 64

24 PUBLICATION 65

25. REFERENCES 66

APPENDIX 1: STUDY TIMETABLE 69

APPENDIX 2: TAMOVAC I VISIT SCHEDULE AND CRF FLOW CHART 71

APPENDIX 3: TAMOVAC I IMMUNIZATION AND LAB FLOW CHART 72

APPENDIX 4A: TAMOVAC I INFORMATION SHEET (ENGLISH) 73

APPENDIX 4B: TAMOVAC I INFORMATION SHEET (SWAHILI) 85

APPENDIX 4C: TAMOVAC I AMENDMENT 1 INFORMATION SHEET, AUGUST 2010 (ENGLISH) 96

APPENDIX 4D: TAMOVAC I AMENDMENT 1 INFORMATION SHEET, AUGUST 2010 (SWAHILI) 98

APPENDIX 5A: TAMOVAC I ASSESSMENT OF UNDERSTANDING (ENGLISH) ... 100

APPENDIX 5B: TAMOVAC I ASSESSMENT OF UNDERSTANDING (SWAHILI).... 102

APPENDIX 6A: TAMOVAC I RISK ASSESSMENT OF VOLUNTEERS (ENGLISH). 104

APPENDIX 6B: TAMOVAC I RISK ASSESSMENT OF VOLUNTEERS (SWAHILI) . 106

APPENDIX 7: TAMOVAC I PROJECT - HIV TESTING ALGORITHM 108

APPENDIX 8A: TAMOVAC I DIARY CARD (ENGLISH) 109

APPENDIX 8B: TAMOVAC I DIARY CARD (SWAHILI) 120

APPENDIX 9: DAIDS TABLE FOR CLINICAL AND LAB CRITERIA 131

APPENDIX 10: FLOW CHART FOR THE REPORTING OF ADVERSE EVENTS 147

1 STUDY TEAM

1.1 Principal Investigator

Muhammad Bakari (MD, MMed, PhD)
Department of Internal Medicine
School of Medicine
Muhimbili University of Health and Allied Sciences (MUHAS)
P.O. Box 65001
Dar es Salaam
TANZANIA
E-mail: mbakari@muhas.ac.tz, drbakari@yahoo.com
Phone: +255 754 387328, and +255 713 702211

The trial will be conducted at two sites:

1.2 Muhimbili University of Health and Allied Sciences (MUHAS)

The investigators at MUHAS are:

Site PI's:
- Muhammad Bakari
- Eligius Lyamuya

Other Investigators:

Laboratory
- Fred Mhalu
- Said Aboud

Clinical
- Patricia Munseri
- Kisali Pallangyo
- Ferdinand Mugusi
- Mohamed Janabi
- Eric Aris
- Andrew Kitua
- Sayoki Mfinanga
- Amos Kahwa

Trial nurses & Deputy Clinical co-ordinator
- Mary Ngatoluwa
- Tumaini Massawa
- Suleiman Chum

Pharmacy
- Deus Buma
- Lughano Kabadi

Data Management
- Candida Moshiro
- Kesheni Senkoro

Internal Monitors:
Asteria Ndomba,
School of Nursing, MUHAS

Eric Sandström (Clinical Monitoring)
Karolinska Institute,
Stockholm, SWEDEN

Charlotta Nilsson (Laboratory Monitoring)
Swedish Institute for Infectious Disease Control (SMI)
Solna, SWEDEN

External Monitor:
Dr Beryl Wessner
WRAIR, USA
bwessner@hivresearch.org

1.3 NIMR- Mbeya Medical Research Programme (MMRP)

The investigators at NIMR-MMRP are:

Site PI's:
- Leonard Maboko
- Arne Kroidl

Other Investigators:

Laboratory
- Frowin Nichombe (Clinical Lab)
- Angela Eser (Clinical Lab)
- Lilly Podola (Immunology)
- Christof Geldmacher (Immunology-supervision)

Clinical
- Bahati Kaluwa
- Issakwisa Mwakyula

Trial nurses
- Rosemary Mwilinga
- Tumpe Muhondwa
- Rhoda Mashauri

Pharmacy
- Revocatus Kunambi
- Theopista Lotto

Data management
- Dickens Kowuor
- Nhamo Chiwerengo

Internal Monitors
- Doreen Pamba
  MMRP
  e-mail: dpamaba@mmrp.org

- Eric Sandström
  Karolinska Institute
  Stockholm, Sweden

Independent Medical Monitor:
Stanley P. Chattanda, (MD, MMed).
Mbeya Referral Hospital, P.O. Box 419, Mbeya, Tanzania
Telephone: 255-733-208-085
Email: stanleychattanda@yahoo.co.uk

External Monitor:
Dr Beryl Wessner
Walter Reed Army Institute of Research (WRAIR),
United States of America (USA)
bwessner@hivresearch.org

1.4 Vaccine supplier

DNA and MVA will be supplied through:
Professor Britta Wahren
Department of Virology
The Swedish Institute for Infectious Disease Control
171 82 Solna
Telephone: 00 46 8 457 2630; 004670 6741527
Fax: 00 46 8 337272
Email: Britta.Wahren@smi.se

MVA will be donated to KI by the WRAIR under a Cooperative Research and Development Agreement with the Karolinska Institutet/SMI

1.5 Study laboratories

MUHAS: ***The Department of Microbiology and Immunology, School of Medicine,***
Lab coordinator: Eligius Lyamuya
Lab Manager: Emmanuel Salala

NIMR- MMRP: ***The Clinical Research Laboratory at NIMR-MMRP,***
Lab Director: M. Hoelscher
Lab Manager Clinical Lab: F. Nichombe and A. Eser
Lab Manager Immunological Lab: L. Podola and C. Geldmacher

The Swedish Institute for Infectious Disease Control, SMI:
Professor Gunnel Biberfeld
Department of Immunology and Vaccinology
Telephone: 00 46 8 457 2660
Email: gunnel.biberfeld@smi.se

Professor Britta Wahren
Department of Virology
Telephone: 00 46 8 457 2630; 00 46 7 06741527
Fax: 00 46 8 337272
Email: Britta.Wahren@smi.se

Walter Reed Army Institute of Research (WRAIR), USMHRP
Rockville, Maryland, USA
Mary Marovich
Telephone: +13012518337
Email: mmarovich@hivresearch.org

1.6 Protocol team

1. Eric Sandström (chair)

2. Michael Hoelscher (co-chair)
3. Muhammad Bakari
4. Patricia Munseri
5. Gunnel Biberfeld
6. Christof Geldmacher
7. Frances Gotch
8. Arne Kroidl
9. Leonard Maboko
10. Mary Marovich
11. Merlin Robb
12. Fred Mhalu
13. Eligius Lyamuya
14. Britta Wahren
15. Said Aboud
16. Alexandra Schütz
17. Deus Buma
18. Mohammed Janabi
19. Charlotta Nilsson

1.7 Trial Coordinating Committee, TCC

- Dr M Bakari
- Dr J Francis
- Prof E Lyamuya
- Prof F Mhalu
- Dr M Hoelscher
- Dr L Maboko
- Dr A. Kroidl (Secretary)
- Dr. Patricia Munseri
- Principal Study Nurses at MUHAS & MMRP
- Dr S. Mfinanga
- WRAIR Representatives
- Sören Andersson (For SMI)
- MUHAS appointee

1.8 Trial Management Groups, TMG at each site

At MUHAS

- Dr M Bakari
- Prof E Lyamuya
- Prof F Mhalu
- Dr. Patricia Munseri
- Dr J Mbwana
- Mr D Buma
- Dr Said Aboud
- Mr Kesheni Senkoro
- Dr C Moshiro

At MMRP

- Dr. L. Maboko
- Dr A. Kroidl
- A Eser
- D. Kowuor
- B. Kaluwa
- D. Pamba

1.9 DSMB

1. Dr Inocent Semali (MUHAS)
2. Dr Kaushik Ramaiya (Hindu Mandal Hospital/MUHAS)
3. Dr Julius Massaga (NIMR)

1.10 TaMoVac Steering Committee

1. Hassan Mshinda, Tanzania Commission for Science and Technology; Independent Chair
2. Muhammad Bakari - MUHAS
3. Eligius Lyamuya -MUHAS
4. Andrew Kitua; suppl Sayoki Mfinanga - NIMR
5. Maboko Leonard – NIMR-MMRP
6. Ilesh Jani – suppl Nafissa Osman
7. Gunnel Biberfeld; suppl Charlotta Nilsson - SMI
8. Britta Wahren; suppl Andreas Bråve - SMI
9. Eric Sandström; suppl Bo Hejdeman - KI
10. Michael Hoelscher – UM
11. Merlin Robb; suppl Mary Marovich - WRAIR
12. Gotch Frances; suppl Nesrina Imani - IC
13. Pontus Blomberg – Vecura
14. Soren Andersson (For SMI)
15. Appointee from MUHAS

1.11 Trial Sponsors

1. Professor Bakari Lembariti (DDS, PhD)
   Deputy Vice Chancellor (Planning, Financeand Administration)
   Muhimbili University of Health and Allied Sciences (MUHAS)
   P.O. Box 65001
   Dar es Salaam
   TANZANIA

   Phone: +255 22 2151306-6 Ext 219
   E-mail: dvcpfa@muhas.ac.tz;

2. Johan Carlson, (MD, PhD)
   Swedish Institute for Infectious Disease Control
   Nobels väg 18
   171 82
   Solna, SWEDEN

   Phone: +46 8 457 2310
   Fax: +46 8 30 36 68
   E-mail: johan.carlson@smi.ki.se

2 SYNOPSIS

The aim is to further explore the optimal HIV-1 DNA vaccine delivery method.

2.1 Primary objectives:

1. Determine safety of HIVIS-DNA at a dose of 600 µg or 1000 µg delivered ID in combination with MVA-CMDR boost IM
2. Determine immunogenicity of HIVIS-DNA at a dose of 600 µg or 1000 µg delivered ID in combination with MVA-CMDR boost IM

2.2 Secondary objective

1. Compare immunogenicity of HIVIS-DNA at a dose of 600 µg given as combined plasmid pools or separate plasmid pools ID in combination with MVA-CMDR boost IM

2. To build expertise and capability in evaluating HIV-1 vaccine candidates in Tanzania

2.3 Methodology

Study Sites

The clinical sites will be MNH/MUHAS in Dar es Salaam and NIMR-Mbeya Medical Research Programme (MMRP) in Mbeya, TANZANIA.

Laboratory tests will be performed at:

1. The Department of Microbiology and Immunology, MUHAS, Dar es Salaam.
2. The main and immunological laboratories of the NIMR-Mbeya Medical Research Programme, Mbeya.
3. The Swedish Institute for Infectious Disease Control, Stockholm, SWEDEN.

Additional studies may be performed at:

4. The Walter Reed Army Institute of Research (WRAIR), USA
5. The Imperial College (IC), London, UK

Study Volunteers

In Dar es Salaam: Healthy Police and Prison officers and volunteers including young adults from the general population.

In Mbeya: Healthy volunteers from the general population in Mbeya Municipality.

A minimum of 30 women will be recruited. Study volunteers should indicate low risk for HIV acquisition and we shall apply a questionnaire to exclude high-risk volunteers.

Study Design

Randomized, controlled, double blinded study.

The study will have six Groups of DNA injections as follows; all followed by MVA boosts.

**Group**	**Number of Volunteers**	**DNA immunization** weeks 0, 4 and 12	**MVA boost** weeks 30 and 46
IA	36	600 µg i.d (combined plasmid pools)	MVA, at 10^8 pfu i.m
IB	4	Saline 2 x 0.1ml i.d	Saline, im
IIA	36	600 µg i.d (separate plasmids pools)	MVA, at 10^8 pfu i.m
IIB	4	Saline 2 x 0.1 ml i.d	Saline, im
IIIA	36	1000 µg i.d (separate plasmids pools)	MVA, at 10^8 pfu i.m
IIIB	4	Saline 5 x 0.1 ml i.d	Saline, im

Randomization procedure

Block randomization in blocks of 10 with a ratio of 9 vaccine recipients to a single placebo will be used with equal distribution among the two sites.

The vaccine (immunogens)
Priming with a plasmid having HIV-1 DNA encoding the following genes:

1. Pool 1
   a. env, from HIV-1 subtypes A, B, C
   b. rev, from HIV-1 subtype B
2. Pool 2
   a. gag, from HIV-1 subtypes A, B
   b. RTmut (enzymatically inactive mutated reverse transcriptase), from HIV-1 subtype B

Boosting will be effected by a Modified Vaccinia Ankara vaccine (MVA-CMDR). It is a attenuated recombinant poxvirus vector that has been genetically engineered to express the following HIV-1 genes:

1. gp150 (Subtype E, CM235), and
2. gag and pol (integrase-deleted and reverse transcriptase nonfunctional, Subtype A, CM240).

HIV-DNA/placebo will be given at weeks 0, 4 and 12 as intradermal injections. Two HIV-MVA/placebo boost injections will be given intramuscularly at 18 and 34 weeks after the last (third) DNA/placebo priming injection, ie at week 30 and 46 respectively.

Duration of study
The enrolment period is planned to be 12 months.
The volunteers will be monitored through week 24 after the last injection ie a total of 70 weeks.

Dosage and product administration

DNA plasmids
The plasmids will be produced by Vecura, Huddinge, Stockholm, SWEDEN for the Swedish Institute for Infectious Disease Control, 171 82, Solna, SWEDEN. Vaccines will be administered i.d using a Biojector.

Group	Left Arm	Right Arm
I	1 injection id of 0.1 ml of 3mg/ml [300µg] Pool 1 and 2 (env/gag) combined	1 injection id of 0.1 ml of 3mg/ml [300µg] Pool 1 and 2 (env/gag) combined
II	1 injection id of 0.1 ml 3mg/ml [300µg] Pool 1 (env)	1 injection id of 0.1 ml 3mg/ml [300µg] Pool 2 (gag)
III	3 injections id of 0.1 ml of 2mg/ml [600µg] Pool 1 (env)	2 injections id of 0.1 ml of 2mg/ml [400µg] Pool 2 (gag)

MVA
MVA will be provided under a Cooperative Research and Development Agreement (It will be administered i.m in a dose of 10^8 pfu infectious doses.

Placebo
Sterile commercially available normal saline for human use will be used as the placebo.

2.4 Endpoints

Safety endpoints
The safety of immunization will be assessed by clinical features and standard clinical chemistry and hematological tests. Safety endpoints: Adverse events will be assessed using a standard format for soliciting local and systemic reactogenicity to the vaccine and collection of unsolicited adverse events. Solicited reactogenicity will be evaluated for 7 days following

each vaccination. All other AE will be collected from the time of first injection until the end of the study follow-up period.

Immunogenicity endpoints
The primary immunogenicity endpoint will be determined by the interferon gamma (IFN-γ) enzyme linked immunospot (ELISPOT) assay.
Secondary immunogenicity endpoints will include cellular immune responses determined by intracellular cytokine staining and T cell proliferation assays as well as binding antibody and neutralizing antibody responses.

Capacity Building end-point
A qualitative and quantitative assessment of the presence of necessary infrastructure and human capacity to conduct HIV-related vaccine studies at MUHAS and MMRP.

3 BACKGROUND AND LITERATURE REVIEW

3.1 TaMoVac Project

This is an EDCTP funded project aimed at exploring DNA prime, MVA boost HIV vaccine strategy, as well as building the capacity to perform HIV vaccine trials in Tanzania and Mozambique. It is a North-South collaborative effort involving institutions in sub-Saharan Africa (Tanzania and Mozambique) and in Europe (Sweden, Germany and the UK).

Involved Tanzanian institutions are the Muhimbili University of Health and Allied Sciences (MUHAS), Muhimbili National Hospital (MNH), National Institute for Medical Research (NIMR), NIMR-Mbeya Medical Research Programme (MMRP), and the Tanzania Police Force.

In Mozambique, the institutions are National Institute of Health (NIH), and the Maputo Central Hospital, where subsequent trials are planned-.

European institutions are the Swedish Institute for Infectious Disease Control (SMI) and the Karolinska Institute (KI) in Stockholm, Sweden; the University of Munich (LMU) in Germany, and the Imperial College (IC) of London, United Kingdom.

TaMoVac project builds upon the earlier HIV Vaccine Immunogenicity Study (HIVIS Project), a study in the United Republic of Tanzania that received support from the European Union (EU) and Sida/SAREC as a collaborative project involving the KI in Sweden, Walter Reed Army Institute of Research (WRAIR) in USA, LMU of Munich, Germany, and the University of Cape Town in South Africa. This protocol is an extension of that programme with the leadership moved to the African institutions of MUHAS and NIMR-MMRP.

3.2 The global HIV-1 Situation

By the end of 2007 it was estimated that about 33.2 (30.6-36.1) million people globally were living with HIV. The annual number of new infections declined from 3.0 million (2.6 million – 3.5 million) in 2001 to 2.7 million (2.2 million -3.2 million) in 2007. Observe that the overall number of people living with HIV has increased as a result of ongoing number of new infections yearly and the beneficial effects of the antiretroviral therapy. Most of these infections are in the Sub-Saharan Africa accounting for 67% of all people living with HIV and 72% of AIDS deaths [1]).

There are also trends of declines in HIV prevalence in some African countries like Uganda, Kenya and Zimbabwe and other parts of Africa, coupled with indications of significant behavioral change. This includes increased condom use, fewer partners and delayed sexual debut. In the rest of sub-Saharan Africa, the majority of epidemics appear to be leveling off

but at exceptionally high levels in most of Southern Africa which alone accounted for almost one third (32%) of all new HIV infections and AIDS deaths globally in 2007 [2].

Despite of that, Africa remains the global epicenter of the AIDS pandemic. South Africa's AIDS epidemic is the highest in the world and shows no evidence of a decline. An estimated 5.1 [4.6 – 5.6] million people were living with HIV in 2005 in South Africa. In Botswana, Namibia and Swaziland the exceptionally high infection levels continue. Botswana's national adult HIV prevalence was estimated at 24.1% [23.0%–32.0%] in 2005, while in Swaziland the national adult HIV prevalence is estimated at 33.4% [21.2%–45.3%], with HIV prevalence among pregnant women attending antenatal clinics that rose from 4% in 1992 to 43% in 2004. The HIV prevalence in Lesotho's was 23.2% [21.9%–24.7%]. More worrying is the fact that a dynamic epidemic is underway in Mozambique, where the estimated national adult HIV prevalence is 16.1% [12.5%–20.0%]. In this country, HIV is spreading fastest in provinces linked by major transport routes to Malawi, South Africa and Zimbabwe [3].

It should be noted that HIV infection in this part of the world is largely due to HIV-1 (as opposed to HIV-2) and the predominant mode of transmission is heterosexual contact.

3.3 HIV-1 situation in Tanzania

The first three cases of AIDS in Tanzania were reported in 1983 from the Kagera region in the northwest, and were serologically confirmed two years later. It was also shown then that different groups of populations had different prevalences of HIV. The National AIDS Control Programme (NACP) estimated that by the end of 2005, 1,770,383 individuals were living with HIV and AIDS, with 656,180 derived from urban and 1,114,203 from rural areas despite great efforts with health preventive education and other interventions [4].

Though the actual prevalence of HIV-infection still varies from place to place, the National AIDS Control Programme estimated the national average for the adult population at 7.9% in the year 2005. In the most populous city, Dar es Salaam, the seroprevalence among pregnant women and sex workers was 10.9% [4] and between 40 and 60%, respectively. The prevalence among hotel workers was 10.4% [5] and among members of the police force and antenatal clinic attendee's prevalence was found to range from 13-18% [4, 6]. Among blood donors at Muhimbili National Hospital (MNH) the prevalence of HIV was 8.7% [7]. The age group that is particularly affected is 20-49 years, a group that is reproductively important, and also the economically most productive [4]. The most recent data as reported in the Tanzania HIV and Malaria Indicator Survey (THMIS) of 2007- 08 revealed the overall prevalence of HIV/AIDS to be 6%. The prevalence was higher among women than men, being 7% and 5% percent respectively.

In the Mbeya region, the Regional AIDS Control Program has conducted sentinel surveillance amongst women attending antenatal clinics for the past 19 years. These data have been recently complemented by the MMRP with in-depth analysis of specific study populations (CODE and EMINI studies). In 1988, the HIV prevalence varied between 4-8% amongst pregnant women [8]. By 1995 the prevalence had increased substantially in all antenatal centers to 11.9% overall with higher prevalence in urban sites (17%) [9]. In addition to data in pregnant women, MMRP collected population-based HIV prevalence data during the recruitment of its study populations (cohorts). These data correlate very well with the data in sentinel surveillance. The prevalence in the high-risk population of women working in bars (HISIS) is as high as 68% [10], in the urban general population (18-45 years) 19.6% and in the rural population 14.4% (CODE) (unpublished data). Incidence in these populations varies but it is in excess of 1.4%. The EMINI population data revealed that males and females are equally affected, however women are infected at a younger age than men, but the prevalence of men above 50 years was much higher than that in women (unpublished data).

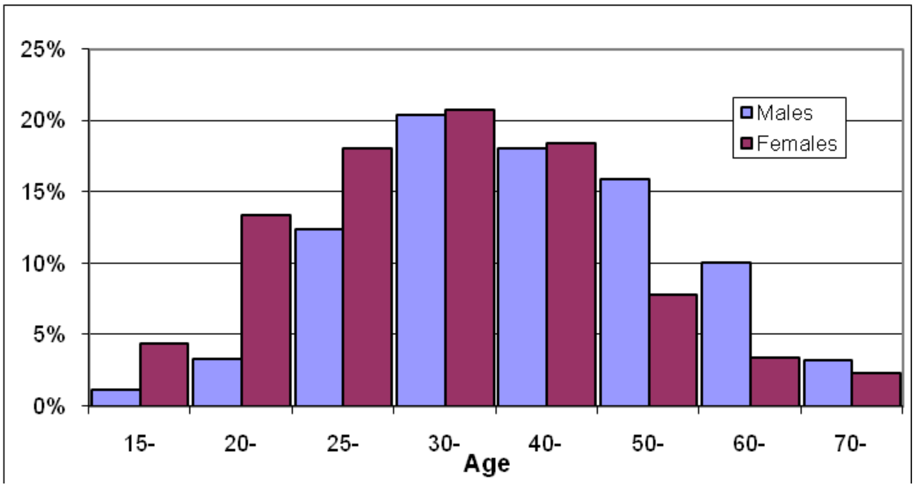

Data based on 9615 adolescent and adult participants from 9 different communities in Mbeya region

Studies conducted in Dar es Salaam, Mbeya and Kilimanjaro have shown that the important circulating HIV-1 subtypes in Tanzania are clade A, C, D and their circulating recombinant forms [11-14].

3.4 Need for an HIV vaccine in Africa

While antiretroviral drugs have shown great promise in reducing the replication of HIV and thus in reducing HIV and AIDS associated morbidity, mortality and HIV transmission, the cost is substantial and side effects are a potentially limiting factor. As shown above from the Global AIDS report the sub Saharan Africa is most affected in terms of prevalence and mortality therefore development of an effective safe and affordable vaccine is likely to be the best way to stop further spread of the virus. Despite the on-going efforts, many uncertainties still remain about the most appropriate vaccines, the best effective timing of the immunization and correlates of protective immunity.

An AIDS vaccine is urgently required for Sub-Saharan Africa and special efforts to support the development of such a vaccine relevant to Africa in the shortest possible period are needed for the following reasons:

- The majority (68%) of new HIV infections in the world continue to occur in Africa and unlike other regions of the world up to 61% of those infected in Africa are females.
- AIDS has serious and devastating social, economic, health and security consequences in Africa where 1.6 million out of the estimated 2.1 million (76%) deaths during 2007 globally, occurred in the region.
- Treatment for HIV and AIDS including access to antiretroviral therapy is not yet readily available to most affected individuals in Africa and will therefore, be unlikely to suffice in HIV prevention and control.
- Private sector market driven HIV vaccine research and development has mainly been directed to HIV-1 of subtype B, while in Africa many other HIV-1 subtypes are prevalent.

3.5 Need for developing countries especially in Africa to participate in HIV vaccine development and trials

Immunogenicity of vaccines in industrialized world populations may not necessarily predict the vaccine induced response in developing countries. Nutritional deficiencies, genetic differences, HIV strain characteristics and microbial burden of endemic infections in

individuals may alter immune constitution. Such differences have been documented with polio, rabies, and yellow fever vaccines.
In addition, the distribution of Class I and Class II HLA types is determined genetically and is highly divergent across populations of different racial origins. Both humoral and cellular immune responses are determined to a large extent by the particular HLA Class I and Class II types in vaccine recipients. Therefore, the humoral and cellular immune responses to immunogens administered in the developing world should be compared with those responses among individuals in the developed world receiving the same vaccines. Not only HLA, but also the incidences of viral co-receptor deficiency, of chemokine and cytokine polymorphism and of intracellular viral inhibitors vary between races and between individuals.

Also the common infectious disease epidemiology such as activation of the immune response to vaccine or vaccine vectors differs. A more sophisticated analysis of the individual response and susceptibility to vaccine or virus infection might then be possible in the suggested vaccine cohorts.

The conduct of HIV vaccine trials will contribute to capacity building and infrastructure development in Africa. In addition, if African countries are not involved in HIV vaccine research and development, clinical trial findings and benefits from developed countries may not be generalizable and may take a long time to benefit Africa.

Participation of Africa in HIV vaccine trials will also provide an opportunity for scientists to generate results in a shorter period using a small sample size due to the high incidence of HIV in the region. By participating in vaccine trials, African countries will have ownership of research results and thus guarantee that candidate vaccines found to be safe and efficacious would be available for Africa.

In this project, PhD students/trainees will finalize or register for training at the Karolinska Institute using data generated from the trial. Necessary laboratory equipment for monitoring immune responses such as Colour Flow-cytometers as well as appropriate training will be made available to the MUHAS and MMRP sites. Furthermore, GCP and GCLP training will be conducted for all staff that will be involved in the trial. There will also be built capacity for data management at NIMR.

The expertise and competence acquired will subsequently form an international network of Scientists that will continue to spearhead the development of HIV vaccine.

3.6 Tanzania's National HIV Vaccine Strategic Framework

It is of note that the ongoing HIVIS Project was conceived as a consequence of the first National HIV Vaccine Strategic Plan Workshop in Tanzania that took place in July 2001 at the Bahari Beach Hotel, Dar es Salaam, which received support from the WHO-UNAIDS HIV Vaccine Initiative, the Sida/SAREC program of Sweden, the European Union, the Walter Reed Army Institute and the Henry M. Jackson Foundation both of the U.S.A. Government.

A further workshop to develop Tanzania's National HIV Vaccine Framework was conducted in mid-September 2004. This workshop was supported by, among others, the WHO/UNAIDS and the African AIDS Vaccine Programme (AAVP).

The framework aims to guide the development and evaluation of HIV vaccines in the country, with a vision being to ensure availability of effective, safe, affordable and accessible HIV vaccines for the benefit of all Tanzanians. The mission is to promote research, development, production and evaluation of suitable HIV vaccines and ensure sufficient availability of the vaccine in an equitable way for the entire needy population of the country, through national,

regional and international collaboration. The framework document was approved by the Tanzania Government's Ministry of Health in February 2005.

3.7 African experience in AIDS vaccine research and development

In collaboration between developed countries and several African countries, efforts are being made to develop suitable HIV vaccines for the continent. By 2008, eleven phase I trials and four phase I/II HIV vaccine trials have been or are being conducted in sub-Saharan Africa (IAVI AIDS Vaccine Trials Database Report 2007). The first one, conducted in Uganda in 1999, was sponsored by the National Institute of Health (NIH) of the United States of America. In this ALVAC-HIV prophylactic HIV vaccine study, a canary pox vector containing HIV-1 clade B antigens was used. Immunogenicity was low but the vaccine elicited CD8+T-cell responses with detectable cross-activity against clade A and D antigens in a significant proportion of vaccine-recipients [15-16]. Three other trials were sponsored by the International AIDS Vaccine Initiative (IAVI) and were conducted in Kenya in 2001 and 2002, with one on-going in Uganda. The vaccine concept being evaluated was based on a prime-boost combination using DNA and MVA candidate vaccines expressing a number of genes from clade A HIV-1 strain. It is reassuring that preliminary results from these trials have revealed that the candidate vaccines were safe and well tolerated [17]. To date there are completed or on-going phase I or I/II HIV vaccine trials in Uganda, Kenya, Rwanda, South Africa, Tanzania, Zambia and Botswana. Some of the organizers/sponsors of these trials include International AIDS Vaccine Initiative (IAVI), National Institute of Allergy and Infectious Diseases (NIAID), HIV Vaccine Trials Network (HVTN), Merck, South African AIDS Vaccine Initiative (SAAVI), Kenya AIDS Vaccine Initiative (KAVI), Uganda Virus Research Institute, AlphaVax, Targeted Genetics, US Military HIV Research Programme (USMHRP), the European Union, Sida/SAREC (Sweden) and the Muhimbili University of Health and Allied Sciences (MUHAS). There are also VRC TRIAD trials, such as RV172, IAVI V001 and HVTN 204.

These trials involve vaccines that are DNA based with HIV-genes, and some include priming and boosting strategies with various viral vectors including adenovirus or vaccinia virus vectors [18].

3.8 Global efforts to develop an AIDS Vaccine

In many countries, development of candidate HIV vaccines is at an advanced stage, with some already evaluated in clinical trials.

The first human trial of an HIV vaccine candidate was conducted in the United States in 1987. While the first generation of vaccine products was based on recombinant proteins (gp120, gp140, V3) and elicited mainly antibodies, the current generation of vaccines is based on vectors, DNA or the combination of both.

The first phase I/II vaccine trial in a developing country was conducted in China in 1993 [18]. Since then, more than 20 phase I/II trials have been completed in developing countries, the majority in Thailand, but also in Brazil, Cuba, Haiti, Kenya, Peru, Tanzania, Trinidad, Uganda, Rwanda, Zambia, Botswana and South Africa. Vaccine concepts which have been tested include: envelope based candidate vaccines (gp120, V3 peptides and V3 protein); Canarypox and Modified Vaccinia Ankara (MVA) vectors; DNA constructs; prime-boost combinations including DNA/MVA; multi-epitopes DNA vaccines, Adenovirus vectored vaccines, and VEE vectored vaccines.

The first phase III HIV vaccine trials, assessing the protective efficacy of two different versions of a gp120 candidate vaccine, were initiated in North America and Europe in 1998 and in Thailand in 1999. Preliminary results from the North American trial became available in February 2003, and they showed that while the candidate was safe the vaccine failed to confer protective efficacy in the majority of the population [19]. A preliminary subset analysis of less than 10% of the enrolled volunteers suggested vaccine efficacy among black volunteers; however, the interpretation of those data is still being debated. The data also

suggested that women produced higher levels of antibodies than men and that vaccinated volunteers preferentially excluded viruses resembling vaccine antigens (virus sieving)[19]. Those results are controversial. Results from the Thai trial indicated that the vaccine was safe but failed to confer protection [19].

A second phase III trial, with an Alvac vector and recombinant gp160, started in Thailand in 2003 and just recently results were released from the trial showing a 30% reduction in infection rate in the vaccinees as compared to placebo controls [20].
Two large phase IIB trials, STEP and Phambili, of a non-replicating recombinant Adenovirus 5 vector with HIV subtype B genes *gag*, *pol* and *nef* have been terminated due to futility of proving vaccine efficacy during 2007.

In Tanzania two HIV-1 candidate vaccines were tested in 2006/2007 in Mbeya and in Dar es Salaam. Both vaccine strategies were based on a DNA prime with a vector boost. Both approaches used DNA priming and either Ad5 or MVA boosting. Together 120 individuals have been vaccinated without major vaccine related side effects. Both approaches used in Tanzania represent state of the art of current vaccine development strategies.

4 Implications of recent HIV vaccine trial results

Over the past years there had been a new optimism that preventive HIV vaccines were feasible sparked to a large degree by the Merck sponsored phase IIB trials, STEP and Phambili, of adenovirus vectors with HIV subtype B genes [21-22]. The closure of those trials due to futility has come as a great disappointment and is aggravated by the observation that individuals with preexisting immunity to the adenovirus serotype 5 vector seem to have a higher risk of becoming HIV infected after vaccination with an adenovirus vector based HIV vaccine. It is unknown if this was a vector specific affect or if a sufficiently different vector or absence of pre immunity can avoid this. Nor is it known if this was due to other factors such as concomitant HSV-2 infection or other unknown factors that caused a reduced transmission among placebo recipients. It must however for safety reasons be assumed that any HIV vaccine can potentially cause an increase in transmission until proven otherwise. This should be evident from the information material as well as the safer sex guidelines even in early trials like this where HIV infections should be avoided as a principle. In fact the largest identified risk factor was the lack of circumcision. The Relative Risk (RR) in circumcised men without prior adenovirus infection was below 1; however the confidence interval was very large.

There has been an intensive discussion within the scientific community whether vector based vaccines that primarily induce cellular immunity should be abandoned. And while this discussion is still ongoing there seems to emerge increasing support for the notion that the observations of the Merck trial cannot be generalized to all vector based vaccines.

Pox vectors have been shown to be immunogenic in humans, safe according to numerous studies conducted to date and associated with protection in animal models.
It is our view that induction of strong cell mediated immunity will be the necessary base on which future vaccines that also induce humoral and mucosal immunity, will build. Major differences between the different vaccine approaches are listed in the table below.

While poxviruses as boosts were initially favored, they were subsequently thought to be less efficient following trials in England and Kenya. However, recently the EuroVacc consortium and the HIVIS program (ourselves) have been able to show excellent boosting capacity of two vaccinia-based vectors. Vaccinia is a stable vector that can carry large foreign inserts and replicates only in the cytoplasm. Therefore the risk of integration of inserted genes or viral genes in the host genome is very low. The trials with the vaccinia-based vaccines MVA and NYVAC also to some degree reflect the discussion about the desirability of ‘broadness’ of the immune response in that the former, used in this application, favors a multiclade

vaccine, while the latter is purposely restricted to a homologous clade C prime-boost platform. Such breadth has been demonstrated by a broad response to both gag and env in the HIVIS vaccine schedule, as opposed to an env-oriented response in the EuroVacc study [23].

Given the low immunogenicity of the DNA priming alone used in above-mentioned studies, we set out to optimize DNA delivery in the HIVIS studies. We have good evidence that we can reduce the amount of DNA in the priming significantly by intradermal delivery by Biojector and/or by electroporation. Our aim now is to lay the foundation for further studies of DNA delivery together with electroporation. This methods have been shown to induce good antibody production in addition to strong stimulation of the cell-mediated immunity in preclinical studies [24].

As stated above, recently, we and the EuroVacc consortium have shown excellent boosting capacity of two vaccinia virus vectors with stable and efficiently expressed HIV inserts. Vaccinia immunity is absent in people younger than 35 years of age. While adenovirus might persist in mucus membranes of adenovirus-infected individuals and cause local immune activation after an adenovirus immunization, this risk does not exist with poxviruses.

4.1 Table 1: Differences of immunogens and delivery by different recent prime boost candidate HIV DNA HIV-1 vaccines

**Category/ Trials**	**Merck STEP**	**VRC product PAVE 100**	**HIVIS DNA/WRAIR MVA HIVIS/TaMoVac**	**EuroVacc EuroVac/AfrEvac**
**Schedule**	*Repeated dosing* with homologous vector	**Heterologous** vector prime-boost	**Heterologous** vector prime-boost	**Heterologous** vector prime-boost
**Components**	3 rAd5 vectors	6 **DNA** plasmids<br>4 rAd5 vectors	7**DNA** plasmids<br>1 **MVA** vector	2 **DNA** plasmids<br>1 **NYVAC** vector
**Vector**	Adenovirus : rAd5 E1-deleted; PerC.6; x3	Adenovirus rAd5 E1, **E3, E4-deleted**, 293-ORF6 **Boost x1**	**Vaccinia** virus MVA *Env* truncated, RNaseH and *int* deleted, RT inactive. **Boost x1 (-2)**	**Vaccinia** virus NYVAC **codon optimized**. gag/pol/nef + env. **Boost x 2**
**Vector dose**	10^10	10^10	**10^8**	**10^7.7**
**Antigens**	*Gag, Pol, Nef*	***Envx3****,Gag, Pol, Nef*	***Envx3****,Rev,***Gagx2****, RT/Pol*	***Env****, Gag, Pol, Nef*
**HIV subtypes**	B	**A,** B, **C**	**A, B, C, E**	**C**
**Table 1. Continued**				
**Delivery**	rAd5 by needle and syringe im	DNA by **Biojector** im<br>rAd5 by needle and syringe im	DNA by **Biojector i.d/** im<br>MVA by needle and syringe im	DNA and NYVAC by needle and syringe im

**Vector immunity**	Adenovirus 5 immunity common in D.C.	Adenovirus 5 immunity common in D.C.	**No vaccinia immunity** in people younger than 35 years	**No vaccinia immunity** in people younger than 35 years
**Vector repl.**	Nucleus; persistent	Nucleus; persistent	**Cytoplasm; not persistent**	**Cytoplasm; not persistent**
**ELISpot resp**	50-80%	>80%?	**>90%**	**>90%**

Vaccine strategies attempting to induce both enhanced T cell and antibody responses have focused primarily on the prime boost strategy [18].
It has been suggested that HIV-1 specific T cell responses may facilitate protective immunity. Individuals who are exposed to HIV-1, but do not become persistently infected, develop HIV-1 specific cytotoxic T lymphocytes (CTL) and T-helper lymphocytes without the generation of systemic HIV-1 antibodies, although mucosal HIV-1 antibodies have been detected [25-26]. The generation of CTL and T- helper responses correlates in time with the control of acute HIV-1 viremia in humans and macaques. Such a correlation is not seen with antibodies and therefore, the induction of HIV-1 specific CTL and T-helper responses is widely seen as critical to the success of an HIV-1 vaccine.

4.2 DNA HIV Vaccines

Plasmid DNA has been shown to induce primarily cell-mediated memory immune responses, but these responses have generally been weak. They have been successfully boosted by immunogens included in various viral vectors, such as vaccinia virus, fowlpox virus or adenoviruses. However the doses of DNA that need to be given are substantial and it would be a great advantage if more efficient delivery modes/routes can be established.

The optimal antigens to be used in a vaccine have not been determined. At this point it is advisable to include as many as practically feasible, as long as there is no risk of reconstructing a viable virus. **Env** is desirable primarily in order to raise antibodies. Since it is highly variable it is prudent to try to match it as to the predominating subtypes. **Gag** and **RT** are known to give rise to potent CTL responses in HIV-1 infected individuals and these proteins are less likely to vary. Additional desirable immunogens would be early regulatory proteins such as the genes for **nef** and **tat.** In the current trial plasmids containing genes for **env** of subtypes A and C are included since these are the major subtypes present in Tanzania [11-14, 27]. **Env** of subtype B is included since it is closely related to subtype D that also is circulating in Tanzania, and is also highly immunogenic
The cross-reactivity between HIV-1 subtypes is supported by the following data. Immunization with a recombinant glycoprotein 160 envelope immunogen derived from a virus of genetic subtype B was shown to induce strong specific T-helper cell responses in asymptomatic human immunodeficiency virus (HIV) carriers infected with subtypes B to G [28]. Patients infected with subtype D thus responded to the subtype B vaccination with the B envelope protein. Other cross-reactivities also occurred. This indicates that both homologous and heterologous antigens can induce the HIV-specific T-helper immunity, important for the development of antibodies and cytotoxic T lymphocytes. It also suggests that a particular immunogen can be effective against several different HIV strains. Furthermore, inactivated virions of subtypes A, B, and D viruses were evaluated for their ability to stimulate T cell responses in PBMC's. Rutebemberwa and colleagues showed 2007 that individuals with subtype B infection responded well to whole viral preparations of subtype D with CD8 and CD4 reactivity [29]. It was also shown that certain Ugandan individuals infected with non-D strain could react with the subtype D virions. There is also preliminary data from Britta

Wahren's laboratory whereby mice have been immunized with the planned DNA HIVIS immunogens. After three electroporations reactivity was seen to the immunogens, but also to subtype D envelope.
Subtype B is the dominating subtype in Europe and the US. **Gag** of subtypes A and B are included for the same reason. The vaccine is complemented with **RT** of subtype B, since the pol gene does not vary so much between the subtypes. **Rev** of subtype B is included to augment the expression of the envelope genes.
The favored route of injection has been intramuscular injection by needle. Recently immunogenicity has been increased by using a needle-free device that disperses the plasmids to a wider area in the tissue. It has also been found that intradermal injections will target antigen-presenting cells that are present in the skin. Intradermal injections given by needle are difficult to standardize due to technical difficulties. Injection by needle-free devices can potentially offer advantages such as better dispersion of vaccine in tissues, standardized inoculation and less risk of exposure to unsterilized needles. One such commercially available device is the Bioject®, which is to be used in this study. The most important advantage of the intradermal (i.d.) Biojector is the delivery of DNA intradermally in a controlled manner, where it can be expressed as an endogenous protein in transfected cells. Disposable single use devices are now available which addresses the risk for cross-contamination in previous studies with needle-free devices.

All DNA vaccine immunogens described are encoded in expression vector pKCMV, which contains the promoter sequence from CMV, the poly (A) signal from HPV-16, the E.coli origin of replication and encodes kanamycin resistance. The pKCMVgp160B encodes gp160 of subtype B (a fusion protein of gp120 and gp41).The pKCMVgp160B/A and pKCMV gp160B/C encode chimeric gp160B proteins with the hypervariable loops (V1-V5) exchanged for subtype A or C sequences, respectively. The p37 gag subtypes A and B constructs encode the antigenically most relevant p17 matrix and p24 capsid proteins. p24 is the main vaccine target of the gag proteins and the exchange of p17 subtype A for the subtype B p17 will increase the response to conserved epitopes. The seven plasmids, expressing ten different HIV-1 genes, have previously been delivered at two different sites; one containing the p37 (ABA and B) and RT genes and the other the gp160 (A, B and C) and rev genes, in order to avoid A possible interference between vaccine antigens [30].

4.3 Modified Vaccinia Ankara (MVA)

MVA-CMDR is a recombinant attenuated modified vaccinia virus-vectored vaccine that has been genetically engineered to express HIV-1 gp150 (Subtype E, isolate CM235) and gag and pol (integrase-deleted and reverse transcriptase nonfunctional, Subtype A, isolate CM240**)** developed at the Laboratory of Viral Disease (LVD), that does not replicate in mammalian cells, National Institute of Allergy and Infectious Diseases (NIAID) by Bernard Moss and Patricia Earl. The vaccine preparation is formulated to contain 10^8 plaque forming units (pfu)/mL in sterile PBS (without Ca^{2+} and Mg^{2+}), 7.5% lactose, pH 7.4. The vaccine is filled in 2 mL glass vials in a volume of 1.2 mL (10^8 pfu/mL) or 0.4 mL (10^9 pfu/mL). The vials are stoppered with gray butyl rubber stoppers and sealed with aluminum crimp seals. Storage of the MVA-CMDR vaccine is at -80°C (± 10°C).

Rationale

The use of live attenuated Modified Vaccinia Ankara virus recombinants as expression vectors for heterologous HIV gene inserts has been shown in clinical trials to be safe and immunogenic [31-34]. In particular, Modified Vaccinia Ankara (MVA)-based recombinants have been shown to induce antibodies and specific cellular immune responses mediated by CTLs. The Walter Reed Army Institute of Research/National Institutes of Health (WRAIR/NIH) have produced a live recombinant poxvirus vector, MVA-CMDR, that is genetically engineered to express the following HIV genes: gp150 (Subtype E, CM235), gag, and pol (integrase-deleted and reverse transcriptase nonfunctional, Subtype A, CM240). This live attenuated vector vaccine candidate has been shown to be safe and immunogenic

in two Phase I protocols, either administered alone (RV158 US, Thailand) or when used in a DNA prime/MVA boost immunization strategy (32, Tanzania-ongoing).

The MVA product is a derivative of vaccinia virus. Recent batches of unattenuated vaccinia (small pox) vaccine have been associated with rare but severe cardiac complications after administration. The US FDA thus routinely requires additional monitoring for cardiac complications after administration of small pox vaccine, MVA and similar pox virus vectors even though the cardiac complications associated with vaccinia have not been reported with MVA. In addition, the US FDA has required investigators to exert a higher degree of scrutiny to prospective volunteers to reduce the risk of cardiac complications. Although this study is not conducted under IND to the FDA, the manufacturer of the MVA will meet both local standards and those of the US FDA in its investigational program of MVA. So far MVA has not been associated with any cardiac toxicity in any of the ongoing studies.

4.4 Prime – boost strategy

The rationale for the combined prime-boost regimen stems from pre-clinical data demonstrating_that priming with DNA vaccines and boosting with poxvirus vaccines induces stronger cellular immunity than either DNA or MVA alone. The DNA prime is thought to focus the immune response to the desired antigens, whereas the recombinant pox virus booster immunization is thought to boost this response, both by expressing higher levels of recombinant antigen and by the immunostimulatory activity of a pox virus infection. Excler [35]and Plotkin [36] have recently reviewed prime-boost approaches to HIV preventive vaccination. They described animal data, as well as results from initial phase I clinical trials that assessed prime-boost regimens in HIV-1/2 uninfected volunteers using poxvirus-based HIV recombinants to prime and recombinant envelope glycoproteins to boost HIV-specific immune responses. The prime-boost approach induced both HIV-specific humoral and cell-mediated responses and conferred partial protection in animal models.

4.5 A Phase I HIV-1 vaccine trial in Sweden

A randomized, open label, phase I trial to assess the safety of different modes of administering the same DNA vaccine candidate (HIVIS-DNA, a plasmid DNA with inserted HIV genes env, rev, gag and RT) has recently been completed in Stockholm, Sweden. The combination of HIVIS-DNA and MVA-CMDR proved safe and highly immunogenic. Thirty-four out of 37 vaccinees (92%) having valid assays became positive in the Interferon gamma (IFN-gamma) ELISPOT assay, 32 vaccinees reacted to Gag peptide pools (p17 and p24) and 24 to Env peptide pools (gp120 and gp41). When combined with lymphoproliferative assays used to identify HIV-specific immunity, all but one vaccinees (37/38, 97%) developed HIV-specific immune responses. All DNA injections were delivered by the Biojector needle-free device as is happening with the current HIVIS03 trial in Dar es Salaam, Tanzania. Preliminary analysis indicate that ¼ of the amount of DNA given i.d. primed equivalently to a 3.8mg dose given i.m., and that boosting with HIV-MVA can be achieved in volunteers despite previous small pox immunization. It was also shown that 10^8 pfu HIV-MVA i.m. gave a better boosting of the HIVIS-DNA priming than did 10^7 pfu given i.d. Furthermore, volunteers below the age of 40 years exhibited higher levels of immune responses as compared to the other volunteers (>40 years) whose immune responses were markedly dampened [37]. The vaccines and delivery methods were well tolerated and not related to any grade 3 or 4 adverse events.

4.6 Preliminary work on HIV vaccine trials in Dar es Salaam

Tanzania was among the first countries assessed by WHO in 1994 and recommended to be considered as a potential country for future HIV/AIDS vaccine trials. To that end, a cohort of police officers was recruited to assess its suitability for future HIV vaccine trials by determining the prevalence and incidence of HIV-1 infections. The number of police officers in Dar es Salaam is around 6,000 and they have a relatively low turnover. The overall HIV-1 sero-prevalence at recruitment among 2850 police officers recruited in 1994-1996 was found

to be 13.8%. The overall crude HIV-1 incidence was 19.6/1000 PYAR, a figure that is within the 10-30/1000 PYAR interval recommended by the WHO for efficient vaccine trials [6]. These results indicated that the police officers cohort is a potentially suitable population for vaccine trials. With the help of Sida/SAREC funding, there were also efforts to develop the human resource and laboratory infrastructure necessary for the conduct of HIV vaccine trials. A number of Scientists have hitherto been trained at different levels. They included Scientists as well as laboratory technicians at the Muhimbili University of Health and Allied Sciences (MUHAS). The laboratory at the Microbiology/Immunology department was also improved in terms of space and needed equipment to enable screening and safety evaluation (HIV serology, biochemical tests, as well as hematological tests) of potential volunteers as well as monitoring of immune responses following vaccination (Interferon-gamma ELISPOT assay). In collaboration with the MNH, a dedicated HIV clinic was initiated that allowed for the ethical conduct of HIV vaccine trial since there was now a place where volunteers found to be HIV infected could be attended to. Indeed these were the preliminaries that paved the way for the HIVIS03 trial in Dar es Salaam.

Local reference values have been determined for T-lymphocyte subsets, haematological and biochemical parameters using samples from police officers and healthy Tanzanian blood donors. BD 4-colour FACSCalibur, Beckman Coulter AcT5 Diff hematology and Roche Cobas Integra 400 Plus analyzers were used for determination of CD4/CD8 T-cell counts, haematological and biochemical reference values, respectively. Internal quality control program is strictly implemented including daily runs of quality control (low, normal and high for haematology, normal and abnormal for biochemistry, negative and positive for qualitative assay and, negative, low positive and high positive controls for viral load) for every test/assay, regular preventive maintenance services for equipments/instruments, recalibration of analyzers at recommended intervals, monitoring temperature sensitive equipments/instruments, use of SOPs for each assay/test performed and documentation in the daily operational, reagent, calibration, temperature and corrective action logs. In keeping with GCLP, the Microbiology and Immunology Laboratories at Muhimbili University of Health and Allied Sciences routinely participate in the proficiency testing from the UKNEQAS and QASI EQA programmes for CD4/CD8 T-cell immunophenotyping; CAP EQA programmes for hematology, virology and biochemistry; the WHO/NICD and CDC quality assurance programmes for bacteriology and serology and Virology Quality Assurance (VQA) program for HIV-1 DNA PCR and viral load. During the study strict adherence to SOPs will be observed and all laboratory test results will be checked by a senior laboratory technician and verified by a Clinical Microbiologist/Immunologist before dispatch to the clinics.

Assays for detection of HIV-specific cell mediated immune responses have been established and standardized. The cell preparation technique employed has been carefully selected prior to being evaluated (Nilsson et al. Clinical Vaccine Immunology 2008). Standard operating procedures have been developed for each of the methods used. Furthermore, the assay used as primary readout (ELISpot) has also been validated using samples collected from healthy Tanzanian donors. This has enabled establishment of internal controls as well as cut-off values. An internal control is used on each ELISPOT plate as part of quality control. Also for quality control purposes, PBMC samples are provided from the Central study lab and testing in ELIspot is performed every other month. Quarterly on-site laboratory review is planned. ICS assay using 4-colour FACSCalibur has been currently established and optimized for use in the monitoring of immune response in the ongoing HIVIS03 trial.

All laboratory staff have undergone GCLP training and repeat training is performed every other year. Operators performing the ELISpot assay have received training and passed an operators validation test which involves running three known samples with low, medium and high IFN-gamma ELISpot reactivity to a peptide pool on three different occasions. To pass the operator validation test the average percent coefficient of variation (%CV) should not exceed 20%.

4.6.1 HIVIS 03

The preparation for the continued phase I/II trial in Tanzania, HIVIS 03, has been successfully completed and the study is now fully enrolled with the required 60 volunteers, all of them Police Officers. The volunteers are of less than 40 years of age, HIV negative and generally healthy at recruitment. This is a randomized, placebo controlled trial with DNA priming and MVA boosting and preliminary results have shown excellent safety, and that the level of immunogenicity after the DNA prime alone could indeed be better than that found among the Swedish volunteers in HIVIS01 [38]. The HIVIS03 study is still blinded, and follow up of volunteers is continuing. All 50 eligible volunteers have received the three DNA injections and one booster MVA injection. The vaccines and delivery methods were well tolerated and not probably or definitely related to any grade 3 or 4 adverse events. Although the study is still blinded the immunogenicity results are similar to, or better than, those obtained in HIVIS01/02. Out of 59 vaccinees who received 3 DNA/placebo, 23 (39%) had HIV-specific IFN-gamma ELISPOT responses. On the other hand, 34 (68%) of 50 volunteers who received a MVA/Placebo boost after DNA/placebo priming had HIV-specific IFN-gamma ELISPOT responses two weeks after the boost. One third got placebo. The study is still blinded.

The continued conduct of this trial will ensure the sustainability of the whole trial organization at the Dar es Salaam site. The current programme is limited to a short follow-up after a single MVA boost, which is clearly insufficient to properly evaluate strength and durability of the immune response. The vaccine producer, WRAIR, has decided to donate further immunogens to enable a continuation with a second boost, which is currently ongoing.

4.6.2 Cohort capacity building

Multiple cohorts for phase IIB and III studies will soon be needed in many places in Africa. We have worked for many years to develop such cohorts. However, only in the context of real trials will the efforts be tested to actually be ready to perform such trials. It is furthermore very difficult to maintain the cohorts and the supporting mechanisms without the presence of ongoing trials. Such a cohort among Police Officers is currently maintained in Dar es Salaam and serves as the basis for the recruitment for the ongoing HIV vaccine trial, HIVIS 03. This cohort must be maintained and further developed in the context of a future phase II HIV vaccine trial.

In Mbeya, preparations for HIV vaccine evaluations started in 1999 with the establishment of two major cohorts which included a high-risk cohort of 600 bar workers (HISIS) and a general population cohort of 3000 adults (CODE). In 2006 a third, even larger cohort (EMINI) of 18000 individuals (above the age of 6 months) was initiated. Follow-up intervals are different in each of the cohorts. While bar workers form the HISIS cohort were followed up every 3 months, CODE participants were followed up every 6 months and EMINI participants are followed up once a year. These cohorts do not only provide detailed incidence rates and therefore allow precise planning of future vaccine trials, but also have created trust in potential vaccine populations between researchers and participants.

4.6.3 Police Officers Cohort in Dar es Salaam

The incidence and prevalence of HIV was investigated in 2,850 police officers in 1994-96 in the TANSWED programme, as mentioned above. It proved feasible to work ethically in this hierarchical group and the incidence of 1.96% together with a low turnover of personnel indicated that this group represented a potential HIV vaccine phase III cohort. Good relations have been maintained with the cohort since then and it was again approached in 2004 to prepare for a new round of incidence studies, this time coupled with questions about HIV vaccine acceptability. In the process a core group in the police force interested in working against HIV transmission was identified. This group now consists of more than 400 police officers that have been further trained to serve as the primary recruiting ground for further phase I and II studies and to facilitate future phase III HIV vaccine studies in the Police

cohort. On-going socio-behavioral studies on various aspects related to HIV and participation in HIV vaccine trials are expected to yield valuable information for the sustenance of this cohort as well as elucidation of factors that may facilitate or impede recruitment and retention of volunteers for HIV vaccine trials from the PO cohort as well as from the general public if there will be a need to do so.

4.6.4 Ongoing or planed HIV vaccine and cohort studies in Dar es Salaam

At the Dar es Salaam site the on-going HIVIS-03 (or WP-1 of TaMoVaC-01) study is now administering 2nd dose of MVA/placebo. This is a natural transition to the present study drawing on the existing resources. It is also envisaged that additional staff will be recruited as the need arise. The site will also be involved in cohort and cross sectional studies along the lines of HIV vaccine trials. These are expected to be supported through a grant application to Sida/SAREC. Recently an additional grant has been approved by the EDCTP for continued HIV vaccine studies of the HIVIS DNA and MVA-CMDR in Tanzania and Mozambique.

4.7 Preliminary work on HIV vaccine trials and HIV cohorts in Mbeya

MMRP had been involved in past HIV vaccine and cohort trials and has established an experienced study clinic, community outreach team, pharmacy and laboratories. The MMRP labs include a clinical laboratory (hematology, clinical chemistry, serology, CD4 count analysis, HIV testing and HIV RNA) as well as an immunological lab which has established FACScanto flow cytometry, ICS and ELISPOT assays throughout various studies. Both labs have been accredited by the College of American Pathologists (CAP) and participate in external QA/QC programs with UKNequas and CAP and follow therefore the highest standard of quality control and validation which is audited and monitored yearly by independent consultants. The MMRP labs have been recognized as one of the national reference laboratories. All lab staff members are GCLP trained and repeat training is performed every other year.

4.7.1 HIV cohorts

At MMRP three cohorts have been or are currently under observation, with a fourth cohort study being in the initiation process:

HISIS is a high-risk cohort of bar workers that has a cumulative HIV incidence of 7% over 3 years (14% first year, 5% and 3% second and third year, respectively). It has maintained in this difficult environment a high follow up rate of above 70% after 4 years [10, 39-41].

CODE is a general population cohort with 3000 individuals in Mbeya town and surrounding rural areas. Here, specific subgroups, such as young adults between the age of 18 and 25 have been identified to have the highest risk of HIV acquisition, with an HIV incidence of 3.2% and 2.3% for women and men.

The **EMINI** cohort consisting of 18,000 individuals has been established in 9 geographic distinct areas of Mbeya in preparation for large-scale phase III and IV trials.

RV217 is a study funded by USMHRP and is currently in the process of being initiated. In this cohort bar workers will be recruited to assess the HIV incidence and retention rate of the risk group to be included in HIV vaccine trials.

These cohorts thus form a solid base for the recruitment of volunteers for the proposed phase II trial and for future phase IIB/III cohorts. The trial will also strengthen the community activities, the clinical trial organization and ensure that laboratory capacity is strengthened and continuously kept up to highest international standard.

4.7.2 Phase I/II HIV vaccine trial in Mbeya

MMRP participated in the multi-country (Tanzania, Kenya, Uganda) RV172: A Phase I/II Clinical Trial to Evaluate the Safety and Immunogenicity of a multiclade HIV-1 DNA Plasmid Vaccine, VRC-HIVDNA016-00-VP, Boosted by a multiclade HIV-1 Recombinant ADENOVIRUS-5 Vector Vaccine, VRC-HIVADV014-00-VP, in HIV uninfected adult volunteers in East Africa. The study was sponsored by DAIDS/NIH and conducted by the U.S. Military HIV Research Program (USMHRP). Enrollment for RV 172 began in May 2006

and ended in late October 2006. Overall 324 were enrolled in the study. In Mbeya 491 volunteers from the general population were briefed, 279 were screened and 60 participants were finally enrolled in the study. The study was conducted throughout 14 consecutive visits including 4 vaccination visits (3 DNA prime, 1 Ad5 boost vaccination). All vaccinations were completed at all RV 172 sites in April 2007 and all follow-up visits were completed in October 2007. No safety concerns for acute reactogenicity or adverse events have been recognized. The study has shown excellent immunogenicity for HIV antigens. Overall, 63% of vaccine volunteers receiving vaccine have shown a positive γ - IFN ELISpot response using cryopreserved cells. Response rates of up to 85% have been observed at sites employing fresh cells for this assay and the ICS assay [42-43]. During the study, one HIV infection was observed within the RV 172 volunteers, and 3 further HIV infections were detected after study completion.

4.7.3 Ongoing or planed HIV vaccine and cohort studies in Mbeya

Phase I HIV vaccine studies apart from the TaMoVac 1 study are planned in Mbeya including the HIV/DAIDS funded RV262 and the EDCTP AfreVac study. Both studies might start in 2010 and are currently in the process of protocol development. A larger HIV incidence and prevalence study (RV217) in an HIV high risk population is under ethical review and is expected to start in 2009. The RV172 vaccine trial shall be amended for safety and immunogenicity follow-up and is currently under ethical review to start in 2009. The site is capable and will be prepared to conduct these studies in parallel in respect to administrative, recruitment, outreach, clinical and laboratory activities. Mbeya is part of the approved EDCTP grant mentioned above.

4.8 Rationale for the proposed study objectives

In the HIVIS 01/02 trial 1mg DNA i.d. given at three occasions with the Biojector gave lower immune responses than 3.8mg i.m after the DNA priming alone while after a single MVA immunization the immune responses were indistinguishable. Although all but one volunteers responded with HIV specific immunity in this trial, the effect of the DNA delivery was suboptimal in a number of aspects. Immune responses clearly were dampened in volunteers older than 40 years of age. Half of the volunteers were given GMCSF (as a possible adjuvant), which was found to be associated with lower immune responses. The boost of MVA was given either as a low dose i.d. or a 10-fold higher dose i.m. (since the optimal dose was not known) and the i.d. dose was markedly inferior. In fact the only non-responder was a 57 year old vaccinia-vaccinated male primed with DNA i.m. + GMCSF and boosted with 10^7 pfu MVA i.d.

Preliminary data from the HIVIS 03 trial, which was designed with the above findings in mind, indicate better priming after DNA alone in Tanzania compared to Sweden. This may be related to the fact that trial volunteers are below 40 years of age in Tanzania.
In the current study we are in addition planning to give the MVA vaccine boost twice since we noted only moderate induction of vaccinia immunity following a single vaccination with MVA in the Swedish trial.

Since the initial immune response after DNA alone did not predict the immune response after boosting with MVA it is possible to investigate if the repeated priming with a lower dose of DNA with optimized background conditions can give the same net effect after 2 MVA boosts. This would mean that we could simplify the rather cumbersome method of 5 simultaneous i.d. immunizations and reduce them to 1-2 i.d. inoculations at the same event. This would make this form of administration feasible for larger studies and significantly reduce the cost of the vaccine. Furthermore such studies will be a stepping-stone into further trials with different adjuvants, or optimized delivery methods, i.e. electroporation via Derma Vax™. These methods will not only increase the immunogenicity of the DNA prime but also induce a substantial antibody response.

A unique feature of the HIVIS 01/02 and HIVIS 03 trials is the separation of the injection of the *gag* and *env* containing pools to different arms. This was based on a superiority of this modality to a combination of all plasmids in mouse models. It would however be much more practical if all plasmids could be combined as proposed in this study.

Furthermore the project is part of a larger programme funded by the EDCTP to improve North-South collaboration in HIV vaccine trials, improve the capacity for future HIV vaccine trials in MUHAS, MMRP and Tanzania in general, through better networking of the involved institutions and to provide information as a basis for future HIV vaccine trials in Tanzania and Mozambique.

5 STUDY OBJECTIVES

The objectives of the current proposal are as follows:

5.1 Primary objectives

1. Determine safety of the DNA vaccine developed at Karolinska Institutet/SMI (HIVIS-DNA) at a dose of 600 µg or 1000 µg delivered i.d in combination with MVA-CMDR boost i.m
2. Determine immunogenicity of HIVIS-DNA at a dose of 600 µg or 1000 µg delivered i.d in combination with MVA-CMDR boost i.m.

5.2 Secondary objectives

1. Compare immunogenicity of KI-DNA at a dose of 600 µg given as combined plasmid pools or separate plasmid pools i.d in combination with MVA-CMDR boost i.m.

2. To build expertise and capability in evaluating HIV-1 vaccine candidates in Tanzania.

6 METHODOLOGY

6.1 Study design

This is a randomized, controlled, double blinded study with regard to placebo/vaccine but not group.

6.2 Study setting

The clinical sites will be MNH/MUHAS in Dar es Salaam and Mbeya Medical Research Programme (MMRP) in Mbeya, TANZANIA.

Laboratory tests will be performed at:

1. the Department of Microbiology and Immunology, MUHAS, Dar es Salaam,
2. the main and immunological laboratories of the Mbeya Medical Research Programme, Mbeya
3. the Swedish Institute for Infectious Disease Control, Stockholm, SWEDEN

Additional studies may be performed at:

4. The Walter Reed Army Institute of Research (WRAIR), USA
5. The Imperial College (IC), London, UK

6.3 Duration of study

The recruitment and enrolment period is planned to be 12 months. The volunteers will be monitored through week 24 after the last injection ie a total of 70 weeks.

6.4 Study volunteers

Primarily, volunteers will be recruited in Dar es Salaam from the Police force with whom there has been extensive prior contact for the last 10 years. Additionally volunteers will also

be recruited from the Prison force. Volunteers from the general public including young adults attending the Infectious Disease Clinic (IDC) who would have been assessed by the clinical team regarding their availability for long-term follow up will also be invited.
In Mbeya healthy volunteers will be recruited from the Mbeya urban environment. MMRP has developed over the years throughout the past cohort studies a well-established relationship to the communities and community leaders of various areas in the Mbeya region.
The study will consist of 120 healthy volunteers, of whom no less than 30 will be females found to HIV negative in the screening process and be at low risk for HIV infection.
Differential recruitment will be accommodated in case a site is not able to recruit the 60 volunteers or is unable to recruit the 15 females in the specified time of enrolment

6.5 Study groups

The study will have six Groups of DNA injections as follows; All followed by MVA boosts.

**Group**	**Number of Volunteers**	**DNA immunization** weeks 0, 4 and 12	**MVA boost** weeks 30 and 46
IA	36	600 µg i.d (combined plasmid pools)	MVA, at 10^8 pfu i.m
IB	4	Saline 2 x 0.1ml i.d	Saline, im
IIA	36	600 µg i.d (separate plasmids pools)	MVA, at 10^8 pfu i.m
IIB	4	Saline 2 x 0.1 ml i.d	Saline, im
IIIA	36	1000 µg i.d (separate plasmids pools)	MVA, at 10^8 pfu i.m
IIIB	4	Saline 5 x 0.1 ml i.d	Saline, im

HIV-DNA/placebo will be given at weeks 0, 4 and 12 as i.d injections with the Biojector 2000 device in the skin over the deltoid muscles.

**Group**	**Left Arm**	**Right Arm**
**I**	1 injection id of 0.1 ml of 3mg/ml [300µg] Pool 1 and 2 (env/gag) combined	1 injection id of 0.1 ml 3mg/ml [300µg] Pool 1 and 2 (env/gag) combined
**II**	1 injection id of 0.1 ml 3mg/ml [300µg] Pool 1 (env)	1 injection id of 0.1 ml 3mg/ml [300µg] Pool 2 (gag)
**III**	3 injections id of 0.1 ml of 2mg/ml [600µg] Pool 1 (env)	2 injections id of 0.1 ml of 2mg/ml [400µg] Pool 2 (gag)

Two HIV-MVA/placebo boost i.m. injections will be given at 18 and 34 weeks after the last (third) DNA/placebo priming injection at 10^8 pfu in the deltoid muscle.

6.6 Randomization procedure

Block randomization in blocks of 10 with a ratio of 9 vaccine recipients to a single placebo will be used with equal distribution among the two sites.

6.7 Inclusion criteria

1. Age: 18 to 40 years
2. Willing to undergo counseling and HIV testing
3. Have a negative antigen/antibody ELISA for HIV infection
4. Able to give informed consent
5. Satisfactory completion of an assessment of understanding prior to enrolment defined as 89% correct answers after three opportunities to take test.
6. Basic abilities to read and write.
7. Resident in Dar es Salaam or Mbeya, and willing to remain so for the duration of the study
8. At low risk of HIV infection, defined as the absence of an identifiable risk factor/ behavior (Their presence is therefore an exclusion criteria):

- sexual partner with HIV

- sexual partner with unknown HIV serostatus who is also unwilling to use protective condoms consistently in all sexual relations
- sexual partner is known to be at high risk for HIV
- more than one sexual partner in the last 6 months
- history of being an alcoholic [as medically defined or more than 35 units /week]
- history of STI within past 6 months.

9. Verbal assurances that adequate birth control measures are used not to conceive/father a child during the study and up to 6 months after the last vaccine injection
10. Women shall have a negative urinary pregnancy test
11. Be willing to practice safe sex for the duration of the study to avoid sexually transmitted infections including HIV.
12. Good health as determined by medical history, physical examination, clinical judgment and by key laboratory parameters as judged by the study physician.

Reference ranges will be in accordance with data generated at MUHAS for the Dar es Salaam site, and that generated at Mbeya (MMRP) for the Mbeya site. Exclusion by presence of Diabetes mellitus will be based on the DAIDS Table for Lab Criteria cut-off value of a Fasting Blood Glucose ≥6.11 mmol/L if random Blood Glucose is ≥6.44 mmol/L (Grade 1 Toxicity). Local reference ranges will be use if the DAIDS Table for Lab Criteria refers to upper limit of normal (ULN), e.g. liver enzymes, creatinine, bilirubin) or lower limit of normal, e.g. albumin (LLN). For hematology and urine analysis results of the DAIDS toxicity table criteria will be applicable.

- *Hb >10.5 g/dl*
- *White blood cell count >13,300/mm^3*
- *Neutrophils >1,200/mm^3*
- *Lymphocytes >1.0/ mm^3*
- *Platelets >120,000/ mm^3*
- *Random Blood Glucose <6.44 mmol/L; if elevated, then a Fasting Blood Glucose <6.11 mmol/l*
- *Bilirubin <1.25 x uln (DAIDS <1.1)*
- *ALT <1.25 x uln*
- *Creatinine <1.25 x uln (DAIDS <1.1)*
- *Urine dipstick for protein and blood: negative or trace. (If either is ≥ 1+, obtain complete urinalysis (UA). If microscopic UA confirms evidence of hematuria or if proteinuria ≥ 1+ (with a protein/creatinine ratio < 45mg/mmol), the volunteer is ineligible).*

6.8 Exclusion criteria

The following are the criteria for exclusion from the study:

1. At risk of HIV infection as mentioned above in the inclusion criteria
2. Active tuberculosis or other systemic infectious process elicited by review of systems, physical examination and laboratory detection (for example detection of Hepatitis B surface antigen, or active syphilis).
3. A history of immunodeficiency, chronic illness requiring continuous or frequent medical intervention
4. Autoimmune disease by history and physical examination.
5. Hives or recurrent hives and severe eczema
6. A history of psychiatric, medical (including traditional medicine) and/or substance abuse problems during the past 6 months that the investigator believes would adversely affect the volunteer's ability to participate in the trial.
7. History of grand-mal epilepsy, or currently taking anti-epileptics
8. Received blood or blood products or immunoglobulins in the past 3 months.
9. Receiving immunosuppressive therapy such as systemic corticosteroids or cancer chemotherapy.

10. Use of experimental therapeutic agents within 30 days of study entry.
11. Reception of any live, attenuated vaccine within 60 days of study entry. Medically indicated subunit or killed vaccines (e.g., Hepatitis A or Hepatitis B) are not exclusionary but should be given at least 2 weeks before or after HIV immunization to avoid potential confusion of adverse reactions.
12. Abnormality in ECG that could indicate risk or make interpretation of vaccine effects difficult according to the study operating procedures.
13. Previously received an HIV candidate vaccine.
14. History of severe local or general reaction to vaccination defined as:
    - **Local**: Extensive, indurated redness and swelling involving most of the major circumference of the arm, not resolving within 72 hours
    - **General**: Fever >= 39.5 ^{0}C within 48 hours; anaphylaxis; bronchospasm; laryngeal edema; collapse; convulsions or encephalopathy within 72 hours
15. Lactating mother
16. Study site employees who are involved in the protocol and may have direct access to the immunogenicity results
17. Unlikely to comply with protocol as judged by the principal investigator or his designate.

7 RECRUITMENT PROCEDURE

7.1 Recruitment in Dar es Salaam

As aforementioned, volunteers will be recruited from cohort of Police Officers' (PO's) volunteers in the on-going HIV incidence and socio-behavioral studies in Dar es Salaam. The established "core group" of about 400 PO's will be used to assist with educational activities on HIV/AIDS and HIV vaccine studies in the force as well as recruitment of the prospective 60 volunteers. The same procedures will be used that have proven to avoid coercion and social discrimination in two incidence studies in this group of policemen. Meetings at the Police stations, efforts by the "core group" of collaborators and a one to one contact, are the main modalities to reach potential volunteers. Information and briefing sessions containing general information through power point presentations about the study provide information sheets as well as the opportunity for individual questions and counseling will be offered. Volunteers are planned to be recruited from other populations (Prison Officers, general population including young adults) and this will be deemed especially necessary with recruitment of the required 15 female volunteers. Experie nces from Mbeya will be shared for Dar es Salaam. The study site for the successive visits and HIV vaccinations will be at the building that houses the current HIVIS project clinic in Dar es Salaam.

7.2 Recruitment in Mbeya

Volunteers will be recruited from the Mbeya urban environment through advertisement using poster, newspaper advertisement and flyers. MMRP has developed over the years throughout the past cohort studies a well-established relationship to the communities. Community leaders and community advisory boards of various areas in the Mbeya region will help in the advertising and recruiting process. Recruitment of the required number of female participants is not considered to be a problem in Mbeya as most study participants in past studies were females (e.g. 60% in RV172). Interested volunteers will be invited to an information and briefing session. These sessions will contain general information through power point presentations about the study. Information sheets as well as the opportunity for individual questions and counseling will be provided. There will also be time for questions on an individual basis without ties to the particular group involved. Interested volunteers will receive an appointment for the first screening visit or contact details for later screening assignment. Information sessions will also be provided for the community leaders and the community advisory board. The briefing and information sessions will take place at the MMRP vaccine trial location in Mbeya.

8 STUDY PROCEDURES

An overview on study procedures are provided in the Study Timetable ***(Appendix 1)***, in the Visit Schedule and Forms Flow ***(Appendix 2)*** and in the Lab Schedule Flow ***(Appendix 3)***.

8.1 Screening I (Study Visit 1, 4-8 weeks before enrolment)

8.1.1 Informed consent procedures and study information

The first screening visit (study visit 1) will take place between 4 and 8 weeks before the first planned injection. At this visit the study volunteer will meet the study doctor(s) and study nurse(s), and the trial will be discussed in detail associated with thorough informed consent procedures (***see Appendix 4 A or B***). This discussion will also address on the following: the meaning of 'randomization', potential adverse effects of the vaccine, and about the fact that it is unknown whether or not the study vaccines will protect or even increase the risk for him/her to acquire HIV infection. They will be informed that following immunization they may develop antibodies that will produce a positive reaction in a routine HIV test, but that provisions have been made to distinguish between a post vaccination response and HIV infection during and after the trial. This includes that volunteer will be excluded from donating blood or organs and that they may be vulnerable to social risk if they develop HIV antibodies, or by revealing their participation in the study. They will be assured of the level of care that will be made available to them should they be found to be HIV infected at any time during their participation in the study, including the screening period (see also section 17.1). HIV infections occurring after the study and observation period will be handled in the regular established HIV clinic in the police force or community as applicable. The placebo concept will also be explained. Study Participants who have received and signed the initial informed consent (Version 2.0, September 2009) will receive written information regarding the changed visit schedule. Signed and dated informed consent will be requested (***see Appendix 4 C and D***).

Volunteers will be counseled by study personnel about safer sex. They will be counseled on the importance of contraception from starting immunization up to 3 months after the last immunization and that they should continue to use condoms with sexual partners whose HIV status is not known. They will also be reminded about these items on each day of immunization. High quality hypoallergenic condoms will be provided free of charge to volunteers throughout the trial.

Upon this information about the trial and the informed consent procedures, consenting volunteers will be requested to successfully pass a test of understanding ***(Appendix 5, CRF 1-I)*** with at least 90% of correct answers with the possibility of maximum 3 repeated attempts.

Demographic data and participant's contact information will be collected and recorded (CRF 5-I).

8.1.2 HIV counseling and testing

Study personnel will assess volunteers for past and current risk of HIV infection using the Risk Assessment Questionnaire ***(Appendix 6, CRF 1-II)***, followed by counseling for those who will consent to undergo HIV test prior to collecting blood. The counseling process will ensure that volunteers have sufficient knowledge about HIV infection to understand what the test is for, the implications of a positive, negative and equivocal result and the standard of care available for HIV infection locally. They will also be informed on how and when they will receive the result.

8.1.3 Clinical history and examination

The clinical contact will be documented by using the Screening Visit Contact Form ***(CRF 2-I)*** and Screening I Physical Examination Form ***(CRF 3-I)***. The clinical contact will include a complete physical examination and medical history, including any ongoing medication and

any previous allergic reactions, any previous reaction to vaccination, history of epileptic fit, exposure to smallpox vaccine, and smoking practices. The general examination will include weight (kg), height (cm), mouth and throat inspection, palpation of lymph nodes (cervical, clavicles, and axillaries), blood pressure, and inspection of the skin to exclude severe eczema and check for the presence of a vaccinia vaccination scar, respiratory, cardiovascular, genital urinary, central nervous system and gastrointestinal system examination. A genital infection screening will be performed if indicated on the account of risk and symptoms according to trial specific clinical standardized operating procedures: urethral, vaginal, cervical specimens, as appropriate for common sexually transmitted infections. The medical history should ensure good general health and the absence of any chronic medical condition that could influence the safety evaluation. Relevant past and current medical conditions during the entire screening period will be documented on the Previous Condition Form ***(CRF 4-II)*** and current or ongoing medication documented on the Concomitant Medication Form ***(CRF 4-I)***.

8.1.4 Safety laboratory tests and HIV testing

All so far eligible (with among other things, at least 1 negative HIV Antibody test result within previous 3 months) volunteers will have their blood sample screened for HIV. HIV antigen and antibody ELISA will be used for initial testing. Non-reactive samples on the first ELISA will be regarded as negative. All reactive samples will be tested in duplicate on a second ELISA. Discrepant results between two ELISA's will be resolved by Western Blot and HIV RNA PCR methods (***see Appendix 7***). HIV results throughout the study will be reported to the clinic from the laboratory as infected or not infected without providing the test methods as false positive HIV ELISA or rapid test result could indicate a participants randomization to the vaccine Group ***(CRF 9-I)***.

Safety laboratory test will include the status of the liver (serum alanine aminotransferase and bilirubin), renal function (serum creatinine) and random blood glucose that will be followed by fasting blood sugar during screening visit 2 for individuals whose tests will be suspicious of diabetes mellitus ***(CRF 9-V)***. Other tests will include a complete blood count with 5 part differentials ***(CRF 9-IV)*** and screening for syphilis, hepatitis B (surface antigen) infection ***(CRF 9-VI)*** as well as urine dipsticks ***(CRF 9- VII)***. All women will have a urine pregnancy test done ***(CRF 9-II)***.

8.1.5 Blood and urine collection

Blood will be collected using a sterile needle, usually from the ante-cubital fossa, according to the SOP's and the visit schedule and transported to the appropriate laboratories. Urine will be collected into a sterile container and tested by a member of the clinical team according to trial specific SOP's. Urine dipsticks and pregnancy test will be performed at the clinic. If urine dipsticks indicate abnormal results (≥ *1+)* urine will be transferred to the labs for microscopic examination and if indicated for culture and sensitivity. The volunteer will as well be reviewed to rule out other systemic conditions that can result into abnormal urine results such as proteinuria. In cases with confirmed proteinuria of unclear etiology a quantitative/qualitative urine protein investigation can be ordered at the referral laboratories in Dar es Salaam. Determination of Protein/Creatinine Ratio by comparing the concentration of protein in the urine to the creatinine_level in a spot urine sample may be performed and in so doing a Protein:creatinine ratio >45 mg/mmol would be considered to indicate presence of proteinuria. It should be noted that the provision of expedited cold chain transport of samples for not available lab investigations from Mbeya to Dar es Salaam is possible.

All samples for routine hematology, clinical chemistry and other investigations will be labeled appropriately with the full study volunteer identity. Samples that will be analyzed after freezing shall be prepared, labeled, and stored at the appropriate temperature. Specimens that are shipped will be correctly prepared, labeled, and kept at the correct temperature.

The total estimated volume of blood to be collected at different time points is shown on the investigations flow chart for each scheduled visit ***(see Appendix 3)***. All biological samples to

be analyzed by the research laboratory will be collected and processed according to institutional guidelines and regulations regarding biobanks. All samples sent to the appropriate laboratories at MUHAS and MMRP will be labeled appropriately.

8.1.6 Labeling procedures

Scheduled study visits will be numbered 01, 02, etc. with the addition A-I for post vaccination visits (e.g. 03A, 03B etc.) and J-Z for unscheduled visit (e.g. 03J, 03K etc.). Site and participant code number will contain 4 digitals indicating the first digital 3 for Dar es Salaam and 2 for Mbeya followed by 3 digitals for participant identification (e.g. Dar: No. 3001, Mbeya: No. 2001). Laboratory samples labels will contain the study visit number, the site and participant code, the date and time of collection, the type of sample (serum, plasma, blood etc.) and a unique sample identification number assigned by the site laboratory.

8.2 Screening II (Study Visit 2, 2-4 weeks before enrolment)

The second screening visit (study visit 2) takes place two to four weeks before the first planned injection. This visit will include review of informed consent, inclusion criteria, medical conditions and laboratory results. Reassurance has to be made that the study volunteer fully understands the concept of the study. Volunteers will also be reminded that they are free to end all obligations in the study whenever they wish and that a premature termination will not have any negative influence in future contacts with the clinic. Available HIV results will be discussed during post-test counseling by the nursing staff, consent process reviewed and volunteers probed for questions/concerns before referral to the medical officer. The medical officer will review the informed consent process, questions/answers, review of health and concomitant medications since last visit with the volunteer as well as check inclusion/exclusion and eligibility criteria ***(CRF 2-I, CRF 3-II)***. An ECG will be performed for baseline assessment **(CRF9-III)** if not done at Visit 1. A competent study physician will do the interpretation of ECG results and furthermore all ECG tracings will be sent as a PDF for interpretation/approval to a cardiologist panel. If a volunteer is found to be not eligible, a Screening Outcome Form ***(CRF 5-IV)*** will be filled indicating reasons for ineligibility. In the case of HIV positive test results or other medical conditions leading to ineligibility volunteers will receive medical advice and/or indication concerning the referral to appropriate health facilities outside the study premises. Minor or curable medical conditions identified during the first or current screening visit which do not necessarily lead to ineligibility are subject for care and treatment provided by the study site. This might include the control of selected lab results or medical conditions as indicated by the investigator. Additional screening visits to follow-up on such medical conditions might be necessary. If more than 3 months have elapsed since Visit 1 baseline, lab examinations (CBC and chemistry) as well as a medical status examination will have to be repeated. The latest visit should be 2-4 weeks before study visit 3, with an HIV serology no older than 8 weeks. Volunteers found to be eligible throughout the screening process will be asked to provide blood for baseline immunology tests as indicated in the Lab Schedule Flow and will then receive an appointment for the enrolment and first vaccination at the study visit 3.

8.3 Enrolment and Vaccination I (1st DNA/Placebo) (Study Visit 3, week 0)

The study volunteer again meets both study nurse(s) and study doctor(s) ***(CRF 2-II)***. Confirmation has to be made and recorded that the volunteer fulfils all inclusion/exclusion criteria including laboratory results ***(CRF 5-II)*** and eligibility criteria ***(CRF 5-III)***. If a volunteer is not found to be eligible a Screening Outcome Form ***(CRF 5-IV)*** will be filled indicating reasons for ineligibility. Enough time has to be planned for the visit so that all questions from the volunteer about the study can be answered or reflected upon. A senior Physician will confirm if the volunteer understands the concepts of the study and will also assess the willingness and voluntariness the study volunteer. As indicated during the informed consent procedure the volunteer had consented on a study photo identification card produced and provided by the site. This photo ID will be kept by the study participant and will have to be

presented at each study clinic contact for identification purposes, unless other unequivocal identification can be documented.

For enrolment the initial screening number will be used as the study number. This means that study numbers may be discontinuous. The participant will then be randomized into one of the study Groups.

8.3.1 Randomization procedure

Sixty plain envelopes numbered from 1 to 60 for each site are provided by the Swedish Institute for Infectious Disease Control which is responsible for the vaccine/placebo product supply. They will be divided in 6 sets of 10 envelopes. Each set will contain 3 assignments to Group IA, 3 to Group IIA and 3 to Group IIIA and 1 to either of Group IB, IIB or IIIB. Group numbers refer to the immunization schedule. The study Pharmacist will take the prepared envelopes and put them in a box that is kept under lock. The pharmacist will randomize participants accepted into the study by selecting the next consecutive envelope containing the vaccine or placebo assignment in accordance with the generated randomization list. This will be documented in ***CRF 8-I*** pharmacy copy. The Pharmacist will then inform the Study Nurse/medical officer at the clinic of the randomization group of the volunteer (2x versus 5x i.d. DNA priming) on the ***CRF 8-II*** clinical copy, including the number of the envelope, but will not inform on whether this is vaccine or placebo. The Study Nurse will record the envelope number and the group assignment (2x versus 5x i.d. DNA priming) into a register book, the medical record file (source document), as well as the outer cover of the participant's file.

8.3.2 Blinding procedure

The vaccines will be kept in the department of Microbiology/Immunology at MUHAS or at the study pharmacy at NIMR-MMRP under appropriate storage conditions according to manufacturer's specifications. The study pharmacist who has access to the code will draw the study vaccine and the placebo into syringes and label them with the study number, the site, randomization group of the volunteer (2x versus 5x) and participant code, date and time and hand them over to the study nurse for injection. Nurses administering the vaccines, all other site investigators and lab personnel as well as the participants themselves will be blinded to the type of vaccine that the participant will be receiving.

8.3.3 Vaccination

Prior to the first vaccination the enrolled study participant will receive an abbreviated risk assessment and risk behavior counseling as well as pre HIV test counseling prior to HIV testing. Vital signs will be recorded and blood will be donated according to the visit schedule (safety laboratory tests, urine dip stick, HIV test, CD4 count and baseline immunogenicity tests) including pre vaccination pregnancy test from urine for female participants. The participants will then attend the medical officer who will evaluate the pre vaccination medical status ***(CRF 3-II)***.
A pregnancy test for female participants before each vaccination will have to show negative results *before* vaccination.
After confirming the medical eligibility for vaccine administration including a negative pregnancy test for female participants the vaccine request form ***(CRF 10-II)*** will be filled by the medical officer, transferred to the pharmacist who will then deliver to the nurse the appropriate vaccine/placebo that corresponds to the assigned group. The participant will then receive the first injection. After vaccination the participants will be observed for 30 minutes at the clinic for potential early adverse reactions (anaphylactic reactions). Follow-up and safety procedures as well as distribution and handling of diary cards will be performed. A detailed description of the post vaccination procedures is provided in the protocol section ***Immunization*** further below.

The participant will then be compensated and assigned to his next scheduled visit.

8.4 Study visit 4 – Follow up
(Study week 2; 14 days after V3 +/- 3 days)

This is the follow up visit two weeks post 1st DNA/Placebo vaccination. It is essentially a safety assessment visit conducted by both study nurse(s) and study doctor(s) ***(CRF 2-III)***. At this visit diary cards will be collected by study nurses and discussed with the participants addressing also problems and deficiencies arrived within in recording process. Assistance and explanation will be again provided and sufficient time given for questions and answers. HIV posttest counseling and risk behavior counseling will be performed as well as recording of vital signs. Blood will be donated according to the flow chart. The participant will then attend the medical officer who will revise the diary card and discuss potential adverse reactions documented. Diary entries will be transcribed into the vaccine reaction form ***(CRF 10-1)***. The medical status will be evaluate and documented ***(CRF 3-II)***, new or changed medication ***(CRF 4-I)*** as well as medical events ***(CRF 6-I)*** since the last visit will be recorded. All medical conditions (symptoms and/or diagnosis) that are not defined and indicated in the vaccine reaction form ***(CRF 10-1)*** will be recorded in the adverse events form ***(CRF 6-I)*** including the severity grade and the relationship to the study agent. The participant will then be compensated and assigned to his next scheduled visit.

8.5 Study visit 5 – Vaccination II (2nd DNA/Placebo)
(Study week 4; 28 days after V3 +/- 3 days)

The visit is for the second DNA/Placebo injection of the study vaccine and is conducted by both the study nurse and the study doctor ***(CRF 2-III)***. An abbreviated risk assessment and risk behavior counseling as well as pre HIV test counseling will be performed, vital signs will be recorded and blood will be donated according to the visits schedule including pre vaccination pregnancy test for female participants. The vaccination procedures including pre- and post vaccination assessments will be conducted as described above.

8.6 Study visit 6 – Follow up
(Study week 6; 14 days after V5 +/- 3 days)

This is the first follow up visit two weeks post 2nd DNA/Placebo vaccination. It is essentially a safety assessment visit and will be conducted as described for study visit 4. Post HIV test counseling will be performed, diary cards discussed, blood drawn according to the flow chart and participants will be seen by the medical officer for medical status recording and diary transcription. The forms will be filled according to the visit schedule checklist. The participant will then be compensated and assigned to his next scheduled visit.

8.7 Study visit 7 – Follow up
(Study week 8; 28 days after V5 +/- 5 days)

This is the second follow up visit post 2nd DNA/Placebo vaccination. It is essentially a safety assessment visit and will be conducted by the study nurse(s) and the study doctor. Abbreviated risk assessment and risk behavior counseling will be done, vital signs performed, blood drawn according to the flow chart and participants will be seen by the medical officer for medical status recording. CRF's are filled according to flow chart. The participant will then be compensated and assigned to his next scheduled visit.

8.8 Study visit 8 – Vaccination III (3rd DNA/Placebo)
(Study week 12, 84 days post V3 +/- 7days)

This visit is for the 3rd DNA/Placebo vaccination and is conducted by both the study nurse and the study doctor and will be conducted as described above.

8.9 Study visit 9 – Follow up
(Study week 14; 14 days after V8 +/- 3 days)

This is the first follow up visit two weeks post 3rd DNA/Placebo vaccination. It is essentially a safety assessment visit and will be conducted as described for study visit 4. Blood draw and

filling of forms will be done according to the visit schedule. The participant will then be compensated and assigned to his next scheduled visit.

8.10 Study visit 10 – Follow up
(Study week 16; 28 days after V8 +/- 5 days)
This is the second follow up visit post 3rd DNA/Placebo vaccination. It is essentially a safety assessment visit and will be conducted as described for study visit 7. Blood draw and filling of forms will be done according to the visit schedule. The participant will then be compensated and assigned to his next scheduled visit.

8.11 Study visit 11 – Vaccination IV (1st MVA/Placebo)
(Study week 30; 126 days post V8 +14/-28 days)
The visit is for the first MVA/Placebo injection of the study vaccine conducted by both the study nurse and the study doctor and will be conducted as described above. The participant will then be compensated and assigned to his next scheduled visit.

8.12 Study visit 12 – Follow up
(Study week 32; 14 days after V11 +/- 3 days)
This is the first follow up visit two weeks post 1st MVA/Placebo vaccination. It is essentially a safety assessment visit and will be conducted as described for study visit 4. Blood draw and filling of forms will be done according to the visit schedule. In addition a post vaccination ECG will be performed and results will be interpreted and compared to baseline ECG results by competent study physicians and send to the cardiologist panel for review and approval. Post vaccination ECG will be graded as ***normal***, ***abnormal/clinically insignificant*** and ***abnormal/clinically significant***. Post vaccination ECG findings will be graded as ***no changes, changes/not significant*** and ***changes/significant*** as compared to baseline results. Clinical chemistry analysis will include a Troponin I test. The participant will then be compensated and assigned to his next scheduled visit.

8.13 Study visit 13 – Follow up
(Study week 34; 28 days after V11 +/- 5 days)
This is the second follow up visit post 1st MVA/Placebo vaccination. It is essentially a safety assessment visit and will be conducted as described for study visit 7. Blood draw and filling of forms will be done according to the visit schedule. The participant will then be compensated and assigned to his next scheduled visit.

8.14 Study visit 14 – Vaccination V (2nd MVA/Placebo)
(Study week 46; 168 days post V11 +14/-28 days)
The visit is for the second MVA/Placebo injection of the study vaccine conducted by both the study nurse and the study doctor and will be conducted as described for Visit 11. The participant will then be compensated and assigned to his next scheduled visit.

8.15 **Study visit 15 – Follow up**
(Study week 48; 14 days after V14 +/- 3 days)
This is the first follow up visit two weeks post 2nd MVA/Placebo vaccination. It is essentially a safety assessment visit and will be conducted as described for study visit 4. Blood draw (including Troponin I test when available) and filling of forms will be done according to the visit schedule. In addition a post vaccination ECG will be performed and results will be interpreted and compared to previous ECG as described for Study Visit 12. The participant will then be compensated and assigned to his next scheduled visit.

8.16 Study visit 16 – Follow up
(Study week 50; 28 days after V14 +/- 5 days)
This is the second follow up visit post 2nd MVA/Placebo vaccination. It is essentially a safety assessment visit and will be conducted as described for study visit 7. Blood draw and filling

of forms will be done according to the visit schedule. The participant will then be compensated and assigned to his next scheduled visit.

8.17 Study visit 17 – Follow up
(Study week 58; 84 days post V14 +/- 14 days)

This is the first long time follow-up visit after the completed vaccination schedule. It is essentially a safety and immunogenicity assessment visit. It will be conducted by the study nurse(s) and the study doctor. A risk assessment and risk behavior counseling as well as pre HIV test counseling will be performed. Vital signs will be recorded, blood will be drawn according to the flow chart and participants will be seen by the medical officer for medical status recording. CRF's are filled according to flow chart. The participant will then be compensated and assigned to his next scheduled visit.

Post HIV test counseling will be performed 2-3 weeks after this visit only by the nursing/counseling staff in the case of negative test results. This post HIV test contact will be recorded as an additional study visit (Visit 17J) and compensated as indicated for unscheduled visits.

8.18 Study visit 18 – Final study visit
(Study week 70; 168 days post V14 +/- 14 days)

This is the second long time follow up visit after the completed vaccination schedule and the final study visit. It is essentially a safety and immunogenicity assessment visit. The study nurse(s) and the study doctor will conduct it. Pre HIV test counseling and extended risk assessment and risk behavior counseling is performed. Vital signs will be recorded and blood will be drawn according to the flow chart. The medical officer will conduct a medical status examination and forms will be filled according to the visit schedule including the disclosure of the Patients Status Form ***(CRF 7-I)***, the Concomitant Medication Form ***(CRF 4-I)*** and the Adverse Events Form ***(CRF 6-I)***. Ongoing or new medical events if not related to the study agents will be reported and might be followed up in 1 or 2 additional unscheduled visits but should preferentially be referred to the appropriate health facilities for further management. In the case of ongoing pregnancies study visits will proceed as unscheduled visits until pregnancy and infant outcome can be recorded. Volunteers may be asked to participate in long-term follow-up studies. All study volunteers will be issued a card signed by the principal investigators (full names, tel. numbers and addresses) stating that the holder has participated as a volunteer in a HIV-1 immunization study and that the outcome of standard HIV diagnostic tests do not signify HIV-1 infection. Study participants will be invited to perform HIV testing if wished by the study site also after the completion of the study.
Post HIV test counseling will be performed 2-3 weeks after this visit only by the nursing/counseling staff in the case of negative test results. This post HIV test contact will be recorded as an additional study visit (Visit 18J) and compensated as indicated for unscheduled visits.

8.19 Additional visits

If the volunteer attends or contacts the clinic for any health related changes or event outside the regular study visit schedule the Visit Contact Form ***(CRF 2-III)*** will indicate this contact as an unscheduled visit. This might include also hospital visits, home visits or the documentation of contacts by phone. A Physical Examination Form ***(CRF 3-II)*** might be filled as well as the Adverse Event Form ***(CRF 6-I)*** and Concomitant Medication Form ***(CRF 4-I)*** if indicated. Unscheduled visits will be numbered based on the previous scheduled visits plus a capital letters ranging from J-Z indicating the sequence of the unscheduled visit (e.g. visit 10I, 10J, etc.).

8.20 Compensation for study participation

Regular compensations will be made to all volunteers/participants to cover their travel expenses as well as time and inconvenience compensation. For scheduled visits this will

amount to 20,000 TShs (~ 20 USD) for Dar es Salaam and 15,000 TShs (~ 15 USD) for Mbeya, where transport and living costs are lower compared to Dar es Salaam. Unscheduled visits will be compensated with 10,000 TShs in Dar es Salaam and 8,000 TShs in Mbeya. Compensation for unscheduled visits might be rejected in the case of assumed abuse of compensation, which will be up to the study team's decision. During the study volunteers/participants will receive hypoallergenic latex condoms free of charge. If any medication is required as a result of the study, this will also be provided free of charge. Such medication will include painkillers, antipyretics and the like. Treatment and care for minor diseases can be provided by the study and might include painkillers, antipyretics, antibiotics or anti malaria treatment, and iron substitution. In case of hospitalization/or if further care will be required, these will be covered under the medical insurance provided by the National Insurance Corporation of Tanzania Limited; these will be confined to the period of the study.

8.21 Pregnancy reporting and follow up

For volunteers who become pregnant before completing the vaccine series, no further immunizations will be given. Any discontinuation of the vaccine schedule will be recorded in the Subject Status Form ***(CRF 7-I)***. A positive UPT test result will be communicated to the volunteer as well as her ineligibility to continue with the vaccination schedule. The volunteer will be followed for all the remaining scheduled visits according to the schedule of procedures for safety evaluation, as well as the maternal outcomes during pregnancy, labor, delivery and post-delivery periods. The infant outcome will also be documented. A Pregnancy Report Form ***(CRF 11-I)***, which included also information on the maternal and infant outcome will be filled at various time points. According to the study protocol female participants will be required to use an effective contraceptive method until 6 months after the last vaccination, which is the last study visit (Visit 18). Pregnancy test will be performed according to the study flow schedule.

8.22 In the event of study discontinuation

Study volunteer/participants may withdraw his/her consent to participate in the study at any time without prejudice. Wherever possible, the tests and evaluations listed for the termination visit will be carried out if the volunteer/participant refuses follow-up according to the protocol visit schedule. The study monitor and Data and Safety Monitoring Board (DSMB) will be notified of all study withdrawals within 2 working days for enrolled participants.
Volunteers/participants who withdraw from the study will be replaced only as long as the study is still open for enrolment. No replacement will be allowed when the enrolment is closed. All participants who receive at least one immunization will be included in the safety analysis. If a participant does not complete the immunization schedule secondary to a serious adverse event or toxicity, he or she will continue to be followed according to the protocol visit schedule, *and,* at a minimum, until the adverse event/toxicity is resolved and/or the cause is identified.

A genuine effort will be made to determine the reason(s) why a volunteer fails to return for the necessary visits. This information will be recorded on the Subject Status Form ***(CRF 7-I)***.

9 IMMUNIZATION

9.1 Immunization schedule

While DNA/Placebo immunizations will be performed at weeks 0, 4 and 12, MVA/Placebo will be given at weeks 30 and 46. The immunization schedule is summarized below:

Study week		0	4	12	30	46
**Injection schedule in days +/- acceptable time frame**		0	28 days (+/-3) post DNA I	84 days (+/- 7) post DNA I	126 days post V8 +14 /-28 days	112 days post V11 +14 /-28 days
**Study Group**	**N**	**1st vaccination**	**2nd vaccination**	**3rd vaccination**	**4th vaccination**	**5th vaccination**
**IA**	36	**DNA I**<br>2 x 0.1ml i.d (combined plasmid pools in left and right arm) total 600µg	**DNA II**<br>2 x 0.1ml i.d (combined plasmid pools in left and right arm) total 600µg	**DNA III**<br>2 x 0.1ml i.d (combined plasmid pools in left and right arm) total 600µg	**MVA I**<br>1x 1ml of 10^8 pfu MVA given i.m.	**MVA II**<br>1x 1ml of 10^8 pfu MVA given i.m.
**IB**	4	**DNA placebo I**<br>2 x 0.1 ml of saline given i.d.	**DNA placebo II**<br>2 x 0.1 ml of saline given i.d.	**DNA placebo III**<br>2 x 0.1 ml of saline given i.d.	**MVA placebo I**<br>1x 1ml of saline given i.m.	**MVA placebo II**<br>1x 1ml of saline given i.m.
**IIA**	36	**DNA I**<br>2 x 0.1 ml i.d (1 pool 1 (env) in left arm, 1 pool 2 (gag) in right arm) total 600µg	**DNA II**<br>2 x 0.1ml i.d (1 pool 1 (env) in left arm, 1 pool 2 (gag) in right arm) total 600µg	**DNA III**<br>2 x 0.1ml i.d (1 pool 1 (env) in left arm, 1 pool 2 (gag) in right arm) total 600µg	**MVA I**<br>1x 1ml of 10^8 pfu MVA given i.m.	**MVA II**<br>1x 1ml of 10^8 pfu given i.m.
**IIB**	4	**DNA placebo I**<br>2 x 0.1ml saline given i.d.	**DNA placebo II**<br>2 x 0.1ml saline given i.d.	**DNA placebo III**<br>2 x 0.1ml saline given i.d.	**MVA placebo I**<br>1x 1ml of saline given i.m	**MVA placebo II**<br>1x 1ml of saline given i.m
**IIIA**	36	**DNA I**<br>5 x 0.1ml i.d (3 pool 1 (env) in left arm,2 pool 2 (gag) in right arm) total 1000µg	**DNA II**<br>5 x 0.1ml i.d (3 pool 1 (env) in left arm, 2 pool 2 (gag) in right arm) total 1000µg	**DNA III**<br>5 x 0.1ml i.d (3 pool 1 (env) in left arm, 2 pool 2 (gag) in right arm) total 1000µg	**MVA I**<br>1x 1ml of 10^8 pfu MVA given i.m.	**MVA II**<br>1x 1ml of 10^8 pfu MVA given i.m.
**IIIB**	4	**DNA placebo I**<br>5 x 0.1 ml saline given i.d.	**DNA placebo II**<br>5 x 0.1 ml saline given i.d.	**DNA placebo III**<br>5 x 0.1 ml saline given i.d.	**MVA placebo I**<br>1x 1ml of saline given i.m	**MVA placebo II**<br>1x 1ml of saline given i.m

9.2 Doses and mode of vaccine delivery

All DNA/Placebo immunizations will be performed with a Biojector device according to the manufacturer's instruction. Intradermal (i.d.) injections will be given in the skin over the deltoid muscle with the extension supplied by the manufacturer for this purpose. The DNA injection of 0.1 ml is performed at a concentration of 3mg/ml in Groups I and II, and 2 mg/ml in Group III. The MVA-boost injection will be delivered intramuscularly in the deltoid muscle.

9.3 Vaccination procedures and safety follow-up after immunization

The immunizations will take place in an outpatient setting. Participants will receive a baseline medical status check up including vital signs assessment to confirm on the medical eligibility before vaccine administration. Results will be documented in the appropriate CRF forms ***(CRF 3-II, CRF 10-I)***. Safety lab procedures, urine analysis as well as HIV testing will be performed before vaccine administration according to the visit schedule. A pregnancy test for female participants before each vaccination will have to show negative results vaccination. The pregnancy test for female participants before each vaccination will have to show negative results *before* vaccination. A vaccine request form ***(CRF 10-II)*** indicating the study visit number, the site and participant code, the date as well as the immunization number and immunization Group will be filled then by the study medical officer and transferred to the study pharmacist, who will deliver to the nurse the appropriate vaccine/placebo corresponding to the assigned study group. After each immunization participants will be closely observed at the clinic for 30 minutes. Vital signs and any local or systemic reaction will then be recorded and participants will be seen briefly by the medical officer to document the post vaccination status (***CRF 3-II, CRF 10-I*** - this record will be indicated in the CRF with the visit number plus A). The needles, syringes and other materials will be disposed according to MNH /MMRP biohazard regulations. As with any parenteral vaccine, epinephrine and corticosteroids will be available for immediate use should an immediate hypersensitivity reaction, such as anaphylaxis, occur. Intravenous injections of vaccines will NOT be performed. Study vaccine will not be administered to individuals with hypersensitivity to any component of the vaccine. All immediate hypersensitivity reactions will be managed according to the developed SOP's. Volunteers will be contacted by phone, home visit or clinical contact at the first day following immunization for a brief adverse reaction interview. Additionally, volunteers will complete diaries over events for the evening of the vaccination day and the 7 days following each vaccination. A diary card (***see Appendix 8***) will be provided after each vaccination schedule, with instructions and a full verbal explanation for volunteers to record local and systemic adverse events following immunization, as well as any medication taken. A folder with diary cards, disposable thermometers and a ruler will be issued to each volunteer. The clinical team will provide contact information as well as a telephone numbers to call 24 hours, in order to assure contact with the study personnel if needed.

All volunteers will be asked to contact the study site in the case of adverse events of suspected grade 2 or more for evaluation. A decision to offer the volunteer a visit to the clinic should be made and documented in the source documents. All volunteers with suspected grade 3 or more should be seen by the study physician and documented in the source documents.

Additional visits may be recommended at the discretion of the clinical and principal investigators, if clinically indicated or in order to clarify observations.

Safety follow-up visits are scheduled at week 2 and week 4 after each immunization visit. Diary cards will be reviewed and discussed latest at the scheduled 2 weeks follow-up visit after each vaccination. Diary entries will be transcribed after revision and discussion with the participant during the follow up visits after each vaccination into the Vaccine/Reaction Form (**CRF 10-I**). All concomitant medication must be recorded in study form **CRF 4-I** with the

reason for administration, the dosage regimen, the onset and end of treatment. All medical conditions (symptoms and/or diagnosis) that are not defined and indicated in the vaccine reaction form (**CRF 10-1**) will be recorded in the adverse events form (**CRF 6-I**) including the severity grade and the relationship to the study agent. Each of the 7 recording days will be recorded as a visit (**CRF 2-II**) with the visit number of the respected vaccination visit together with a capital letter indicating the recording day for adverse reaction (B for day 1, C for day 2, etc.).

The need for the following medications will lead to study discontinuation

- Immunomodulatory agents (i.e. immunoglobulin)
- Immunosuppressive agents (i.e., systemic steroids, chemotherapy)
- Live, attenuated vaccines. Other vaccines (subunit or killed) should be given at least two weeks before or two weeks after HIV immunization.

10 THE VACCINE (TRIAL PRODUCTS)

10.1 DNA Priming

Will be effected with DNA plasmids derived from plasmid puC8 and carrying a kanamycin resistance gene, promoter from human cytomegalovirus (hCMV), poly A signal from Human Papilloma Virus 16 and origin of replication for *E. coli*. The plasmids will be carrying HIV-1 genes of subtypes A, B and C. These are pKCMV**gp160**A, pKCMV**gp160**B, pKCMV**gp160**C, pKCMV**rev**, pKCMV**p37**A(ba), pKCMV**p37**B, and pKCMV**RT**B.

The primary envelope gene is that of subtype B. This gene was used for homologous recombination to obtain variable sites of subtypes A and C. Thus the most variable regions of gp120 from subtypes other than the primary could be included, and the most conserved proteins conserved in the vaccine mixture [44-46].

<u>*Supply, storage and composition of DNA*</u>

Vecura Company (Karolinska University Hospital at Huddinge, Stockholm, Sweden) will be responsible for bulk manufacture of the DNA vaccine, release testing and technical release of vialled product and labeling. All procedures will be according to Good Manufacturing Practice. The presentation is in liquid form at either 2 or 3 mg per mL, which should be stored at –20 ^{0}C until use. Care will be taken not to break the cold chain.

The DNA plasmids were approved for human use on November 27th 2004 by the Swedish Medical Products Agency, (Svenska Läkemedelsverket), and the second revision of the protocol ethically cleared by the Swedish Internal Review board, (Regionala forskningsetiska kommitten) in December 2004. The new batch of the same plasmids will need to have a further approval by the Tanzania Food and Drugs Authority (TFDA).

Sterile commercially available normal saline for human use will be used as the placebo.

<u>*Labels*</u>

DNA will be packaged by Vecura. The trial products will be in vials pre-labeled according to standardized operating procedures. Each vial will be packed in a box labeled with the name of the clinical site and principal investigator, the storage details and the name of the supplier of the product, the name of the manufacturer, as well as the manufacture and expiry dates. The labels should also have the caption “for clinical trial only”.

Cartons will be supplied to the designated pharmacist at MUHAS and NIMR-MMRP for storing the used vials, labeled with the supplier, the name of the trial and protocol version, the name of the clinical site and principal investigator and the content of the material (DNA vaccine or placebo).

Having been manufactured by Vecura Company, the DNA vaccine will be supplied to the study by the Swedish Institute for Infectious Disease Control, 171 82 Solna. SWEDEN.

Prior to use the vials will be thawed at room temperature. Thawed vials will then gently be swirled. Care will be taken not to shake or invert the vials.

10.2 MVA Boosting

Boosting will be effected by a Modified Vaccinia Ankara vaccine (MVA-CMDR). The MVA - CMDR, produced by WRAIR/NIH, is a live recombinant poxvirus vector vaccine that has been genetically engineered to express the following HIV-1 genes:

1. **gp**150 (Subtype E, CM235), and
2. **gag** and **pol** (integrase-deleted and reverse transcriptase nonfunctional, Subtype A, CM240).

MVA-CMDR is formulated in a liquid form in vials and will be administered at a dose of 10^8 pfu by intramuscular route following appropriate dilutions.

<u>*Supply, storage and composition of MVA*</u>

Walter Reed Army Institute of Research (WRAIR), USA, will be responsible for the bulk manufacture of the clinical material, release testing, as well as the technical release of vialled product, and labeling. The full address of the manufacturer is: *Walter Reed Army Institute of Research (WRAIR), Forest Glen Section, Building 501, Department of Biologics Research The Pilot Bioproduction Facility, Silver Spring, MD 20910, USA.* All procedures will be according to Good Manufacturing Practice. The presentation is in liquid form at 10^8 pfu per mL with an extractable volume of 1ml vials, which should be stored at -20 ^{0}C up to 3 months (-80°C if longer then 3 months) until use. Care should be taken not to break the cold chain. The product has already received approval from the USA Food and Drugs Authority (FDA), and the Swedish Medical Products Agency and will be presented to the TFDA for approval before use in Tanzania.

Sterile commercially available normal saline for human use will be used as the placebo.

<u>*Labeling*</u>

Each vial will be packed in a box labeled with the name of the clinical site and principal investigator, the storage details and the name of the supplier of the product, the name of the manufacturer, as well as the manufacture and expiry dates. The labels should also have the caption “for clinical trial only”.

<u>*Measures to ensure cold-chain maintenance and storage*</u>.

Cold chain will be maintained by transportation packages according to manufacturer’s instruction during shipment. The packing at Sweden will be divided into two different parcels (one parcel for each site) to avoid re-opening the parcel before transporting to Mbeya site. The storage facilities have automatic backup generators to maintain storage temperature. At Dar es Salaam site, transport of the investigational product from microbiology laboratory to clinic site will be done using study van in a container packed with ice bags. At the MMRP site investigational products will immediately be transferred and stored into the existing freezer at

the MMRP pharmacy. The study pharmacy and study clinic are located on the same ground within the same building.

Transportation to the Mbeya Site
The Pharmacist at Dar es Salaam site will be responsible for transporting the investigational products allocated to Mbeya site after being handled to him/her by world courier at Dar es Salaam Airport. In the same day, the investigational products will be transported using study van to Mbeya site. Investigational products allocated to Mbeya site will be transported into their original packaging containers without opening at Dar es Salaam site. The pharmacist at Mbeya site will receive the products handled to him/her by filling the handling documents before storing into the respective storage temperatures.

10.3 Dispensing records and disposal of unused product

The designated pharmacist will, upon receipt of supplies prior to commencement of the trial, conduct an inventory and complete a receipt, one copy of which will be retained at the site, and the original returned to the supplier (Karolinska Institute, Sweden). During the trial the pharmacist will be responsible for reviewing the dispensing log. On the day of immunization the study nurse will fill in the vaccine request form with the study visit number, the site and participant code, the date as well as the immunization number and immunization Group (2x versus 5x i.d. DNA priming). Upon receipt of the syringes with the vaccine from the pharmacist, the study nurse will deliver the vaccine. The immunization number and date will be entered against the study number in the dispensing log. The vial label will be cross-checked against the details on the vaccine request form and dispensing log by two individuals (pharmacist and nurse), prior to the vaccine/placebo being administered.

The Pharmacist will be responsible for ensuring that the return of used vials is recorded in the dispensing log at the end of the clinical session, and that they are placed in the appropriate volunteer carton. At the end of the trial all used and unused vials will be checked against the inventory by the monitor before disposal on site according to MNH/MMRP biohazard regulations. Documentation of disposal will be provided to the monitor and supplier. During the trial, product accountability will be monitored by the vaccine request form, the dispensing log, the returns, the trial register and data collected on the clinical research forms.

11 ENDPOINTS

11.1 Safety

The safety of immunization with the seven DNA plasmids carrying HIV-1 genes and the MVA will be assessed by clinical features as well as standard biochemical and hematological laboratory tests. Any worsening of the severity grade will be considered for causality with the vaccine.

Safety endpoints: Adverse events will be assessed using a standard format for soliciting local and systemic reactogenicity to the vaccine and collection of unsolicited adverse events. Solicited reactogenicity will be evaluated for 7 days following each vaccination. All other AE will be collected from the time of first injection until 3 months after the last injection.

The primary safety parameters will be graded according to the DAIDS Table for Clinical and Lab Criteria for Definitions ***(see Appendix 9)*** and are:

1. Local adverse event Grade 3 or above (pain, cutaneous reactions including indurations)
2. Systemic adverse event Grade 3 or above (fever, chills, headache, nausea, malaise, myalgia, and arthralgia)

3. Other clinical and laboratory adverse event Grade 3 or above confirmed at examination or on repeat testing respectively

The relationship to the vaccine should be evaluated as:

1= not related
2= probably not related
3= possibly related
4= probably related
5= definitely related

Tests for possible toxicity due to vaccination will include: Liver function tests, renal function tests and complete blood count and differential with platelets. Any event attributable to vaccine leading to discontinuation of the immunization regimen must be documented.

Data on local and systemic events listed above will be solicited with specific questions or using a diary card for a minimum of 7 days following each immunization. Data on other clinical events and laboratory events will be collected with an open question at each visit and through routine scheduled investigations respectively.

11.2 Immunogenicity

HIV-specific cellular and humoral immune responses will be assessed.

Primary endpoint
The primary immunogenicity endpoint will be assessed at the trial sites by the IFN-γ ELISPOT assay using fresh cells.

Secondary endpoints
Intracellular cytokine staining quantifying IFN-γ and IL-2 production by CD4 and CD8 T-cells and lymphoproliferation against inactivated HIV using fresh cells.
Multi-color intracellular cytokine staining using cryopreserved cells for assessment of multiple T-cell effector functions including immunophenotyping of responding cells.
Binding (and if present, also neutralizing) antibodies.

12 SAFETY AND IMMUNOGENICITY ASSESSMENTS

12.1 Safety assessment

Local and systemic assessments whether normal or abnormal will be recorded starting from 30 minutes up to 4 weeks after immunization in the following intervals: 30 minutes as direct observation after immunization, at the evening of the vaccination day and at days 1-7 as recorded by diary cards by the participants with clinic or home visit if indicated, plus follow-up safety visits 2 weeks and 4 weeks after each immunization. While study personnel will use the vaccine/reaction form ***(CRF 10-I)*** to record all local and systemic events at the clinic, participants will use diary cards at home for the same. On the Adverse Events Form ***(CRF 6-I)*** all medical events throughout the entire study period will be reported, excluding those local or systemic reactions indicated on the Vaccine/Reaction Form ***(CRF 10-I)*** during the first 7 days post vaccination. The study site will contact participants at the first day after vaccination for an adverse events/reaction interview. Participants will be instructed to call or report to the specified study personnel immediately if any unusual or severe sign or symptom appears after immunization. Participants with suspected grade 3 or above events should be seen in the clinic at the time of maximal symptoms, if possible, and will be followed up clinically until resolution of symptoms.

An adverse event is any undesired, noxious or pathological change in a participant as indicated by physical signs, symptoms, and/or laboratory changes that occurs following administration of one of the vaccines, whether or not considered vaccine related. This definition includes intercurrent illnesses or injuries, and exacerbation of pre-existing conditions.

12.1.1 Local adverse events

Pain at the injection site will be graded by the volunteer according to the criteria in the DAIDS Table for Clinical and Lab Criteria for Definitions ***(see Appendix 9)*** as mild (1) moderate (2), severe (3) or serious/life threatening (4) and recorded in the appropriate case record form. In the diary card grading will refer only to mild (1) moderate (2) and severe (3) as the distinction between severe and serious should be made in association with the medical officer after having reported to the clinic once the volunteer has left clinic. Swelling, redness and indurations will be recorded as the maximum diameter as well as the presence of any itching or other discomfort and any medication taken for relief of symptoms. Blistering (vesiculation) or ulceration will be graded according to size, depth, time to healing and character of blisters (blood-filled).

Clinical staff will complete the case record form following each immunization. In the event that there are two observations on the same day, those confirmed at a visit will take precedence over diary card observations in the analysis. Study personnel should verify all grade 3 or 4 events.

12.1.2 Systemic adverse events

Temperature will be measured by the axillary route prior to immunization and 30 minutes after the immunization by study personnel and graded according to the DAIDS Table for Clinical and Lab Criteria for Definitions. Participants will be given thermometers to record their temperature in the diary card on the evening of immunization, and daily thereafter for 7 days, and if still raised, they will be advised to continue to monitor their temperature daily until it returns to normal. The temperature observed by the clinical team will be recorded in the case record forms for visits. In case of grade 3 and 4 fever, malaise, chills/rigors and headache, and where indicated, additional tests to exclude other common causes of fever like malaria will be performed.

Chills, headache, nausea, malaise, myalgia, and arthralgia will be graded by the participants according to mild, moderate or severe impression and recorded in the diary card on the evening following immunization and daily for 7 days, or until resolution of symptoms whichever is longer. At the next study clinic visit diary entries will be reviewed and discussed by the medical officer with the participants and events will be transferred into the case report form according to the DAIDS Table for Clinical and Lab Criteria for Definitions. Data entry in the CRF will take precedence over diary card entries in the analysis. Study personnel will verify all grade 3 or 4 events.

12.1.3 Other adverse events

These will be recorded as reported following an open question to volunteers, with the dates of commencement and resolution, the outcome at the end of the study, required treatment, relationship to the study agent and potential effects on the vaccination schedule. They will be graded according to the general principles outlined in the DAIDS Table for Clinical and Lab Criteria for Definitions. Social harm will also be recorded as an adverse event, graded according to the general guidelines (which include denial of health and life insurance,

employment, immigration and marriage relationships). All grade 3 or 4 events will be verified by study personnel.

12.1.4 Routine laboratory parameters for safety assessments

The following safety assessments will be undertaken at appropriate laboratories at MUHAS and MMRP according to standard operating procedures. All lab values will be interpreted according to established MMRP and MUHAS local reference ranges. Lab results requiring AE reporting after enrolment will be graded according to the DAIDS Table for Clinical and Lab Criteria for Definitions ***(see Appendix 9)***. In order to harmonize with local reference ranges, the cut-off for Grade I lab toxicity reporting have been modified for neutrophils from ≤1.300 cells/µl (DAIDS) to ≤1.100 cells/µl (local values).

Hematology
Hemoglobin; white blood cell count, total and differential counts, specifically granulocytes and lymphocytes; platelet count and red blood cell counts.

Clinical chemistry
Liver function tests (ALT, bilirubin total and direct), serum creatinine and blood glucose. A Troponin I test will be performed 2 weeks after each MVA vaccination.

Immunology
Determination of CD4 counts and percentage only at the enrolment visit.

Serology
Syphilis (VDRL, and when necessary TPHA) and screening for HBsAg during screening. Tests might be repeated during the study if clinically indicated.

Urinalysis
Urine dipsticks will be performed during screening and at each vaccination day by clinical nursing staff and recorded as normal/abnormal. If abnormal results are seen (≥ +1) urine specimen will be send to the clinical lab for microscopic analysis and validation. Results will be graded by a study Physician as normal, abnormal/clinically insignificant and abnormal/clinically significant.

Pregnancy test
Pregnancy test will be performed by study nurses at the clinic or at the study lab from urine during screening, at each vaccination day with results available before vaccine administration and at the long term follow-up visits 17 (week 58) and 18 (week 70)."

ECG
ECG will be done during screening/before enrolment as a baseline assessment to exclude volunteers with significant ECG findings. Significant ECG findings leading to exclusion are defined as "Abnormality in ECG that could indicate risk or make interpretation of vaccine effects difficult according to the study operating procedures." According to this definition volunteers will be excluded who have:

1. Evidence in the ECG for cardiologic disease
2. Variations in the ECG which might not be clinical significant but will complicate later interpretation for post vaccination ECG results.

ECG's will be interpreted by competent study physicians and be graded as ***normal***, ***abnormal/clinically insignificant*** and ***abnormal/clinically significant.*** Any abnormal finding

will be commented in the case report form ***(CRF 9-III).*** All ECG's will be sent prior to enrolment to a cardiologist panel for review and approval via email. The final verdict on the ECG with regards to enrolling or not enrolling the volunteer into the study will be provided by the Chairperson of the panel. The ECG panel will consist of:

Dr. Bernard R Chaitman (Chairperson)
chaitman@swbell.net

Dr. Johnson Lwakatare
medi@raha.com

ECG will be repeated 2 weeks post MVA/Placebo vaccinations or at anytime whenever a volunteer comes with symptoms and signs suggestive of peri-myocarditis. Results will be interpreted and compared to baseline ECG results by competent study physicians and send to the cardiologist panel for review and approval. Post vaccination ECG will be graded as ***normal***, ***abnormal/clinically insignificant*** and ***abnormal/clinically significant***. Post vaccination ECG findings will be graded as ***no changes, changes/not significant*** and ***changes/significant*** as compared to baseline results. Any abnormal finding and changes will be commented in the case report form ***(CRF 9-III).***

All grade 3 or 4 adverse events or serious events and/or those that necessitate a physician's visit or are treated with a prescribed medication will be evaluated. The volunteer will be followed carefully until the condition is resolved and/or the cause is identified. Any medication or other therapeutic measure taken to relieve symptoms of the medical problem must be recorded on the appropriate case report form page(s) ***(CRF 4-I)*** in addition to the outcome of the adverse event ***(CRF 6-1)***. Where a diagnosis is possible, it is preferable to report this rather than a series of terms relating to the diagnosis. When reporting a syndrome, the associated signs and symptoms will be parenthetically indicated following the syndrome rather than as separate events

12.1.5 Following adverse event

Other assessments (e.g. extended clinical chemistry, malaria microscopy, stool analysis, urine culture, TB culture) may be performed as clinically indicated at the sites due to an adverse event. There is the possibility to ship blood samples for specific investigation to specialized laboratories in Europe for further diagnosis.

12.2 Immunogenicity assessment

12.2.1 Antibody responses

Antibodies to HIV proteins covering the vaccines will be assessed using ELISA. Following completion of the study, neutralizing antibodies to HIV subtypes A, C and D will be performed at SMI if binding antibodies are detected.
Serum samples at different time points will be collected as per specimen schedule and will be stored until the time for the assays at the end of the study. In vivo antibody neutralization will be used for testing. At this point in time, it is not known which time point sample is going to induce broadly neutralizing antibodies or not. Therefore, testing will be performed on the last serum sample collected 2 weeks post vaccination and other earlier time point samples should the last sample testing show any interesting results

The specific assessment of HIV-1 specific antibody responses will thus include:

- Serum binding antibody determination by ELISA and Western blot assay

- Serum neutralization antibody assay including cross clade reactivity (against HIV-1 subtypes A, C and D which are prevalent in Tanzania)

12.2.2 Cellular responses

The following HIV-1 specific cellular immune responses will be determined:

- CD8 and CD4 T cell responses
  - IFN-γ ELISPOT using fresh PBMC including determination of cross clade responses, using pools of overlapping peptides representing env, gag and pol proteins
  - IFN- γ/IL-2 Intracellular cytokine staining (ICS) using fresh PBMC and conducted against the same peptide pools as in the ELISPOT and testing both for CD8 and CD4 T cell responses
  - Multicolor ICS staining for measuring polyfunctional responses using cryo-preserved PBMC, including cytokines/chemokines and CD107 (for assessment of degranulation activity) and testing for both CD8 and CD4 T cell responses

- Mainly CD4 T cell responses

  Lymphocyte proliferation assay (LPA) to the immunizing antigens using fresh PBMC and a standard ^{3}H-thymidine uptake assay and /or a flow cytometry-based assay (CFSE staining or FASCIA). A comparative evaluation of lymphocyte proliferation assays for measuring HIV vaccine–induced immune responses will be performed before the start of the TaMoVac trial.

- Optional assays
  - Viral inhibition assay
  - Cytotoxic T lymphocytes (CTL) by chromium release assay

The primary immunogenicity endpoint will be measured by a standard IFN-γ ELISPOT assay using fresh PBMC. ELISPOT responses are considered positive if the numbers of spot-forming cells (SFC) are at least four times the background (RPMI medium only) and > 55 SFC per million PBMC (Merck definition).
Quality control and exchange of specimens with international laboratories will be performed.

12.2.3 Storage of Cell pellets for HLA testing

Lymphocyte cell pellets will be stored at the MUHAS department of Microbiology/Immunology and at the MMRP Immunological Lab for later HLA typing that will be performed by molecular methods at a laboratory to be identified later.

12.3 Other assessments

12.3.1 HIV testing

Samples will be tested at MUHAS Department of Microbiology/Immunology and at MMRP laboratories using HIV antigen/antibody ELISA. Positive results will be confirmed by a duplicated second ELISA and after confirmation with a HIV PCR. In the case of discordant results a duplicated second ELISA and if needed a Western Blot analysis and HIV PCR will be used (***see Appendix 7***).

12.3.2 Antibodies against Vaccinia

Serum samples will be stored at MUHAS, Department of Microbiology/Immunology and MMRP for later testing.

13 CONCOMITANT MEDICATION

Volunteers/participants will be asked about medication taken at each visit. All medication including other vaccinations (e.g. tetanus toxoid) or herbal/traditional remedies will be recorded in the case record form ***(CRF 4-I)***. Documentation should include the generic name of the drug, indication for use, dose and route of administration, frequency, start and stop dates. For medication available over the counter or other sources, the maximum information available on questioning the volunteer will be recorded. For combination medication all generic drug names included will be named into one entry (e.g. Coartem® = Artemether + Lumefantrine). For herbal or traditional remedies as much information as possible will be sought about the content, the indication or the indigenous name.

14 ADVERSE EVENTS

14.1 Definitions and Classification

An adverse event (AE) is any untoward medical occurrence in a patient or clinical investigation subject administered a pharmaceutical product and which does not necessarily have a casual relationship with this treatment. An AE can therefore be any unfavorable and unintended sign (including an abnormal laboratory finding), symptom, or disease temporally associated with the use of a medicinal (investigational) product, whether or not related to the medicinal (investigational) product (ICH E6). An adverse event in this study is any adverse experience occurring during the course of the study starting after vaccination and will be documented in the adverse report form (CRF 6-I). During the screening period any previous, current or ongoing conditions will be documented in the previous condition case report form (CRF 4-II). Adverse reaction as defined in the vaccine/reaction form (CRF 10-I) during the 7 days post vaccination will not be again recorded in the AE case report form unless one of the symptoms can be clearly associated with another diagnosis (e.g. fever post vaccination with the diagnosis of confirmed malaria). Criteria for grading clinical and laboratory events are listed in the DAIDS Table for Clinical and Lab Criteria for Definitions **(*see Appendix 9*)**.

The severity of events reported on the adverse event form will be determined by the investigator, based on the Toxicity Grading Scale or if the event is not listed the event will be graded following these guidelines:

- Mild (Grade 1): Transient or mild symptoms; no limitation in activity; no intervention required. Symptoms causing no or minimal interference with usual social and functional activities
- Moderate (Grade 2): Symptoms causing greater than minimal interference with usual social and functional activities. No or minimal intervention required. It is uncomfortable or an embarrassment.
- Severe (Grade 3): Symptoms causing inability to perform usual social and functional activities. Medical intervention often required.
- Life-threatening (Grade 4): Symptoms causing inability to perform basic self-care functions OR Medical or operative intervention indicated to prevent permanent impairment, persistent disability, or death.

The relationship of adverse events to study product will be classified as:

Not related: Adverse events felt to be due to extraneous causes that neither follows a known pattern of response nor a reasonable temporal relationship to study product.

Probably not related: Adverse events that are unlikely to be related to product but which follow a reasonable temporal relationship, such that this cannot be completely excluded or events that could be associated with product but which are unrelated in time.

Possibly related: Adverse events that may be due to extraneous causes but which follow a known pattern of response and/or a reasonable temporal relationship to study product.

Probably related: Adverse events that cannot be explained by extraneous causes; which follow a known pattern of response and/or a reasonable temporal relationship; which disappear or decrease on cessation of study product and reappear on re-challenge.

Definitely related: Adverse events that have a definite relationship to the study agent (e.g. anaphylactic reaction after vaccination) without any other explained causes; which follow a known pattern of response and/or a reasonable temporal relationship; which disappear or decrease on cessation of study product and reappear on re-challenge.

A severe adverse event is any **graded 3 or 4** by criteria in DAIDS Toxicity Table (*Appendix 7*). Some, but not all grade 3 and 4 adverse events will be "serious" by ICH GCP criteria below.

14.2 Serious Adverse Events (SAEs)

An adverse event is considered to be a "serious adverse event" by ICH Good Clinical Practice (ICH E6 and E2A) criteria if it results in the following:

1. Death,
2. A threat to life (any grade 4 event)
3. Requires in-patient hospitalization or prolongs existing hospitalization (hospitalization for elective treatment of a pre-existing condition is not included),
4. Results in persistent or significant disability or incapacity,
5. Is a congenital anomaly (i.e. the outcome of pregnancy involving a volunteer),
6. Is any other important medical condition*.

*Examples of conditions regarded as "any other important medical condition" include allergic bronchospasm requiring intensive emergency treatment, seizures or blood dyscrasias, which did not result in hospitalization or development of drug dependency.

Note that grade 4 lab abnormalities are not serious adverse events, unless they are related to a clinically event matching the SAE definition.

Any HIV infection occurring throughout the study period will be defined as a Grade 4 Serious Adverse Event and has to be reported to the study monitor and DSMB accordingly.

14.3 Reporting of adverse events

Adverse and serious adverse events should be recorded on the appropriate case record form ***(CRF 6-I)***. Any grade 3 or 4 adverse event or any event resulting in discontinuation of the

vaccination schedule should be reported within 2 working days after site awareness to the study monitor, sponsor, TCC, WRAIR and DSMB. The TCC, WRAIR and DSMB will review and make the appropriate recommendations to the monitor, who will transmit these to the sponsor for a decision on the continuation of the study. The decision whether to continue, pause or discontinue the study will be submitted to the monitor, Steering committee, TCC, the local IRB, NIMR and TFDA for further review and action

Serious adverse events should be reported to the monitor and DSMB the same day on an expedited reporting level at the same working day that the Clinical Investigator becomes aware of the event when the following criteria are fulfilled:

1. Result in death regardless of relationship to study agent.
2. Congenital anomalies, birth defects, or fetal losses regardless of relationship to study agent.
3. Result in persistent or significant disabilities or incapacities regardless of relationship to study agent.
4. Any suspected adverse drug reaction, i.e., definitely, probably, possibly, and probably not related to a study agent that requires or prolongs existing hospitalization, or requires intervention to prevent significant/permanent disability or death.
5. Any life threatening (including all Grade 4 adverse events) suspected adverse drug reactions, i.e., definitely, probably, possibly, and probably not related to a study agent.

“Life threatening” refers to an adverse event that at occurrence represents an immediate risk of death to the subject. An experience which may have caused death had it occurred in a more severe form is not considered life threatening. Similarly, a hospital admission for an elective procedure is not considered a Serious Adverse Event.

In addition to the reporting requirements described above, sites should report any of the following adverse events on an expedited basis:

- Suspected adverse drug reactions, i.e., definitely, probably, possibly, and probably not related to a study agent, that do not meet the protocol-required reporting criteria, but the Investigator believes are of sufficient concern to be reported on an expedited basis to the study monitor and DSMB. This includes adverse events that, based upon appropriate medical judgment, may jeopardize the subject and may require medical or surgical intervention to prevent a serious adverse event. Examples of such events are intensive treatment in an emergency room or at home for allergic bronchospasm or blood dyscrasias or convulsions that do not result in hospitalization.
- Unexpected, serious suspected adverse drug reactions, i.e., definitely, probably, possibly, and probably not related to a study agent, that occur at any time after the protocol-defined expedited reporting period if the study staff become aware of its occurrence. These events include deaths, permanent disabilities, congenital anomalies, hospitalizations, and life-threatening clinical events. (Do not report Grade 4 laboratory values unless associated with a life-threatening clinical event.)
- Serious adverse events that are not related to a study agent, but could be associated with study participation or procedure (e.g., pulmonary embolism secondary to an intravenous catheter placed for study agent administration).

Examples of SAEs that do not require expedited reporting include:

1. Hospitalization for scheduled surgery unrelated to vaccine, other than temporally (within 30 days).
2. Orthopedic or traumatic injuries requiring hospitalization.
3. Hospitalization planned for pre-existing conditions not due to an aggravation in the condition.
4. SAEs occurring more than 30 days after vaccination or having appeared before vaccination without any aggravation after vaccination

Reporting of serious adverse events or expedited serious adverse events can be done by telephone, e-mail or fax. The minimum criteria required in reporting a SAE are the volunteer identifiers (trial number, site and participant code, date of birth), reporting source (name of the site, the reporting site investigator and the site PI), a description and grading of the adverse event, the onset of the adverse event and current status, the relationship to the study agent and why the adverse event is identifiable as serious. Associated medication, lab information and other relevant clinical information shall be provided as soon as possible. For reporting SAE and expedited SAE the SAE reporting form ***(CRF 6-II)*** should be used.

Important Adverse Event Reporting contacts are:

Name	***Position***	***email***
Sören Andersson	SMI, Sponsor	soren.andersson@smi.se
Muhammad Bakari	PI	drbakari@yahoo.com
Muhsin Aboud	MUHAS Sponsor	maboud@muhas.ac.tz, abouduhsin@gmail.com
Eric Sandström	Internal monitor, Karolinska Institute	eric.sandstrom@sodersjukhuset.se
Gunnel Biberfeld	SMI	gunnel.biberfeld@smi.se
Britta Wahren	SMI	britta.wahren@smi.se
Eligius Lyamuya	MUHAS	eligius_lyamuya@yahoo.com
Leonard Maboko	MMRP, site PI	lmaboko@mmrp.org
Arne Kroidl	MMRP, site CRC	akroidl@mmrp.org
Patricia Munseri	MUHAS, site CRC	pmunseri@yahoo.com
Michael Hoelscher	LMU	hoelscher@lrz.uni-muenchen.de
Merlin Robb	WRAIR	mrobb@hivresearch.org
Mary Marovich	WRAIR	mmarovich@hivresearch.org
Beryl Wessner	External monitor, WARIR	bwessner@hivresearch.org
Sheena McCormack	MRC	smc@ctu.mrc.ac.uk
Innocent Semali	Chairman of DSMB	isemali@muhas.ac.tz
Sayoki Mfinanga	NIMR	gsmfinanga@yahoo.com
NIMR chairman	NIMR ethics	headquarters@nimr.or.tz
MUHAS chairman	MUHAS IRB	drp@muhas.ac.tz
Mbeya chairman	Mbeya IRB	esamky@muchs.ac.tz
TFDA chairman	TFDA	info@tfda.or.tz

Any serious adverse event or SUSAR should be reported within 2 working days on **CRF 6-II** after site awareness to the study monitor, sponsor, TCC, WRAIR and DSMB (***Appendix 10***). The TCC, WRAIR and DSMB will review and make the appropriate recommendations to the monitor, who will transmit these to the sponsor for a decision on the continuation of the study. The decision whether to continue, pause or discontinue the study will be submitted to the

monitor, Steering committee, TCC, the local IRB, NIMR and TFDA for further review and action

Trial Management Group (TMG) research safety calls will be performed every other week and upon request/need. The calls will be organized by the Medical Research Council Clinical Trials Unit in London/UK chaired by Sheena McCormack.

15 PROTOCOL DEVIATION

Any deviation of the study schedule or study protocol will have to be recorded in the appropriate case report form ***(CRF 7-II)*** including the date, a description, the category and the reason of the deviation. Also a description of potential steps taken to resolve deviation or how to avoid recurrence should be attempted. These deviation should be reported to the study monitor, sponsor, TCC, WRAIR and DSMB within 2 working days. The TCC, WRAIR and DSMB will review and make the appropriate recommendations to the monitor, who will transmit these to the sponsor. Records of these reports will be submitted to the, Steering committee, TCC, the local IRB, NIMR and TFDA at regular intervals.

16 DISCONTINUATION PROCEDURES

Volunteers/participants may withdraw at any time if they wish to do so, for any reason. The date of withdrawal and reason for doing so should be recorded in the appropriate case record form ***(CRF 7-I)***. The clinical investigator may decide that it is not in the best interests of the volunteer/participant to proceed to the next immunization following an adverse event (see below). The discontinuation and reason should be forwarded to the study monitor, sponsor and DSMB within the next 2 working days. Trial visits should carry on at least until resolution or stabilization in the case of an event, but ideally up to the last visit in the schedule, provided the volunteer is willing. The frequency of visits and laboratory investigations may be reduced on consultation with the Principal Investigator or Trial Management Group

16.1 Criteria for withdrawal of volunteers from injection schedule

Under certain circumstances, a volunteer will be terminated from participating in further injections. These events include:

1. HIV infection
2. Pregnancy
3. Grade 3 or 4 systemic events classified as probably or definitely associated with immunization.
4. Type 1-hypersensitivity associated with immunization.
5. Serious intercurrent illness that is not expected to resolve prior to next scheduled immunization.
6. Any medical condition that can recur/deteriorate with further immunizations.
7. Need for systemic glucocorticoids or other immunomodulators than NSAIDs for any reason. Repeated failure to comply with protocol requirements.
8. The Sponsor, vaccine provider or principal investigator decides to stop or cancel the study.
9. The NIMR and or MUHAS or Mbeya Hospital ethical committee decides that the study be stopped.

Trial volunteers who are discontinued from additional study injections will continue to be followed according to the schedule to further evaluate safety and monitor adverse events. They will also continue to be covered by the medical insurance until the end of the study period.

16.2 Criteria for pausing the study

1. The study monitor will closely monitor and analyze study data as it becomes available and will make determinations regarding the presence and severity of the adverse events and keep the sponsor informed.
2. Any grade 3 systemic event that has been judged to be definitely related to the vaccination (following exclusion of other unrelated common causes) or if one volunteer experiences grade 4 SAE assessed as possibly related to the vaccination. During the pause the DSMB will be consulted with the facts on the case, and a review of the event in a broader context will be undertaken. Their verdict will then be conveyed to the steering committee and the sponsor for review and decision on what to do next.
3. If any AE report raises concern that the study may cause significant harm to the participants.folloing a safety board consensus decision ***(See section 14.3 study monitor, sponsor, TCC, WRAIR and DSMB).***

The study injections and enrolments would resume only after review of the adverse events by the DSMB and a decision by the TCC and steering committee including the Chairman resulting in a recommendation to the sponsor to permit further study injections and study enrolments. These decisions will be cleared by the local IRBs, NIMR and TFDA. Safety data and changes in study status will be submitted to the ethics committee.

17 CLINICAL MANAGEMENT

The TMGs who will assess and treat the event as appropriate will manage events. The TCC will meet at regular intervals during conference calls (once weekly during the vaccination period, every second week after completion of vaccination schedules until the end of the study) to discuss the progression of the study, any relevant medical or adverse events as well as up-coming other study related issues. An independent medical monitor will be assigned for each study site to assure independent judgment on medical events.

17.1 Management of HIV issues during and following the trial

Individuals who test HIV-positive during the screening process will be referred for HIV care, treatment and support at the HIV/AIDS clinics at MNH and Mbeya Referral Hospital. At these clinics the following services will be provided; sustained counseling about HIV/AIDS, counseling on diet, management of symptoms and opportunistic infections as well as provision of antiretroviral drugs if indicated. The study sites will provide all clinically relevant information in the form of a referral report to the HIV/AIDS clinic. CD4 T-cell counts and viral loads will be done at the study clinics before referral.

17.2 HIV testing

Only volunteers with a non-reactive antigen/antibody HIV ELISA result will be enrolled. The HIV testing (simple rapid Antibody test at the Laboratory) will be obtained before each vaccination} and at the long-term follow-up visit at study weeks 84 (Visit 18) and 108 and whenever a volunteer has the need to establish his/her HIV status (Visit 19).

It is possible that volunteers may develop antibodies and test 'positive' in routine commercial HIV ELISA assays subsequent to immunization. The laboratories at MUHAS, Department of Microbiology/Immunology and MMRP, will conduct molecular tests (HIV-1 PCR) to distinguish between infection and a post-immunization response required either for clinical management, or at the request of a volunteer. For unblinding reasons laboratories will only provide the

information to the clinic whether a participant is “infected” or “uninfected” to not expose false positive ELISA results suggestive for vaccine or placebo randomization.

After study completion participants will be advised to attend the study clinic for any further HIV testing to avoid confusion of false positive test results. After the completion and analysis of the whole study with consecutive unblinding of the study Groups, participants will receive an individual unblinding session, indicating the vaccine Group plus false positive HIV test results. In the event of ongoing post-immunization positive ELISA, the volunteers will be invited to attend a specified centre annually until such time as this response has disappeared, and provided with an explanatory identity card in the interim.

17.3 Verification of HIV status of volunteers

If certification is required at the request of the recruited volunteer/participant, this can be provided by the trial Principal investigator after testing at the trial laboratory. A member of the counseling study team will always give HIV test results at post-test counseling session, unless the volunteer/participant requests otherwise. If a specimen from a volunteer/participant suggests that the volunteer is HIV infected, a second specimen will be collected, retested and the results confirmed. If a volunteer/participant for any reason needs to establish his or her HIV status extended tests will be offered as described above.

17.4 Management of volunteers who acquire HIV infection during the study period

In the unexpected circumstances that a volunteer in the trial acquires HIV infection they will be managed in the following way:

Referral for clinical care

Volunteers will be referred initially to a study HIV specialist physician at MUHAS/MNH or NIMR-MMRP/Mbeya Referral Hospital for a full discussion of the clinical aspects of HIV infection and planning for further care. Further investigations will be undertaken as necessary. At this clinic the following services will be provided: sustained counseling about HIV/AIDS, counseling on diet, management of symptoms and opportunistic infections as well as provision of antiretroviral drugs if indicated.

Referral for counseling

A referral to a counselor at the HIV clinic at MNH/Mbeya Referral Hospital will be arranged by the specialist physician. The counseling process will assist the volunteer in the following issues:

a. Psychological and social implications of HIV infection
b. Who to inform and what to say
c. Implications for sexual partners
d. Facilitation of risk reduction plan
e. Avoidance of risk to others in the future
f. Information on available medical treatment

Informing the other involved heath care personnel

The volunteer will be encouraged to do this, but the decision will remain at the discretion of the individual.

Immunological follow-up

Follow-up of HIV infected individuals who have received study vaccine products will be determined by the Trial Management Group for the duration of the study and after the study period they will be referred to the existing treatment center. The intensity of assessments will

be dependent on clinical progression, including changes in surrogate markers such as CD4 count.

If a volunteer is found to be HIV-infected during the trial, he/she will be excluded from further immunizations in the trial.

17.5 Social discrimination as result of a post-vaccine response

The aim is to minimize the possibility of social discrimination in volunteers who develop a positive HIV-ELISA test by providing HIV testing by PCR and certification for volunteers as required. In addition, an identification card stating that the individual has participated in a vaccine trial, with a contact number in case of medical emergency, will be provided. In the unlikely event that a volunteer suffers social discrimination as a result of a post-vaccination response, the clinical investigators will assist the volunteer by asking the volunteers to attend at the study site for counseling and testing whereby the necessary HIV testing including HIV-DNA/PCR will be performed to rule out HIV infection.

18 TRIAL DATA MANAGEMENT

18.1 Data management and entry at the clinical centre

Data will be recorded directly onto the case report forms, which will provide the majority of source data for the trial or entered onto a source document checklists and transcribed to the appropriate case report form later. There will be some additional source data in the clinical notes, such as medical history related to eligibility, results of laboratory analyses and details of clinical management (description of adverse events and concomitant medication). The site research coordinator and internal monitors will crosscheck the completeness of the CRF's or the correctness of data transcription to the CRF's from source documents at the clinic. The original copies of all CRF's and source documents will be kept at the clinic in the volunteers/participants study binder, which is placed in a secured and lockable locker at the study site. The documents in the binder will be organized according to study number study visit. Any changes to the CRF's should be signed and dated.

Data entry from CRF's will be performed at the sites by data entry clerks who are under supervision of a site data manager. All data will be double entered into a computerized database. An internal data monitoring with inconsistency and logic checks will run before double data entry. The resolution of queries and clearance of inconsistent data will be performed by the site data manager. Enquires might be communicated with the clinic and will be passed for review by the trial physician if required. Consistency checks and range checks will be performed at the data entry level on a daily basis. Cleared data will then be transferred to the Central Data Management Centre.

18.2 Data management in the clinical/immunology laboratories

Standardized operating procedures will be followed in all laboratories to ensure the quality of data. Data will be stored in both hard copies and electronically in an agreed format. As for clinical data all results from the laboratory will be reviewed and signed by the lab manager for completeness and correctness. Clinical lab reports will be sent as signed copies to the clinical sites where lab reports will be transcribed into the appropriate CRF's and filed as source documents in the volunteer's/participant's study binder. The original copy will be stored at the laboratory. In Mbeya lab data is first entered by the lab and then by the data entry unit. At MUHAS double data entry will be performed only by the data entry clerks.
Immunogenicity data is managed entered and analyzed by the responsible immunologist on site (single data entry) and will then be transferred to the Central Study Lab in Sweden and

further on to the Central Data Management Centre. Cellular immunology units participate in external PT panels for ICS (BD) and ELISpot (NIH) to ensure proper working of the assays.

18.3 Data monitoring at the Central Data Management Centre

The location of the data management centre is still to be determined and will be either situated at Mbeya supported by LMU in Munich or in Dar es Salaam conducted by NIMR. Data from both sites will be centrally checked for inconsistencies and logic. Queries and reports will be produced and referred to the clinical site data manager for resolution. Data queries might also include clarification on adverse event description (diagnosis and symptoms), medications or the relationship of medical events, medications and relationship to study agents to each other. Enquiries will be communicated with the clinical sites and will be passed for review by the trial physician if required. Copies including documentation detailing all the queries and changes will be stored in a study specific binder at the trial coordination centre.

18.4 Archiving

Study related documents including clinical notes, completed CRF’s and original laboratory results, hard copies and electronic copies will be kept in a secure location at MUHAS and MMRP.

18.5 Data ownership

The data generated in this study will be the property of the TaMoVac Investigators and will be held on their behalf by MUHAS and NIMR-MMRP until completion of the study or until it is stopped. Thereafter the data will be passed over to MUHAS and NIMR-MMRP for archival purposes according to accepted regulation for a minimum of 15 years. If required at an earlier date for the purposes of regulatory submissions, a request should be made to the Trial Coordinating Committee.
WRAIR will require access to all data in order to meet obligations with full reporting of data to the FDA regarding any use of the MVA product. KI/SMI will maintain data in accordance with US FDA guidelines until 2 years after licensure or retiring the IND.

19 STUDY MONITORING

Internal study monitoring will be performed as described above. An external trial monitor will be appointed by the sponsors of the study, MUHAS and SMI; he/she will be responsible to the Principal Investigators of the Project and the Trial Coordinating Committee to ensure that the trial is conducted according to ICH GCP guidelines including monitoring vaccine data consistency, vaccine accountability, dispatch and arrival of immunological specimens.

19.1 Preparation of reports to assist the monitoring

The monitor will visit the clinical centers to validate trial data against the clinical records. The following data should be verifiable from source documents:

i. Site and study participants registration number, sex, birth date, address
ii. Documentation of any existing conditions or past conditions relevant to eligibility
iii. A signed consent
iv. Dates of visits including dates of immunizations
v. Source verification of all data entered into the database
vi. Grade 3 or 4 adverse events and any events leading to discontinuation of the immunization schedule
vii. Concomitant prescribed medication

Vaccine stocks will also be monitored at visits to the clinical centre/pharmacy.

19.2 Trial committees

19.2.1 Trial Coordinating Committee (TCC)

The supervision of the trial will be a responsibility of the Trial Coordinating Committee (TCC). The members will include the 2 Principal Investigators, study directors, clinical coordinators, representative from WRAIR, laboratory coordinators, study nurse and data manager. The TCC will be meeting at least once a week during the trial period. The TCC will prepare a monthly report for presentation at the TaMoVac steering committee meeting. This committee will be responsible for decisions about grade of adverse events and relationship to study vaccine. The final decisions on grading of AE and relationship with vaccine may be ascertained by an independent End-Point Committee. Notes of meetings will be kept, and the Committee will report to the TaMoVac Steering Committee. The trial may be suspended by this Committee for any reason, including on the recommendation of the DSMB.

19.2.2 Trial Management Group (TMG)

This group will oversee the day-to-day running of the trial and the members will be primarily the clinical and data management teams. The members will be among others, the site PI, clinical coordinator, laboratory coordinator, study Pharmacist, study nurse and data manager. The lab manager will join if there are relevant items on the agenda. Notes will be taken and will form the basis of the progress report to the Trial Coordinating Committee. Each site will have its own TMG.

19.2.3 TaMoVac Steering committee

The TaMoVac Steering committee will review the notes from the TCC meetings regarding progress of the trial, and will be meeting/communicating once a month. The committee will have an independent chair and a secretary and advice the sponsor on the conduct of the study. The steering committee may initiate an interim analysis provided blinding is maintained..

19.3 Data and Safety Monitoring Board (DSMB)

An independent Data and Safety Monitoring Board will be appointed by the sponsor of the study, MUHAS and SMI. The role of the DSMB will include:

1. To review the research design and the plans for data and safety monitoring
2. To receive and review at intervals the progress reports from the investigators
3. To monitor safety data
4. To monitor and evaluate trial progress, including
    a. Periodic assessment of data quality and timelines
    b. Performance of the study sites
5. To recommend to the sponsor, local IRB's and investigator concerning continuation or to stop the study.

The DSMB will meet whenever a safety report has been submitted and make a recommendation to the TCC within 7 days as to the future conduct of the trial.
They will also meet on one occasion to review the safety data, after half the volunteers have received the first immunization in order to make recommendations to the sponsors of the study.

19.4 Community Advisory Board (CAB)

At each study site there will be a Community Advisory Board drawing representative members from the Police Force and general population in Dar es Salaam and representative members from the general population in Mbeya. There may also be members from the media, legal representation and study participants. In Mbeya a CAB has been established since 2005 and

regular meetings are ongoing since then concerning current and past research activities. In Dar es Salaam the CAB will meet before the onset of the study for a general study information session, discussion of recruitment strategies, outreach activities and tracing, participant identification, insurance, and compensation issues. The CAB will be informed in detail about benefits and risks concerning study participation, informed consent procedures as well as HIV risk questioners. The purpose of this meeting is also to receive comments about the comprehensibility and clarity of the proposed participant study information/briefing sessions and informed consent procedures. Regular CAB meetings will then be held at 3 monthly intervals throughout the trial. Up-dates or changes of the study status and information of general HIV Vaccine related issues (e.g. STEP trial) will be provided for the CAB members. Problems concerning individual study participants also in respect to community related topics (e.g. stigmatization) will be discussed in an anonymous fashion and additional CAB meetings might be called at any time in the case of special circumstances.
In addition, the wider Tanzanian community has since the first high profile National HIV Vaccine Strategy Plan Workshop held in Bagamoyo in 2001 been informed through the mass media on the need and on plans for HIV vaccine trials in Tanzania. The public will continue to be informed through press releases and through other channels on all the stages of the TaMoVac trial.

19.5 Indication for additional review

There will be an additional meeting of the DSMB if 3 or more volunteers experience an unexplained, unexpected grade 3 or 4 clinical or laboratory event (confirmed on attendance or repeat testing) not resolved within 72 hours and considered probably or possible and likely to be related to vaccine product.

20 STATISTICAL CONSIDERATIONS

By the end of this study 120 study participants will have been exposed to DNA or placebo as well as MVA or saline placebo and described in the quantitative and qualitative immunogenicity and safety evaluation.
The sample size for this study is similar to other Phase 1 safety and immunogenicity studies of vaccines published in the literature. In the primary analysis, the results of the IFN-gamma ELISpot 2 weeks after the second MVA will be used as the primary endpoint. We expect 100% of those in group IIIA (controls) to be responders. We will need 36 in each group to be able to detect a 20% difference in either group IA or IIA, with a power of 80% and 5% significance level (one sided). In each vaccine group, 4 volunteers will be assigned to receive placebo. Statistical significance may not be attained with such low power but would provide justification of the need for a study with greater power. The sample size will allow preliminary evaluation of between-group variability.

20.1 Analysis

All safety end-points will be graded by the Clinical Investigator and reviewed by the Trial Management Group. Any queries about grade and relationship to study product that cannot be resolved will be referred to the Trial Coordinating Committee for a final decision.
The primary safety end-points are: The safety of immunization with the seven DNA plasmids carrying HIV-1 genes as assessed by clinical signs, and standard clinical chemistry/hematological laboratory tests where any worsening of the severity grade will be considered for causality by the vaccine.

The alternative immunization applications (2x i.d. versus 5x i.d. with the Biojector device) will be evaluated for adverse events by assessing local (pain, cutaneous including indurations),

general (fever, chills, headache, nausea, arthralgia, malaise, myalgia) and other unsolicited adverse events within 7 days.

Immunogenicity endpoints
The primary immunogenicity endpoint is the frequency, magnitude and quality of HIV-specific cellular immune responses determined by the IFN-γ ELISPOT assay.
The secondary immunogenicity endpoints include cellular immune responses determined by ICS and T cell proliferative responses and antibody responses determined by binding and neutralizing antibody assays.

20.2 Statistical analysis
The final analysis will be conducted once all study participants have received all their immunizations and completed the 84 weeks follow-up period, or have permanently withdrawn from the study. Safety analyses will further be updated in the event that there is 108 weeks follow up. Analyses will be based on the principle of intention-to-treat. Interim analysis of safety and immunogenicity may be performed if measures are taken to maintain blinding.

20.3 Volunteer characteristics
Baseline characteristics will be summarized by study Groups, using appropriate summary statistics. Study participant characteristics to be summarized will include demographic variables and baseline values relevant to safety and immunogenicity.

20.4 Safety data
The number and percentage of subjects experiencing any adverse events (AE), grade 3 or 4 adverse events, serious adverse events (SAE), and the number of events reported, will be summarized by severity and reported relationship to study treatment for each treatment group, both for all adverse events and by type of adverse event. Calculation of incidence rates of adverse events will include each subject only once, either according to worst severity or first reported event.

Rates of the various adverse events will be compared between the study Groups using Fisher's exact test. Rates will be summarized using exact binomial confidence intervals

Safety hematological and clinical chemistry laboratory parameters will be summarized at each study visit according to study Group. Comparisons will be done using 2-sample t-tests or non-parametric equivalents as appropriate to the data

20.5 Immunogenicity Data
Immunogenicity endpoints will be compared formally at baseline and two weeks after the last immunization, according to study dosing group. Binary endpoints will be compared using Fischer's exact test. Continuous endpoints will be compared using 2-sample t-tests or non-parametric equivalents as appropriate to the data.

Immunogenicity of the candidate vaccine will be determined based on comparisons between study treatment Groups. Associations between immunogenic endpoints will be assessed using multivariate regression models.

21 ETHICAL CONSIDERATIONS
Full medical confidentiality will be preserved. The study will be conducted according to ICH GCP guidelines and the Declaration of Helsinki (Version 2008), and it is the responsibility of the Clinical Investigators to ensure adherence. Before the study starts all personnel involved

in the clinical and laboratory trial units will be trained for Good Clinical Practice (GCP) and/or Good Laboratory Practice (GLP) respectively. Study personnel will also be trained on standard operating procedures (SOPs) that are related to the protocol and will follow the GCP and GLP guidelines.

This protocol has been submitted to the WHO VAC advisory board for review. The input has been incorporated in this version of the protocol, see appendix 10. It will then be submitted to the National Ethics Committee at the National Institute of Medical Research (NIMR) for ethical approval. NIMR is registered with the Office for Human Research Protections (OHRP), US (FWA00002632). in parallel the proposals will be submitted to the institutional ethical committees. These are the Research and Publications Committee at the Muhimbili University of Health and Allied Sciences (MUHAS) having the FWA 00004301, and the Mbeya Municipal Medical Authorities in Mbeya (FWA00002469), and subsequently to the appropriate ethics committee in Sweden and Germany. The DNA and MVA vaccines, which will be donated by the KI/SMI in Sweden, have been approved by the Swedish Medical Products Agency and are currently used in the ongoing clinical trials in Sweden. The US Food and Drug Authority has previously approved the MVA which is in ongoing clinical trials in the USA. Approval of these immunogens will also be undertaken independently by the Tanzania Food and Drug Authority (TFDA).

The Principal and Clinical Investigators are also responsible for informing the ethics committees of any SAE's as required, and submitting annual reports as required. No study materials will be obtained from study volunteers before approval from the relevant bodies. The KI/SMI in Sweden will be responsible for providing the Investigator's Brochure (IB) for DNA as well as the Investigator's brochure for MVA.

The TCC will be responsible for preparing the randomization list, all aspects of data management including monitoring of the clinical sites, and the analysis. Staff will also be responsible for coordinating the response to any SAE's that arise during the course of the trial and reporting these if indicated to the regulatory authorities.

22 INDEMNITY

Insurance for investigators

The Principal Investigator will ensure that relevant clinical and laboratory staffs engaged in the study are covered for possible legal actions against the investigators for harm through indemnity insurance schemes, which will be paid for by the project during the study period.

Health care Insurance for trial volunteers

All trial volunteers will be provided with health insurance cover for injury or death related to the trial through insurance schemes.

Vecura Company in Sweden will have an insurance that covers any defect in the production of the DNA vaccine only.

SMI will in the role of sponsor assume the liability for the DNA vaccine and under a separate agreement with the Karolinska Institute its role in the CRADA with Walter Reed Army Institute for the MVA.

23 PROTOCOL AMENDMENTS

The TCC will be responsible for preparing protocol amendments needed by the TaMoVac Steering Committee. Amendments to the protocol will be made only after consultation and

agreement between sponsors and Principal investigators. The only exception is where any of the site PIs considers that a volunteer's safety is compromised without immediate action. All amendments that have an impact on volunteer risk or the study objectives, or require revision of the informed consent document, must receive approval from the relevant ethical committees prior to their implementation.

24 PUBLICATION

It is intended that the results of this study will be published in an appropriate peer-reviewed journal, with the aim of submitting a paper for publication within 6 months of the study's completion.

The TaMoVac Trial Coordinating Committee and Bioject Inc will have 30 days to comment on any manuscript. No other publications, whether in writing or verbally, will be made before the definitive manuscript has been agreed and accepted for publication, without the prior approval of this committee. A final report of the study will be prepared by the Investigators and circulated to the Steering Committee for comments.
The presenting author for the first conference presentation of the main trial results will be one of the Tanzanian principal investigators or their deputy.

Apart from the international publication(s), results from the trial will be presented at various national and international conferences. Regular updates will be provided to the study participants, relevant local authorities as well as local media for wider dissemination.

25. REFERENCES

[1] UNAIDS Global Report 2007
http://www.unaidsorg/en/KnowledgeCentre/HIVData/GlobalReport/2008/2008_Global_report.asp
[2] UNAIDS AIDS Epidemic Update 2007.
http://www.unaidsorg/en/KnowledgeCentre/HIVData/EpiUpdate/EpiUpdArchive/2007/
[3] UNAIDS Global Report 2006.
http://dataunaidsorg/pub/GlobalReport/2006/2006_GR_CH02_en.pdf.
[4] The United Republic of Tanzania, Ministry of Health and Social Welfare, National AIDS Control Programme. HIV/AIDS/STI Surveillance Report, January – December 2005. March 2007. Report Number 20.
[5] Bakari M, Urassa W, Pallangyo K, Swai A, Mhalu F, Biberfeld G, et al. The natural course of disease following HIV-1 infection in dar es salaam, Tanzania: a study among hotel workers relating clinical events to CD4 T-lymphocyte counts. Scand J Infect Dis 2004;36(6-7):466-73.
[6] Bakari M, Lyamuya E, Mugusi F, Aris E, Chale S, Magao P, et al. The prevalence and incidence of HIV-1 infection and syphilis in a cohort of police officers in Dar es Salaam, Tanzania: a potential population for HIV vaccine trials. AIDS 2000 Feb 18;14(3):313-20.
[7] Matee MI, Lyamuya EF, Mbena EC, Magessa PM, Sufi J, Marwa GJ, et al. Prevalence of transfusion-associated viral infections and syphilis among blood donors in Muhimbili Medical Centre, Dar es Salaam, Tanzania. East Afr Med J 1999 Mar;76(3):167-71.
[8] Tanzania Demographic and Health Survey 1992/1992, Bureau of Statistics, Dar es Salaam, Tanzania.
[9] Tanzania Reproductive and Child Health Survey 1999, Bureau of Statistics, Dar es Salaam, Tanzania.
[10] Riedner G, Hoffmann O, Rusizoka M, Mmbando D, Maboko L, Grosskurth H, et al. Decline in sexually transmitted infection prevalence and HIV incidence in female barworkers attending prevention and care services in Mbeya Region, Tanzania. AIDS 2006 Feb 28;20(4):609-15.
[11] Hoelscher M, Kim B, Maboko L, Mhalu F, von Sonnenburg F, Birx DL, et al. High proportion of unrelated HIV-1 intersubtype recombinants in the Mbeya region of southwest Tanzania. AIDS 2001 Aug 17;15(12):1461-70.
[12] Kiwelu IE, Renjifo B, Chaplin B, Sam N, Nkya WM, Shao J, et al. HIV type 1 subtypes among bar and hotel workers in Moshi, Tanzania. AIDS Res Hum Retroviruses 2003 Jan 1;19(1):57-64.
[13] Lyamuya E, Olausson-Hansson E, Albert J, Mhalu F, Biberfeld G. Evaluation of a prototype Amplicor PCR assay for detection of human immunodeficiency virus type 1 DNA in blood samples from Tanzanian adults infected with HIV-1 subtypes A, C and D. J Clin Virol 2000 Jun;17(1):57-63.
[14] Renjifo B, Chaplin B, Mwakagile D, Shah P, Vannberg F, Msamanga G, et al. Epidemic expansion of HIV type 1 subtype C and recombinant genotypes in Tanzania. AIDS Res Hum Retroviruses 1998 May 1;14(7):635-8.
[15] Cao H, Kaleebu P, Hom D, Flores J, Agrawal D, Jones N, et al. Immunogenicity of a recombinant human immunodeficiency virus (HIV)-canarypox vaccine in HIV-seronegative Ugandan volunteers: results of the HIV Network for Prevention Trials 007 Vaccine Study. J Infect Dis 2003 Mar 15;187(6):887-95.
[16] Mugerwa RD, Kaleebu P, Mugyenyi P, Katongole-Mbidde E, Hom DL, Byaruhanga R, et al. First trial of the HIV-1 vaccine in Africa: Ugandan experience. BMJ 2002 Jan 26;324(7331):226-9.
[17] Jaoko W, Omosa G, Bhatt K. Safety and immunogenicity of DNA and MVA vaccines in phase I HIV-1 vaccine trials in Nairobi, Kenya. AIDS Vaccine Conference; 2004; Laussane, Switzerland, August 30th – Sept 1st. Oral abstract No 56; 2004.

[18] Esparza J, Osmanov S, Pattou-Markovic C, Toure C, Chang ML, Nixon S. Past, present and future of HIV vaccine trials in developing countries. Vaccine 2002 May 6;20(15):1897-8.
[19] HIV gp120 vaccine - VaxGen: AIDSVAX, AIDSVAX B/B, AIDSVAX B/E, HIV gp120 vaccine - Genentech, HIV gp120 vaccine AIDSVAX - VaxGen, HIV vaccine AIDSVAX - VaxGen. Drugs R D 2003;4(4):249-53.
[20] RV144 Phase III HIV Vaccine Trial Press Release. http://www.hivresearch.org/phase3/phase3pressrelease.html.
[21] Buchbinder SP, Mehrotra DV, Duerr A, Fitzgerald DW, Mogg R, Li D, et al. Efficacy assessment of a cell-mediated immunity HIV-1 vaccine (the Step Study): a double-blind, randomised, placebo-controlled, test-of-concept trial. Lancet 2008 Nov 29;372(9653):1881-93.
[22] Robb ML. Failure of the Merck HIV vaccine: an uncertain step forward. Lancet 2008 Nov 29;372(9653):1857-8.
[23] Harari A, Bart PA, Stohr W, Tapia G, Garcia M, Medjitna-Rais E, et al. An HIV-1 clade C DNA prime, NYVAC boost vaccine regimen induces reliable, polyfunctional, and long-lasting T cell responses. J Exp Med 2008 Jan 21;205(1):63-77.
[24] Bråve A, Wahren B. Experimental and clinical approaches for genetic immunization against HIV-1. DNA Vaccines; 2008; Las Vegas, USA; 2008.
[25] Mazzoli S, Trabattoni D, Lo Caputo S, Piconi S, Ble C, Meacci F, et al. HIV-specific mucosal and cellular immunity in HIV-seronegative partners of HIV-seropositive individuals. Nat Med 1997 Nov;3(11):1250-7.
[26] Rowland-Jones S, Sutton J, Ariyoshi K, Dong T, Gotch F, McAdam S, et al. HIV-specific cytotoxic T-cells in HIV-exposed but uninfected Gambian women. Nat Med 1995 Jan;1(1):59-64.
[27] Koulinska IN, Ndung'u T, Mwakagile D, Msamanga G, Kagoma C, Fawzi W, et al. A new human immunodeficiency virus type 1 circulating recombinant form from Tanzania. AIDS Res Hum Retroviruses 2001 Mar 20;17(5):423-31.
[28] Leandersson AC, Gilljam G, Fredriksson M, Hinkula J, Alaeus A, Lidman K, et al. Cross-reactive T-helper responses in patients infected with different subtypes of human immunodeficiency virus type 1. J Virol 2000 May;74(10):4888-90.
[29] Rutebemberwa A, Bess JW, Jr., Brown B, Arroyo M, Eller M, Slike B, et al. Evaluation of aldrithiol-2-inactivated preparations of HIV type 1 subtypes A, B, and D as reagents to monitor T cell responses. AIDS Res Hum Retroviruses 2007 Apr;23(4):532-42.
[30] Bråve A, Ljungberg K, Boberg A, Rollman E, Engstrom G, Hinkula J, et al. Reduced cellular immune responses following immunization with a multi-gene HIV-1 vaccine. Vaccine 2006 May 22;24(21):4524-6.
[31] Dorrell L, Williams P, Suttill A, Brown D, Roberts J, Conlon C, et al. Safety and tolerability of recombinant modified vaccinia virus Ankara expressing an HIV-1 gag/multiepitope immunogen (MVA.HIVA) in HIV-1-infected persons receiving combination antiretroviral therapy. Vaccine 2007 Apr 30;25(17):3277-83.
[32] Gilbert PB, Chiu YL, Allen M, Lawrence DN, Chapdu C, Israel H, et al. Long-term safety analysis of preventive HIV-1 vaccines evaluated in AIDS vaccine evaluation group NIAID-sponsored Phase I and II clinical trials. Vaccine 2003 Jun 20;21(21-22):2933-47.
[33] Jaoko W, Nakwagala FN, Anzala O, Manyonyi GO, Birungi J, Nanvubya A, et al. Safety and immunogenicity of recombinant low-dosage HIV-1 A vaccine candidates vectored by plasmid pTHr DNA or modified vaccinia virus Ankara (MVA) in humans in East Africa. Vaccine 2008 May 23;26(22):2788-95.
[34] Peters BS, Jaoko W, Vardas E, Panayotakopoulos G, Fast P, Schmidt C, et al. Studies of a prophylactic HIV-1 vaccine candidate based on modified vaccinia virus Ankara (MVA) with and without DNA priming: effects of dosage and route on safety and immunogenicity. Vaccine 2007 Mar 1;25(11):2120-7.

[35] Excler JL, Rida W, Priddy F, Fast P, Koff W. A strategy for accelerating the development of preventive AIDS vaccines. AIDS 2007 Nov 12;21(17):2259-63.
[36] Plotkin SA. Vaccines: correlates of vaccine-induced immunity. Clin Infect Dis 2008 Aug 1;47(3):401-9.
[37] Sandström E, Nilsson C, Hejdeman B, Bråve A, Bratt G, Robb M, et al. Broad immunogenicity of a multigene, multiclade HIV-1 DNA vaccine boosted with heterologous HIV-1 recombinant modified vaccinia virus Ankara. J Infect Dis 2008 Nov 15;198(10):1482-90.
[38] Bakari M, Mhalu F, Aboud S, Nilsson C, Francis J, Janabi M, et al. Safety and immunogenicity of an HIV-1 DNA plasmid vaccine boosted with HIV-1 MVA among Police Officers (PO's) in Dar esSalaam, Tanzania (HIVIS03). AIDS Vaccine Conference. Cape Town, South Africa 2008.
[39] Arroyo MA, Hoelscher M, Sateren W, Samky E, Maboko L, Hoffmann O, et al. HIV-1 diversity and prevalence differ between urban and rural areas in the Mbeya region of Tanzania. AIDS 2005 Sep 23;19(14):1517-24.
[40] Herbinger KH, Gerhardt M, Piyasirisilp S, Mloka D, Arroyo MA, Hoffmann O, et al. Frequency of HIV type 1 dual infection and HIV diversity: analysis of low- and high-risk populations in Mbeya Region, Tanzania. AIDS Res Hum Retroviruses 2006 Jul;22(7):599-606.
[41] Hoffmann O, Zaba B, Wolff B, Sanga E, Maboko L, Mmbando D, et al. Methodological lessons from a cohort study of high risk women in Tanzania. Sex Transm Infect 2004 Dec;80 Suppl 2:ii69-73.
[42] Kibuuka H, Kimutai R, Maboko L, Schunk M, Kroidl A, Shaffer D, et al. Safety and Immunogenicity of the VRC Multiclade HIV-1 DNA Plasmid Vaccine/Adenovirus-5 Vector Boost Vaccine in a phase I/II Study in HIV-1 Uninfected Adult East African Volunteers. AIDS Vaccine Conference; 2008; Cape Town, South Africa; 2008.
[43] Schuetz A, Haule A, Mwalongo A, Kiwole C, Schindler K, Schunk M, et al. Cellular immune responses in HIV-1 uninfected adult Tanzanian volunteers enrolled in a phase I/II multiclade HIV-1 DNA plasmid vaccine. AIDS Vaccine Conference; 2007; Seattle, Washington; 2007.
[44] Ljungberg K, Rollman E, Eriksson L, Hinkula J, Wahren B. Enhanced immune responses after DNA vaccination with combined envelope genes from different HIV-1 subtypes. Virology 2002 Oct 10;302(1):44-57.
[45] Rollman E, Brave A, Boberg A, Gudmundsdotter L, Engstrom G, Isaguliants M, et al. The rationale behind a vaccine based on multiple HIV antigens. Microbes Infect 2005 Nov;7(14):1414-23.
[46] Wahren B, Ljungberg K, Rollman E, Levi M, Zuber B, Kjerrstrom Zuber A, et al. HIV subtypes and recombination strains--strategies for induction of immune responses in man. Vaccine 2002 May 6;20(15):1988-93.

Appendix 1: Study Timetable

Target day	Week	Visit	Visit purpose	Visit Window
	-4-8	V1	Screening I	4-8 weeks before V3 enrolment
	-2-4	V2	Screening II + baseline immunogenicity I	2-4 weeks before V3 enrolment
0	0	V3	Enrolment + DNA I + baseline immunogenicity II	Week 0
0		V3A	30 min post vaccination	
0		V3B	Evening of vaccination day	
1		V3C	Day 1 post vaccination	
2		V3D	Day 2 post vaccination	
3		V3E	Day 3 post vaccination	
4		V3F	Day 4 post vaccination	
5		V3G	Day 5 post vaccination	
6		V3H	Day 6 post vaccination	
7		V3I	Day 7 post vaccination	
14	2	V4	Follow-up safety (+ immunogenicity)	14 days post V3 +/- 3 days
28	4	V5	DNA II + Follow-up safety	28 days post V3 +/- 3 days
28		V5A	30 min post vaccination	
28		V5B	Evening of vaccination day	
29		V5C	Day 1 post vaccination	
30		V5D	Day 2 post vaccination	
31		V5E	Day 3 post vaccination	
32		V5F	Day 4 post vaccination	
33		V5G	Day 5 post vaccination	
34		V5H	Day 6 post vaccination	
35		V5I	Day 7 post vaccination	
42	6	V6	Follow-up safety + immunogenicity	14 days post V5 +/- 3 days
56	8	V7	Follow-up safety	28 days post V5 +/- 5 days
84	12	V8	DNA III	84 days post V3 +/- 7 days
84		V8A	30 min post vaccination	
84		V8B	Evening of vaccination day	
85		V8C	Day 1 post vaccination	
86		V8D	Day 2 post vaccination	
87		V8E	Day 3 post vaccination	
88		V8F	Day 4 post vaccination	
89		V8G	Day 5 post vaccination	
90		V8H	Day 6 post vaccination	
91		V8I	Day 7 post vaccination	
98	14	V9	Follow-up safety + immunogenicity	14 days post V8 +/- 3 days
112	16	V10	Follow-up safety	28 days post V8 +/- 5 days
210	30	V11	MVA I + immunogenicity	126 days post V8 +14 /-28 days
210		V11A	30 min post vaccination	
210		V11B	Evening of vaccination day	
211		V11C	Day 1 post vaccination	
212		V11D	Day 2 post vaccination	
213		V11E	Day 3 post vaccination	
214		V11F	Day 4 post vaccination	

215		V11G	Day 5 post vaccination	
216		V11H	Day 6 post vaccination	
217		V11I	Day 7 post vaccination	
224	32	V12	Follow-up safety + immunogenicity	14 days post V11 +/- 3 days
238	34	V13	Follow-up safety	28 days post V11 +/- 5 days
322	46	V14	MVA II + immunogenicity	112 days post V11 +14 /-28 days
322		V14A	30 min post vaccination	
322		V14B	Evening of vaccination day	
323		V14C	Day 1 post vaccination	
324		V14D	Day 2 post vaccination	
325		V14E	Day 3 post vaccination	
326		V14F	Day 4 post vaccination	
327		V14G	Day 5 post vaccination	
328		V14H	Day 6 post vaccination	
329		V14I	Day 7 post vaccination	
336	48	V15	Follow-up safety + immunogenicity	14 days post V14 +/- 3 days
350	50	V16	Follow-up safety + immunogenicity	28 days post V14 +/- 5 days
406	58	V17	Follow-up immunogenicity	84 days post V14 +/- 14 days
490	70	V18	Follow-up immunogenicity	168 days post V14 +/- 14 days

Appendix 2: TaMoVac I Visit Schedule and CRF Flow Chart

Visit Week	-4-8	-2-4	0	2	4	6	8	12	14	16	30	32	34	46	48	50	58	70
Target day			0	14	28	42	56	84	98	112	210	224	238	322	336	350	406	490
Visits	V1	V2	V3	V4	V5	V6	V7	V8	V9	V10	V11	V12	V13	V14	V15	V16	V17	V18
Visit description	Screening 1	Screening 2	Enrolment + DNA 1	Follow-up	DNA 2	Follow-up	Follow-up	DNA 3	Follow-up	Follow-up	MVA 1	Follow-up	Follow-up	MVA 2	Follow-up	Follow-up	Follow-up	Follow-up
Clinical Case Forms																		
CRF Informed Consent Screening	x																	
CRF 1-I Assessment of Understanding	x																	
CRF 1-II Risk Assessment	x																	
CRF 5-III Eligibility Form		(x)	x															
CRF 5-II Inclusion/Exclusion Form		(x)	x															
CRF 2-I Visit Contact Form Screening	x	x																
CRF 3-I Physical Exam Form Screening	x																	
CRF 5-IV Screening Outcome Form		(x)	x															
CRF 5-I Basic Demographic Form	x																	
CRF 2-II Visit Contact Form Enrolment			x															
CRF 2-III Visit Contact Form				x	x	x	x	x	x	x	x	x	x	x	x	x	x	x
CRF 3-II Physical Examination Form		x	x	x	x	x	x	x	x	x	x	x	x	x	x	x	x	x
CRF 4-II Previous Condition Form	(x)	(x)	x															
CRF 4-I Concomitant Medication Form	(x)	(x)	(x)	(x)	(x)	(x)	(x)	(x)	(x)	(x)	(x)	(x)	(x)	(x)	(x)	(x)	(x)	x
CRF 6-I Adverse Events Form				(x)	(x)	(x)	(x)	(x)	(x)	(x)	(x)	(x)	(x)	(x)	(x)	(x)	(x)	x
CRF 6-II SAE Reporting Events Form				(x)	(x)	(x)	(x)	(x)	(x)	(x)	(x)	(x)	(x)	(x)	(x)	(x)	(x)	(x)
Diary Card			x		x			x			x			x				
CRF 10-I Vaccine/Reaction Form			x		x			x			x			x				
CRF 10-II Vaccine Request Form			x		x			x			x			x				
CRF 8-I Randomization Pharmacy			x															
CRF 7-I Subject Status Form			x	(x)	(x)	(x)	(x)	(x)	(x)	(x)	(x)	(x)	(x)	(x)	(x)	(x)	(x)	x
CRF 7-II Protocol Deviation Form			(x)	(x)	(x)	(x)	(x)	(x)	(x)	(x)	(x)	(x)	(x)	(x)	(x)	(x)	(x)	x
CRF 11-I Pregnancy Report Form			(x)	(x)	(x)	(x)	(x)	(x)	(x)	(x)	(x)	(x)	(x)	(x)	(x)	(x)	(x)	x
CRF 9-IV Hematology Form	x		x	x	x	x	x	x	x	x	x	x	x	x	x	x	x	x
CRF 9-V Chemistry Form	x		x	x	x	x	x	x	x	x	x	x	x	x	x	x	x	x
CRF 9-VII Urine Analysis Form	x		x		x			x			x			x			x	x
CRF 9-VI Serology Form	x																	
CRF 9-VIII CD4 Count Form			x															
CRF 9-I HIV Status Form	x		x		x			x			x			x			x	x
CRF 9-III ECG Result Form		x										x			x			
CRF 9-II Pregnancy Result Form	x		x		x			x			x			x			x	x

Appendix 3: TaMoVac I Immunization and Lab Flow Chart

	Weeks		-4-8	-2-4	0	2	4	6	8	12	14	16	30	32	34	46	48	50	58	70
	Target days				0	**14**	18	42	56	84	98	112	210	224	238	322	336	350	406	490
	Visits		V1	V2	V3	**V4**	V5	V6	V7	V8	V9	V10	V11	V12	V13	V14	V15	V16	V17	V18
**Investigations**	Amount (ml)	Type and # of tubes	**Screening 1**	Screening 2	Enrolment + DNA 1		DNA 2			DNA 3			MVA 1			MVA 2				
**Hematology (H)**																				
CBC	3 ml	Purple top EDTA	3ml		3ml	3ml	3ml	3ml	3ml	3ml	3ml	3ml	3ml	3ml	3ml	3ml	3ml	3ml	3ml	3ml
Biochemistry (BC)																				
ALT, bilirubin, Creatinine, glucose	6 ml	Red top coagulant	6ml		6ml	6ml	6ml	6ml	6ml	6ml	6ml	6ml	6ml	6ml	6ml	6ml	6ml	6ml	6ml	6ml
Troponin I	From BC													from BC			from BC			
Serology																				
HbsAg, VDRL/TPHA	From BC	Red top coagulant	from BC																	
HIV test algorithm																				
HIV ELISA/WB	From BC	Purple top EDTA	from H		from H		from H			from H			from H			from H			from H	from H
Immunological tests																				
CD4 count	From BC	Purple top EDTA			from H															
HLA typing	8 ml	Purple top EDTA			8ml															
**Immunogenicity tests**																				
Binding and neutralizing Ab. incl. Vaccinia Ab.	1x10ml	Red top coagulant		X	X			X			X			X	X	X	X	X	X	X
IFN-gamma ELISPOT, fresh cells	9x10ml	Green top Na-heparin			X						X			X		X	X		X	X
ICS, fresh cells					X									X			X		X	X
Lymphocyte proliferation assay, fresh cells (optional)					X						X			X			X		X	X
ICS multi-colors, cryo-preserved cells					X						X			X			X		X	X
Optional assays, cryo-preserved cells				X	X			X			X			X	X	X	X	X	X	X
**Total volume for immunogenicity testing:**				100	100			100			100			100	100	100	100	100	100	100
**Total blood vol.= 1261 ml**			9	100	117	9	9	109	9	9	109	9	9	109	109	109	109	109	109	109
Urine																				
Urine for pregnancy test, urine dipstick	5 ml	Sterile urine container	x		x		x			x			x			x			x	x

D= DNA 1, 2, 3, M = MVA 1, 2 each to be given at specified visit; CBC – complete blood count, ICS - intracellular cytokine staining; CTL - cytotoxic lymphocyte assay; Red top=coagulant; Purple top=EDTA; Green top=sodium heparin tubes.
Stored samples will be used for antibody testing. Two weeks after DNA 2 PBMC will be collected for cryo-preservation. Tests using fresh cells will not be performed.

Appendix 4A: TaMoVac I Information Sheet (English)

***"A Phase I trial to assess safety and immunogenicity of i.d. DNA priming and i.m MVA boosting in healthy volunteers in Tanzania and to develop further vaccine trial capacity in Tanzania*" August 2010**

Dear prospective volunteer,
We would like to welcome you to read the information about the study and how to participate in a Phase I/II trial that aims to assess the Safety and Immunogenicity of a plasmid DNA-MVA prime boost HIV-1 vaccine candidate among volunteers in Dar es Salaam and Mbeya, Tanzania (TaMoVac I). The vaccine candidates have been developed in Sweden and in the USA based on the circulating HIV subtypes in Tanzania.
The study is performed at the two sites, the Muhimbili University of Health and Allied Sciences (MUHAS) and NIMR-Mbeya Medical Research Program (MMRP). This is a collaborative study involving the Karolinska Institute, the Swedish Institute for Infectious Disease Control (SMI) in Sweden, University of Munich in Germany, and the Imperial College of London, United Kingdom.

This is an early phase of research that wants to evaluate if the vaccine is safe and produces an immune response against HIV. The study does not test whether the vaccine works in preventing HIV infection, ***and therefore we do not know whether vaccinated people will be protected against HIV infection or not.***

This document gives you information regarding the purpose, how to participate, what you will have to do if you decide to participate, and potential benefits of participating as well as risks to you. This will therefore enable you to willingly make an informed decision as to whether to participate or not.

Purposes

The purposes of this research as said above are:

1. To determine the safety of the vaccine candidate
2. To determine the ability of the candidate vaccine in inducing body defense responses that may be capable of preventing infection with HIV.
3. To determine the minimum number of DNA vaccine injections that can elicit enough immune response.
4. To further develop the capacity in conducting HIV vaccine trials in Tanzania that includes strengthening the collaboration between the Tanzanian institutes involved in research on HIV vaccine, especially MUHAS, NIMR and MMRP.

The Vaccine

This candidate vaccine approach is known scientifically as DNA-MVA prime boost. The DNA part is made in Sweden and the MVA part is made in the USA. This vaccine product was investigated among 40 volunteers in Sweden and also in the ongoing HIVIS study in Dar es Salaam, Tanzania. In these studies the vaccine has been shown to be safe and elicited immune responses.

What is a DNA Vaccine?

DNA is the natural substance in the body that provides instructions for cells to make structures (proteins) and material for the cell and the body. The DNA vaccine contains a

piece of man-made DNA which contains instructions for some of the HIV-1 structures (proteins), found in the kind of HIV-1 that are common to the Americas, Asia, Europe, and Africa. DNA cannot reproduce or grow, but can carry instructions into the body of those who receive the DNA vaccine. With these instructions, the body cells can then build look-alike proteins that can imitate some of the proteins of the HIV-1 virus and this may stimulate immune responses, however, it does not make the HIV virus

What is a MVA Vaccine?
The MVA vaccine is made from a virus called Modified Vaccinia Ankara virus. MVA was developed as a vaccine against smallpox. In order to reduce adverse reactions to the conventional smallpox vaccine it was modified to prevent growth of the virus in the human body. It has been tried in over 100,000 people without serious adverse reactions. The MVA virus is a vehicle to carry information that imitates some of the proteins of the HIV-1 virus into the body to induce an immune response. The MVA virus cannot cause HIV. The MVA vaccine is given to increase the immune response produced by the DNA vaccine.
The original smallpox vaccine has been seen to cause inflammation in the heart in very few people. This has so far not been seen with MVA, but investigations are done to exclude that this can be the case.

The study
This study has received ethical clearance from the National Ethics Committee of the Ministry of Health and Social and Welfare, situated at the National Institute for Medical Research (NIMR) in Dar es Salaam; as well as the ethical committees at MUHAS in Dare s Salaam, and NIMR-MMRP in Mbeya.
The candidate vaccine has also been evaluated, registered and approved for use among Tanzanians, by the Tanzania Food and Drugs Authority (TFDA).

This study will enrol in total 120 HIV negative, healthy volunteers who have a very low likelihood of becoming HIV infected (Low Risk). 60 Volunteers will be enrolled in Dar es Salaam and 60 will be enrolled in Mbeya.
Through a chance process (randomization), 108 volunteers will receive 3 doses of DNA vaccine (Priming) at 0, 4, and 12 weeks; to be followed by 2 doses of MVA vaccine (Boosting). The first MVA vaccine will be given 18 weeks after the last DNA injection, and the second MVA vaccine will be given 16 weeks after the first MVA vaccine. The remaining 12 volunteers will receive a Placebo (Saline) injection, which is an inactive substance that looks like the vaccine candidate given at the same time intervals as the vaccine candidates. All volunteers will then be closely followed up for 24 weeks after the last injection.

Visits	1	2	3	4	5	6	7	8	9	10	11	12	13	14	15	16	17	18
Visit weeks	-4-8	-2-4	0	2	4	6	8	12	14	16	30	32	34	46	48	50	58	70
Visit description	Screening 1	Screening 2	Enrolment and DNA 1	Follow-up 1	DNA 2	Follow-up 2	Follow-up 3	DNA 3	Follow-up 4	Follow-up 5	MVA 1	Follow-up 7	Follow-up 8	MVA 2	Follow-up 9	Follow-up 10	Follow-up 11	Follow-up 12

How to Participate in the Study
You will have to sign in this form accepting that you have read or received oral presentation of the form, that you have understood the contents, and that you therefore agree to join this study.

You will qualify to take part in this study if you have completed the screening process, including this form, and have successfully answered the Test of Understanding questions. Furthermore, following medical evaluation you will have to be, among other things:

1. Between 18 and 40 years old.
2. Free of significant medical problems, including HIV infection or active Hepatitis B and syphilis
3. At low risk for HIV infection, including absence of dual or multiple concurrent relationships
4. Not pregnant or lactating
5. Willing to donate blood samples for research purposes

It is also important to remind you that to participate in this study you will need to agree to possible telephone contacts or home visits by the clinic staff. This will be done in order to remind you of your scheduled visit or for follow up.

A photograph will also be taken for your personal identification card (ID) that you will receive once you are considered eligible for study participation. You will be required to show your ID whenever you are visiting the clinic.

What will happen during clinic visits?
Screening Process: The Screening process will require at least two visits to this clinic At the first screening visit you are provided with detailed information about this study. After all your questions have been answered, and if you are willing to participate, you will be requested to sign the provided informed consent form. You will then undergo a test of understanding that wants to assure the investigators that you really have understood the concept of the study; you will have to pass the test of understanding. You will then be given a copy of the information sheet to keep as a personal record.

Thereafter you will undergo a Medical and laboratory screening process. In this you will be interviewed about your current and past health status as well as any medications taken recently. You will also be asked questions around your sexual behaviour.
You will then have a physical examination and several investigations done to ascertain that you are healthy, including 9 milliliters (about 2 table spoons) of blood drawn for laboratory tests at this visit. This is a very small amount of blood compared to the more than 5000 milliliters (5 liters) of blood, which you carry. The blood tests will include a check for diseases such as HIV, hepatitis, and syphilis, and a check of your general health. An ECG (heart test) will also be done. You will then be asked to provide a urine sample for tests aimed at making sure that you do not have problems with your kidneys and liver.
All females will also receive a pregnancy test from the urine sample.

This visit will take approximately 2-3 hours.

At the second screening visit about 2 weeks later, you will receive the results from the first tests and again have a discussion about the research.

The results of the screening tests may show that you cannot join this study, e.g. because of a relevant illness or abnormal lab results. The clinic staff will explain the results to you, and tell you about places where you can get support and medical care if you need it.
If you are pregnant or you are breastfeeding, you will not be able to join this study. You have to be able to practice adequate contraception for the duration of the study. If you are HIV positive, have active hepatitis B or syphilis; you cannot join this study either. If you are found to be HIV infected you will receive additional information about HIV and an appointment with doctors experienced in HIV care.

If you are found eligible for study participation and still feel comfortable and willing to participate the study personnel will again draw blood from you (about 120 milliliters, 14 table spoons) to determine your current immune status. This visit will take approximately 60 minutes.

<u>Study Enrolment Visit:</u> At the third visit, about 2 weeks after the last screening visit, you will again be asked about your willingness to participate in this study and the study team will once again confirm that you are eligible for study participation. If you still are willing to participate and you are found to be eligible, you will be enrolled as a study participant and receive your personal photo identification card. You will then be assigned by chance to one of the study groups and will either receive placebo (saltwater) or vaccine.

Groups IA and IIA: Will receive 2 injections/dose of the DNA vaccine under the skin followed by MVA in the muscle on the upper arm. The difference between Groups IA and IIA are due to different combinations of components within the vaccine.

Group III A: Will receive 5 injections/dose of the DNA vaccine under the skin followed by MVA in the muscle on the upper arm. This is the same dose that has been given in previous studies.

Groups IB and IIB: Will receive 2 injections/dose of salt-water (placebo) under the skin followed by salt-water injection into the muscle of the arm.

Group IIIB: Will receive 5 injections/dose of salt-water (placebo) under the skin followed by salt-water injection into the muscle of the arm.

Salt water (Placebo) is known to be safe and the body does not produce antibodies against it. For that reason salt-water will be used in this research for comparison purposes about the safety and the ability to produce immunological markers by the vaccine candidates.
It is important to realize that as a study participant you will not be able to choose the group that you would belong. Similarly, the study personnel as well as yourself, will not be aware as to whether you are receiving the vaccine or salt water until the end of study, or in case of an emergency that will require that information to be known.

At the enrolment visit you will receive the first vaccination according to the study group you have been randomized to. Before each vaccination a Doctor will ensure that your current health status is good. Blood and urine samples will be collected from you before vaccination. Also on each vaccination visit an HIV test will be repeated. Furthermore, all female participants will be tested for pregnancy from urine and a negative pregnancy test result will have to be present before the vaccine is administered.

While the DNA/Placebo vaccine will be delivered using a Biojector (a device without a needle) in the skin, the MVA/Placebo vaccine during the later vaccination visits will be given with an ordinary needle in the arm muscle as for most vaccines. After immunization you will remain at the clinic for 30 minutes so that you are closely observed. Vaccination visits will take approximately 1 hour and 30 minutes.

At each vaccination visit you will be provided with a diary card, in which you are asked to record potential side site effects as well as any medical condition or drugs taken following the 7 days after vaccination. This will be included in a folder that will also contain a thermometer and a ruler to facilitate recording activities at home. Study staff will explain to you how to use these. Furthermore, study nurse will contact you by phone one day after immunization to follow up on any adverse events. During the 7 days after vaccination you will also be asked to contact the study site for all medical or adverse events that are considered more than mild.

During subsequent visits after vaccinations you will be requested to show your record of events in the diary card, as well as drugs taken since the last vaccine injection. At these follow-up visits blood will be drawn for safety evaluations. Before and after the MVA vaccinations an ECG will be performed to look for heart problems. At each visit you will also be asked for any medical events or taken medications since the last study visit.

The results of all tests will be available and discussed with you on the next study visit, or two weeks after study discontinuation, or earlier if an abnormal value that impacts your health is found. You will be tested for HIV regularly. You will be counseled about the test and your results. You will also get regular counseling on how to reduce your risk of getting HIV.

In total, during the entire study period of about 17 months, you will be required to visit the clinic about 18 times and you will be requested to donate a total of 1200 milliliters of blood (about two normal blood donations) at different intervals to assess your health as well as the immunological response to immunization. Normally it is quite safe to make a blood donation of 500 milliliters after every 6 months.

The study wants to know about all medical events and medication taken during the study period. If you experience any illness between the regular study visits, which you feel, should be discussed with a medical doctor you are invited to visit the study clinic. These visits will be called unscheduled visits. The study team will be able to perform basic diagnostic test and provide you with basic treatment if needed. Also the study team will find out about the relationship of any medical event to the vaccine. For complicated medical events the study team might refer you to an appropriate health facility, but will also in such cases assist and follow up on your health problem.

What will happen to my blood samples?
Use in this study: The study investigators will use some of your blood to check your health status and monitor any potential side effects you may have.

The study investigators will use some of your blood to see how well your body is creating protection against HIV. These tests are for HIV-related or vaccine-related research only (not to check your health), so the study investigators will not inform you or the clinic of these results. Some of these tests cannot be done at your study site, so some samples will be sent to a central laboratory in Sweden for this purpose.

Risk of genetic tests and Human Leukocyte Antigen (HLA) testing:
Some of the blood drawn from you as part of this study will be used for genetic tests. Some genetic tests can help researchers study how health or illness is passed on to you by your parents or from you to your children.

HLA is a genetic test that will be used to provide the study investigators with information about your immune system. The results of these genetic tests will be used for research purposes only. Neither you nor your doctor will be given the results of tests. The greatest risk associated with genetic testing is to your privacy. It is possible that if others found out information about your genes from this test, it could cause you problems with family members (having a family member learn about a disease that may be passed on in the families or learning who the true parent of a child is). Additionally, HLA testing may determine if you are at an increased risk or severity of certain diseases. If anyone knows that you have this increased risk by learning your HLA status it may be used to discriminate against you and prevent you from getting insurance or a job. The results of your HLA test will be used only in connection with the immune responses to HIV. Your HLA results will only be connected to you by the PIN number and not by your name or other personal information.

<u>Storage and future testing:</u> *HIV vaccine studies are conducted in order to measure how the vaccine causes your body's immune system to respond to the HIV virus. To better understand this, the study investigators want to take blood samples for testing now and for testing in the future. During this study, blood samples will be tested soon after collection. The remaining sample will then be stored also in Sweden and tested again after the vaccinations are completed.*
Any future testing of your stored samples will only be used to learn more about HIV and vaccines. Approval from an ethical review committee must be obtained for studies using your stored samples. Your samples will not be sold or used directly to produce commercial products.

The researchers do not plan to contact you or your regular doctor with any results from these studies done on your stored samples. This is because research test results will not be used to make a decision on how to manage your health.

Your samples and your privacy will be protected because the information you provide and all blood and urine samples will be labeled with four pieces of information: (1) an assigned number that does not have any of your personal information this number is called a Personal Identification Number (PIN); (2) the type of specimen that is stored inside the tube; (3) a study and visit number; and, (4) a visit date. All personal information will be stored by the study investigator at the study site.

Risks associated with participating in this Study
The vaccine candidates that are going to be used in this study have been studied in animals and proved to be safe, and have undergone trials in humans among Swedish, Tanzanian and American volunteers, where it has so far been shown to be safe. Since the candidate vaccines contain only an incomplete part of the HIV genetic material, <u>there is no possibility that you may become HIV-infected by being injected with this vaccine.</u>

However, as with all vaccinations, you may experience local tenderness and swelling at the site of injection, run a low-grade fever, or feel unwell after the immunization. Both you and the study personnel will look very hard for any such unwanted effects of the vaccine. You may also experience discomfort during vaccination, feel dizzy or even faint and may have a bruise or swelling where the needle goes into your arm.

Allergic reaction: As with all injections there is a risk for allergic reactions, like a rash, hives, or even difficulty breathing. Allergic reactions can be life threatening. *No such event has been ascribed to the vaccines so far.*

Blood drawing: Drawing blood may cause pain and bruising. On rare occasions, it may cause bacterial infection at the part of your body where the blood is taken. Sometimes, drawing blood causes people to feel lightheaded or to faint. Some people, especially women, may become anemic (have a low red blood cell count).

Although the safety of this type of vaccination has been largely established, it is important to realize that the experiences with them are still limited, and there may be other unknown effects. It is therefore necessary that you and the study personnel have a close watch over your health.

Since risks related to pregnancy and new born babies are as yet unknown, you must ensure that you practice effective birth control measures in order to avoid being pregnant or impregnating a woman. Effective and adequate sexual protective measures are also necessary so as to avoid the risk of becoming sexually infected with HIV. ***This is especially important since, as mentioned above, we are not as yet sure as to whether this vaccine candidate is protective against HIV or not***. These measures have to go on until 6 months after the last vaccine injection.

Risks of the experimental DNA vaccine:
Possible theoretical side effects of DNA vaccines include: muscle damage; antibodies to DNA leading to illness; and insertion of the vaccine DNA into the body's DNA (leading to cancer) or into the DNA of a bacteria or virus in your body. None of these possible risks of DNA vaccines have been seen in laboratory tests or in animals or humans so far

Based on previous studies, other possible side effects of the experimental DNA vaccine could include dizziness, hives and irritation/scabbing around the injection site. There could also be changes for liver activity, red blood cell count, white blood cell count and urine protein. During this study therefore, regular check-ups including blood and urine tests will be performed to monitor for these possible side effects.

Risks of the MVA vaccine:
The original smallpox vaccine has been seen to cause inflammation in the heart in very few people. This has so far not been seen with MVA, but an electrical tracing of your heart will be done at baseline (known as an ECG). The tracing will be reviewed by a panel of 2 heart experts, who will advise whether or not you can enroll, and whether further investigations are needed.

Possible risks related to HIV exposure
Receiving the study vaccines will not necessarily provide you with any protection against HIV infection, and because you may be receiving placebo, you must not do anything that might expose you to HIV (such as unprotected sex or sharing

needles for injection). HIV infection and AIDS can occur in a person who has received a study vaccine. In past studies, some people who got HIV study vaccine or placebo became infected with HIV through sex or drug use.

If you are exposed to HIV after receiving these study vaccinations your risk of getting infected with HIV and developing AIDS is not known. If you do get infected, the research team also does not know what effect the vaccine may have on the disease. The time that it takes for you to become sick from HIV/AIDS may be the same, or longer, or even shorter than the time that it is expected without the vaccine.

You may have a positive HIV test result

By being injected with the active candidate vaccine, you may test HIV positive in the commonly used laboratory tests for detection of HIV, e.g. ELISA tests. This does not imply that you are infected with HIV but it shows that the immunization was successful. **HOWEVER IT DOES NOT MEAN THAT YOU ARE PROTECTED FROM THE RISK OF BECOMING HIV INFECTED**. At the end of this study you will be issued a card signed by the principal investigators stating that you have participated as a volunteer in a HIV-1 immunization study and that the outcome of standard HIV diagnostic tests does not signify HIV-1 infection. Should you at any time consider yourself to be possibly HIV infected, the project has at its disposal tests that will be able to differentiate actual infection from vaccine induced positive tests, and this will be done on you at no cost. Should you encounter any difficulties on account of this positive HIV test, the investigators will be willing to provide a scientific explanation if and when requested to do so.

In case of HIV infection

During the screening process, if you are found HIV positive, you will be referred to the MNH HIV clinic or the Mbeya Referral Hospital CTC respectively, whereby you will be provided with counselling, drug to prevent bacterial infections, treatment for presenting associated illnesses as well as antiretroviral drugs if indicated.

If you are enrolled in the study as one of the 120 volunteers and become infected with HIV through risky behaviour like unprotected sex during the study, then arrangements will be made so that you will be assured of appropriate care (close follow up and provision of HIV drugs) according to the National guidelines.

Potential Benefits

There may be no direct benefits to you from participating in this research; however, there may be benefits to others. By taking part in this research study, you will have personally contributed to the global and national initiatives to an HIV vaccine development that may eventually combat further spread of HIV/AIDS pandemic in Tanzania and the rest of the world.

Your health will also be thoroughly examined and investigated by experienced medical personnel free of charge. The study site is able to provide you with basic medical care and treatment for common illnesses. However, for major medical problems we would refer you to an appropriate health facility. Altogether, the knowledge to be gained from this study is likely to be of assistance to millions of people in Tanzania and elsewhere.

Criteria for stopping you from further participation in the study

You may be removed from the study without your consent for the following reasons:
1. The investigators decide that continuing in the study would be harmful to you
2. The study governing board and other national and international authorities stop the study.

What if I choose to leave this study?
If you join this study, you can leave it at any time. If you leave this study, you will not lose any benefits or rights you would normally have or be disadvantaged in any way.

If you decide to leave this study, please tell the clinic staff. The study investigators will ask you to come back to the clinic at least once, to check your health and your immune response. The study team would also like to know why you decided to leave the study; however, you do not have to tell reasons. Like everyone else in this study, you will have to wait until all volunteers complete their final study visit to find out whether you got the experimental vaccine or the placebo.

What are my rights and responsibilities as a study volunteer in this research study?
If you join this study, you are responsible for:
(1) Coming to all clinic visits;
(2) Recording your temperature and other side effects on the diary card the evening of the vaccination and for the following 7 days, or longer if necessary;
(3) Informing the clinic about any symptoms or side effects you have, any medications you are taking, any other vaccines such as a flu shot; and if you have moved or want to leave the study;
(4) Following instructions from the clinic;
(5) Staying in touch with the clinic;
(6) Getting your HIV testing done only at the clinic; and
(7) Avoiding pregnancy for at least 6 months after the last study vaccination.

Who makes sure that this study is done correctly?
The National Ethics Committee of the Ministry of Health and Social Services at NIMR, Ethics Committees of the Muhimbili University of Health and Allied Sciences (MUHAS) and NIMR-Mbeya Medical Research Programme (MMRP), the Tanzania Food and Drugs Authority (TFDA)*,* the Karolinska Institute and the Swedish Institute for Infectious Disease Control (SMI) in Sweden, and people who work for these organizations watch over this study to see that your rights are protected and that the researchers are following this study plan an in accordance with the Internationally accepted standards of conducting research.

Confidentiality
The respective Principal Investigators (**Dr Muhammad Bakari** at MUHAS, and **Dr. Leonard Maboko** at NIMR-MMRP) will maintain research records of your taking part in this study.
Your research records will be confidential to the extent permitted by law. You will be identified by a code, and not by name. Personal information from your records will not be released without your written permission. You will not be personally identified in any publication about this study. If necessary, your records may be reviewed by regulatory authorities such as the Tanzania Food and Drugs Authority (TFDA) and National Institutes of Medical Research (NIMR); the study monitors and DSMB (Data Safety and Monitoring Board).

What if the researchers learn new information during this study?
Results of this study or other scientific research may affect your willingness to continue to take part in this study. During the course of the study, you will be informed of any significant new findings (either good or bad), such as changes in the risks or benefits resulting from participation in the research, or new alternatives to participation that might cause you to change your mind about continuing in the study. If new information is provided to you, your consent to continue participating in this study will be re-obtained. If the study investigators learn new information of this kind, the study investigators will share it with you.

Compensation for time and inconvenience
Concurrent with your participation, the project will provide you with a total of Tsh 20.000,- (Dar es Salaam), Tsh 15.000,- (Mbeya) per scheduled study visit to cover for your transport costs to and from the clinic, as well as a compensation for the time accorded to this research. For unscheduled visits your compensation will be Tsh 10.000,- (Dar es Salaam), Tsh 8.000,- (Mbeya). It is hoped though that there will be no misuse of unscheduled visits for monetary benefits.

Research-Related Injury
If you are injured as a result of participation in this study, the study clinic will give you immediate necessary treatment for your injuries. The cost of this treatment will be born by the study. You will then be told where you may receive additional treatment for injuries.

Insurance
As a volunteer in this study, you will be covered with an insurance policy against trial related injuries as well as medical problems in case they do so happen during the study.

Persons to contact in case of problems or questions
If you ever have questions about this study or in case you are injured as a result of participation in this research study, you should contact any of the following:

At MUHAS, Dar es Salaam:

Study Office (TaMoVac) contacts:
Telephone No: 2 15 30 27
Fax No: 2 15 30 27

Postal address: TaMoVac Project
Muhimbili University of Health and Allied Sciences,
P.O. Box 65001,
Dar es Salaam.

Name	Mobile Tel No:	Responsibility
Dr. Muhammad Bakari	0713702211	Principal Investigator
Prof Eligius Lyamuya	0754495933	Co-Principal Investigator
Prof Fred Mhalu	0713227944	Senior Investigator
Dr. Patricia Munseri	0754562784	Clinical Research Coordinator
Dr Mohamed Janabi	0755495270	Senior Investigator

Dr Eric Aris	0754824071	Senior Investigator
Ms Mary Ngatoluwa	0784608841	Principal Study Nurse

At NIMR- MMRP, Mbeya:

Study office (TaMoVac) contacts:
Telephone no: +255 (25) 2503364
Fax No: +255 (25) 2503134

Postal address: NIMR-Mbeya Medical Research Programme (MMRP)
P.O. Box 2410
Mbeya

**Name**	**Mobile Tel No:**	**Responsibility**
Dr. Leonard Maboko	0754 – 645 672	Principal Investigator
Dr. Lucas Maganga	0784 – 514 311	Senior Investigator
Dr. Arne Kroidl	0752 – 880 382	Clinical Research Coordinator
Dr. Bahati Kaluwa	0653 – 221944	Study Medical Officer
Mrs Doreen Pamba	0717 – 339057	Outreach Manager
Mrs. Sekela Mwagobele	0755 – 805 407	Study Secretary

Contact with Ethics Committees
You may also wish to communicate with the ethics committees regarding this study by contacting the following:

At MUHAS, Dar es Salaam:
MUHAS Ethics Committee, Chairman
P.O. Box 65001
Dar es Salaam
Phone: 2150331/2150302, Ext: 272
2152489 – Direct line

At NIMR- MMRP, Mbeya:
Mbeya Medical Research and Ethics Committee
Dr. Eleuter Samky, Mbeya Referral Hospital
P.O. Box 419
Mbeya
Phone: +255-25-2503456 or 2503351
Fax: +255-25-2503577

Chairman
National Ethics Committee
National Institute for Medical Research (NIMR)
Ocean Road,
P.O. Box 9653,
Dar es Salaam
Tel: 2121400
Attention: Ms Joyce Ikingura

CONSENT

I acknowledge to have read the TaMoVac 1 information sheet, August 2010 about this study and all my questions have been adequately answered.

________________________________ __________________

Name and signature of the volunteer Date

____

Study Number

________________________________ __________________

Name and signature of the researcher Date

Appendix 4B: Fomu ya Maelezo Juu ya Utafiti wa TaMoVac Agosti 2010 (Swahili)

MAELEZO JUU YA UTAFITI WA TaMoVac-I KWA AJILI YA MSHIRIKI

"Hatua ya I/II ya utafiti wa chanjo ya majaribio dhidi ya virusi vya ukimwi (VVU) ili kubaini usalama wa chanjo hiyo na uwezo wake wa kujenga vichocheo vya kinga miongoni mwa watakaojitolea kushiriki jijini Dar es Salaam na Mbeya, Tanzania na pia kujenga uwezo wa tafiti za chanjo nchini"

Mpendwa Mshiriki Mtarajiwa,

Tunapenda kukuomba usome kwa makini maelezo kuhusu utafiti wa chanjo ya majaribio dhidi ya VVU na jinsi ya kushiriki.

Utafiti huu unafanywa ikiwa ni sehemu ya Programu ya utafiti ijulikanayo kama TaMoVac-I(Tanzania and Mozambique HIV Vaccine Programme) wenye lengo la kujenga uwezo wa kufanya tafiti za chanjo dhidi ya VVU katika nchi za Tanzania na Msumbiji.

Huu ni utafiti unaoratibiwa na Chuo Kikuu cha Afya na Sayansi Shirikishi cha Muhimbili (MUHAS) kwa kushirikiana na Mradi wa utafiti wa Mbeya-NIMR (NIMR-MMRP), Hospitali ya Taifa Muhimbili hapa Tanzania. Utafiti huu pia unahusisha taasisi za nje ya Tanzania, ambazo ni Taasisi ya Karolinska (KI) na Taasisi ya Udhibiti wa Magonjwa ya kuambukiza, (SMI) za Sweden, Chuo Kikuu cha Munich Ujerumani, na Chuo cha Imperial cha London, Uingereza.

Utafiti huu unahusu chanjo ya majaribio dhidi ya VVU (DNA-MVA) iliyotengenezwa Sweden na Marekani katika jitihada za kupambana na janga la ukimwi. Hii ni awamu ya awali ya utafiti ambayo haihusiki na kupima uwezo wa chanjo kukinga dhidi ya mambukizi ya VVU, na kwa hiyo hakuna uhakika kwamba chanjo hii ina uwezo wa kinga dhidi ya VVU.

Maelezo haya muhimu yanahusu lengo la utafiti, namna ya kushiriki, faida ya kushiriki, yapi yampasayo mshiriki, na madhara yapi yanaweza kumtokea mshiriki wa utafiti huu. Taarifa hizi ni muhimu ili kukuwezesha kufanya maamuzi baada ya kuelewa, juu ya kushiriki au kutokushiriki katika utafiti.

Malengo

Utafiti huu una malengo yafuatayo:

1. Kubaini usalama wa chanjo hii katika mwili wa binadamu
2. Kubaini uwezo wa chanjo kuuwezesha mwili kutengeneza vichocheo vya kinga ambavyo huenda vikatoa kinga dhidi ya mambukizi ya VVU
3. Kubaini idadi ya chini kabisa ya sindano za chanjo za DNA ambazo zitatoa kiwango cha kutosha cha kinga.
4. Kuimarisha zaidi uwezo wa kitaalamu katika kufanya majaribio ya chanjo dhidi ya VVU nchini Tanzania na pia kuongeza ushirikiano kati ya taasisi za Tanzania zinazoshiriki katika utafiti huu ambazo ni MUHAS, NIMR na NIMR-MMRP.

Chanjo
Namna hii ya utoaji wa chanjo hujulikana kitaalamu kama (DNA-MVA Prime boost). Katika chanjo hii, vinyororo vya DNA vimetengenezwa nchini Sweden, na vile vya MVA vimetengenezwa Marekani. Chanjo hii tayari imeshafanyiwa majaribio ya kubaini usalama wake kwa watu 40 waliojitolea kule nchini Sweden na pia kwa washiriki 60 wa utafiti wa chanjo wa mradi was HIVIS uliokamilika hivi karibuni jijini Dar es Salaam na kuonyesha kuwa ni salama na ina uwezo mzuri wa kutengeneza vichocheo vya kinga.

Chanjo ya DNA ni nini?
DNA ni kitu cha asili katika miili ya viumbe hai ambacho kazi yake ni kutoa maelekezo kwa chembe hai ili ziweze kutengeneza protini na malighafi kwa ajili ya chembe hai na mwili kwa ujumla. Chanjo za DNA zina vipande vya DNA kutoka kwa Virusi vya Ukimwi vilivyotengenezwa na binadamu ambavyo mshiriki anapopatiwa mwili wa mshiriki huyo unaweza kuvitambua na kutengeneza kinga dhidi ya Virusi vya Ukimwi. DNA haiwezi kuzaa wala kukua, na kamwe DNA haiwezi kubadilika na kuwa kirusi kinachosababisha Ukimwi.

Chanjo ya MVA ni nini?
Chanjo ya MVA imetengenezwa kutoka kwa kirusi kinachoitwa Modified Vaccinia Ankara (MVA), na hii ilitengenezwa kama chanjo dhidi ya virusi vinavyosababisha ndui. MVA imepunguzwa nguvu ili isiweze kusababisha ndui kwa washiriki na imejaribiwa kwa takribani watu 100,000 bila ya kuonyesha madhara makubwa.
Huyu kirusi wa MVA anabeba protini ipatikanayo kutoka VVU. Mshiriki akipewa MVA yenye protini za VVU mwili wake unaweza kutengeneza kinga dhidi VVU. Ni vema ukafahamu kuwa chanjo ya MVA haiwezi kusababisha maambukizi ya virusi vya Ukimwi. Chanjo hii ya MVA inatolewa ili kuboresha vichocheo vya kinga vilivyotengenezwa na chanjo za awali za DNA. Ingawa chanjo ya ndui yenyewe ilionyesha kusababisha madhara kiasi kwenye mioyo ya watu wachache sana waliopatiwa huko nyuma, madahara haya hayajaonekana katika washiriki wa tafiti zilizopita waliopatiwa chanjo hii ya MVA. Hata hivyo, ili kuwa makini zaidi uchunguzi wa moyo huwa unafanyika ili kuhakikisha usalama wa mshiriki. Majibu ya kipimo hicho cha ECG yataangaliwa na jopo la watalaamu wawili wa magonjwa ya moyo ambao watatoa ushauri kama ni sawa au si sawa kukuingiza kwenye utafiti huu, au kama vipimo zaidi vitahitajika.

Utafiti
Utafiti huu umepata ridhaa kutoka kwa kamati ya maadili mema katika utafiti chini ya Taasisi ya Taifa ya Utafiti wa Magonjwa ya Binadamu (National Institute for Medical Research, NIMR), kamati za maadili za taasisi husika yaani MUHAS-Dar es Salaam na NIMR-MMRP-Mbeya na vile vile chanjo inayotumika imehakikiwa, imesajiliwa rasmi, na kuruhusiwa kutumika miongoni mwa wa-Tanzania na Mamlaka ya Madawa na Chakula ya Tanzania (Tanzania Food and Drugs Authority-TFDA).

Utafiti unahusu watu watakaojitolea kushiriki kwa RIDHAA yao wenyewe, ambao idadi yao ni 120. Kati yao washiriki 60 ni kutoka Dar es Salaam, na washiriki 60 ni kutoka Mbeya. Washiriki hawa wanatakiwa wawe ni wenye afya njema, wasio na maambukizi

ya VVU, na vile vile wawe ni watu ambao ama hawana au wana uwezekano mdogo sana wa kuambukizwa VVU kufuatana na tabia zao.

Kwa kupitia utaratibu wa kubahatisha bila ya anayetoa chanjo au anayepewa chanjo kuelewa wanachopewa (Randomization/mfano wa ”Kutupa shilingi”), washiriki 108 kati ya hao 120 watapatiwa chanjo 3 za awali za DNA katika wiki ya mwanzoni (0), wiki ya 4 na wiki ya 12, ikifuatiwa na chanjo mbili za MVA (kipiga jeki). Chanjo ya kwanza ya MVA itatolewa wiki 18 baada ya chanjo ya tatu ya DNA, na chanjo ya pili ya MVA itatolewa wiki 16 baada ya chanjo ya kwanza ya MVA.
Washiriki 12 waliobaki, bila kutambua, watapatiwa chanjo inayofanana na zile chanjo halisi kwa mwonekano, lakini itakuwa tu na maji ya chumvi yatumikayo katika sindano za kawaida (Placebo) kwa nyakati zile zile zinazofanana na zile za chanjo halisi. Baada ya hapo, washiriki wote watafuatiliwa kwa ukaribu sana kwa takriban wiki 24, Haya yanaonyweshwa vizuri zaidi katika jedwali hili:

**Hudhurio**	**1**	**2**	**3**	**4**	**5**	**6**	**7**	**8**	**9**	**10**	**11**	**12**	**13**	**14**	**15**	**16**	**17**	**18**
**Wiki**	**-4-8**	**-2-4**	**0**	**2**	**4**	**6**	**8**	**12**	**14**	**16**	**30**	**32**	**34**	**46**	**48**	**50**	**58**	**70**
**Maelezo ya hudhurio**	Uchunguzi 1	Uchunguzi 2	Chanjo ya 1 ya DNA	Ufuatiliaji 1	Chanjo ya 2 ya DNA	Ufuatiliaji2	Ufuatiliaji 3	Chanjo ya 3 ya DNA	Ufuatiliaji 4	Ufuatiliaji 5	Chanjo ya 1 ya MVA	Ufuatiliaji 7	Ufuatiliaji 8	Chanjo ya 2 ya MVA	Ufuatiliaji 9	Ufuatiliaji 10	Ufuatiliaji 11	Ufuatiliaji 12

Namna ya Kushiriki
Kufuatia majadiliano na kupatiwa maelezo ya kina kuhusu utafiti huu, na pia baada ya kwamba maswali yako yamejibiwa kiunaga-ubaga, kufaulu ”mtihani” wa uelewa wa chanjo za majaribio za VVU, na una NIA thabiti ya kuwa mshiriki katika utafiti huu, ni lazima pia uwe na sifa nyingine muhimu zikiwemo:

1. Umri kati ya miaka 18 na 40,
2. Kutokuwa na magojwa yafuatayo Hepatitis, VVU na kaswende,
3. Uwezekano mdogo wa kupata maambukizi ya VVU,
4. Kutokuwa na mimba wala kunyonyesha, na
5. Kuwa tayari kutolewa sampuli za damu kwa ajili ya utafiti.

Sifa hizo zikitimia,utaombwa kuweka sahihi yako katika fomu maalum ya kuthibitisha utayari wako wa kushiriki. Nakala ya fomu hii utapewa mwenyewe kwa ajili ya kumbukumbu zako. Ni vizuri kukumbuka kwamba tutaomba kukutembelea nyumbani kwako au kukupigia simu kwa ajili ya kukumbushia kuhusu hudhurio la kuja kliniki ya utafiti. Vile vile, utapigwa picha kwa ajili ya kitambulisho pindi utakapojiunga na utafiti na utapaswa kuja na kitambulisho chako katika kila hudhurio.

Katika hudhurio la mwanzo utafanyiwa usaili kuhusu afya yako, dawa ambazo unatumia, pamoja na historia yako inayohusu mapenzi na kujamiiana. Baadaye utapimwa mwili na Daktari na vipimo kadhaa kuchukuliwa ili kuhakikisha kwamba u mwenye afya njema. Kiasi cha damu kitakachochukuliwa kwa ajili ya vipimo ni mililita 9 (kama vijiko vikubwa 2 vya kulia chakula). Hiki ni kiasi kidogo tu cha damu ikilinganishwa na zaidi ya mililita 5000 (Lita 5) zilizoko katika mwili wa binadamu.

Katika damu vipimo vya magonjwa yafuatayo vitafanywa, Virusi vya Ukimwi (HIV) , Kaswende(Syphilis) na Virusi vinavyoshambulia ini (Hepatitis). Kipimo cha moyo (ECG) pia kitafanyika. Utaombwa kutoa mkojo kwa ajili ya kufanya vipimo vya kuangalia hali za figo na ini; na kwa kina mama kipimo cha mimba kitafanyika. Hudhurio hili litachukua takriban masaa 2 hadi 3.

Katika hudhurio la pili, wiki 2 baadaye, utapewa majibu ya vipimo vilivyochukuliwa awali, na baadaye kuwa na mjadala kuhusu utafiti huu kwa mara nyingine tena. Iwapo bado utapenda kushiriki, na vipimo vya kitaalamu vitaruhusu, utasaini fomu ya pili ya utayari wako wa kushiriki na kuruhusu watafiti kuchukua vipimo vya damu takribani millilita 120 (vijiko 14 vya kulia chakula) ili kupima vichocheo vya kinga mwilini. Hudhurio hili litachukua takriban saa 1.

Katika hudhurio la tatu, wiki 2 baada ya hudhurio la 2, utaulizwa tena kuhusu ukubali wako wa kushiriki katika utafiti huu. Iwapo utakuwa bado unapenda kushiriki, utapewa kitambulisho chako chenye picha na kwa kupitia utaratibu wa kubahatisha, utasajiliwa katika mojawapo ya makundi matatu ya kupatiwa chanjo.

Kundi la IA/IIA:
Watapatiwa sindano mbili (2) za chanjo ya DNA chini ya ngozi ya mkono ikifuatiwa na MVA katika musuli wa mkono. Tofauti kati IA na IIA ni mchanganyiko wa chanjo ya DNA.

Kundi la IIIA:
Watapatiwa sindano tano(5) za chanjo ya DNA chini ya ngozi ya mkono,
ikifuatiwa na chanjo ya MVA kwenye musuli wa mkono, hii ni kama ilivyokuwa katika tafiti zilizopita.

Kundi la IB/IIB:
Watapatiwa sindano mbili za chanjo yenye maji ya chumvi ya sindano chini ya ngozi na kwenye musuli wa mkono.

Kundi la IIIB:
Watapatiwa sindano tano za chanjo yenye maji ya chumvi ya chini ya ngozi ya mkono na kwenye msuli wa mkono.

Inafahamika kwamba maji ya chumvi ni salama na kwamba mwili hauzalishi vichocheo vya kinga dhidi yake. Kwa sababu hiyo maji ya chumvi yatatumika katika utafiti huu kwa ajili ya kulinganisha usalama na uwezo wa kuchochea mwili kuzalisha vichocheo vya kinga kati yake na chanjo za majaribio. Maji ya chumvi yatatengenezwa katika mwonekano utakaofanana na chanjo zitakazotumika katika utafiti huu, na hakuna mtu ambaye ataweza kutofautisha kwa kuangalia tu. Tunajua kwamba ufuatiliaji thabiti katika utafiti huu waweza kusababisha watu kutoa taarifa za dalili zisizohusiana na chanjo hizi, hivyo basi, baadhi ya washiriki watapewa maji ya chumvi ili kutuwezesha kujua kwa uhakika ni dalili zipi zinasababishwa na chanjo.

Ni muhimu kufahamu kwamba wewe kama mshiriki au daktari au muuguzi anayekuhudumia hamtakuwa na uwezo wa kuchagua uwe kwenye kundi lipi mojawapo. Vile vile watafiti na wewe mwenyewe hamtafahamu uko kwenye kundi lipi hadi mwisho wa utafiti, au iwapo itatokea dharura itakayolazimu hilo lijulikane.

Kabla ya kupewa chanjo watafiti watahakikisha kwamba upo katika hali ya afya njema na utapimwa kuona hali ya maambukizi ya VVU na utaombwa kutoa damu na mkojo kwa ajili ya vipimo kabla ya chanjo. Washiriki wote wa jinsia ya kike watafanyiwa kipimo cha kuangalia hali ya mimba. Mshiriki mwenye ujauzito (Mimba) hatapatiwa chanjo.

Utoaji wa chanjo ya DNA kwenye ama musuli wa mkono au chini ya ngozi utafanyika kwa kutumia kifaa maalum kisichotumia sindano kiitwacho “Bioject”. Chanjo ya MVA itatolewa kwa kutumia sindano ya kawaida. Kabla ya kuchanjwa utatolewa tena damu kwa ajili ya kubaini vichocheo vya kinga na usalama na kisha kupatiwa chanjo au maji ya chumvi. Mara tu baada ya kila chanjo utatakiwa kuwepo kliniki kwa muda usiopungua nusu saa kwa ajili ya kuwa na uangalizi wa karibu. Hudhurio litakalohusisha chanjo litatumia muda wa saa moja na nusu.

Katika mahudhurio yatakayofuata utatakiwa kutupa taarifa zinazohusu afya yako , pamoja na dawa zozote ambazo utakuwa umezitumia tangu upate chanjo ya mwisho katika kitabu cha kumbukumbu (diary) utakachopewa. Hivyo utapatiwa faili litakalokuwa na diary, kipima joto na rula ili kufanikisha zoezi hili la kuweka kumbukumbu uwapo nyumbani. Muuguzi wa utafiti atakupigia simu siku moja baada ya chanjo kujua hali yako na pia utaombwa kuwasiliana na watafiti iwapo utapata hali ya kushtusha zaidi ya kiwango cha 1 (tafadhali angalia jedwali).

Katika mahudurio yatakayofuata baada ya chanjo, mshiriki anatakiwa kutoa taarifa za matukio yote ya kiafya na kumbukumbu ya dawa alizotumia katika kipindi chote hicho kama ilivyoandikwa kwenye kitabu cha kumbukumbu. Katika mahudhurio haya mshiriki atatoa damu kwa ajili ya vipimo vya kinga na usalama. Vile vile, kabla na baada ya chanjo ya MVA mshiriki atafanyiwa kipimo cha moyo (ECG).

Matokeo ya vipimo vya utafiti yatakuwa tayari kwa majadiliano katika hudhurio linalofuata au wiki mbili baada ya kuacha utafiti au mapema zaidi kama kuna kipimo ambacho kimeonyesha matatizo. Utapimwa hali ya maambukizi ya VVU mara kwa mara na pia pia utaptiwa ushauri kwa ajili ya kujikinga na VVU mara kwa mara.

Kwa ujumla katika kipindi chote cha utafiti kitakachokuwa takribani miezi 17 utatakiwa kufanya mahudhurio yapatayo 18 katika kliniki ya utafiti huu na utatakiwa kutoa damu takriban mililita 1,200 (kama mara mbili ya kiwango cha kawaida ambacho watu hujitolea damu mahospitalini kwa mara moja) kwa ajili ya vipimo katika nyakati tofauti ili kufuatilia afya yako, na vile vile kufuatilia uwezo wa mwili wako kutengeneza vichocheo vya kinga kufuatia chanjo hii ya majaribio. Kwa kawaida ni salama kabisa kwa mtu kujitolea damu kiwango cha mililita 500 kila baada ya miezi sita.

Tutapenda kujua matukio yote ya afya yanayokupata, kwa hiyo mshiriki anakaribishwa kuhudhuria kliniki wakati wowote anapokuwa na tatizo la kiafya kwa ajili ya uchunguzi na itakaposhindikana msaada wa uchunguzi utaombwa katika hospitali na taasisi nyingine ukisaidiwa na watafiti.

Damu yako itafanyiwa nini?

Katika utafiti huu: Watafiti watatumia damu kuchunguza hali yako ya afya na pia kuangalia vichocheo vya kinga. Vipimo vingine ambavyo ni vigumu kuvifanya katika

mazingira yetu vitafanywa katika maabara za Sweden. Mshiriki hatafahamishwa kuhusu matokeo ya vichocheo vya kinga.

Athari za vipimo vya jenetiki/vinasaba na HLA (Human Leucocytes Antigen)
Kiasi fulani cha damu yako kitafanyiwa vipimo vya jenetiki. Vipimo hivi vya jenetiki vina lengo la kuwasaidia watafiti kushabihisha jinsi maambukizi ya ugonjwa/maradhi ya kurithi kutoka kwa wazazi wako au kutoka kwako kwenda kwa watoto wako.
HLA ni kipimo cha jenetiki ambacho kitatumika kuwawezesha watafiti kupata habari za mfumo wako wa kinga. Matokeo ya ya vipimo vya jenetiki yatatumika kwa shughuli za utafiti tu.

Wewe wala daktari wako hamtapewa majibu ya vipimo hivi.

Tatizo ambalo linaweza kutokea ni usiri wa matokeo ya vipimo hivi vya jenetiki. Kama watu wengine wakijua kuhusu jenetiki yako inaweza kukuletea madhara na familia yako kwani wataweza kutambua kama una ugonjwa wa kurithi ambao wao wanaweza kuupata, au wewe kugundua kwamba mzazi wako alikuambukiza ugonjwa huo. Vile vile kipimo hiki cha HLA kinaweza kuonyesha kama upo katika uwezekano mkubwa wa kupata madhara zaidi ya magonjwa fulani na hii inaweza kukuletea madhara kwa mwajiri au katika bima ya afya.
Katika utafiti huu kipimo cha HLA kitatumika kuangalia uhusiano na utengenezaji wa kinga dhidi ya VVU. Matokeo ya kipimo hiki yatatambuliwa na namba ya utambulisho na si kwa jina au kitambulisho kingine ambacho kitawawezesha watu wengine kutambua ni kipimo cha nani.

Uhifadhi kwa ajili ya baadaye: Sehemu ya damu utakayotoa itahifadhiwa kwa ajili ya kufanya vipimo vingine siku za mbeleni. Kibali kwa ajili ya kutumia damu iliyohifadhiwa vitaombwa kutoka kwa kamati za maadili. Damu itakayohifadhiwa haitatumiwa kwa shughuli za kibiashara.

Vipimo vyako vitapewa namba za siri (Personal Identification Number au PIN).

Kumbukumbu zote zitahifadhiwa katika vituo vya utafiti.

Madhara yawezayo kupatikana kwa ushiriki katika utafiti huu
Chanjo hizi zinazofanyiwa utafiti zimefanyiwa majaribio kwa wanyama na kuthibitika kwamba ni salama. Kwa vile chanjo hii inahusu tu sehemu ndogo ya jenetiki ya chembe uhai ya VVU, **hakuna uwezekano kabisa wa mtu kuambukizwa VVU kutokana na kupatiwa chanjo hii**. Hata hivyo, kama ambavyo hutokea kwa chanjo zingine, unaweza kupata maumivu kidogo, uvimbe kidogo au wekundu kiasi katika sehemu utakayochomwa chanjo hii. Yawezekana pia ukapata homa kiasi au kujisikia mchovu kwa siku moja au mbili baada ya chanjo. Pia utasikia maumivu wakati wa kutolewa vipimo vya damu, na kwa baadhi ya watu waweza kupata kizunguzungu au hata kuzimia wakati huo wa kutolewa damu.

Ingawa majaribio ya chanjo za jamii hii yamethibitishwa usalama wake, na kwamba chanjo hii nayo imethibitika kuwa salama kwa wanyama na haijaonyesha madhara yoyote na inavumilika vizuri miongoni mwa watu waliojitolea kule Sweden Marekani na katika jaribio liliofanyika hapa Tanzania, ni muhimu kufahamu kuwa bado uzoefu na

chanjo hii si mkubwa sana, na yawezekana kukawepo na athari zingine. Kwa hiyo ni muhimu wewe mwenyewe kwa kushirikiana na watafiti kuwa makini katika kuchunguza afya yako. Tunafahamu kwamba kwa umakini huu wa ziada, kuna uwezekano wa kutajwa dalili nyingi zaidi, na hivyo ni muhimu kuwa na kundi la ulinganishi ambalo litapatiwa tu maji ya chumvi ya kawaida.

Kwa vile madhara yawezayo kutokea kutokana na chanjo hii kwa mama mjazito na mtoto mchanga tumboni hayafahamiki kwa sasa, ni muhimu sana kuhakikisha kwamba unatumia njia za kujikinga kupata mimba au kumpa mimba mwanamke yeyote kwa kipindi chote cha utafiti na miezi minne baada ya kupatiwa chanjo ya mwisho.

Vile vile ni muhimu sana kuhakikisha kwamba unatumia njia za kuzuia kupata maambukizi ya VVU kwa njia ya kujamiiana, ikiwemo kutumia kondom wakati wa tendo la ndoa nje ya ndoa yako. Hii ni muhimu sana, kwa vile kama ilivyoelezwa hapo juu, hakuna uhakika kwa sasa kama chanjo hii ina uwezo wa kutoa kinga ya kutosha dhidi ya VVU au la.

Madhara dhidi ya VVU
Ni muhimu sana ukafahamu kuwa kwa kupatiwa chanjo hii dhidi ya VVU si lazima upate kinga dhidi ya maambukizi ya VVU, na pia kwa kuwa mshiriki hatotambua yupo katika kundi gani, la chanjo halisi au la maji ya chumvi chumvi, mshiriki unashauriwa kutoshiriki katika mambo ambayo yanaweza kukusababishia maambukizi ya VVU (Kama kufanya ngono bila kinga, au kushirikiana sindano na vitu vyenye ncha kali).

Maambukizi ya VVU na Ukimwi vinaweza vikampata mshiriki wa utafiti, kwani katika tafiti zilizopita washiriki wa utafiti wa chanjo walipata maambukizi kutokana na ngono au kupitia matumizi ya madawa ya kulevya kwa njia ya sindano.

Kama utajihusisha na vitendo ambavyo vinaweza kukusababishia maambukizi baada ya kupata chanjo uwezekano wa wewe kupata maambukizi na kasi ya kupata Ukimwi havijulikani.

Yawezekana vipimo vya VVU vikaonyesha Positive"
Kwa vile unaweza kupata chanjo halisi, inawezekana vipimo vya kawaida vya kugundua kuwepo maambukizi ya VVU kwa mfano ELISA test ikawa "positive". Hii haitakuwa na maana kwamba umepata maambukizi ya VVU. Itamaanisha tu kwamba chanjo uliyopewa imetambulika na mwili wako na hivyo kutengeneza vichocheo vya kinga (antibodies). **NI MUHIMU HATA HIVYO KUTAMBUA KWAMBA HII HAIMAANISHI KWAMBA SASA UMEPATA KINGA MADHUBUTI DHIDI YA MAAMBUKIZI YA VVU**.
Itakavyotokea pia ni kwamba mwishoni au wakati wa utafiti huu utapewa kadi maalum itakayosainiwa na mkuu wa utafiti itakayokutambulisha kwamba wewe ulikuwa ni mshiriki wa utafiti wa chanjo dhidi ya VVU na hivyo vipimo vya kawaida havimaanishi maambukizi ya VVU.
Hata hivyo, wakati wowote utakapokuwa na wasiwasi kwamba inawezekana umepata maambukizi halisi ya VVU, kwa mfano baada ya kujamiiana na mwenza ambaye ana VVU, basi utafiti huu utakufanyia vipimo ambavyo vina uwezo wa kubaini maambukizi halisi bila ya gharama yoyote.
Vile vile, iwapo patatokea utatanishi kuhusu vipimo hivyo, wataalamu wa utafiti huu watakuwa tayari kutoa maelezo iwapo wataombwa na kutakiwa kufanya hivyo.

Iwapo utagundulika kuwa na maambukizi ya VVU

Wale wote ambao wakati wa uchunguzi wa mwanzo wa kubaini kama mtu ana maambukizi ya VVU au la watakutwa tayari wameshapata maambukizi hayo watapatiwa huduma katika kliniki ya HIV pale Hospitali ya Taifa Muhimbili (kwa washiriki wa Dar es Salaam), au Hospitali ya Rufaa ya Mbeya (kwa washiriki wa Mbeya). Huduma zitakazotolewa pale ni pamoja na ushauri nasaha, dawa ya kinga dhidi ya maambukizi ya bacteria (k.m. Septrin), tiba ya magonjwa nyemelezi, pamoja na dawa za kudhibiti mazaliano ya VVU (ART) kwa wale wataokuwa wamefikia kuzihitaji baada ya kufanyiwa vipimo.

Iwapo utakuwa ni miongoni mwa washiriki 120 wa utafiti huu na kwa bahati mbaya ikathibitika kwamba umepata maambukizi ya VVU kwa njia ya ngono isiyo salama, mradi huu wa utafiti utakufanyia utaratibu wa kuhakikisha kwamba unaendelea kupatiwa matibabu yanayostahili (k.m. uangalizi wa karibu na kupatiwa ARV's) kwa kufuata taratibu za kitaifa.

Manufaa ya Ushiriki
Yawezekana usipate faida yoyote ya moja kwa moja kwa wewe kushiriki katika utafiti huu, lakini kushiriki kwako kwaweza kuwa na faida kwa wengine. Iwapo utashiriki utafiti huu, itakuwa umetoa mchango binafsi wa hali ya juu katika juhudi za kitaifa na kimataifa zenye lengo la kutafuta chanjo bora kwa ajili ya kudhibiti maambukizi ya VVU.

Vile vile, afya yako itachunguzwa kwa undani zaidi na hilo litafanywa na madaktari wataalam bila ya gharama yoyote kwako.

Kwa ujumla, ufahamu utakaopatikana kutokana na utafiti huu utakuwa ni wa manufaa makubwa kwa siku za baadaye.

Sababu zinazoweza kusitisha ushiriki wako katika utafiti
Ushiriki wako katika utafiti huu utasitishwa, hata bila ya ridhaa yako iwapo:
1. Watafiti wataamua kwamba kuendelea kwako kushiriki katika utafiti kutasababisha madhara kwako binafsi au kwa utafiti huu.
2. Bodi ya usimamizi wa utafiti au mamlaka zingine za kitaifa au kimataifa zikiamua kusitisha mradi kuendelea

Je kama nikiamua kuacha kushiriki katika utafiti?
Ukijiunga na utafiti huu unaweza kuacha muda wowote, na ikitokea ukaacha kushiriki hutapoteza faida na haki zako kwa maana ya kupata huduma katika Hospitali za Muhimbili au Mbeya.

Kama utaamua kuacha ushiriki katika utafiti huu, tafadhali wafahamishe watafiti katika kliniki ya utafiti. Watafiti watakuomba uhudhurie kliniki kama watakavyokupangia kwa ajili ya vipimo vya kuangalia kinga na pia usalama.

Watafiti pia watapenda kujua sababu zilizokufanya uamue kuacha ushiriki.lakini pia ni uamuzi wako kusema sababu au kutosema.

Kama itakavyokuwa kwa washiriki wengine wote, itakubidi usubiri mpaka mwisho wa utafiti ili kujua kama ulikuwa katika kundi la chanjo au maji ya chumvi chumvi.

Nini haki zangu na majukumu yangu kama mshiriki wa utafiti huu

Kama ukijiunga na utafiti huu utatakiwa:

1. Kuhudhuria mahudhurio yote uliyopangiwa
2. Kupima jotoridi na kurekodi matukio yote ya kiafya kwenye karatasi ya kumbukumbu siku saba baada ya chanjo au zaidi
3. Kuitaarifu kliniki ya utafiti matukio yote ya kiafya, kumbukumbu za tiba na madawa unayotumia vile vile kama kuna chanjo nyingine unazitumia kama bado ni mshiriki na hata ukiacha ushiriki.
4. Kufuata maagizo ya unayopewa katika kliniki
5. Kuwa na mawasiliano na kliniki
6. Kufanya vipimo na kupata majibu ya VVU katika kliniki yetu ya utafiti
7. Kujiepusha na mimba katika kipindi cha miezi sita baada ya chanjo ya mwisho.

Usimamizi wa utafiti

Kamati ya Taifa ya maadili ya utafiti ya wizara ya afya Tanzania (NIMR), kamati ya maadili ya utafiti ya chuo kikuu cha Afya na Sayansi Shirikishi (MUHAS) na kamati ya maadili ya utafiti pale Mbeya.
Pia utafiti huu unasimamiwa na mamlaka ya chakula na madawa Tanzania(TFDA), Taasisi ya Karolinska(KI) na Taasisi ya magonjwa ya kuambukiza ya Sweden (SMI). Watu wanaofanya kazi na hizi taasisi wanaangalia kuona utafiti unafanyika katika taratibu zilizokubalika kimataifa.

Usiri

Watafiti wakuu wa utafiti huu (Dr Muhammad Bakari-MUHAS na Dr Leonard Maboko-NIMR-MMRP) watatunza kumbukumbu zenu za utafiti; na pia taarifa zote zinazokuhusu ambazo zitapatikana katika utafiti huu zitakuwa siri kwa kiwango kinachokubalika kisheria. Utambulisho wako itakuwa ni kwa namba maalum bila jina lako, na taarifa zako binafsi hazitatolewa bila ya idhini yako. Maandiko yeyote yatakayotokana na utafiti huu hayatakutambua ki-binafsi. Kama ikibidi, taarifa zako zaweza kupitiwa na Mamlaka ya Chakula na Madawa ya Taifa (TFDA), Taasisi ya Taifa ya Utafiti wa Magonjwa ya binadamu (NIMR), Kamati ya Utafiti ya MUHAS, pamoja na Mfuatiliaji wa utafiti (Study monitor).

Matokeo mapya kuhusu tafiti za chanjo

Utaarifiwa matokeo ya utafiti huu na nyinginezo ambazo zinahusiana na chanjo za majaribio ya VVU.

Malipo

Kama mshiriki, utapewa kiasi kidogo cha fedha (Shilingi za kitanzania 20,000/= kwa washiriki wa Dar es Salaam na Shilingi za kitanzania 15,000/= kwa washiriki wa Mbeya) kwa kila hudhurio lililopangwa kwa madhumuni ya kugharimia usafiri wako wa kwenda na kurudi katika kliniki ya utafiti na vile vile kufidia muda wako utakaoutumia kwa ajili ya mahudhurio hayo. Kwa mahudhurio yasiyokuwa rasmi mshirirki atapewa (shilingi 10,000/= kwa washiriki wa Dar es Salaam na shilingi 8,000/= kwa washiriki wa Mbeya). Ni matumaini yetu kwamba mahudhurio yasiyo rasmi yatatumiwa vizuri na si kwa misingi ya kupata fedha.

Maumivu /Madhara yatokanayo na utafiti

Iwapo utapata maumivu/madhara yatokanayo na ushiriki wako katika utafiti huu, Kliniki ya utafiti itahakikisha unapatiwa tiba haraka iwezekanavyo na gharama zote za matibabu zitabebwa na utafiti. Utafahamishwa pia wakati gani utahitaji matibabu zaidi ya madhara hayo.

Bima
Kama mshiriki wa utafiti hii, utapatiwa bima dhidi ya ajali inayohusiana na utafiti huu, pamoja na bima itakayohusu matibabu ya madhara yawezayo kutokea wakati wa ushiriki wako

Watu wa kufanya nao mawasiliano iwapo una tatizo au maswali zaidi
Endapo itatokea kwamba una maswali yanayohitaji maelezo zaidi, au iwapo utapata madhara yatokanayo na kupatiwa chanjo hii ya majaribio, unashauriwa kufanya mawasiliano na yeyote kati ya hawa wafuatao:

MUHAS, Dar es Salaam:

Ofisi ya Utafiti (TaMoVac):
Simu ya mezani : +255222 15 30 27
Faksi : +255222 15 30 27

Anuani ya Posta: TaMoVac Project
Muhimbili University of Health and Allied Sciences,
P.O. Box 65001,
Dar es Salaam.

**Jina**	**Simu za Kiganjani**	**Majukumu**
Dr. Muhammad Bakari	0713702211	Mtafiti Mkuu
Prof Eligius Lyamuya	0754495933	Mtafiti mkuu Mwenza
Prof Fred Mhalu	0713227944	Mtafiti Mwandamizi
Dr. Patricia Munseri	0754562784	Mratibu wa Utafiti
Dr Mohamed Janabi	0755495270	Mtafiti Mwandamizi
Dr Eric Aris	0754824071	Mtafiti Mwandamizi
Ms Mary Ngatoluwa	0784608841	Muuguzi Mkuu

NIMR- MMRP, Mbeya:
Ofisi ya utafiti (TaMoVac):
Simu ya Mezani: +255 (25) 2503364
Faksi: +255 (25) 2503134

Anuani ya Posta: NIMR-Mbeya Medical Research Programme (MMRP)
P.O. Box 2410
Mbeya

**Jina**	**Simu y Kiganjani**	**Majukumu**
Dr. Leonard Maboko	0754 – 645 672	Mtafiti Mkuu
Dr. Lucas Maganga	0784 – 514 311	Mtafiti Mwandamizi
Dr. Arne Kroidl	0752 – 880 382	Mratibu wa Utafiti
Dr. Bahati Kaluwa	0653 – 221944	Daktari wa Utafiti
Mrs Doreen Pamba	0717 – 339057	Mratibu wa shughuli za jamii
Mrs. Sekela Mwagobele	0755 – 805 407	Karani wa Utafiti

Mawasiliano na Kamati za Maadili Mema ya Utafiti
Vile vile kama mshiriki waweza kuwasiliana na kupata taarifa zaidi kuhusu utafiti huu na wafuatao:

Mwenyekiti
MUHAS Ethics Committee
P.O. Box 65001
Dar es Salaam
Simu: 2150331/2150302 Ext: 272
2152489 – Direct Line

Mwenyekiti
Mbeya Medical Research and Ethics Committee
P.O. Box 419
Mbeya
Simu: +255-25-2503456 or 2503351
Faksi: +255-25-2503577
Mhusika:Dr. Eleuter Samky, Mbeya Referral Hospital

Mwenyekiti
Kamati ya Maadili ya Utafiti
Taasisi ya Utafiti wa Magonjwa ya Binadamu
Mtaa wa Ocean Road
S.L.P. 9653,
Dar es Salaam
Simu: 2121400
Mhusika: Bibi Joyce Ikingura

TAMKO LA UTHIBITISHO WA KUPATIWA MAELEZO YA KUTOSHA KUHUSU UTAFITI

Ninathibitisha kwamba nimesoma makala ya taarifa zinazouhusu utafiti huu na nimepata majibu ya kutosholeza kwa maswali yangu yote niliyokuwa nayo kuhusu utafiti huu. Nimeamua kwa hiari yangu mwenyewe kushiriki katika utafiti huu.

_______________________________ _________________
Jina na sahihi ya Mshiriki Tarehe

____
Namba ya utafiti

_______________________________ _________________
Jina na sahihi ya Mtafiti Tarehe

Appendix 4C: TaMoVac I Amendment 1 Information Sheet, August 2010 (English)

Dear study participant,

We would like to inform you that we have introduced some changes in the visit and vaccination schedule of the TaMoVac I study in which you are participating. These changes include:

1. A shortening of the gaps between some vaccinations
2. A shortening of the total follow up time

The main reason for making these changes is that it is desirable for individuals to complete any vaccination schedule as quickly as possible, to keep the time without immunity to a minimum.

1. Shortening the gaps between some vaccinations

You will still receive five vaccinations (3 DNA vaccinations and 2 MVA vaccinations). However, the gap between the 3rd DNA and 1st MVA vaccination, and the gap between the 1st and 2nd MVA will be shortened as followed:

	1st Vaccination (DNA I)	2nd Vaccination (DNA II)	3rd Vaccination (DNA III)	4th Vaccination (MVA I)	5th Vaccination (MVA II)
Old schedule	Week 0	Week 4	Week 12	Week 36	Week 60
**New Schedule**	Week 0	Week 4	Week 12	**Week 30**	**Week 46**

2. Shortening of total follow up time

Each vaccination will still be followed by a safety visit 2 and 4 weeks later. The interim visit between the last DNA safety visit and the 1st MVA vaccination visit, planned to maintain contact, is no longer needed in the shorter schedule, so the total number of visits will be reduced from 19 visits to 18 visits. The last two safety visits in the new schedule will be 58 and 70 weeks from enrolment, instead of 84 and 108 weeks respectively. Therefore the total duration you will be followed for is 38 weeks less than the old schedule.

**Old Visit Schedule**																			
Visits	1	2	3	4	5	6	7	8	9	10	11	12	13	14	15	16	17	18	19
Visit weeks	-4-8	-2-4	0	2	4	6	8	12	14	16	32	36	38	40	60	62	64	84	108
**New Visit Schedule**											**Interim visit not required**								
Visits	1	2	3	4	5	6	7	8	9	10		**11**	**12**	**13**	**14**	**15**	**16**	**17**	**18**
Visit weeks	-4-8	-2-4	0	2	4	6	8	12	14	16		**30**	**32**	**34**	**46**	**48**	**50**	**58**	**70**
**Visit description**	Screening 1	Screening 2	Enrolment and DNA 1	Follow-up 1	DNA 2	Follow-up 2	Follow-up 3	DNA 3	Follow-up 4	Follow-up 5		MVA 1	Follow-up 7	Follow-up 8	MVA 2	Follow-up 9	Follow-up 10	Follow-up 11	Follow-up 12

Besides these changes all procedures and requirements as explained to you at the beginning of the study will remain the same. Your study team is happy to explain and answer further questions.

CONSENT

I acknowledge having read and understood the Amendment 1 information sheet, July 2010, about this study and all my questions have been adequately answered.

I am still willing to continue with study procedures: ☐ yes ☐ no

______________________________ ________________
Name and signature of the volunteer Date

____
Study Number

______________________________ ________________
Name and signature of the researcher Date

Appendix 4D: TaMoVac I Amendment 1 Information Sheet, August 2010 (Swahili)
Dibaji: Marekebisho ya kwanza ya fomu ya maelezo kuhushu utafiti wa TaMoVac 1, Agosti 2010

Mpendwa mshiriki
Tunapenda kukutaarifu kuwa imebidi tufanye mabadiliko ya ratiba ya mahudhurio na utolewaji wa chanjo katika utafiti wa TaMoVac 1 ambapo wewe ni mshiriki.
Mabadiliko haya ni kama ifuatavyo:

1. Kufupisha muda wa kipindi kati ya chanjo moja na nyingine.
2. Kufupisha muda wa ufuatiliaji kwa ujumla.

Mabailiko haya yatasabisha muda mzima wa utafiti upungue.
Sababu kuu ya kufupisha muda wa utafiti huu ni kwamba watafiti wameona ya kuwa ni muhimu washiriki wakamilishe utaratibu wao wa chanjo mapema iwezekanavyo ili kufupisha kipindi ambacho mshirki atakuwa bila ya vichocheo vya kinga.

1. Kufupisha muda wa kipindi kati ya chanjo moja na nyingine.

Awali ulikuwa umetarifiwa kuwa utapokea chanjo tano (chanjo 3 za DNA na 2 za MVA). Idadi ya chanjo hizi itabaki kama hapo awali. Lakini, muda kati ya chanjo ya 3 ya DNA na chanjo ya kwanza ya MVA na ule kati ya chanjo ya kwanza ya MVA na ya pili ya MVA umefupishwa kama ilivyoainishwa kwenye jedwali hapo chini:

	Chanjo ya I<br><br>(DNA I)	Chanjo ya II<br><br>(DNA II)	Chanjo ya III<br>(DNA III)	Chanjo ya IV<br>(MVA I)	Chanjo ya V<br><br>(MVA II)
Ratiba ya awali	Wiki 0	Wiki 4	Wiki 12	Wiki 36	Wiki 60
**Ratiba mpya**	Wiki 0	Wiki 4	Wiki 12	**Wiki 30**	**Wiki 46**

2. Kufupisha muda wa ufuatiliaji kati ya mahudhurio.

Mahudhurio ya ufuatiliaji wa usalama wa chanjo ni kila baada ya wiki 2 na 4 baada ya kupatiwa chanjo. Mahudhuriio hayo yatabaki kama hapo awali. Lakini, badala ya jumla ya mahudhurio 19 kwenye ratiba ya awali sasa kutakuwa na mahudhurio 18 tu kwenye ratiba mpya. Hudhurio la 11, ambalo lilikuwa ni la ufuatiliaji wiki 4 kabla ya chanjo ya 4 (MVA 1) limefutwa. Lengo la hudhurio hili ni kuwasiliana nawe ili kufuatiliia kama kuna matatizo yeyote, maana muda kati ya chanjo ya 3 na ya 4 ulikuwa mrefu mno. Kwa vile kipindi kati ya chanjo ya 3 na ya 4 kimefupishwa tumeona ya kuwa hudhurio hili halitahitajika tena. Pia yale mahudhurio ya muda mrefu ya meizi 6 na 12 baada ya kukamilisha chanjo ya mwisho kama yalivyoainishwa kwenye ratiba ya awali yatafanywa meizi 3 na 6 baada ya chanjo ya mwisho kwenye ratiba mpya. Hivyo jumla ya wiki za ufuatiliaji itakuwa ni wiki 38 pugungufu ukilinganisha na utaratibu wa awali.

**Ratiba ya mahudhurio ya awali**																			
Hudhurio	1	2	3	4	5	6	7	8	9	10	11	12	13	14	15	16	17	18	19
Wiki za Hudhurio	-4-8	-2-4	0	2	4	6	8	12	14	16	32	36	38	40	60	62	64	84	108
**Ratiba mpya ya mahudhurio**											**Hudhurio lilifutwa**								
Hudhurio	1	2	3	4	5	6	7	8	9	10		**11**	**12**	**13**	**14**	**15**	**16**	**17**	**18**
Wiki za hudhurio	-4-8	-2-4	0	2	4	6	8	12	14	16		**30**	**32**	**34**	**46**	**48**	**50**	**58**	**70**
**Sababu ya hudhurio**	uchunguzi wa awali 1	Uchunguzi wa awali 2	Chanjo ya kwanza DNA 1	Ufuatiliaji1	Chanjo ya DNA 2	Ufuatiliaji 2	Ufuatiliaji 3	Chanjo ya DNA 3	Ufuatiliaji 4	Ufuatiliaji 5		MVA 1	Ufuatiliaji 7	Ufuatiliaji 8	MVA 2	Ufuatiliaji 9	Ufuatiliaji 10	Ufuatiliaji 11	Ufuatiliaji 12

Mabadiliko haya yamejadiliwa na kuidhinishwa na kamati ya sayansi ya utafiti wa TaMoVac-1 na hatudhani kuwa yatasababisha ongezeko la madhara kwako. Mabadiliko haya pia yalipelekwa na kuidhinishwa na kamati za maadili ya utafiti, ile ya kitaifa iliyopo NIMR, na zile za kitaasisi zilizopo Chuo Kikuu Muhimbili na mkoa wa Mbeya.

Pamoja na mabadiliko haya yatakayojitokeza mahudhurio ya awali hayatabadilika. Vile vile, kama utakuwa na maswali ya ziada, timu ya utafiti wana furaha kukupa taarifa zaidi na kujibu maswali yako yote.

Ninakiri kwamba nimesoma na kuelewa mabadiliiko haya ya kwanza kwenye fomu ya maelezo ya utafiti ya Agosti 2010, na nimeridhika na majibu niliyopatiwa ya maswali yangu yote.

Ninapendelea kuendelea na taratibu za utatifi huu: ☐ ndiyo ☐ hapana

____________________________ ________________
Jina na sahihi ya mshiriki Tarehe

____
Namba ya ushiriki

____________________________ ________________
Jina na sahihi ya mtafiti Tarehe

Appendix 5A: TaMoVac I Assessment of Understanding (English)

Please read each question and answer whether the statement is True or False.

True	False		
☐	☐	1.	Volunteers in this vaccine study will be protected against HIV and never have to worry about becoming HIV infected.
☐	☐	2.	The vaccine in this study will give you HIV.
☐	☐	3.	A purpose of this study is to see whether the vaccine is safe.
☐	☐	4.	If you enter this study, you may receive an inactive substance called a placebo instead of the vaccine.
☐	☐	5.	This vaccine has been shown to be safe in pregnant women, and a woman may become pregnant during this study if she wishes.
☐	☐	6.	This study will require you to come to the clinic multiple times for visits and blood draws over the next 17 months.
☐	☐	7.	Volunteers in this vaccine study can get infected with HIV if they have unprotected sex or if they share sharp instruments.
☐	☐	8.	You may take other experimental (test) products while you are taking part in this study.
☐	☐	9.	You may withdraw from the study at any time if you choose or your participation may be stopped if the study team decides it is in your best interest.
☐	☐	10.	As a result of the immunization, you may test positive for HIV. However, by using other tests we can determine that you are not infected with HIV.
☐	☐	11.	All components of this vaccine regimen have been previously used in human volunteers.
☐	☐	12.	The vaccine used in this study has copied parts of the HIV virus.
☐	☐	13.	I can participate in other studies investigating new drugs or vaccines during the same time.
☐	☐	14.	This study tests if this HIV vaccine can protect individuals from acquiring HIV infection.

☐	☐	15.	The conduct of the study is overseen by others including the regulatory authorities in Tanzania
☐	☐	16.	The multiple blood samples taken from me could be subjected to genetic tests
☐	☐	17.	By participating in this study, I am not allowed to father a child or become pregnant during the study period and 6 months after receiving the last vaccine dose
☐	☐	18.	If I voluntarily decide to withdraw from the study, I will continue to receive care at the Muhimbili National Hospital or the Mbeya Referral Hospital in case of sickness.
☐	☐	19.	The nurse who gives me the vaccine knows whether I am getting vaccine or placebo

Test repetition number: 1 2 3

Number and % or correct answers: ☐☐/19, ☐☐☐%

Appendix 5B: TaMoVac I Assessment of Understanding (Swahili)

TaMoVac I-MTIHANI WA KUPIMA UFAHAMU (TOU)

Tafadhali soma kila swali na kujibu maelezo yanayofuata kama Kweli (K) au Si kweli (S).

Kweli	Si kweli		
☐	☐	1.	Washiriki wa utafiti huu watapata kinga dhidi ya VVU na hawatakuwa na wasiwasi wa kupata maambukizi.
☐	☐	2.	Chanjo katika utafiti huu itakupa maambukizi ya VVU
☐	☐	3.	Lengo la utafiti huu ni kutafiti kama chanjo hii ni salama
☐	☐	4.	Kama utajiunga na utafiti huu, unaweza kupata mchanganyiko usiofanya kazi (placebo) badala ya chanjo.
☐	☐	5.	Chanjo hii imethibitika kuwa ni salama kwa wanawake wajawazito na mama anaweza kushika mimba wakati utafiti ukiendelea akipenda
☐	☐	6.	Utahitajika ufike kliniki mara nyingi kwa ajili ya mahudhurio na vipimo vya damu kwa kipindi cha miezi 17
☐	☐	7.	Washiriki wanaweza kupata maambukizi ya VVU kama watafanya ngono bila kinga au kushirikiana vyombo vyenye makali.
☐	☐	8.	Unaweza kutumia chanjo au dawa nyingine za majaribio wakati ukishiriki katika utafiti huu
☐	☐	9.	Unaweza kuacha kushiriki muda wowote kama ukiamua au kusimamishwa kama ikionekana kuwa kufanya hivyo ni kwa faida yako.
☐	☐	10.	Baada ya kupata chanjo, unaweza kuonekana kuwa umeathirika kwa vipimo vya kawaida kama ELISA lakini kwa kutumia vipimo vingine inaweza kuthibitishwa kwamba wewe huna maambukizi ya VVU.
☐	☐	11.	Viasili mchanganyiko vyote vya chanjo hii vimekuwa vikitumika huko nyuma katika binadamu wa kujitolea.
☐	☐	12.	Chanjo hii katika utafiti huu ina viasili vitokanavyo na sehemu za kirusi cha Ukimwi.
☐	☐	13.	Naweza kushiriki katika tafiti zingine za kugundua dawa mpya au chanjo wakati mmoja.
☐	☐	14.	Utafiti huu unapima kama chanjo hii ya VVU inaweza kuzuia watu wasipate maambukizi ya VVU.

☐	☐	15.	Utafiti unavyoendeshwa chini ya uangalizi wa watu wengine ikiwemo na mamlaka za maadilli za utafiti Tanzania,
☐	☐	16.	Damu zinazochukuliwa toka kwangu zitatumika kupima viasili vilivyomo.
☐	☐	17.	Wakati wa kushiriki katika utafiti huu,siruhusiwi kuzaa mtoto au kupata ujauzito na miezi 6 baada ya kupata chanjo ya mwisho.
☐	☐	18.	Kama nitajiondoa kutoka katika utafiti,nitaendelea kupata huduma toka hospitali ya taifa Muhimbili au hospitali ya rufaa ya Mbeya kama nitakuwa naumwa.
☐	☐	19.	Muuguzi anayenipa chanjo huwa anafahamu kama ananipa chanjo harisi au isio harisi (placebo).

Kurudia kufanya Mtihani huu wa kupima ufahamu ni mara 1,2,3

Idadi ya maswali aliojibu na asilimia yake au majibu sahihi: ☐☐/19, ☐☐☐%

Appendix 6A: TaMoVac I Risk Assessment of Volunteers (English)

We will ask you about your sexual experience. You do not have to answer any questions that make you feel uncomfortable however; this may affect your eligibility and opportunity to participate in this trial. All your answers will be treated confidentially. No one will be allowed to disclose our answers outside our research group.

1. Have you ever had sexual intercourse?
   a. [ ] Yes
   b. [ ] Never had *(jump to questions 7, 8, 9 and 10)*

2. How many sexual partners have you had during the past six months?
   a. [ ] ≤ Two (2)
   b. [ ] More than Two

3. Are you in a relationship with an HIV infected partner?
   a. [ ] Yes
   b. [ ] No
   c. [ ] I don't know

4. Among the people you have had sex with, are there any individuals who you knew or suspected were HIV positive in the past 6 months?
   a. [ ] Yes (Knew or Suspected)
   b. [ ] No

5. In the past 6 months, how often have you had sexual intercourse with prostitutes or exchanged sex for money, goods or services?
   a. [ ] Have NOT had sexual intercourse with prostitutes in the past 6 months
   b. [ ] Have NOT exchanged sex for money, goods or serviced in the past 6 months
   c. [ ] Have had sexual intercourse with prostitutes in the past 6 months
   d. [ ] Have exchanged sex for money, goods or serviced in the past 6 months

6. During the past 6 months, have you used condoms during sexual intercourse with casual partners?
   a. [ ] Always
   b. [ ] Sometimes
   c. [ ] Seldom or Never

7. In the past 6 months, have you been diagnosed with a sexually transmitted disease?
   a. [ ] Yes
   b. [ ] No

8. In the past 6 months, have you had any of the following symptoms?
   a. [ ] Genital sores
   b. [ ] Discharge from vagina with itching or burning
   c. [ ] Pus discharge from penis
   d. [ ] Frequent, difficult, or painful urination
   e. [ ] None of the above

9. Are you regular drinking alcohol defined as >21 units per week (male), >14 units per week (female) → 1 units = 1 beer or 1 glass of wine or measure of strong alcohol.
   a. [ ] Yes
   b. [ ] No

10. Have you ever used intravenous recreational drugs?
   a. [ ] Yes
   b. [ ] No

The use of this assessment tool is designed to facilitate a discussion with the patient and inform clinical judgment about volunteer risk. It is not a validated instrument and seeks to briefly identify high-risk behaviors which would be grounds for excluding a volunteer.

Appendix 6B: TaMoVac I Risk Assessment of Volunteers (Swahili)

KUPIMA UWEZEKANO WA MTU KUWA KATIKA HATARI YA KUPATA MAAMBUKIZO YA VVU

Tutakuuliza kuhusu historia yako ya kujamiana. Huwezi kujibu maswali yote ambayo hutajisikia vizuri kuyajibu japo itaathiri vigezo vyako na nafasi yako ya kushiriki katika utafiti wa majaribio haya. Majibu yako yote yatakuwa siri. Hakuna atakayeruhusiwa kutoa taarifa zako za siri nje ya kundi la utafiti.

1. Umewahi kujamiana?
   a. [ ] Ndiyo
   b. [ ] Hapana Sijawahi [nenda swali la 7,8, 9 na 10]

2. Umekuwa na wapenzi wangapi kipindi cha miezi sita iliyopita?
   a. [ ] Wawili au mmoja
   b. [ ] Zaidi ya wawili

3. Je una mahusiano ya kimapenzi na mtu mwenye Virusi Vya Ukimwi (VVU)?
   a. [ ] Ndiyo
   b. [ ] Hapana
   c. [ ] Sifahamu

4. Katika kipindi cha miezi iliyopita, kati ya wale uliofanya nao mapenzi, kuna ambao unafahamu au unawahisi wana maambukizi ya VVU?
   a. [ ] Ndiyo (Kufahamu au Kuhisi)
   b. [ ] Hapana

5. Katika kipindi cha miezi sita iliyopita, ni mara ngapi umefanya mapenzi na wanawake/wanaume wanaojiuza (changudoa) au kufanya mapenzi kwa sababu ya kupata pesa,vitu au huduma?
   a. [ ] Sijafanya mapenzi na watu wanaojiuza (changudoa) ndani ya miezi sita (6) iliyopita
   b. [ ] Sijafanya mapenzi kwa ajili ya kupata pesa, vitu au huduma katika miezi sita (6) iliyopita
   c. [ ] Nimefanya mapenzi na watu wanaojiuza (changudoa) ndani ya miezi sita (6) iliyopita
   d. [ ] Nimefanya mapenzi ili kupata pesa, vitu au huduma katika miezi sita (6) iliyopita

6. Katika miezi sita (6) iliyopita, umewahi kutumia kondomu wakati wa kujamiana na mpenzi/wapenzi wako?
   a. [ ] Mara zote
   b. [ ] Mara nyingine
   c. [ ] Mara Chache au Sijawahi

7. Katika miezi sita (6) iliyopita, umewahi kupata magonjwa ya zinaa?
   a. [ ] Ndiyo
   b. [ ] Hapana

8. Ndani ya miezi sita (6) iliyopita, je umepata moja ya dalili zifuatazo?
   a. [ ] Vidonda sehemu za siri
   b. [ ] Uchafu kutoka ukeni ukiambatana na kuwashwa/maumivu
   c. [ ] Usaha kutoka kwenye uume
   d. [ ] Maumivu/shida wakati wa kukojoa na kukojoa mara kwa mara
   e. [ ] Hakuna dalili

9. Una kawaida ya kunywa pombe zaidi ya 21 kwa wiki? (pombe = chupa ya bia au glasi ya waini au pombe kali)
   a. [ ] Ndiyo
   b. [ ] Hapana

10. Umewahi tumia dawa za kulevya?
   a. [ ] Ndiyo
   b. [ ] Hapana

Maswali haya katika kipengele hiki yamelenga kurahisisha majadiliano na mshiriki ili kuweza kuamua kama mshiriki yupo katika hatari kubwa ya kupata maambukizi ya VVU. Maswali haya si kipimo halisi cha uwezekano wa mshiriki kupata maambukizi ya VVU bali yanadhamiria kutoa taarifa ambazo zinaweza kumfanya mtu asishiriki katika utafiti.

Appendix 7: TaMoVac I Project - HIV Testing Algorithm

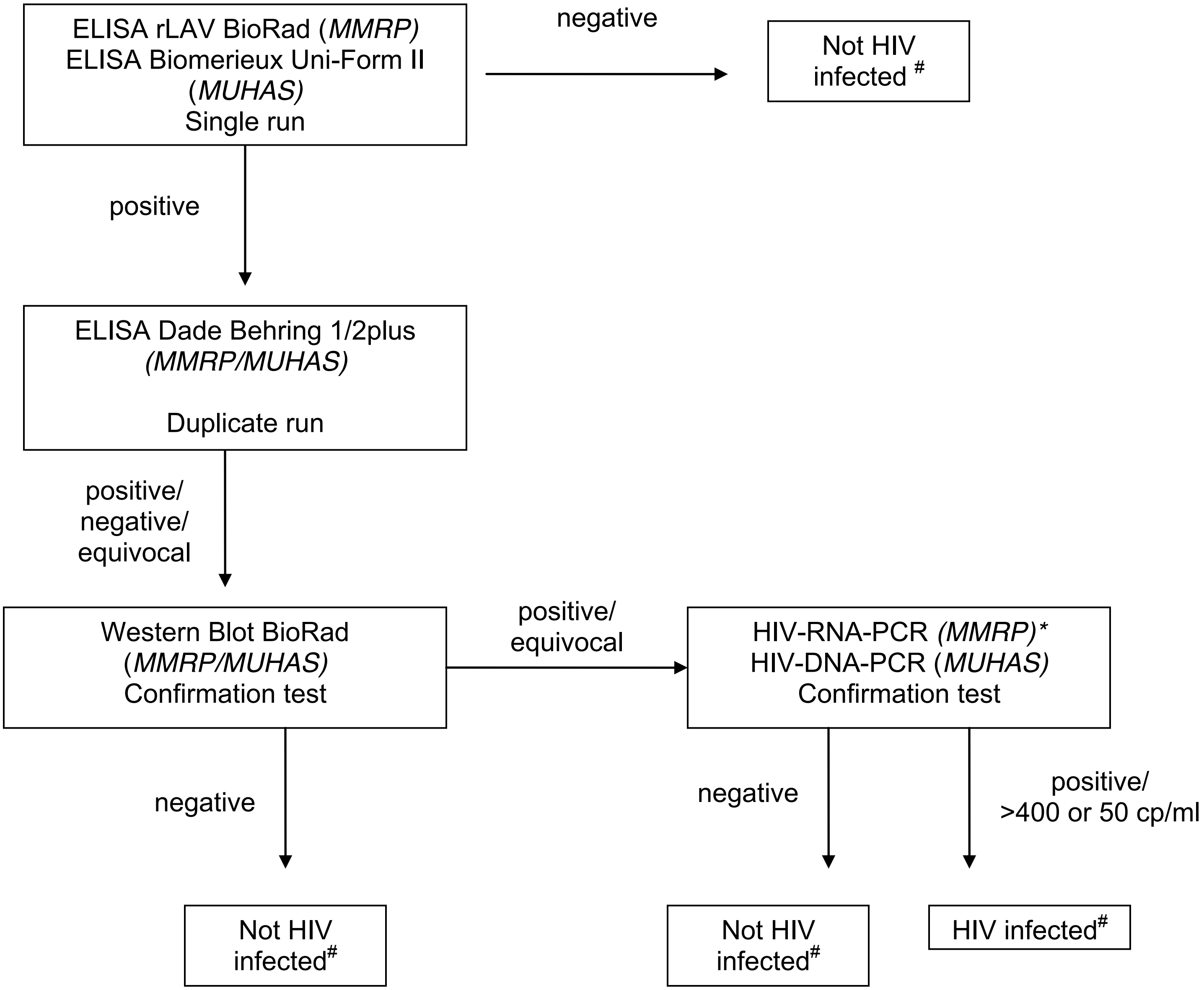

* First quantitative HIV-RNA-PCR with detection limit <400 copies/ml, if negative the ultra-sensitive HIV-RNA-PCR with detection limit <50 copies/ml will be performed for confirmation.

HIV test results during the study will be reported to the clinic only as "infected" of "not infected" for the purpose of not unblinding due to potentially "false positive" test results in vaccine recipients.

Appendix 8A: TaMoVac I Diary Card (English)

(Enter Site Logo Here)

TaMoVac I Participant Diary Card

Site Name and Location

Version 1.0

18 September 2008

Study ID	
Vaccination #	

Site Address

Phone: *Site Phone Number*

After Work Hours: *Site Phone Number*

FAX: *Site FAX Number*

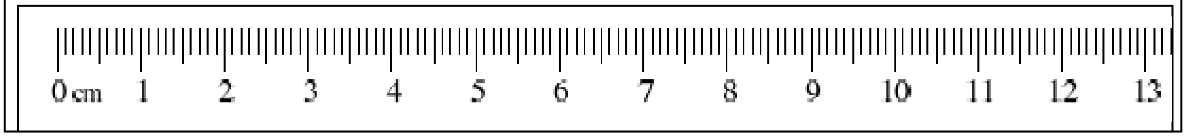

Symptoms Severity Scale

None

Mild= minimal symptoms; cause minimal or no interference with work, school, or self care activities.

Moderate=notable symptoms; required modification in activity; did not result in loss of work or cancellation of social activities

Severe = incapacitating symptoms; requiring bed rest and/or resulted in loss of work or cancellation of social activities.

For any Problems Call *Site Name* at
( ) *Site Phone Number* OR ( ) *Site Phone Number*

Injection Site Severity Scale

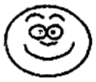

None

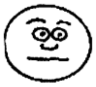

Mild= minimal symptoms; cause minimal or no interference with work, school, or self care activities.

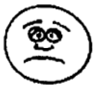

Moderate=notable symptoms; required modification in activity; did not result in loss of work or cancellation of social activities

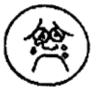

Severe = incapacitating symptoms; requiring bed rest and/or resulted in loss of work or cancellation of social activities.

Evening of Vaccination
Date:

Temp: __________ Time Taken: ___________

If you have a fever and wish to take your temperature during the night, record your additional temperature:
Temp: ________ Time Taken: _______ Temp: ________ Time Taken: _______

Are you taking any medication? Yes No
Specify:

Please use the symptom scale at the bottom of the page

General Symptoms	None	Mild	Moderate	Severe
Malaise/ Fatigue				
Chills				
Aching Joints				
Muscle Aches				
Headache				
Nausea				
Vomiting				

Injection Site Symptoms	None	Mild	Moderate	Severe	None	Mild	Moderate	Severe
Local Pain								
Local Itching								
Warmth								
Swelling (in cm)	__ __. __X __ __. __ cm				__ __. __X __ __. __ cm			
Redness (in cm)	__ __. __X __ __. __ cm				__ __. __X __ __. __ cm			
Induration (in cm)	__ __. __X __ __. __ cm				__ __. __X __ __. __ cm			
Clear blister (in cm)	__ __. __X __ __. __ cm				__ __. __X __ __. __ cm			
Blood blister (in cm)	__ __. __X __ __. __ cm				__ __. __X __ __. __ cm			
Papule (in cm)	__ __. __X __ __. __ cm				__ __. __X __ __. __ cm			

Symptoms Severity Scale

None
Mild=minimal symptoms; cause minimal or no interference with work, school, or self care activities.
Moderate=notable symptoms; required modification in activity or medication; did not result in loss of work or cancellation of social activities.
Severe = incapacitating symptoms; requiring bed rest and/or resulted in loss of work or cancellation of social activities.

Injection Site Severity Scale

None
Mild=minimal pain or tenderness; no limitation of use of arm
Moderate=notable pain or tenderness; some limitation of use of arm
Severe= extreme pain or tenderness; complete limitation of use of arm

For any problems call *Site Name* at ( ) *Site Phone Number* or ( ) *Site Phone Number*

Day 1 After Vaccination
Date:

Temp: __________ Time Taken: ___________

If you have a fever and wish to take your temperature during the night, record your additional temperature:
Temp: ________ Time Taken: _______ Temp: ________ Time Taken: _______

Are you taking any medication? Yes No
Specify:

Please use the symptom scale at the bottom of the page

General Symptoms	None	Mild	Moderate	Severe
Malaise/ Fatigue				
Chills				
Aching Joints				
Muscle Aches				
Headache				
Nausea				
Vomiting				

Injection Site Symptoms	None	Mild	Moderate	Severe	None	Mild	Moderate	Severe
Local Pain								
Local Itching								
Warmth								
Swelling (in cm)	__ __. __X __ __. __ cm				__ __. __X __ __. __ cm			
Redness (in cm)	__ __. __X __ __. __ cm				__ __. __X __ __. __ cm			
Induration (in cm)	__ __. __X __ __. __ cm				__ __. __X __ __. __ cm			
Clear blister (in cm)	__ __. __X __ __. __ cm				__ __. __X __ __. __ cm			
Blood blister (in cm)	__ __. __X __ __. __ cm				__ __. __X __ __. __ cm			
Papule (in cm)	__ __. __X __ __. __ cm				__ __. __X __ __. __ cm			

Symptoms Severity Scale

None
Mild=minimal symptoms; cause minimal or no interference with work, school, or self care activities.
Moderate=notable symptoms; required modification in activity or medication; did not result in loss of work or cancellation of social activities.
Severe = incapacitating symptoms; requiring bed rest and/or resulted in loss of work or cancellation of social activities.

Injection Site Severity Scale

None
Mild=minimal pain or tenderness; no limitation of use of arm
Moderate=notable pain or tenderness; some limitation of use of arm
Severe= extreme pain or tenderness; complete limitation of use of arm

For any problems call *Site Name* at ( ) *Site Phone Number* or ( ) *Site Phone Number*

Day 2 After Vaccination
Date:

Temp: __________ Time Taken: ___________

If you have a fever and wish to take your temperature during the night, record your additional temperature:
Temp: ________ Time Taken: _______ Temp: ________ Time Taken: _______

Are you taking any medication? Yes No
Specify:

Please use the symptom scale at the bottom of the page

General Symptoms	None	Mild	Moderate	Severe
Malaise/ Fatigue				
Chills				
Aching Joints				
Muscle Aches				
Headache				
Nausea				
Vomiting				

Injection Site Symptoms	None	Mild	Moderate	Severe	None	Mild	Moderate	Severe
Local Pain								
Local Itching								
Warmth								
Swelling (in cm)	__ __. __X __ __. __ cm				__ __. __X __ __. __ cm			
Redness (in cm)	__ __. __X __ __. __ cm				__ __. __X __ __. __ cm			
Induration (in cm)	__ __. __X __ __. __ cm				__ __. __X __ __. __ cm			
Clear blister (in cm)	__ __. __X __ __. __ cm				__ __. __X __ __. __ cm			
Blood blister (in cm)	__ __. __X __ __. __ cm				__ __. __X __ __. __ cm			
Papule (in cm)	__ __. __X __ __. __ cm				__ __. __X __ __. __ cm			

Symptoms Severity Scale

None
Mild=minimal symptoms; cause minimal or no interference with work, school, or self care activities.
Moderate=notable symptoms; required modification in activity or medication; did not result in loss of work or cancellation of social activities.
Severe = incapacitating symptoms; requiring bed rest and/or resulted in loss of work or cancellation of social activities.

Injection Site Severity Scale

None
Mild=minimal pain or tenderness; no limitation of use of arm
Moderate=notable pain or tenderness; some limitation of use of arm
Severe= extreme pain or tenderness; complete limitation of use of arm

For any problems call *Site Name* at ( ) *Site Phone Number* or ( ) *Site Phone Number*

Day 3 After Vaccination
Date:

Temp: __________ Time Taken: ___________

If you have a fever and wish to take your temperature during the night, record your additional temperature:
Temp: ________ Time Taken: _______ Temp: ________ Time Taken: _______

Are you taking any medication? Yes No
Specify:

Please use the symptom scale at the bottom of the page

General Symptoms	None	Mild	Moderate	Severe
Malaise/ Fatigue				
Chills				
Aching Joints				
Muscle Aches				
Headache				
Nausea				
Vomiting				

Injection Site Symptoms	None	Mild	Moderate	Severe	None	Mild	Moderate	Severe
Local Pain								
Local Itching								
Warmth								
Swelling (in cm)	__ __. __X __ __. __ cm				__ __. __X __ __. __ cm			
Redness (in cm)	__ __. __X __ __. __ cm				__ __. __X __ __. __ cm			
Induration (in cm)	__ __. __X __ __. __ cm				__ __. __X __ __. __ cm			
Clear blister (in cm)	__ __. __X __ __. __ cm				__ __. __X __ __. __ cm			
Blood blister (in cm)	__ __. __X __ __. __ cm				__ __. __X __ __. __ cm			
Papule (in cm)	__ __. __X __ __. __ cm				__ __. __X __ __. __ cm			

Symptoms Severity Scale

None
Mild=minimal symptoms; cause minimal or no interference with work, school, or self care activities.
Moderate=notable symptoms; required modification in activity or medication; did not result in loss of work or cancellation of social activities.
Severe = incapacitating symptoms; requiring bed rest and/or resulted in loss of work or cancellation of social activities.

Injection Site Severity Scale

None
Mild=minimal pain or tenderness; no limitation of use of arm
Moderate=notable pain or tenderness; some limitation of use of arm
Severe= extreme pain or tenderness; complete limitation of use of arm

For any problems call *Site Name* at ( ) *Site Phone Number* or ( ) *Site Phone Number*

Day 4 After Vaccination
Date:

Temp: __________ Time Taken: ___________

If you have a fever and wish to take your temperature during the night, record your additional temperature:
Temp: ________ Time Taken: _______ Temp: ________ Time Taken: _______

Are you taking any medication? Yes No
Specify:

Please use the symptom scale at the bottom of the page

General Symptoms	None	Mild	Moderate	Severe
Malaise/ Fatigue				
Chills				
Aching Joints				
Muscle Aches				
Headache				
Nausea				
Vomiting				

Injection Site Symptoms	None	Mild	Moderate	Severe	None	Mild	Moderate	Severe
Local Pain								
Local Itching								
Warmth								
Swelling (in cm)	__ __. __X __ __. __ cm				__ __. __X __ __. __ cm			
Redness (in cm)	__ __. __X __ __. __ cm				__ __. __X __ __. __ cm			
Induration (in cm)	__ __. __X __ __. __ cm				__ __. __X __ __. __ cm			
Clear blister (in cm)	__ __. __X __ __. __ cm				__ __. __X __ __. __ cm			
Blood blister (in cm)	__ __. __X __ __. __ cm				__ __. __X __ __. __ cm			
Papule (in cm)	__ __. __X __ __. __ cm				__ __. __X __ __. __ cm			

Symptoms Severity Scale

None
Mild=minimal symptoms; cause minimal or no interference with work, school, or self care activities.
Moderate=notable symptoms; required modification in activity or medication; did not result in loss of work or cancellation of social activities.
Severe = incapacitating symptoms; requiring bed rest and/or resulted in loss of work or cancellation of social activities.

Injection Site Severity Scale

None
Mild=minimal pain or tenderness; no limitation of use of arm
Moderate=notable pain or tenderness; some limitation of use of arm
Severe= extreme pain or tenderness; complete limitation of use of arm

For any problems call *Site Name* at ( ) *Site Phone Number* or ( ) *Site Phone Number*

Day 5 After Vaccination
Date:

Temp: __________ Time Taken: ___________

If you have a fever and wish to take your temperature during the night, record your additional temperature:
Temp: ________ Time Taken: _______ Temp: ________ Time Taken: _______

Are you taking any medication? Yes No
Specify:

Please use the symptom scale at the bottom of the page

General Symptoms	None	Mild	Moderate	Severe
Malaise/ Fatigue				
Chills				
Aching Joints				
Muscle Aches				
Headache				
Nausea				
Vomiting				

Injection Site Symptoms	None	Mild	Moderate	Severe	None	Mild	Moderate	Severe
Local Pain								
Local Itching								
Warmth								
Swelling (in cm)	__ __. __X __ __. __ cm				__ __. __X __ __. __ cm			
Redness (in cm)	__ __. __X __ __. __ cm				__ __. __X __ __. __ cm			
Induration (in cm)	__ __. __X __ __. __ cm				__ __. __X __ __. __ cm			
Clear blister (in cm)	__ __. __X __ __. __ cm				__ __. __X __ __. __ cm			
Blood blister (in cm)	__ __. __X __ __. __ cm				__ __. __X __ __. __ cm			
Papule (in cm)	__ __. __X __ __. __ cm				__ __. __X __ __. __ cm			

Symptoms Severity Scale

None
Mild=minimal symptoms; cause minimal or no interference with work, school, or self care activities.
Moderate=notable symptoms; required modification in activity or medication; did not result in loss of work or cancellation of social activities.
Severe = incapacitating symptoms; requiring bed rest and/or resulted in loss of work or cancellation of social activities.

Injection Site Severity Scale

None
Mild=minimal pain or tenderness; no limitation of use of arm
Moderate=notable pain or tenderness; some limitation of use of arm
Severe= extreme pain or tenderness; complete limitation of use of arm

For any problems call *Site Name* at ( ) *Site Phone Number* or ( ) *Site Phone Number*

Day 6 After Vaccination
Date:

Temp: __________ Time Taken: ___________

If you have a fever and wish to take your temperature during the night, record your additional temperature:
Temp: ________ Time Taken: _______ Temp: ________ Time Taken: _______

Are you taking any medication? Yes No
Specify:

Please use the symptom scale at the bottom of the page

General Symptoms	None	Mild	Moderate	Severe
Malaise/ Fatigue				
Chills				
Aching Joints				
Muscle Aches				
Headache				
Nausea				
Vomiting				

Injection Site Symptoms	None	Mild	Moderate	Severe	None	Mild	Moderate	Severe
Local Pain								
Local Itching								
Warmth								
Swelling (in cm)	__ __. __X __ __. __ cm				__ __. __X __ __. __ cm			
Redness (in cm)	__ __. __X __ __. __ cm				__ __. __X __ __. __ cm			
Induration (in cm)	__ __. __X __ __. __ cm				__ __. __X __ __. __ cm			
Clear blister (in cm)	__ __. __X __ __. __ cm				__ __. __X __ __. __ cm			
Blood blister (in cm)	__ __. __X __ __. __ cm				__ __. __X __ __. __ cm			
Papule (in cm)	__ __. __X __ __. __ cm				__ __. __X __ __. __ cm			

Symptoms Severity Scale

None
Mild=minimal symptoms; cause minimal or no interference with work, school, or self care activities.
Moderate=notable symptoms; required modification in activity or medication; did not result in loss of work or cancellation of social activities.
Severe = incapacitating symptoms; requiring bed rest and/or resulted in loss of work or cancellation of social activities.

Injection Site Severity Scale

None
Mild=minimal pain or tenderness; no limitation of use of arm
Moderate=notable pain or tenderness; some limitation of use of arm
Severe= extreme pain or tenderness; complete limitation of use of arm

For any problems call *Site Name* at ( ) *Site Phone Number* or ( ) *Site Phone Number*

Day 7 After Vaccination
Date:

Temp: __________ Time Taken: ___________

If you have a fever and wish to take your temperature during the night, record your additional temperature:
Temp: ________ Time Taken: _______ Temp: ________ Time Taken: _______

Are you taking any medication? Yes No
Specify:

Please use the symptom scale at the bottom of the page

General Symptoms	None	Mild	Moderate	Severe
Malaise/ Fatigue				
Chills				
Aching Joints				
Muscle Aches				
Headache				
Nausea				
Vomiting				

Injection Site Symptoms	None	Mild	Moderate	Severe	None	Mild	Moderate	Severe
Local Pain								
Local Itching								
Warmth								
Swelling (in cm)	__ __. __X __ __. __ cm				__ __. __X __ __. __ cm			
Redness (in cm)	__ __. __X __ __. __ cm				__ __. __X __ __. __ cm			
Induration (in cm)	__ __. __X __ __. __ cm				__ __. __X __ __. __ cm			
Clear blister (in cm)	__ __. __X __ __. __ cm				__ __. __X __ __. __ cm			
Blood blister (in cm)	__ __. __X __ __. __ cm				__ __. __X __ __. __ cm			
Papule (in cm)	__ __. __X __ __. __ cm				__ __. __X __ __. __ cm			

Symptoms Severity Scale

None
Mild=minimal symptoms; cause minimal or no interference with work, school, or self care activities.
Moderate=notable symptoms; required modification in activity or medication; did not result in loss of work or cancellation of social activities.
Severe = incapacitating symptoms; requiring bed rest and/or resulted in loss of work or cancellation of social activities.

Injection Site Severity Scale

None
Mild=minimal pain or tenderness; no limitation of use of arm
Moderate=notable pain or tenderness; some limitation of use of arm
Severe= extreme pain or tenderness; complete limitation of use of arm

For any problems call *Site Name* at ( ) *Site Phone Number* or ( ) *Site Phone Number*

If you have any of the General or Injection Site Symptoms that is still ongoing beyond day 3, please complete stop dates below.	
General Symptoms	Date the Symptom Ended
Malaise/ Fatigue	
Chills	
Aching Joints	
Muscle Aches	
Headache	
Nausea	
Vomiting	

Injection Site Symptoms	Date the Symptom Ended
Local Pain	
Local Itching	
Warmth	
Swelling	
Redness	
Induration	
Clear blister	
Blood blister	
Papule	

Are you or have you taken any medication after day 7? If so, please record the name or type of medication and the date you stopped taking the medication below.	
Name or Type of Medication	Date You Stopped Medication

Appendix 8B: TaMoVac I Diary Card (Swahili)

(Enter Site Logo Here)

TaMoVac I Kitabu Cha Kumbukumbu cha Mshiriki

Jina na mahali unapofanyika utafiti

Chapa 2.0
09 March 2010

Namba ya Utafiti

Namba ya Chanjo #

Anwani ya mahali pa utafiti

Simu: simu ya mahali pa utafiti

Baada za saa za kazi: *Simu ya mahali pa utafiti*

FAXI: *Faxi ya mahali pa utafiti*

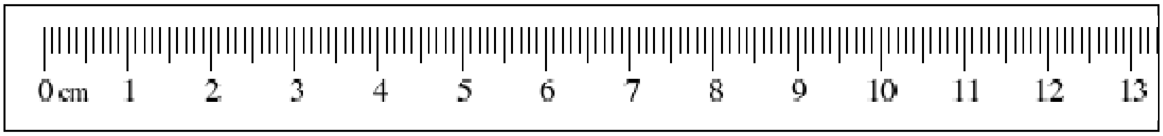

Mizani ya Ukubwa wa Dalili

Hakuna

Kidogo = dalili ndogo; hazijasababisha mtu kutokwenda kazini, shuleni au kushindwa kujifanyia usafi wa mwili.

Wastani=dalili za kuonekana; zimeathiri utendaji lakini hazijasababisha mtu kutofanya kazi au kutojishughulisha na michezo au shughuli za kila siku.

Sana = dalili kubwa; za kuhitaji kupumzika kitandani na/au zimesababisha mtu kuacha kazi au shughuli nyingine za kila siku.

Kwa Tatizo lolote, Piga Simu ....
Namba (……..) AU (……….)

Mizani ya Dalili Sehemu ya Uchomaji Sindano

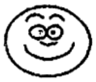

Hakuna

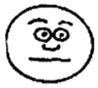

Kidogo = dalili ndogo; hazijasababisha mtu kutokwenda kazini, shuleni au kushindwa kujifanyia usafi wa mwili.

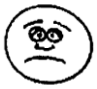

Wastani=dalili za kuonekana; zimeathiri utendaji lakini hazijasababisha mtu kutofanya kazi au kutojishughulisha na michezo au shughuli za kila siku.

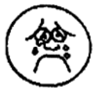

Sana = dalili kubwa; za kuhitaji kupumzika kitandani na/au zimesababisha mtu kuacha kazi au shughuli nyingine za kila siku.

Jioni ya Siku ya chanjo
Tarehe:

Jotoridi: ____________ Muda wa Kupima: _____________

Kama una homa na unataka kupima jotoridi wakati wa usiku, rekodi jotoridi la ziada hapa:
Jotoridi: _____________ Muda wa Kupima: ___________
Jotoridi: _____________ Muda wa Kupima: ___________

Je, unatumia dawa? Ndiyo Hapana
Elezea:

Tafadhali tumia mizani ya dalili kama ilivyo chini ya Ukurasa

Dalili za Jumla	Hakuna	Kidogo	Wastani	Sana
Kuchoka sana				
Kutetemeka				
Maumivu ya viungo				
Maumivu ya misuli				
Kuumwa kichwa				
Kichefuchefu				
Kutapika				

	Mkono wa kulia				Mkono wa kushoto			
Dalili Sehemu ya Uchomaji	Hakuna	Kidogo	Wastani	Sana	Hakuna	Kidogo	Wastani	Sana
Maumivu ya Sehemu ya uchomaji								
Kuwashwa sehemu ya uchomaji								
Ujotojoto sehemu ya uchomaji								
Uvimbe sehemu ya uchomaji kwa sm	__ __. __X __ __. __ sm				__ __. __X __ __. __ sm			
Ukubwa wa Uwekundu (kwa sm)	__ __. __X __ __. __ sm				__ __. __X __ __. __ sm			
Ukubwa wa Ugumu (kwa sm)	__ __. __X __ __. __ sm				__ __. __X __ __. __ sm			
Malengelenge yasiyo na damu (kwa sm)	__ __. __X __ __. __ sm				__ __. __X __ __. __ sm			
Malenegelenge yenye damu (kwa sm)	__ __. __X __ __. __ sm				__ __. __X __ __. __ sm			
Kipele (kwa sm)	__ __. __X __ __. __ sm				__ __. __X __ __. __ sm			

Tafadhali rekodi vipimo kwa sm, urefu na upana mkubwa zaidi *(Mfano: 01.5 X 02.5 sm)*

Mizani ya Ukubwa wa Dalili

Hakuna
Kidogo = dalili ndogo; hazijasababisha mtu kutokwenda kazini, shuleni au kushindwa kujifanyia usafi wa mwili.
Wastani = dalili za kuonekana; zimeathiri utendaji lakini hazijasababisha mtu kutofanya kazi au kutojishughulisha na michezo au shughuli za kila siku.
Sana = dalili kubwa za kuhitaji kupumzika kitandani na/au zimesababisha mtu kuacha kazi au shughuli nyingine za kila siku.

Mizani ya Dalili ya Sehemu ya Uchomaji

Hakuna
Kidogo = maumivu kidogo; hayajazuia matumizi yoyote ya mkono
Wastani = maumivu bayana; yamezuia matumizi fulani ya mkono.
Sana = maumivu makali; yamezuia kabisa matumizi ya mkono.

Kwa Tatizo lolote Piga simu .... **Namba (……….) au (…………)**

Siku ya 1
Tarehe:

Jotoridi: ____________ Muda wa Kupima: _____________

Kama una homa na unataka kupima jotoridi wakati wa usiku, rekodi jotoridi la ziada hapa:
Jotoridi: _____________ Muda wa Kupima: ___________
Jotoridi: _____________ Muda wa Kupima: ___________

Je, unatumia dawa? Ndiyo Hapana
Elezea:

Tafadhali tumia mizani ya dalili kama ilivyo chini ya Ukurasa

Dalili za Jumla	Hakuna	Kidogo	Wastani	Sana
Kuchoka sana				
Kutetemeka				
Maumivu ya viungo				
Maumivu ya misuli				
Kuumwa kichwa				
Kichefuchefu				
Kutapika				

	Mkono wa kulia				Mkono wa kushoto			
Dalili Sehemu ya Uchomaji	Hakuna	Kidogo	Wastani	Sana	Hakuna	Kidogo	Wastani	Sana
Maumivu ya Sehemu ya uchomaji								
Kuwashwa sehemu ya uchomaji								
Ujotojoto sehemu ya uchomaji								
Uvimbe sehemu ya uchomaji kwa sm	__ __. __X __ __. __ sm				__ __. __X __ __. __ sm			
Ukubwa wa Uwekundu (kwa sm)	__ __. __X __ __. __ sm				__ __. __X __ __. __ sm			
Ukubwa wa Ugumu (kwa sm)	__ __. __X __ __. __ sm				__ __. __X __ __. __ sm			
Malengelenge yasiyo na damu (kwa sm)	__ __. __X __ __. __ sm				__ __. __X __ __. __ sm			
Malenegelenge yenye damu (kwa sm)	__ __. __X __ __. __ sm				__ __. __X __ __. __ sm			
Kipele (kwa sm)	__ __. __X __ __. __ sm				__ __. __X __ __. __ sm			

Tafadhali rekodi vipimo kwa sm, urefu na upana mkubwa zaidi *(Mfano: 01.5 X 02.5 sm)*

Mizani ya Ukubwa wa Dalili

Hakuna
Kidogo = dalili ndogo; hazijasababisha mtu kutokwenda kazini, shuleni au kushindwa kujifanyia usafi wa mwili.
Wastani = dalili za kuonekana; zimeathiri utendaji lakini hazijasababisha mtu kutofanya kazi au kutojishughulisha na michezo au shughuli za kila siku.
Sana = dalili kubwa za kuhitaji kupumzika kitandani na/au zimesababisha mtu kuacha kazi au shughuli nyingine za kila siku.

Mizani ya Dalili ya Sehemu ya Uchomaji

Hakuna
Kidogo = maumivu kidogo; hayajazuia matumizi yoyote ya mkono
Wastani = maumivu bayana; yamezuia matumizi fulani ya mkono.
Sana = maumivu makali; yamezuia kabisa matumizi ya mkono.

Kwa Tatizo lolote Piga simu .... **Namba (……….) au (…………)**

Siku ya 2
Tarehe:

Jotoridi: ____________ Muda wa Kupima: _____________

Kama una homa na unataka kupima jotoridi wakati wa usiku, rekodi jotoridi la ziada hapa:
Jotoridi: _____________ Muda wa Kupima: ___________
Jotoridi: _____________ Muda wa Kupima: ___________

Je, unatumia dawa? Ndiyo Hapana
Elezea:

Tafadhali tumia mizani ya dalili kama ilivyo chini ya Ukurasa

Dalili za Jumla	Hakuna	Kidogo	Wastani	Sana
Kuchoka sana				
Kutetemeka				
Maumivu ya viungo				
Maumivu ya misuli				
Kuumwa kichwa				
Kichefuchefu				
Kutapika				

	Mkono wa kulia				Mkono wa kushoto			
Dalili Sehemu ya Uchomaji	Hakuna	Kidogo	Wastani	Sana	Hakuna	Kidogo	Wastani	Sana
Maumivu ya Sehemu ya uchomaji								
Kuwashwa sehemu ya uchomaji								
Ujotojoto sehemu ya uchomaji								
Uvimbe sehemu ya uchomaji kwa sm	__ __. __X __ __. __ sm				__ __. __X __ __. __ sm			
Ukubwa wa Uwekundu (kwa sm)	__ __. __X __ __. __ sm				__ __. __X __ __. __ sm			
Ukubwa wa Ugumu (kwa sm)	__ __. __X __ __. __ sm				__ __. __X __ __. __ sm			
Malengelenge yasiyo na damu (kwa sm)	__ __. __X __ __. __ sm				__ __. __X __ __. __ sm			
Malenegelenge yenye damu (kwa sm)	__ __. __X __ __. __ sm				__ __. __X __ __. __ sm			
Kipele (kwa sm)	__ __. __X __ __. __ sm				__ __. __X __ __. __ sm			

Tafadhali rekodi vipimo kwa sm, urefu na upana mkubwa zaidi *(Mfano: 01.5 X 02.5 sm)*

Mizani ya Ukubwa wa Dalili

Hakuna
Kidogo = dalili ndogo; hazijasababisha mtu kutokwenda kazini, shuleni au kushindwa kujifanyia usafi wa mwili.
Wastani = dalili za kuonekana; zimeathiri utendaji lakini hazijasababisha mtu kutofanya kazi au kutojishughulisha na michezo au shughuli za kila siku.
Sana = dalili kubwa za kuhitaji kupumzika kitandani na/au zimesababisha mtu kuacha kazi au shughuli nyingine za kila siku.

Mizani ya Dalili ya Sehemu ya Uchomaji

Hakuna
Kidogo = maumivu kidogo; hayajazuia matumizi yoyote ya mkono
Wastani = maumivu bayana; yamezuia matumizi fulani ya mkono.
Sana = maumivu makali; yamezuia kabisa matumizi ya mkono.

Kwa Tatizo lolote Piga simu .... **Namba (……….) au (…………)**

Siku ya 3
Tarehe:

Jotoridi: ____________ Muda wa Kupima: _____________

Kama una homa na unataka kupima jotoridi wakati wa usiku, rekodi jotoridi la ziada hapa:
Jotoridi: _____________ Muda wa Kupima: ___________
Jotoridi: _____________ Muda wa Kupima: ___________

Je, unatumia dawa? Ndiyo Hapana
Elezea:

Tafadhali tumia mizani ya dalili kama ilivyo chini ya Ukurasa

Dalili za Jumla	Hakuna	Kidogo	Wastani	Sana
Kuchoka sana				
Kutetemeka				
Maumivu ya viungo				
Maumivu ya misuli				
Kuumwa kichwa				
Kichefuchefu				
Kutapika				

	Mkono wa kulia				Mkono wa kushoto			
Dalili Sehemu ya Uchomaji	Hakuna	Kidogo	Wastani	Sana	Hakuna	Kidogo	Wastani	Sana
Maumivu ya Sehemu ya uchomaji								
Kuwashwa sehemu ya uchomaji								
Ujotojoto sehemu ya uchomaji								
Uvimbe sehemu ya uchomaji kwa sm	__ __. __X __ __. __ sm				__ __. __X __ __. __ sm			
Ukubwa wa Uwekundu (kwa sm)	__ __. __X __ __. __ sm				__ __. __X __ __. __ sm			
Ukubwa wa Ugumu (kwa sm)	__ __. __X __ __. __ sm				__ __. __X __ __. __ sm			
Malengelenge yasiyo na damu (kwa sm)	__ __. __X __ __. __ sm				__ __. __X __ __. __ sm			
Malenegelenge yenye damu (kwa sm)	__ __. __X __ __. __ sm				__ __. __X __ __. __ sm			
Kipele (kwa sm)	__ __. __X __ __. __ sm				__ __. __X __ __. __ sm			

Tafadhali rekodi vipimo kwa sm, urefu na upana mkubwa zaidi *(Mfano: 01.5 X 02.5 sm)*

Mizani ya Ukubwa wa Dalili

Hakuna
Kidogo = dalili ndogo; hazijasababisha mtu kutokwenda kazini, shuleni au kushindwa kujifanyia usafi wa mwili.
Wastani = dalili za kuonekana; zimeathiri utendaji lakini hazijasababisha mtu kutofanya kazi au kutojishughulisha na michezo au shughuli za kila siku.
Sana = dalili kubwa za kuhitaji kupumzika kitandani na/au zimesababisha mtu kuacha kazi au shughuli nyingine za kila siku.

Mizani ya Dalili ya Sehemu ya Uchomaji

Hakuna
Kidogo = maumivu kidogo; hayajazuia matumizi yoyote ya mkono
Wastani = maumivu bayana; yamezuia matumizi fulani ya mkono.
Sana = maumivu makali; yamezuia kabisa matumizi ya mkono.

Kwa Tatizo lolote Piga simu .... **Namba (……….) au (…………)**

Siku ya 4
Tarehe:

Jotoridi: ____________ Muda wa Kupima: _____________

Kama una homa na unataka kupima jotoridi wakati wa usiku, rekodi jotoridi la ziada hapa:
Jotoridi: _____________ Muda wa Kupima: ___________
Jotoridi: _____________ Muda wa Kupima: ___________

Je, unatumia dawa? Ndiyo Hapana
Elezea:

Tafadhali tumia mizani ya dalili kama ilivyo chini ya Ukurasa

Dalili za Jumla	Hakuna	Kidogo	Wastani	Sana
Kuchoka sana				
Kutetemeka				
Maumivu ya viungo				
Maumivu ya misuli				
Kuumwa kichwa				
Kichefuchefu				
Kutapika				

	Mkono wa kulia				Mkono wa kushoto			
Dalili Sehemu ya Uchomaji	Hakuna	Kidogo	Wastani	Sana	Hakuna	Kidogo	Wastani	Sana
Maumivu ya Sehemu ya uchomaji								
Kuwashwa sehemu ya uchomaji								
Ujotojoto sehemu ya uchomaji								
Uvimbe sehemu ya uchomaji kwa sm	__ __. __X __ __. __ sm				__ __. __X __ __. __ sm			
Ukubwa wa Uwekundu (kwa sm)	__ __. __X __ __. __ sm				__ __. __X __ __. __ sm			
Ukubwa wa Ugumu (kwa sm)	__ __. __X __ __. __ sm				__ __. __X __ __. __ sm			
Malengelenge yasiyo na damu (kwa sm)	__ __. __X __ __. __ sm				__ __. __X __ __. __ sm			
Malenegelenge yenye damu (kwa sm)	__ __. __X __ __. __ sm				__ __. __X __ __. __ sm			
Kipele (kwa sm)	__ __. __X __ __. __ sm				__ __. __X __ __. __ sm			

Tafadhali rekodi vipimo kwa sm, urefu na upana mkubwa zaidi *(Mfano: 01.5 X 02.5 sm)*

Mizani ya Ukubwa wa Dalili

Hakuna
Kidogo = dalili ndogo; hazijasababisha mtu kutokwenda kazini, shuleni au kushindwa kujifanyia usafi wa mwili.
Wastani = dalili za kuonekana; zimeathiri utendaji lakini hazijasababisha mtu kutofanya kazi au kutojishughulisha na michezo au shughuli za kila siku.
Sana = dalili kubwa za kuhitaji kupumzika kitandani na/au zimesababisha mtu kuacha kazi au shughuli nyingine za kila siku.

Mizani ya Dalili ya Sehemu ya Uchomaji

Hakuna
Kidogo = maumivu kidogo; hayajazuia matumizi yoyote ya mkono
Wastani = maumivu bayana; yamezuia matumizi fulani ya mkono.
Sana = maumivu makali; yamezuia kabisa matumizi ya mkono.

Kwa Tatizo lolote Piga simu .... **Namba (……….) au (…………)**

Siku ya 5
Tarehe:

Jotoridi: ____________ Muda wa Kupima: _____________

Kama una homa na unataka kupima jotoridi wakati wa usiku, rekodi jotoridi la ziada hapa:
Jotoridi: _____________ Muda wa Kupima: ___________
Jotoridi: _____________ Muda wa Kupima: ___________

Je, unatumia dawa? Ndiyo Hapana
Elezea:

Tafadhali tumia mizani ya dalili kama ilivyo chini ya Ukurasa

Dalili za Jumla	Hakuna	Kidogo	Wastani	Sana
Kuchoka sana				
Kutetemeka				
Maumivu ya viungo				
Maumivu ya misuli				
Kuumwa kichwa				
Kichefuchefu				
Kutapika				

	Mkono wa kulia				Mkono wa kushoto			
Dalili Sehemu ya Uchomaji	Hakuna	Kidogo	Wastani	Sana	Hakuna	Kidogo	Wastani	Sana
Maumivu ya Sehemu ya uchomaji								
Kuwashwa sehemu ya uchomaji								
Ujotojoto sehemu ya uchomaji								
Uvimbe sehemu ya uchomaji kwa sm	__ __. __X __ __. __ sm				__ __. __X __ __. __ sm			
Ukubwa wa Uwekundu (kwa sm)	__ __. __X __ __. __ sm				__ __. __X __ __. __ sm			
Ukubwa wa Ugumu (kwa sm)	__ __. __X __ __. __ sm				__ __. __X __ __. __ sm			
Malengelenge yasiyo na damu (kwa sm)	__ __. __X __ __. __ sm				__ __. __X __ __. __ sm			
Malenegelenge yenye damu (kwa sm)	__ __. __X __ __. __ sm				__ __. __X __ __. __ sm			
Kipele (kwa sm)	__ __. __X __ __. __ sm				__ __. __X __ __. __ sm			

Tafadhali rekodi vipimo kwa sm, urefu na upana mkubwa zaidi *(Mfano: 01.5 X 02.5 sm)*

Mizani ya Ukubwa wa Dalili

Hakuna
Kidogo = dalili ndogo; hazijasababisha mtu kutokwenda kazini, shuleni au kushindwa kujifanyia usafi wa mwili.
Wastani = dalili za kuonekana; zimeathiri utendaji lakini hazijasababisha mtu kutofanya kazi au kutojishughulisha na michezo au shughuli za kila siku.
Sana = dalili kubwa za kuhitaji kupumzika kitandani na/au zimesababisha mtu kuacha kazi au shughuli nyingine za kila siku.

Mizani ya Dalili ya Sehemu ya Uchomaji

Hakuna
Kidogo = maumivu kidogo; hayajazuia matumizi yoyote ya mkono
Wastani = maumivu bayana; yamezuia matumizi fulani ya mkono.
Sana = maumivu makali; yamezuia kabisa matumizi ya mkono.

Kwa Tatizo lolote Piga simu .... **Namba (……….) au (…………)**

Siku ya 6
Tarehe:

Jotoridi: ____________ Muda wa Kupima: _____________

Kama una homa na unataka kupima jotoridi wakati wa usiku, rekodi jotoridi la ziada hapa:
Jotoridi: _____________ Muda wa Kupima: ___________
Jotoridi: _____________ Muda wa Kupima: ___________

Je, unatumia dawa? Ndiyo Hapana
Elezea:

Tafadhali tumia mizani ya dalili kama ilivyo chini ya Ukurasa

Dalili za Jumla	Hakuna	Kidogo	Wastani	Sana
Kuchoka sana				
Kutetemeka				
Maumivu ya viungo				
Maumivu ya misuli				
Kuumwa kichwa				
Kichefuchefu				
Kutapika				

	Mkono wa kulia				Mkono wa kushoto			
Dalili Sehemu ya Uchomaji	Hakuna	Kidogo	Wastani	Sana	Hakuna	Kidogo	Wastani	Sana
Maumivu ya Sehemu ya uchomaji								
Kuwashwa sehemu ya uchomaji								
Ujotojoto sehemu ya uchomaji								
Uvimbe sehemu ya uchomaji kwa sm	__ __. __X __ __. __ sm				__ __. __X __ __. __ sm			
Ukubwa wa Uwekundu (kwa sm)	__ __. __X __ __. __ sm				__ __. __X __ __. __ sm			
Ukubwa wa Ugumu (kwa sm)	__ __. __X __ __. __ sm				__ __. __X __ __. __ sm			
Malengelenge yasiyo na damu (kwa sm)	__ __. __X __ __. __ sm				__ __. __X __ __. __ sm			
Malenegelenge yenye damu (kwa sm)	__ __. __X __ __. __ sm				__ __. __X __ __. __ sm			
Kipele (kwa sm)	__ __. __X __ __. __ sm				__ __. __X __ __. __ sm			

Tafadhali rekodi vipimo kwa sm, urefu na upana mkubwa zaidi *(Mfano: 01.5 X 02.5 sm)*

Mizani ya Ukubwa wa Dalili

Hakuna
Kidogo = dalili ndogo; hazijasababisha mtu kutokwenda kazini, shuleni au kushindwa kujifanyia usafi wa mwili.
Wastani = dalili za kuonekana; zimeathiri utendaji lakini hazijasababisha mtu kutofanya kazi au kutojishughulisha na michezo au shughuli za kila siku.
Sana = dalili kubwa za kuhitaji kupumzika kitandani na/au zimesababisha mtu kuacha kazi au shughuli nyingine za kila siku.

Mizani ya Dalili ya Sehemu ya Uchomaji

Hakuna
Kidogo = maumivu kidogo; hayajazuia matumizi yoyote ya mkono
Wastani = maumivu bayana; yamezuia matumizi fulani ya mkono.
Sana = maumivu makali; yamezuia kabisa matumizi ya mkono.

Kwa Tatizo lolote Piga simu .... **Namba (……….) au (…………)**

Siku ya 7
Tarehe:

Jotoridi: ____________ Muda wa Kupima: _____________

Kama una homa na unataka kupima jotoridi wakati wa usiku, rekodi jotoridi la ziada hapa:
Jotoridi: _____________ Muda wa Kupima: ___________
Jotoridi: _____________ Muda wa Kupima: ___________

Je, unatumia dawa? Ndiyo Hapana
Elezea:

Tafadhali tumia mizani ya dalili kama ilivyo chini ya Ukurasa

Dalili za Jumla	Hakuna	Kidogo	Wastani	Sana
Kuchoka sana				
Kutetemeka				
Maumivu ya viungo				
Maumivu ya misuli				
Kuumwa kichwa				
Kichefuchefu				
Kutapika				

	Mkono wa kulia				Mkono wa kushoto			
Dalili Sehemu ya Uchomaji	Hakuna	Kidogo	Wastani	Sana	Hakuna	Kidogo	Wastani	Sana
Maumivu ya Sehemu ya uchomaji								
Kuwashwa sehemu ya uchomaji								
Ujotojoto sehemu ya uchomaji								
Uvimbe sehemu ya uchomaji kwa sm	__ __. __X __ __. __ sm				__ __. __X __ __. __ sm			
Ukubwa wa Uwekundu (kwa sm)	__ __. __X __ __. __ sm				__ __. __X __ __. __ sm			
Ukubwa wa Ugumu (kwa sm)	__ __. __X __ __. __ sm				__ __. __X __ __. __ sm			
Malengelenge yasiyo na damu (kwa sm)	__ __. __X __ __. __ sm				__ __. __X __ __. __ sm			
Malenegelenge yenye damu (kwa sm)	__ __. __X __ __. __ sm				__ __. __X __ __. __ sm			
Kipele (kwa sm)	__ __. __X __ __. __ sm				__ __. __X __ __. __ sm			

Tafadhali rekodi vipimo kwa sm, urefu na upana mkubwa zaidi *(Mfano: 01.5 X 02.5 sm)*

Mizani ya Ukubwa wa Dalili

Hakuna
Kidogo = dalili ndogo; hazijasababisha mtu kutokwenda kazini, shuleni au kushindwa kujifanyia usafi wa mwili.
Wastani = dalili za kuonekana; zimeathiri utendaji lakini hazijasababisha mtu kutofanya kazi au kutojishughulisha na michezo au shughuli za kila siku.
Sana = dalili kubwa za kuhitaji kupumzika kitandani na/au zimesababisha mtu kuacha kazi au shughuli nyingine za kila siku.

Mizani ya Dalili ya Sehemu ya Uchomaji

Hakuna
Kidogo = maumivu kidogo; hayajazuia matumizi yoyote ya mkono
Wastani = maumivu bayana; yamezuia matumizi fulani ya mkono.
Sana = maumivu makali; yamezuia kabisa matumizi ya mkono.

Kwa Tatizo lolote Piga simu .... **Namba (……….) au (…………)**

Iwapo una dalili za mwili mzima au mahali palipochomwa sindano panaendelea zaidi ya siku ya 3,Tafadhari jaza tarehe hapo chini.	
Dalili za Jumla	Tarehe dalili ilipoishi
Kuchoka Sana	
Kutetemeka	
Maumivu ya Viungo	
Maumivu ya Misuli	
Kuumwa Kichwa	
Kichefuchefu	
Kutapika	

Dalili Sehemu ya Uchomaji	Tarehe dalili ilipoishi
Maumivu ya Sehemu	
Kuwashwa	
Ujotojoto	
Uvimbe	
Ukubwa wa Uwekundu	
Ukubwa wa Ugumu kwa sm.	
Malengelenge yasiyo na damu	
Malenegelenge yenye damu	
Uvimbe	

Umekunywa dawa yeyote baada ya siku ya 7? Kama umekunywa,tafadhari andika jina au aina ya dawa na tarehe ya kuacha kutumia hapo chini.	
Jina au aina ya dawa	Tarehe ya kuacha kutumia dawa

Appendix 9: DAIDS Table for Clinical and Lab Criteria

CLINICAL				
**PARAMETER**	**GRADE 1 MILD**	**GRADE 2 MODERATE**	**GRADE 3 SEVERE**	**GRADE 4 POTENTIALLY LIFE-THREATENING**
**ESTIMATING SEVERITY GRADE**				
Clinical adverse event NOT identified elsewhere in this DAIDS AE grading table	Symptoms causing no or minimal interference with usual social & functional activities	Symptoms causing greater than minimal interference with usual social & functional activities	Symptoms causing inability to perform usual social & functional activities	Symptoms causing inability to perform basic self-care functions OR Medical or operative intervention indicated to prevent permanent impairment, persistent disability, or death
**SYSTEMIC**				
Acute systemic allergic reaction	Localized urticaria (wheals) with no medical intervention indicated	Localized urticaria with medical intervention indicated OR Mild angioedema with no medical intervention indicated	Generalized urticaria OR Angioedema with medical intervention indicated OR Symptomatic mild bronchospasm	Acute anaphylaxis OR Life-threatening bronchospasm OR laryngeal edema
Chills	Symptoms causing no or minimal interference with usual social & functional activities	Symptoms causing greater than minimal interference with usual social & functional activities	Symptoms causing inability to perform usual social & functional activities	NA
Fatigue<br>Malaise	Symptoms causing no or minimal interference with usual social & functional activities	Symptoms causing greater than minimal interference with usual social & functional activities	Symptoms causing inability to perform usual social & functional activities	Incapacitating fatigue/ malaise symptoms causing inability to perform basic self-care functions
Fever (nonaxillary)	37.7 – 38.6°C	38.7 – 39.3°C	39.4 – 40.5°C	> 40.5°C
Pain (indicate body site)<br>DO NOT use for pain due to injection (See Injection Site Reactions: Injection site pain)<br>See also Headache, Arthralgia, and Myalgia	Pain causing no or minimal interference with usual social & functional activities	Pain causing greater than minimal interference with usual social & functional activities	Pain causing inability to perform usual social & functional activities	Disabling pain causing inability to perform basic self-care functions OR Hospitalization (other than emergency room visit) indicated
Unintentional weight loss	NA	5 – 9% loss in body weight from baseline	10 – 19% loss in body weight from baseline	≥ 20% loss in body weight from baseline OR Aggressive intervention indicated [e.g., tube feeding or total parenteral nutrition (TPN)]

CLINICAL				
**PARAMETER**	**GRADE 1<br>MILD**	**GRADE 2<br>MODERATE**	**GRADE 3<br>SEVERE**	**GRADE 4<br>POTENTIALLY LIFE-THREATENING**
**INFECTION**				
Infection (any other than HIV infection)	Localized, no systemic antimicrobial treatment indicated AND Symptoms causing no or minimal interference with usual social & functional activities	Systemic antimicrobial treatment indicated OR Symptoms causing greater than minimal interference with usual social & functional activities	Systemic antimicrobial treatment indicated AND Symptoms causing inability to perform usual social & functional activities OR Operative intervention (other than simple incision and drainage) indicated	Life-threatening consequences (e.g., septic shock)
**INJECTION SITE REACTIONS**				
Injection site pain (pain without touching)<br>Or<br>Tenderness (pain when area is touched)	Pain/tenderness causing no or minimal limitation of use of limb	Pain/tenderness limiting use of limb OR Pain/tenderness causing greater than minimal interference with usual social & functional activities	Pain/tenderness causing inability to perform usual social & functional activities	Pain/tenderness causing inability to perform basic self-care function OR Hospitalization (other than emergency room visit) indicated for management of pain/tenderness
Injection site reaction (localized)				
**Adult > 15 years**	Erythema OR Induration of 5x5 cm – 9x9 cm (or 25 cm^2 – $81cm^2$)	Erythema OR Induration OR Edema > 9 cm any diameter (or > 81 cm^2)	Ulceration OR Secondary infection OR Phlebitis OR Sterile abscess OR Drainage	Necrosis (involving dermis and deeper tissue)
**Pediatric ≤ 15 years**	Erythema OR Induration OR Edema present but ≤ 2.5 cm diameter	Erythema OR Induration OR Edema > 2.5 cm diameter but < 50% surface area of the extremity segment (e.g., upper arm/thigh)	Erythema OR Induration OR Edema involving ≥ 50% surface area of the extremity segment (e.g., upper arm/thigh) OR Ulceration OR Secondary infection OR Phlebitis OR Sterile abscess OR Drainage	Necrosis (involving dermis and deeper tissue)
Pruritis associated with injection<br>See also Skin: Pruritis (itching - no skin lesions)	Itching localized to injection site AND Relieved spontaneously or with < 48 hours treatment	Itching beyond the injection site but not generalized OR Itching localized to injection site requiring ≥ 48 hours treatment	Generalized itching causing inability to perform usual social & functional activities	NA
**SKIN – DERMATOLOGICAL**				
Alopecia	Thinning detectable by study participant (or by caregiver for young children and disabled adults)	Thinning or patchy hair loss detectable by health care provider	Complete hair loss	NA

CLINICAL				
**PARAMETER**	**GRADE 1 MILD**	**GRADE 2 MODERATE**	**GRADE 3 SEVERE**	**GRADE 4 POTENTIALLY LIFE-THREATENING**
Cutaneous reaction – rash	Localized macular rash	Diffuse macular, maculopapular, or morbilliform rash OR Target lesions	Diffuse macular, maculopapular, or morbilliform rash with vesicles or limited number of bullae OR Superficial ulcerations of mucous membrane limited to one site	Extensive or generalized bullous lesions OR Stevens-Johnson syndrome OR Ulceration of mucous membrane involving two or more distinct mucosal sites OR Toxic epidermal necrolysis (TEN)
Hyperpigmentation	Slight or localized	Marked or generalized	NA	NA
Hypopigmentation	Slight or localized	Marked or generalized	NA	NA
Pruritis (itching – no skin lesions)<br>(See also Injection Site Reactions: Pruritis associated with injection)	Itching causing no or minimal interference with usual social & functional activities	Itching causing greater than minimal interference with usual social & functional activities	Itching causing inability to perform usual social & functional activities	NA
**CARDIOVASCULAR**				
Cardiac arrhythmia (general)<br>(By ECG or physical exam)	Asymptomatic AND No intervention indicated	Asymptomatic AND Non-urgent medical intervention indicated	Symptomatic, non-life-threatening AND Non-urgent medical intervention indicated	Life-threatening arrhythmia OR Urgent intervention indicated
Cardiac-ischemia/infarction	NA	NA	Symptomatic ischemia (stable angina) OR Testing consistent with ischemia	Unstable angina OR Acute myocardial infarction
Hemorrhage (significant acute blood loss)	NA	Symptomatic AND No transfusion indicated	Symptomatic AND Transfusion of ≤ 2 units packed RBCs (for children ≤ 10 cc/kg) indicated	Life-threatening hypotension OR Transfusion of > 2 units packed RBCs (for children > 10 cc/kg) indicated
Hypertension				
**Adult > 17 years**<br>(with repeat testing at same visit)	> 140 – 159 mmHg systolic<br>OR<br>> 90 – 99 mmHg diastolic	> 160 – 179 mmHg systolic<br>OR<br>> 100 – 109 mmHg diastolic	> 180 mmHg systolic<br>OR<br>> 110 mmHg diastolic	Life-threatening consequences (e.g., malignant hypertension) OR Hospitalization indicated (other than emergency room visit)
**Pediatric ≤ 17 years**<br>(with repeat testing at same visit)	NA	91st – 94th percentile adjusted for age, height, and gender (systolic and/or diastolic)	≥ 95th percentile adjusted for age, height, and gender (systolic and/or diastolic)	Life-threatening consequences (e.g., malignant hypertension) OR Hospitalization indicated (other than emergency room visit)

CLINICAL				
**PARAMETER**	**GRADE 1 MILD**	**GRADE 2 MODERATE**	**GRADE 3 SEVERE**	**GRADE 4 POTENTIALLY LIFE-THREATENING**
Hypotension	NA	Symptomatic, corrected with oral fluid replacement	Symptomatic, IV fluids indicated	Shock requiring use of vasopressors or mechanical assistance to maintain blood pressure
Pericardial effusion	Asymptomatic, small effusion requiring no intervention	Asymptomatic, moderate or larger effusion requiring no intervention	Effusion with non-life threatening physiologic consequences OR Effusion with non-urgent intervention indicated	Life-threatening consequences (e.g., tamponade) OR Urgent intervention indicated
Prolonged PR interval				
**Adult > 16 years**	PR interval 0.21 – 0.25 sec	PR interval > 0.25 sec	Type II 2nd degree AV block OR Ventricular pause > 3.0 sec	Complete AV block
**Pediatric ≤ 16 years**	1st degree AV block (PR > normal for age and rate)	Type I 2nd degree AV block	Type II 2nd degree AV block	Complete AV block
Prolonged QTc				
**Adult > 16 years**	Asymptomatic, QTc interval 0.45 – 0.47 sec OR Increase interval < 0.03 sec above baseline	Asymptomatic, QTc interval 0.48 – 0.49 sec OR Increase in interval 0.03 – 0.05 sec above baseline	Asymptomatic, QTc interval ≥ 0.50 sec OR Increase in interval ≥ 0.06 sec above baseline	Life-threatening consequences, e.g. Torsade de pointes or other associated serious ventricular dysrhythmia
**Pediatric ≤ 16 years**	Asymptomatic, QTc interval 0.450 – 0.464 sec	Asymptomatic, QTc interval 0.465 – 0.479 sec	Asymptomatic, QTc interval ≥ 0.480 sec	Life-threatening consequences, e.g. Torsade de pointes or other associated serious ventricular dysrhythmia
Thrombosis/embolism	NA	Deep vein thrombosis AND No intervention indicated (e.g., anticoagulation, lysis filter, invasive procedure)	Deep vein thrombosis AND Intervention indicated (e.g., anticoagulation, lysis filter, invasive procedure)	Embolic event (e.g., pulmonary embolism, life-threatening thrombus)
Vasovagal episode (associated with a procedure of any kind)	Present without loss of consciousness	Present with transient loss of consciousness	NA	NA
Ventricular dysfunction (congestive heart failure)	NA	Asymptomatic diagnostic finding AND intervention indicated	New onset with symptoms OR Worsening symptomatic congestive heart failure	Life-threatening congestive heart failure
**GASTROINTESTINAL**				
Anorexia	Loss of appetite without decreased	Loss of appetite associated with	Loss of appetite associated with	Life-threatening consequences OR

CLINICAL				
**PARAMETER**	**GRADE 1 MILD**	**GRADE 2 MODERATE**	**GRADE 3 SEVERE**	**GRADE 4 POTENTIALLY LIFE-THREATENING**
	oral intake	decreased oral intake without significant weight loss	significant weight loss	Aggressive intervention indicated [e.g., tube feeding or total parenteral nutrition (TPN)]
Ascites	Asymptomatic	Symptomatic AND Intervention indicated (e.g., diuretics or therapeutic paracentesis)	Symptomatic despite intervention	Life-threatening consequences
Cholecystitis	NA	Symptomatic AND Medical intervention indicated	Radiologic, endoscopic, or operative intervention indicated	Life-threatening consequences (e.g., sepsis or perforation)
Constipation	NA	Persistent constipation requiring regular use of dietary modifications, laxatives, or enemas	Obstipation with manual evacuation indicated	Life-threatening consequences (e.g., obstruction)
Diarrhea				
**Adult and Pediatric ≥ 1 year**	Transient or intermittent episodes of unformed stools OR Increase of ≤ 3 stools over baseline per 24-hour period	Persistent episodes of unformed to watery stools OR Increase of 4 – 6 stools over baseline per 24-hour period	Bloody diarrhea OR Increase of ≥ 7 stools per 24-hour period OR IV fluid replacement indicated	Life-threatening consequences (e.g., hypotensive shock)
**Pediatric < 1 year**	Liquid stools (more unformed than usual) but usual number of stools	Liquid stools with increased number of stools OR Mild dehydration	Liquid stools with moderate dehydration	Liquid stools resulting in severe dehydration with aggressive rehydration indicated OR Hypotensive shock
Dysphagia-Odynophagia	Symptomatic but able to eat usual diet	Symptoms causing altered dietary intake without medical intervention indicated	Symptoms causing severely altered dietary intake with medical intervention indicated	Life-threatening reduction in oral intake
Mucositis/stomatitis (<u>clinical exam</u>)<br>Indicate site (e.g., larynx, oral)<br>See Genitourinary for Vulvovaginitis<br>See also Dysphagia-Odynophagia and Proctitis	Erythema of the mucosa	Patchy pseudomembranes or ulcerations	Confluent pseudomembranes or ulcerations OR Mucosal bleeding with minor trauma	Tissue necrosis OR Diffuse spontaneous mucosal bleeding OR Life-threatening consequences (e.g., aspiration, choking)
Nausea	Transient (< 24 hours) or intermittent nausea with no or minimal	Persistent nausea resulting in decreased oral intake for 24 – 48	Persistent nausea resulting in minimal oral intake for > 48 hours	Life-threatening consequences (e.g., hypotensive shock)

CLINICAL				
**PARAMETER**	**GRADE 1 MILD**	**GRADE 2 MODERATE**	**GRADE 3 SEVERE**	**GRADE 4 POTENTIALLY LIFE-THREATENING**
	interference with oral intake	hours	OR Aggressive rehydration indicated (e.g., IV fluids)	
Pancreatitis	NA	Symptomatic AND Hospitalization not indicated (other than emergency room visit)	Symptomatic AND Hospitalization indicated (other than emergency room visit)	Life-threatening consequences (e.g., circulatory failure, hemorrhage, sepsis)
Proctitis (functional-symptomatic)<br>Also see Mucositis/stomatitis for clinical exam	Rectal discomfort AND No intervention indicated	Symptoms causing greater than minimal interference with usual social & functional activities OR Medical intervention indicated	Symptoms causing inability to perform usual social & functional activities OR Operative intervention indicated	Life-threatening consequences (e.g., perforation)
Vomiting	Transient or intermittent vomiting with no or minimal interference with oral intake	Frequent episodes of vomiting with no or mild dehydration	Persistent vomiting resulting in orthostatic hypotension OR Aggressive rehydration indicated (e.g., IV fluids)	Life-threatening consequences (e.g., hypotensive shock)
**NEUROLOGIC**				
Alteration in personality-behavior or in mood (e.g., agitation, anxiety, depression, mania, psychosis)	Alteration causing no or minimal interference with usual social & functional activities	Alteration causing greater than minimal interference with usual social & functional activities	Alteration causing inability to perform usual social & functional activities	Behavior potentially harmful to self or others (e.g., suicidal and homicidal ideation or attempt, acute psychosis) OR Causing inability to perform basic self-care functions
Altered Mental Status<br>For Dementia, see Cognitive and behavioral/attentional disturbance (including dementia and attention deficit disorder)	Changes causing no or minimal interference with usual social & functional activities	Mild lethargy or somnolence causing greater than minimal interference with usual social & functional activities	Confusion, memory impairment, lethargy, or somnolence causing inability to perform usual social & functional activities	Delirium OR obtundation, OR coma
Ataxia	Asymptomatic ataxia detectable on exam OR Minimal ataxia causing no or minimal interference with usual social & functional activities	Symptomatic ataxia causing greater than minimal interference with usual social & functional activities	Symptomatic ataxia causing inability to perform usual social & functional activities	Disabling ataxia causing inability to perform basic self-care functions
Cognitive and behavioral/attentional disturbance (including dementia and	Disability causing no or minimal interference with usual social &	Disability causing greater than minimal interference with usual social & functional	Disability causing inability to perform usual social & functional activities OR	Disability causing inability to perform basic self-care functions OR Institutionalization

CLINICAL				
**PARAMETER**	**GRADE 1 MILD**	**GRADE 2 MODERATE**	**GRADE 3 SEVERE**	**GRADE 4 POTENTIALLY LIFE-THREATENING**
attention deficit disorder)	functional activities OR Specialized resources not indicated	activities OR Specialized resources on part-time basis indicated	Specialized resources on a full-time basis indicated	indicated
CNS ischemia (acute)	NA	NA	Transient ischemic attack	Cerebral vascular accident (CVA, stroke) with neurological deficit
Developmental delay – **Pediatric ≤ 16 years**	Mild developmental delay, either motor or cognitive, as determined by comparison with a developmental screening tool appropriate for the setting	Moderate developmental delay, either motor or cognitive, as determined by comparison with a developmental screening tool appropriate for the setting	Severe developmental delay, either motor or cognitive, as determined by comparison with a developmental screening tool appropriate for the setting	Developmental regression, either motor or cognitive, as determined by comparison with a developmental screening tool appropriate for the setting
Headache	Symptoms causing no or minimal interference with usual social & functional activities	Symptoms causing greater than minimal interference with usual social & functional activities	Symptoms causing inability to perform usual social & functional activities	Symptoms causing inability to perform basic self-care functions OR Hospitalization indicated (other than emergency room visit) OR Headache with significant impairment of alertness or other neurologic function
Insomnia	NA	Difficulty sleeping causing greater than minimal interference with usual social & functional activities	Difficulty sleeping causing inability to perform usual social & functional activities	Disabling insomnia causing inability to perform basic self-care functions
Neuromuscular weakness (including myopathy & neuropathy)	Asymptomatic with decreased strength on exam OR Minimal muscle weakness causing no or minimal interference with usual social & functional activities	Muscle weakness causing greater than minimal interference with usual social & functional activities	Muscle weakness causing inability to perform usual social & functional activities	Disabling muscle weakness causing inability to perform basic self-care functions OR Respiratory muscle weakness impairing ventilation
Neurosensory alteration (including paresthesia and painful neuropathy)	Asymptomatic with sensory alteration on exam or minimal paresthesia causing no or minimal interference with usual social & functional activities	Sensory alteration or paresthesia causing greater than minimal interference with usual social & functional activities	Sensory alteration or paresthesia causing inability to perform usual social & functional activities	Disabling sensory alteration or paresthesia causing inability to perform basic self-care functions
Seizure: (new onset) **– Adult ≥ 18 years**	NA	1 seizure	2 – 4 seizures	Seizures of any kind which are prolonged,

CLINICAL				
**PARAMETER**	**GRADE 1 MILD**	**GRADE 2 MODERATE**	**GRADE 3 SEVERE**	**GRADE 4 POTENTIALLY LIFE-THREATENING**
See also Seizure: (known pre-existing seizure disorder)				repetitive (e.g., status epilepticus), or difficult to control (e.g., refractory epilepsy)
Seizure: (known pre-existing seizure disorder) **– Adult ≥ 18 years** For worsening of existing epilepsy the grades should be based on an increase from previous level of control to any of these levels.	NA	Increased frequency of pre-existing seizures (non-repetitive) without change in seizure character OR Infrequent break-through seizures while on stable medication in a previously controlled seizure disorder	Change in seizure character from baseline either in duration or quality (e.g., severity or focality)	Seizures of any kind which are prolonged, repetitive (e.g., status epilepticus), or difficult to control (e.g., refractory epilepsy)
Seizure **– Pediatric < 18 years**	Seizure, generalized onset with or without secondary generalization, lasting < 5 minutes with < 24 hours post ictal state	Seizure, generalized onset with or without secondary generalization, lasting 5 – 20 minutes with < 24 hours post ictal state	Seizure, generalized onset with or without secondary generalization, lasting > 20 minutes	Seizure, generalized onset with or without secondary generalization, requiring intubation and sedation
Syncope (not associated with a procedure)	NA	Present	NA	NA
Vertigo	Vertigo causing no or minimal interference with usual social & functional activities	Vertigo causing greater than minimal interference with usual social & functional activities	Vertigo causing inability to perform usual social & functional activities	Disabling vertigo causing inability to perform basic self-care functions
**RESPIRATORY**				
Bronchospasm (acute)	FEV1 or peak flow reduced to 70 – 80%	FEV1 or peak flow 50 – 69%	FEV1 or peak flow 25 – 49%	Cyanosis OR FEV1 or peak flow < 25% OR Intubation
Dyspnea or respiratory distress				
**Adult ≥ 14 years**	Dyspnea on exertion with no or minimal interference with usual social & functional activities	Dyspnea on exertion causing greater than minimal interference with usual social & functional activities	Dyspnea at rest causing inability to perform usual social & functional activities	Respiratory failure with ventilatory support indicated
**Pediatric < 14 years**	Wheezing OR minimal increase in respiratory rate for age	Nasal flaring OR Intercostal retractions OR Pulse oximetry 90 – 95%	Dyspnea at rest causing inability to perform usual social & functional activities OR Pulse oximetry < 90%	Respiratory failure with ventilatory support indicated

CLINICAL				
**PARAMETER**	**GRADE 1 MILD**	**GRADE 2 MODERATE**	**GRADE 3 SEVERE**	**GRADE 4 POTENTIALLY LIFE-THREATENING**
**MUSCULOSKELETAL**				
Arthralgia<br>See also Arthritis	Joint pain causing no or minimal interference with usual social & functional activities	Joint pain causing greater than minimal interference with usual social & functional activities	Joint pain causing inability to perform usual social & functional activities	Disabling joint pain causing inability to perform basic self-care functions
Arthritis<br>See also Arthralgia	Stiffness or joint swelling causing no or minimal interference with usual social & functional activities	Stiffness or joint swelling causing greater than minimal interference with usual social & functional activities	Stiffness or joint swelling causing inability to perform usual social & functional activities	Disabling joint stiffness or swelling causing inability to perform basic self-care functions
Bone Mineral Loss				
**Adult ≥ 21 years**	BMD t-score -2.5 to -1.0	BMD t-score < -2.5	Pathological fracture (including loss of vertebral height)	Pathologic fracture causing life-threatening consequences
**Pediatric < 21 years**	BMD z-score -2.5 to -1.0	BMD z-score < -2.5	Pathological fracture (including loss of vertebral height)	Pathologic fracture causing life-threatening consequences
Myalgia<br>(non-injection site)	Muscle pain causing no or minimal interference with usual social & functional activities	Muscle pain causing greater than minimal interference with usual social & functional activities	Muscle pain causing inability to perform usual social & functional activities	Disabling muscle pain causing inability to perform basic self-care functions
Osteonecrosis	NA	Asymptomatic with radiographic findings AND No operative intervention indicated	Symptomatic bone pain with radiographic findings OR Operative intervention indicated	Disabling bone pain with radiographic findings causing inability to perform basic self-care functions
**GENITOURINARY**				
Cervicitis (symptoms)<br>(For use in studies evaluating topical study agents)<br>For other cervicitis see Infection: Infection (any other than HIV infection)	Symptoms causing no or minimal interference with usual social & functional activities	Symptoms causing greater than minimal interference with usual social & functional activities	Symptoms causing inability to perform usual social & functional activities	Symptoms causing inability to perform basic self-care functions
Cervicitis (clinical exam)<br>(For use in studies evaluating topical study agents)<br>For other cervicitis see	Minimal cervical abnormalities on examination (erythema, mucopurulent discharge, or friability) OR Epithelial	Moderate cervical abnormalities on examination (erythema, mucopurulent discharge, or friability) OR Epithelial	Severe cervical abnormalities on examination (erythema, mucopurulent discharge, or friability) OR Epithelial disruption	Epithelial disruption > 75% total surface

CLINICAL				
**PARAMETER**	**GRADE 1 MILD**	**GRADE 2 MODERATE**	**GRADE 3 SEVERE**	**GRADE 4 POTENTIALLY LIFE-THREATENING**
Infection: Infection (any other than HIV infection)	disruption < 25% of total surface	disruption of 25 – 49% total surface	50 – 75% total surface	
Inter-menstrual bleeding (IMB)	Spotting observed by participant OR Minimal blood observed during clinical or colposcopic examination	Inter-menstrual bleeding not greater in duration or amount than usual menstrual cycle	Inter-menstrual bleeding greater in duration or amount than usual menstrual cycle	Hemorrhage with life-threatening hypotension OR Operative intervention indicated
Urinary tract obstruction (e.g., stone)	NA	Signs or symptoms of urinary tract obstruction without hydronephrosis or renal dysfunction	Signs or symptoms of urinary tract obstruction with hydronephrosis or renal dysfunction	Obstruction causing life-threatening consequences
Vulvovaginitis (symptoms)<br>(Use in studies evaluating topical study agents)<br>For other vulvovaginitis see Infection: Infection (any other than HIV infection)	Symptoms causing no or minimal interference with usual social & functional activities	Symptoms causing greater than minimal interference with usual social & functional activities	Symptoms causing inability to perform usual social & functional activities	Symptoms causing inability to perform basic self-care functions
Vulvovaginitis (clinical exam)<br>(Use in studies evaluating topical study agents)<br>For other vulvovaginitis see Infection: Infection (any other than HIV infection)	Minimal vaginal abnormalities on examination OR Epithelial disruption < 25% of total surface	Moderate vaginal abnormalities on examination OR Epithelial disruption of 25 - 49% total surface	Severe vaginal abnormalities on examination OR Epithelial disruption 50 - 75% total surface	Vaginal perforation OR Epithelial disruption > 75% total surface
**OCULAR/VISUAL**				
Uveitis	Asymptomatic but detectable on exam	Symptomatic anterior uveitis OR Medical intervention indicated	Posterior or pan-uveitis OR Operative intervention indicated	Disabling visual loss in affected eye(s)
Visual changes (from baseline)	Visual changes causing no or minimal interference with usual social & functional activities	Visual changes causing greater than minimal interference with usual social & functional activities	Visual changes causing inability to perform usual social & functional activities	Disabling visual loss in affected eye(s)
**ENDOCRINE/METABOLIC**				
Abnormal fat accumulation	Detectable by study participant (or by	Detectable on physical exam by health care	Disfiguring OR Obvious changes on casual	NA

CLINICAL				
**PARAMETER**	**GRADE 1 MILD**	**GRADE 2 MODERATE**	**GRADE 3 SEVERE**	**GRADE 4 POTENTIALLY LIFE-THREATENING**
(e.g., back of neck, breasts, abdomen)	caregiver for young children and disabled adults)	provider	visual inspection	
Diabetes mellitus	NA	New onset without need to initiate medication OR Modification of current medications to regain glucose control	New onset with initiation of medication indicated OR Diabetes uncontrolled despite treatment modification	Life-threatening consequences (e.g., ketoacidosis, hyperosmolar non-ketotic coma)
Gynecomastia	Detectable by study participant or caregiver (for young children and disabled adults)	Detectable on physical exam by health care provider	Disfiguring OR Obvious on casual visual inspection	NA
Hyperthyroidism	Asymptomatic	Symptomatic causing greater than minimal interference with usual social & functional activities OR Thyroid suppression therapy indicated	Symptoms causing inability to perform usual social & functional activities OR Uncontrolled despite treatment modification	Life-threatening consequences (e.g., thyroid storm)
Hypothyroidism	Asymptomatic	Symptomatic causing greater than minimal interference with usual social & functional activities OR Thyroid replacement therapy indicated	Symptoms causing inability to perform usual social & functional activities OR Uncontrolled despite treatment modification	Life-threatening consequences (e.g., myxedema coma)
Lipoatrophy (e.g., fat loss from the face, extremities, buttocks)	Detectable by study participant (or by caregiver for young children and disabled adults)	Detectable on physical exam by health care provider	Disfiguring OR Obvious on casual visual inspection	NA

LABORATORY				
**PARAMETER**	**GRADE 1<br>MILD**	**GRADE 2<br>MODERATE**	**GRADE 3<br>SEVERE**	**GRADE 4<br>POTENTIALLY LIFE-THREATENING**
**HEMATOLOGY**	***Standard International Units are listed in italics***			
Absolute CD4+ count<br>**– Adult and Pediatric > 13 years**<br>(HIV NEGATIVE ONLY)	300 – 400/mm^3<br>*300 – 400/µL*	200 – 299/mm^3<br>*200 – 299/µL*	100 – 199/mm^3<br>*100 – 199/µL*	< 100/mm^3<br>*< 100/µL*
Absolute lymphocyte count<br>**– Adult and Pediatric > 13 years**<br>(HIV NEGATIVE ONLY)	600 – 650/mm^3<br>*0.600 x 10^9 – 0.650 x 10^9/L*	500 – 599/mm^3<br>*0.500 x 10^9 – 0.599 x 10^9/L*	350 – 499/mm^3<br>*0.350 x 10^9 – 0.499 x 10^9/L*	< 350/mm^3<br>*< 0.350 x 10^9/L*
Absolute neutrophil count (ANC)				
**Adult and Pediatric, > 7 days**	1,000 – 1,300/mm^3<br>*1.000 x 10^9 – 1.300 x 10^9/L*	750 – 999/mm^3<br>*0.750 x 10^9 – 0.999 x 10^9/L*	500 – 749/mm^3<br>*0.500 x 10^9 – 0.749 x 10^9/L*	< 500/mm^3<br>*< 0.500 x 10^9/L*
**Infant[*†], 2 – ≤ 7 days**	1,250 – 1,500/mm^3<br>*1.250 x 10^9 – 1.500 x 10^9/L*	1,000 – 1,249/mm^3<br>*1.000 x 10^9 – 1.249 x 10^9/L*	750 – 999/mm^3<br>*0.750 x 10^9 – 0.999 x 10^9/L*	< 750/mm^3<br>*< 0.750 x 10^9/L*
**Infant[*†], 1 day**	4,000 – 5,000/mm^3<br>*4.000 x 10^9 – 5.000 x 10^9/L*	3,000 – 3,999/mm^3<br>*3.000 x 10^9 – 3.999 x10^9/L*	1,500 – 2,999/mm^3<br>*1.500 x 10^9 – 2.999 x 10^9/L*	< 1,500/mm^3<br>*< 1.500 x 10^9/L*
Fibrinogen, decreased	100 – 200 mg/dL<br>*1.00 – 2.00 g/L*<br>OR<br>0.75 – 0.99 x LLN	75 – 99 mg/dL<br>*0.75 – 0.99 g/L*<br>OR<br>0.50 – 0.74 x LLN	50 – 74 mg/dL<br>*0.50 – 0.74 g/L*<br>OR<br>0.25 – 0.49 x LLN	< 50 mg/dL<br>*< 0.50 g/L*<br>OR<br>< 0.25 x LLN<br>OR<br>Associated with gross bleeding
Hemoglobin (Hgb)				
**Adult and Pediatric ≥ 57 days**<br>(HIV POSITIVE ONLY)	8.5 – 10.0 g/dL<br>*1.32 – 1.55 mmol/L*	7.5 – 8.4 g/dL<br>*1.16 – 1.31 mmol/L*	6.50 – 7.4 g/dL<br>*1.01 – 1.15 mmol/L*	< 6.5 g/dL<br>*< 1.01 mmol/L*
**Adult and Pediatric ≥ 57 days**<br>(HIV NEGATIVE ONLY)	10.0 – 10.9 g/dL<br>*1.55 – 1.69 mmol/L*<br>OR<br>Any decrease<br>2.5 – 3.4 g/dL<br>*0.39 – 0.53 mmol/L*	9.0 – 9.9 g/dL<br>*1.40 – 1.54 mmol/L*<br>OR<br>Any decrease<br>3.5 – 4.4 g/dL<br>*0.54 – 0.68 mmol/L*	7.0 – 8.9 g/dL<br>*1.09 – 1.39 mmol/L*<br>OR<br>Any decrease<br>≥ 4.5 g/dL<br>*≥ 0.69 mmol/L*	< 7.0 g/dL<br>*< 1.09 mmol/L*
**Infant[*†], 36 – 56 days**<br>(HIV POSITIVE OR NEGATIVE)	8.5 – 9.4 g/dL<br>*1.32 – 1.46 mmol/L*	7.0 – 8.4 g/dL<br>*1.09 – 1.31 mmol/L*	6.0 – 6.9 g/dL<br>*0.93 – 1.08 mmol/L*	< 6.00 g/dL<br>*< 0.93 mmol/L*
**Infant[*†], 22 – 35 days**<br>(HIV POSITIVE OR NEGATIVE)	9.5 – 10.5 g/dL<br>*1.47 – 1.63 mmol/L*	8.0 – 9.4 g/dL<br>*1.24 – 1.46 mmol/L*	7.0 – 7.9 g/dL<br>*1.09 – 1.23 mmol/L*	< 7.00 g/dL<br>*< 1.09 mmol/L*

LABORATORY				
**PARAMETER**	**GRADE 1 MILD**	**GRADE 2 MODERATE**	**GRADE 3 SEVERE**	**GRADE 4 POTENTIALLY LIFE-THREATENING**
**Infant*†, 1 – 21 days** (HIV POSITIVE OR NEGATIVE)	12.0 – 13.0 g/dL<br>*1.86 – 2.02 mmol/L*	10.0 – 11.9 g/dL<br>*1.55 – 1.85 mmol/L*	9.0 – 9.9 g/dL<br>*1.40 – 1.54 mmol/L*	< 9.0 g/dL<br>*< 1.40 mmol/L*
International Normalized Ratio of prothrombin time (INR)	1.1 – 1.5 x ULN	1.6 – 2.0 x ULN	2.1 – 3.0 x ULN	> 3.0 x ULN
Methemoglobin	5.0 – 10.0%	10.1 – 15.0%	15.1 – 20.0%	> 20.0%
Prothrombin Time (PT)	1.1 – 1.25 x ULN	1.26 – 1.50 x ULN	1.51 – 3.00 x ULN	> 3.00 x ULN
Partial Thromboplastin Time (PTT)	1.1 – 1.66 x ULN	1.67 – 2.33 x ULN	2.34 – 3.00 x ULN	> 3.00 x ULN
Platelets, decreased	100,000 – 124,999/mm^3<br>*100.000 x 10^9 – 124.999 x 10^9/L*	50,000 – 99,999/mm^3<br>*50.000 x 10^9 – 99.999 x 10^9/L*	25,000 – 49,999/mm^3<br>*25.000 x 10^9 – 49.999 x 10^9/L*	< 25,000/mm^3<br>*< 25.000 x 10^9/L*
WBC, decreased	2,000 – 2,500/mm^3<br>*2.000 x 10^9 – 2.500 x 10^9/L*	1,500 – 1,999/mm^3<br>*1.500 x 10^9 – 1.999 x 10^9/L*	1,000 – 1,499/mm^3<br>*1.000 x 10^9 – 1.499 x 10^9/L*	< 1,000/mm^3<br>*< 1.000 x 10^9/L*
**CHEMISTRIES**	***Standard International Units are listed in italics***			
Acidosis	NA	pH < normal, but ≥ 7.3	pH < 7.3 without life-threatening consequences	pH < 7.3 with life-threatening consequences
Albumin, serum, low	3.0 g/dL – < LLN<br>*30 g/L – < LLN*	2.0 – 2.9 g/dL<br>*20 – 29 g/L*	< 2.0 g/dL<br>*< 20 g/L*	NA
Alkaline Phosphatase	1.25 – 2.5 x ULN†	2.6 – 5.0 x ULN†	5.1 – 10.0 x ULN†	> 10.0 x ULN†
Alkalosis	NA	pH > normal, but ≤ 7.5	pH > 7.5 without life-threatening consequences	pH > 7.5 with life-threatening consequences
ALT (SGPT)	1.25 – 2.5 x ULN	2.6 – 5.0 x ULN	5.1 – 10.0 x ULN	> 10.0 x ULN
AST (SGOT)	1.25 – 2.5 x ULN	2.6 – 5.0 x ULN	5.1 – 10.0 x ULN	> 10.0 x ULN
Bicarbonate, serum, low	16.0 mEq/L – < LLN<br>*16.0 mmol/L – < LLN*	11.0 – 15.9 mEq/L<br>*11.0 – 15.9 mmol/L*	8.0 – 10.9 mEq/L<br>*8.0 – 10.9 mmol/L*	< 8.0 mEq/L<br>*< 8.0 mmol/L*
Bilirubin (Total)				
**Adult and Pediatric > 14 days**	1.1 – 1.5 x ULN	1.6 – 2.5 x ULN	2.6 – 5.0 x ULN	> 5.0 x ULN
**Infant*†, ≤ 14 days** (non-hemolytic)	NA	20.0 – 25.0 mg/dL<br>*342 – 428 µmol/L*	25.1 – 30.0 mg/dL<br>*429 – 513 µmol/L*	> 30.0 mg/dL<br>*> 513.0 µmol/L*
**Infant*†, ≤ 14 days** (hemolytic)	NA	NA	20.0 – 25.0 mg/dL<br>*342 – 428 µmol/L*	> 25.0 mg/dL<br>*> 428 µmol/L*
Calcium, serum, high (corrected for albumin)				

LABORATORY

PARAMETER	GRADE 1<br>MILD	GRADE 2<br>MODERATE	GRADE 3<br>SEVERE	GRADE 4<br>POTENTIALLY<br>LIFE-THREATENING
**Adult and Pediatric ≥ 7 days**	10.6 – 11.5 mg/dL<br>*2.65 – 2.88 mmol/L*	11.6 – 12.5 mg/dL<br>*2.89 – 3.13 mmol/L*	12.6 – 13.5 mg/dL<br>*3.14 – 3.38 mmol/L*	> 13.5 mg/dL<br>*> 3.38 mmol/L*
**Infant[*†], < 7 days**	11.5 – 12.4 mg/dL<br>*2.88 – 3.10 mmol/L*	12.5 – 12.9 mg/dL<br>*3.11 – 3.23 mmol/L*	13.0 – 13.5 mg/dL<br>*3.245 – 3.38 mmol/L*	> 13.5 mg/dL<br>*> 3.38 mmol/L*
Calcium, serum, low (corrected for albumin)				
**Adult and Pediatric ≥ 7 days**	7.8 – 8.4 mg/dL<br>*1.95 – 2.10 mmol/L*	7.0 – 7.7 mg/dL<br>*1.75 – 1.94 mmol/L*	6.1 – 6.9 mg/dL<br>*1.53 – 1.74 mmol/L*	< 6.1 mg/dL<br>*< 1.53 mmol/L*
**Infant[*†], < 7 days**	6.5 – 7.5 mg/dL<br>*1.63 – 1.88 mmol/L*	6.0 – 6.4 mg/dL<br>*1.50 – 1.62 mmol/L*	5.50 – 5.90 mg/dL<br>*1.38 – 1.51 mmol/L*	< 5.50 mg/dL<br>*< 1.38 mmol/L*
Cardiac troponin I (cTnI)	NA	NA	NA	Levels consistent with myocardial infarction or unstable angina as defined by the manufacturer
Cardiac troponin T (cTnT)	NA	NA	NA	≥ 0.20 ng/mL<br>OR<br>Levels consistent with myocardial infarction or unstable angina as defined by the manufacturer
Cholesterol (fasting)				
**Adult ≥ 18 years**	200 – 239 mg/dL<br>*5.18 – 6.19 mmol/L*	240 – 300 mg/dL<br>*6.20 – 7.77 mmol/L*	> 300 mg/dL<br>*> 7.77 mmol/L*	NA
**Pediatric < 18 years**	170 – 199 mg/dL<br>*4.40 – 5.15 mmol/L*	200 – 300 mg/dL<br>*5.16 – 7.77 mmol/L*	> 300 mg/dL<br>*> 7.77 mmol/L*	NA
Creatine Kinase	3.0 – 5.9 x ULN[†]	6.0 – 9.9 x ULN[†]	10.0 – 19.9 x ULN[†]	≥ 20.0 x ULN[†]
Creatinine	1.1 – 1.3 x ULN[†]	1.4 – 1.8 x ULN[†]	1.9 – 3.4 x ULN[†]	≥ 3.5 x ULN[†]
Glucose, serum, high				
Nonfasting	116 – 160 mg/dL<br>*6.44 – 8.88 mmol/L*	161 – 250 mg/dL<br>*8.89 – 13.88 mmol/L*	251 – 500 mg/dL<br>*13.89 – 27.75 mmol/L*	> 500 mg/dL<br>*> 27.75 mmol/L*
Fasting	110 – 125 mg/dL<br>*6.11 – 6.94 mmol/L*	126 – 250 mg/dL<br>*6.95 – 13.88 mmol/L*	251 – 500 mg/dL<br>*13.89 – 27.75 mmol/L*	> 500 mg/dL<br>*> 27.75 mmol/L*
Glucose, serum, low				
**Adult and Pediatric ≥ 1 month**	55 – 64 mg/dL<br>*3.05 – 3.55 mmol/L*	40 – 54 mg/dL<br>*2.22 – 3.06 mmol/L*	30 – 39 mg/dL<br>*1.67 – 2.23 mmol/L*	< 30 mg/dL<br>*< 1.67 mmol/L*
**Infant[*†], < 1 month**	50 – 54 mg/dL<br>*2.78 – 3.00 mmol/L*	40 – 49 mg/dL<br>*2.22 – 2.77 mmol/L*	30 – 39 mg/dL<br>*1.67 – 2.21 mmol/L*	< 30 mg/dL<br>*< 1.67 mmol/L*
Lactate	< 2.0 x ULN without acidosis	≥ 2.0 x ULN without acidosis	Increased lactate with pH < 7.3 without life-threatening	Increased lactate with pH < 7.3 with life-threatening

LABORATORY				
**PARAMETER**	**GRADE 1 MILD**	**GRADE 2 MODERATE**	**GRADE 3 SEVERE**	**GRADE 4 POTENTIALLY LIFE-THREATENING**
			consequences	consequences
LDL cholesterol (fasting)				
**Adult ≥ 18 years**	130 – 159 mg/dL<br>*3.37 – 4.12 mmol/L*	160 – 190 mg/dL<br>*4.13 – 4.90 mmol/L*	≥ 190 mg/dL<br>*≥ 4.91 mmol/L*	NA
**Pediatric > 2 - < 18 years**	110 – 129 mg/dL<br>*2.85 – 3.34 mmol/L*	130 – 189 mg/dL<br>*3.35 – 4.90 mmol/L*	≥ 190 mg/dL<br>*≥ 4.91 mmol/L*	NA
Lipase	1.1 – 1.5 x ULN	1.6 – 3.0 x ULN	3.1 – 5.0 x ULN	> 5.0 x ULN
Magnesium, serum, low	1.2 – 1.4 mEq/L<br>*0.60 – 0.70 mmol/L*	0.9 – 1.1 mEq/L<br>*0.45 – 0.59 mmol/L*	0.6 – 0.8 mEq/L<br>*0.30 – 0.44 mmol/L*	< 0.60 mEq/L<br>*< 0.30 mmol/L*
Pancreatic amylase	1.1 – 1.5 x ULN	1.6 – 2.0 x ULN	2.1 – 5.0 x ULN	> 5.0 x ULN
Phosphate, serum, low				
**Adult and Pediatric > 14 years**	2.5 mg/dL – < LLN<br>*0.81 mmol/L – < LLN*	2.0 – 2.4 mg/dL<br>*0.65 – 0.80 mmol/L*	1.0 – 1.9 mg/dL<br>*0.32 – 0.64 mmol/L*	< 1.00 mg/dL<br>*< 0.32 mmol/L*
**Pediatric 1 year – 14 years**	3.0 – 3.5 mg/dL<br>*0.97 – 1.13 mmol/L*	2.5 – 2.9 mg/dL<br>*0.81 – 0.96 mmol/L*	1.5 – 2.4 mg/dL<br>*0.48 – 0.80 mmol/L*	< 1.50 mg/dL<br>*< 0.48 mmol/L*
**Pediatric < 1 year**	3.5 – 4.5 mg/dL<br>*1.13 – 1.45 mmol/L*	2.5 – 3.4 mg/dL<br>*0.81 – 1.12 mmol/L*	1.5 – 2.4 mg/dL<br>*0.48 – 0.80 mmol/L*	< 1.50 mg/dL<br>*< 0.48 mmol/L*
Potassium, serum, high	5.6 – 6.0 mEq/L<br>*5.6 – 6.0 mmol/L*	6.1 – 6.5 mEq/L<br>*6.1 – 6.5 mmol/L*	6.6 – 7.0 mEq/L<br>*6.6 – 7.0 mmol/L*	> 7.0 mEq/L<br>*> 7.0 mmol/L*
Potassium, serum, low	3.0 – 3.4 mEq/L<br>*3.0 – 3.4 mmol/L*	2.5 – 2.9 mEq/L<br>*2.5 – 2.9 mmol/L*	2.0 – 2.4 mEq/L<br>*2.0 – 2.4 mmol/L*	< 2.0 mEq/L<br>*< 2.0 mmol/L*
Sodium, serum, high	146 – 150 mEq/L<br>*146 – 150 mmol/L*	151 – 154 mEq/L<br>*151 – 154 mmol/L*	155 – 159 mEq/L<br>*155 – 159 mmol/L*	≥ 160 mEq/L<br>*≥ 160 mmol/L*
Sodium, serum, low	130 – 135 mEq/L<br>*130 – 135 mmol/L*	125 – 129 mEq/L<br>*125 – 129 mmol/L*	121 – 124 mEq/L<br>*121 – 124 mmol/L*	≤ 120 mEq/L<br>*≤ 120 mmol/L*
Triglycerides (fasting)	NA	500 – 750 mg/dL<br>*5.65 – 8.48 mmol/L*	751 – 1,200 mg/dL<br>*8.49 – 13.56 mmol/L*	> 1,200 mg/dL<br>*> 13.56 mmol/L*
Uric acid	7.5 – 10.0 mg/dL<br>*0.45 – 0.59 mmol/L*	10.1 – 12.0 mg/dL<br>*0.60 – 0.71 mmol/L*	12.1 – 15.0 mg/dL<br>*0.72 – 0.89 mmol/L*	> 15.0 mg/dL<br>*> 0.89 mmol/L*
**URINALYSIS**	***Standard International Units are listed in italics***			
Hematuria (microscopic)	6 – 10 RBC/HPF	> 10 RBC/HPF	Gross, with or without clots OR with RBC casts	Transfusion indicated
Proteinuria, random collection	1 +	2 – 3 +	4 +	NA
Proteinuria, 24 hour collection				
**Adult and Pediatric ≥ 10 years**	200 – 999 mg/24 h<br>*0.200 – 0.999 g/d*	1,000 – 1,999 mg/24 h<br>*1.000 – 1.999 g/d*	2,000 – 3,500 mg/24 h<br>*2.000 – 3.500 g/d*	> 3,500 mg/24 h<br>*> 3.500 g/d*

LABORATORY				
**PARAMETER**	**GRADE 1 MILD**	**GRADE 2 MODERATE**	**GRADE 3 SEVERE**	**GRADE 4 POTENTIALLY LIFE-THREATENING**
**Pediatric > 3 mo - < 10 years**	201 – 499 mg/m^2/24 h<br>*0.201 – 0.499 g/d*	500 – 799 mg/m^2/24 h<br>*0.500 – 0.799 g/d*	800 – 1,000 mg/m^2/24 h<br>*0.800 – 1.000 g/d*	> 1,000 mg/ m^2/24 h<br>*> 1.000 g/d*

APPENDIX 10: FLOW CHART FOR THE REPORTING OF ADVERSE EVENTS

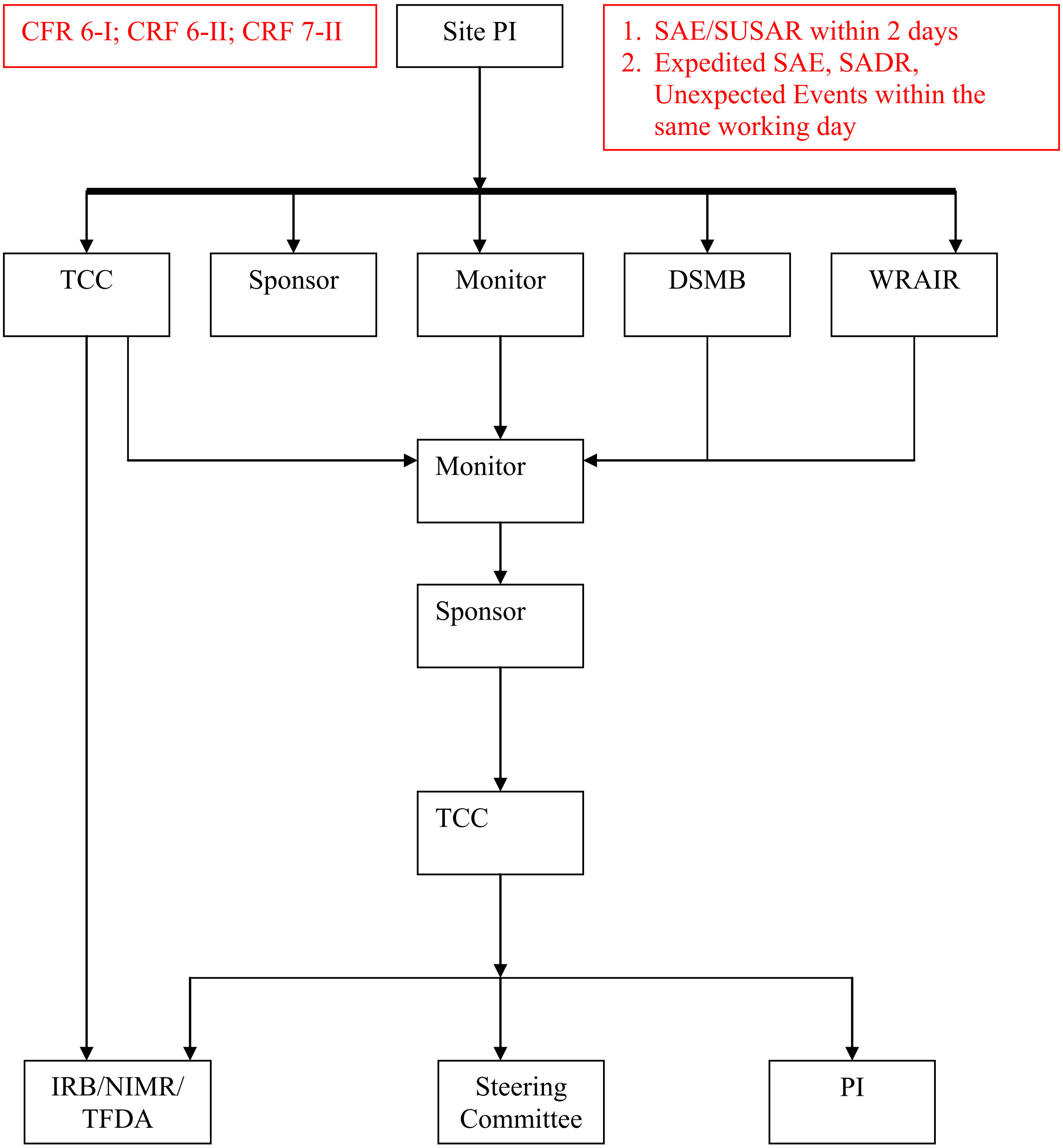

Supplement: S1 File — (ZIP) [file pone.0119629.s001.zip › Supplemental Information/Trial Protocol A.pdf]
