# Supplementary material for: Priming with a Simplified Intradermal HIV-1 DNA Vaccine Regimen followed by Boosting with Recombinant HIV-1 MVA Vaccine Is Safe and Immunogenic: A Phase IIa Randomized Clinical Trial
Source: PLoS One. 2015 Apr 15;10(4):e0119629. doi: 10.1371/journal.pone.0119629 (PMC4398367; doi:10.1371/journal.pone.0119629)
Supplement: S1 File — (ZIP) [file pone.0119629.s001.zip › Supplemental Information/Trial registration A.pdf]

MRC
SOUTH AFRICA
The Medical Research Council

THE COCHRANE COLLABORATION

South African Cochrane Centre

Facilitating the preparation, maintenance and dissemination of systematic reviews of the effects of health care

PO Box 19070, Tygerberg 7505, South Africa
Francie van Zijl Drive, Parow Valley, Cape Town
Tel: +27 21 938 0438, Fax: +27 21 938 0836
E-mail: cochrane@mrc.ac.za

31 May 2010

To Whom It May Concern:

re: TaMoVac-01

As project manager for the PACT Registry database, it is my pleasure to inform you that your application to our registry has been accepted. Your unique identification number for the registry is ATMR2010050002122368.

Please be advised that you are responsible for updating your trial, or for informing us of changes to your trial. Additionally, please provide us with copies of your ethical clearance letters as we must have these on file (via email, postal or fax) at your earliest convenience.

Please do not hesitate to contact us at +27 21 938 0506 or email aabrams@mrc.ac.za should you have any questions.

Yours faithfully,

Amber Abrams
PACT Registry Project Manager

PACTR Registry Account Activation

Sunday, May 16, 2010 3:12 PM

From:

"pactradmin@mrc.ac.za" <pactradmin@mrc.ac.za>
Add sender to Contacts

To:

drbakari@yahoo.com

ATM Registry Registration

Dear MUHAMMAD BAKARI.
Thank you for completing the new user registration form.
To activate your account please click on this link.

Your user ID is : TAMOVAC-01
Your password is: TAMOVAC01

To view/edit your personal details log into the portal
and click on "My profile" in the navigation bar at the top of the page.

Should you forget your password you can click the "forgot your password" in the login section of the home page.

At www.pactr.org

Pan African Clinical Trials Registry

South African Medical Research Council, South African Cochrane Centre
PO Box 19070, Tygerberg, 7505, South Africa
Telephone: +27 21 938 0506 / +27 21 938 0834 Fax: +27 21 938 0836
Email: pactradmin@mrc.ac.za Website: www.pactr.org

Trial no.: WHO	Date registered: WHO 2010/05/23

TRIAL DESCRIPTION	
Public title WHO	TaMoVac-01
Official scientific title WHO	A Phase I/II trial to assess safety and immunogenicity of i.d. DNA priming and i.m. MVA boosting in Healthy volunteers in Tanzania and to develop further HIV vaccine trial capacity building in Tanzania
Brief summary describing the background and objectives of the trial	HIV infection is a public helath priority. Availability of safe and efficacious vaccine is an important desired goal. Participation of developing countries in this effort is crucial. Following earlier investents through Swedish and EU support, a phase I/II trial has been concluded in Tanzania. The DNA-prime, MVA-boost was safe and induced strong cellular immune responses. There is however a need to otpimize DNA delivery, and further build capacity for future efficacy trials in Tanzania
Type of trial	RCT
Acronym (If the trial has an acronym then please provide)	TAMOVAC-01
Disease(s) or condition (s) being studied WHO	HIV/AIDS ,
Purpose of the trial	Prevention
Anticipated trial start date WHO	2010/05/26
Actual trial start date WHO	2010/05/26
Anticipated date of last follow up WHO	2012/11/30
Actual date of last follow up WHO	2012/11/30
Anticipated target sample size (number of participants) WHO	120
Actual target sample size (number of participants) WHO	120
Recruitment status WHO	Open to recruitment: actively recruiting participants

Secondary Ids WHO	Issuing authority/Trial register	Links to Secondary ID

STUDY DESIGN WHO

Intervention assignment	Allocation to intervention	If randomised, describe how the allocation sequence was generated	Describe how the allocation sequence/code was concealed from the person allocating the participants to the intervention arms	Masking	If masking / blinding was used
Factorial: participants	Randomised	Simple	Allocation was determined	Masking/blinding	Outcome Assessors ,

randomly allocated to either no,one,some or all interventions simultaneously		randomisation	by the holder of the sequence who is situated off site	used	Care giver/Provider , Participants ,

INTERVENTIONS[WHO]

Intervention type	Intervention name	Dose	Duration	Intervention description	Group size	Nature of control
Experimental group	Group IIA	600 micrograms of DNA i.d. at weeks 0, 4 and 12; then MVA at 10^8 i.m. at weeks 36 and 60	60 weeks	Low dose, combined plasmids pools DNA	36	
Experimental group	Group IA	600 micrograms of DNA i.d. at weeks 0, 4 and 12; then MVA at 10^8 i.m. at weeks 36 and 60	60 weeks	Low dose, combined plasmids pools DNA	36	
Experimental group	Group IIIA	1,000 micrgrams of DNA i.d. at weeks 0, 4 and 12; then MVA at 10^8 i.m. at weeks 36 and 60	60 weeks	High dose, combined plasmids pools DNA	36	
Control group	Group IB	Saline 2 x 0.1ml i.d. at 0, 4 and 12 weeks, then saline i.m. at 36 and 60 weeks	60 weeks	Saline Placebo	4	Placebo
Control group	Group IIB	Saline 2 x 0.1ml i.d. at 0, 4 and 12 weeks, then saline i.m. at 36 and 60 weeks	60 weeks	Saline Placebo	4	Placebo
Control group	Group IIIB	Saline 5 x 0.1ml i.d. at 0, 4 and 12 weeks, then saline i.m. at 36 and 60 weeks	60 weeks	Saline placebo	4	Placebo

ELIGIBILITY CRITERIA[WHO]

List inclusion criteria	List exclusion criteria	Min age	Max age	Gender
1. Age: 18 to 40 years 2. Willing to undergo counseling and HIV testing 3. Have a negative antigen/antibody ELISA for HIV infection 4. Able to give informed consent 5. Satisfactory completion of an assessment of understanding prior to enrolment defined as 90% correct answers after three opportunities to take test. 6. Basic abilities to read and write. 7. Resident in Dar es Salaam or Mbeya, and willing to remain so for the duration of the study 8. At low risk of HIV infection, defined as the absence of an identifiable risk factor/ behavior 9. Verbal assurances that adequate birth control measures are used not to conceive/father a child during the study and up to 3 months after the last vaccine injection 10. Women shall have a negative urinary pregnancy test 11. Be willing	1. At risk of HIV infection as mentioned above in the inclusion criteria 2. Active tuberculosis or other systemic infectious process elicited by review of systems, physical examination and laboratory detection (for example detection of Hepatitis B surface antigen, or active syphilis). 3. A history of immunodeficiency, chronic illness requiring continuous or frequent medical intervention 4. Autoimmune disease by history and physical examination. 5. Hives or recurrent hives and severe eczema 6. A history of psychiatric, medical (including traditional medicine) and/or substance abuse problems during the past 6 months that the investigator believes would adversely affect the volunteer's ability to participate in the trial. 7. History of grand-mal epilepsy, or currently taking anti-	18 Years	40 Years	Both

to practice safe sex for the duration of the study to avoid sexually transmitted infections including HIV. 12. Good health as determined by medical history, physical examination, clinical judgment and by key laboratory parameters as judged by the study physician.	epileptics 8. Received blood or blood products or immunoglobulins in the past 3 months. 9. Receiving immunosuppressive therapy such as systemic corticosteroids or cancer chemotherapy. 10. Use of experimental therapeutic agents within 30 days of study entry. 11. Reception of any live, attenuated vaccine within 60 days of study entry. {NOTE: Medically indicated subunit or killed vaccines (e.g., Hepatitis A or Hepatitis B) are not exclusionary but should be given at least 2 weeks before or after HIV immunization to avoid potential confusion of adverse reactions}. 12. Abnormality in ECG that could indicate risk or make interpretation of vaccine effects difficult according to the study operating procedures. 13. Previously received an HIV candidate vaccine. 14. History of severe local or general reaction to vaccination in the past 15. Lactating mother 16. Study site employees who are involved in the protocol 17. Ulikeliness of protocol compliance			

ETHICS APPROVAL

Has the study received appropriate ethics committee approval	Date the study will be submitted for approval	Date of approval	Name of the ethics committee
Yes		2009/12/31	National Ethics Committee (NIMR)

Ethics Committee Address

Street address	City	Postal code	Country
Ocean Road	Dar es Salaam	P.O. Box	United Republic of Tanzania

Has the study received appropriate ethics committee approval	Date the study will be submitted for approval	Date of approval	Name of the ethics committee
Yes		2009/11/16	MUHAS IRB

Ethics Committee Address

Street address	City	Postal code	Country
Upanga West, United Nations Road	Dar es Salaam	P.O. Box	United Republic of Tanzania

Has the study received appropriate ethics committee approval	Date the study will be submitted for approval	Date of approval	Name of the ethics committee
Yes		2010/03/22	Mbeya IRB

Ethics Committee Address

Street address	City	Postal code	Country
Mbeya Consultant Hospital	Mbeya	P.O. Box	United Republic of Tanzania

OUTCOMESWHO

Type of outcome	Outcome	Timepoint(s) at which outcome measured

Primary Outcome	Safety	7 days following each vaccination
Primary Outcome	Immunogenicity by IFN-gamma ELISPOT	2 weeks post vaccination
Secondary Outcome	Immunogenicity by intracellular cytokine staining (ICS)	2 weeks post vaccination
Secondary Outcome	Immunogenicity by Lymphoproliferation Assay (LPA)	2 weeks post vaccination

RECRUITMENT CENTRES

Name of recruitment centre	Street address	City	Postal code	Country[WHO]
Muhimbili University of Health and Allied Sciences (MUHAS)	Upanga West, United Nations Road	Dar es Salaam	P.O. Box	United Republic of Tanzania
Mbeya Medical Research Programme (MMRP)	Top Hill	Mbeya	P.O. Box	United Republic of Tanzania

FUNDING SOURCES[WHO]

Name of source	Street address	City	Postal code	Country
European and Developing Countries Clinical Trials Partnership (EDCTP)	334 Laan van Nieuw Osst Indie, 2593 CE	The Hague	2509 AA	Netherlands

SPONSORS[WHO]

Sponsor level	Name	Street address	City	Postal code	Country	Nature of sponsor
Primary Sponsor	Swedish Institute for Infectious Disease Control	Nobels vag 18	Solna	171 82	Sweden	University
Secondary Sponsor	Muhimbili University of Health and Allied Sciences	United Nations Road, Upanga West	Dar es Salaam	P.O. Box 65001	United Republic of Tanzania	University

COLLABORATORS

Name	Street address	City	Postal code	Country
National Institute for Medical Research (NIMR)	Muhimbili, Upanga West	Dar es Salaam	P.O. Box	United Republic of Tanzania
Mbeya Medical Research Programme	Top Hill	Mbeya	P.O. Box	United Republic of Tanzania
Swedish Institute for Medical Research	Nobels vag 18	Solna	171 82	Sweden
Karolinska Institutet	Sjukhusbacke 10	Stockholm	S118 83	Sweden
University of Munich	Geschwister-Scholl-Platz 1, 80539 Munchen	Munich	80802	Germany
Imperial College of Science, Technology and Medicine	Exhibition Road	London	SW7 2AZ	United Kingdom

CONTACT PEOPLE

Role	Name	Email	Phone	Fax
**Principal Investigator** [WHO]	Dr Muhammad Bakari	drbakari@muhas.ac.tz	+255 754 387 328	+255 22 2153027

Street address	City	Postal	Country	Position /

		code		Affiliation
United Nations Road, Upanga West	Dar es Salaam	P.O. Box 65001	United Republic of Tanzania	Senior Lecturer at MUHAS

Role	Name	Email	Phone	Fax
**Public Enquiries** WHO	Dr Muhammad Bakari	drbakari@yahoo.com	+255 754 387328	+255 22 2153027

Street address	City	Postal code	Country	Position / Affiliation
United Nations Road, Upanga West	Dar es Salaam	P.O. Box 65001	United Republic of Tanzania	Senior Lecturer at MUHAS

Role	Name	Email	Phone	Fax
**Scientific Enquiries** WHO	Dr Muhammad Bakari	drbakari@yahoo.com	+255 754 387328	+255 22 2153027

Street address	City	Postal code	Country	Position / Affiliation
United Nations Road, Upanga West	Dar es Salaam	P.O. Box 65001	United Republic of Tanzania	Senior Lecturer at MUHAS

Changes to trial information

Date	Reason	Old Value	Update Value
2010-05-31 06:04:20.0	Modified	RCT	RCT
